# Making the Genome Huge: The Case of *Triatoma delpontei*, a Triatominae Species with More than 50% of Its Genome Full of Satellite DNA

**DOI:** 10.3390/genes14020371

**Published:** 2023-01-31

**Authors:** Pablo Mora, Sebastián Pita, Eugenia E. Montiel, José M. Rico-Porras, Teresa Palomeque, Francisco Panzera, Pedro Lorite

**Affiliations:** 1 Department of Experimental Biology, Genetics Area, University of Jaén, Paraje las Lagunillas s/n, 23071 Jaén, Spain; 2 Evolutionary Genetic Section, Faculty of Science, University of the Republic, Iguá 4225, Montevideo 11400, Uruguay

**Keywords:** Triatominae, Chagas disease vectors, repetitive DNA, satellite DNA, satellitome, genome evolution, fluorescence in situ hybridization

## Abstract

The genome of *Triatoma delpontei* Romaña & Abalos 1947 is the largest within Heteroptera, approximately two to three times greater than other evaluated Heteroptera genomes. Here, the repetitive fraction of the genome was determined and compared with its sister species *Triatoma infestans* Klug 1834, in order to shed light on the karyotypic and genomic evolution of these species. The *T. delpontei* repeatome analysis showed that the most abundant component in its genome is satellite DNA, which makes up more than half of the genome. The *T. delpontei* satellitome includes 160 satellite DNA families, most of them also present in *T. infestans*. In both species, only a few satellite DNA families are overrepresented on the genome. These families are the building blocks of the C-heterochromatic regions. Two of these satellite DNA families that form the heterochromatin are the same in both species. However, there are satellite DNA families highly amplified in the heterochromatin of one species that in the other species are in low abundance and located in the euchromatin. Therefore, the present results depicted the great impact of the satellite DNA sequences in the evolution of Triatominae genomes. Within this scenario, satellitome determination and analysis led to a hypothesis that explains how satDNA sequences have grown on *T. delpontei* to reach its huge genome size within true bugs.

## 1. Introduction

The subfamily Triatominae (Hemiptera: Heteroptera: Reduviidae) comprises a group of more than 150 blood-sucking species distributed in 18 genera, with *Triatoma* Laporte, 1832 being by far the genus with the largest number of species, 82 species [1,2]. These insects act as Chagas disease vectors, which is the most serious human parasitic disease in Latin America, affecting 6–7 million people worldwide [3]. In the absence of vaccines or adequate drugs for large-scale treatment, the reduction in disease burden critically depends on the control of transmission by triatomine vectors [4]. Then, to ensure a successful control campaign, knowledge about these insects’ genetics is an extremely important factor.

In the Southern Cone of South America, the main vector species is *T. infestans*, with a great capability to colonize human dwellings [5]. It is a very polymorphic species regarding ecological and genetic traits. *T. infestans* presents two chromosomal groups, Andean and non-Andean [6,7], which were later supported with nuclear and mitochondrial DNA sequences [8,9] as well as cuticle hydrocarbon patterns [10]. In contrast, the evolutionary-related species *T. delpontei* was much less studied. *T. delpontei* inhabits a relatively small region within the range of *T. infestans* [11]. It is an ornithophilic species, associated with monk parakeets (*Myiopsitta monachus* Boddaert, 1783), living in its colonial nests. Together with *Triatoma platensis* Neiva 1913, also an ornithophilic species, the three species make up the so-called infestans sub-complex (*T. infestans* clade sensu Monteiro et al. [12]), a monophyletic grouping within the South American lineage of the Triatomini tribe. This group of the three-sister species is an early branching clade within the South American lineage [13], and poses striking cytogenetics characteristics. As all Triatominae in particular, and Heteroptera in general, these species bear holocentric chromosomes, characterized by the presence of a diffuse or non-localized centromere [14]. In spite of sharing the same chromosome number (2n = 22 with an XY/XX sex determination system), a great variation was reported regarding the amount and location of constitutive heterochromatin, its base-pair enrichment and the location and number of 45S ribosomal DNA (rDNA) loci [7,15,16,17,18,19,20]. These studies clearly demonstrated that these genomes suffered pronounced changes during their karyotypic evolution. Variability within the infestans sub-complex species also involves striking differences in their genomic sizes. In fact, *T. delpontei* is the species with the greatest C-value in Triatominae, i.e., 2.7 pg [21] and even Heteroptera (Sadílek et al. [22] and references therein).

The employment of other cytogenetic approaches, such as genomic in situ hybridization (GISH) or specific chromosomal probes obtained by chromosome microdissection, evidenced an overall conservation of the repetitive DNA sequences between *T. infestans* and *T. delpontei* [23,24,25]. Genomic probes of one species hybridized on chromosomes of the other one showed that signals were distributed along the C-banded regions. In addition, there were also hybridization signals dispersed in the euchromatic regions [23,26].

Since neither *T. infestans* nor *T. delpontei* genomes are published, the only genome-wide sequence analysis hitherto available on these species is the *T. infestans* repeatome [26]. The term “repeatome” [27] was adopted to include the set of all repetitive DNA sequences composing the genomes, i.e., transposable elements (TEs) (mobilome) and satellite DNA sequences (satDNA) (satellitome). TEs are genetic elements that are able to replicate and move among genomes by themselves. This behavior led to an early view of TEs as selfish DNA sequences that parasitize the host genome. Nowadays, TEs are seen as key players in organism evolution, shaping their genomes [28,29], whereas satDNAs are noncoding, head-to-tail arrays of tandemly repeated DNA sequences that constitute large portions of eukaryotic genomes [30]. As for TEs, currently, satDNA sequences are viewed as important genomic functional components. Aside from the most known functions of centromeric or telomeric repeats, transcripts of satDNAs have been reported in different species, including triatomines [31], highlighting a possible role for satellite ncRNAs in the regulation of gene expression [32,33]. In addition, satDNA rapid turnover has been shown to favor genome plasticity triggering genome evolution, which can sometimes lead to speciation [34,35,36,37].

Satellitome analyses are helpful in the understanding of genome organization and evolution. It is quite frequent nowadays, with some examples on insects from several orders as Coleoptera [38,39], Diptera [35,40], Hemiptera [41], Lepidoptera [42,43], Hymenoptera [44] and Orthoptera [45,46,47], as well as other invertebrates [48,49]. This was led by the cheaper cost of Next Generation Sequencing, together with the development of bioinformatic software [50,51]. RepeatExplorer is one of the most successful examples. A tool that is able to determine the complete repeatome de novo, using low-pass Illumina sequencing data, without the need to perform a genome assembly nor having a reference genome [52,53]. Hence, the tool has been impressively relevant for non-model species, as in the case of *T. infestans*, cited earlier [26]. The *T. infestans* genome is widely populated by satDNA sequences that reach up to nearly 30% of the genome. In fact, these sequences are mainly responsible for genome content variability between Andean and non-Andean lineages, shaping their karyotypic through evolution, since satDNA families build the C-heterochromatic blocks. Remarkably, despite presenting a large number of satDNA families (42), just four families were located on the heterochromatin and comprised the great majority of satDNA content in the genomes. The rest of the satDNA families evaluated by fluorescence in situ hybridization (FISH) were located in the euchromatic regions, and the hybridization pattern resembles those seen in GISH assays. Bearing in mind that GISH analysis revealed a similarity between the overall repetitive content of *T. infestans* and *T. delpontei*, it is probable that at least some satDNA families were shared between both species. In extension, the variation in the genome DNA content between *T. infestans* and *T. delpontei* could be explained by the expansion of similar, or even more, satDNA families in the heterochromatin. To address these ideas, the *T. delpontei* repeatome was determined, with a special focus on its satellitome. In addition, a deeper analysis of the *T. infestans* satellitome was performed in order to better compare it with *T. delpontei*.

## 2. Material and Methods

### 2.1. Samples and DNA Extraction

Individuals of *T. delpontei* were collected in the Rivadavia department from Salta province, Argentina. Genomic DNA was extracted from one male, as in this species, males are the heterogametic sex, in order to retrieve information on all the chromosomes, including the X and Y. For DNA extraction, the head and leg muscles were used using a commercial kit (Gentra Puregene Qiagen, Hilden, Germany) according to the manufacturer’s guidelines. For chromosome preparations, males from several localities from Argentina and Uruguay were dissected, and the testes were preserved in ethanol: glacial acetic acid (3:1) and stored at −20 °C.

### 2.2. Genome Sequencing and Graph-Based Clustering of Sequencing Reads

Genomic DNA was sequenced using the Illumina HiSeq 2500 platform at Macrogen. The low-coverage sequencing yielded around 2.2 Gb of 101 bp paired-end reads. Raw reads data were quality trimmed, and adaptors were removed using Trimmomatic (v.0.36) [54]. Fastq files were modified to fasta using seqtk version 1.3-r106 (https://github.com/lh3/seqtk, accessed on 5 September 2022). The first step in the repeatome characterization was to run the RepeatExplorer2 pipeline [53,55], including the TAREAN analysis within the Galaxy portal (https://repeatexplorer-elixir.cerit-sc.cz, accessed on 6 September 2022). For this analysis, we randomly selected a total of 16,000,000 paired reads. Default options were selected except the computing time (extra-long), the filtration of the most abundant repeats and the threshold of the analysis to 0.001%. The repeatome annotation was performed in those clusters that represented more than 0.001% of the genome (top clusters). In the analysis, a custom database with the satDNA families of *T. infestans* [26] and *Rhodnius prolixus* Stål, 1859 [31] was used in order to test the presence of these satDNAs in *T. delpontei* genome. This was also aided by a custom database containing TEs of *T. infestans* and *R. prolixus*. Cluster reads with similarity with the *T. infestans* satDNAs and those identified as satDNAs by TAREAN were deeply analyzed. Other clusters with satDNA-compatible graphs were also analyzed. For each cluster, the contig with the highest number of reads was used to produce a dot plot with Geneious Pro v.4.8.5. (https://www.geneious.com, accessed on 23 November 2022) in order to determine the existence of tandem repeats and to estimate the size of the repeat unit. All reads containing satDNA sequences in the clusters were aligned using Geneious Pro v.4.8.5. and then a consensus sequence from each family was created. Once all consensus sequences were retrieved, a BLAST “all-to-all” was performed using blast and -e value of 0.001 in order to find similarities among the satDNA families. Moreover, abundance and divergence of each satDNA family were calculated using the RepeatMasker tool (http://www.repeatmasker.org, accessed on 9 December 2022), keeping the alignment (“-a” option) and using the RMBlast search engine. A total of 5,000,000 randomly selected reads were used for this analysis, and then aligned back to the total collection of satDNA dimers (for those families with a repetition unit length larger than 100 bp) or a concatenation of at least 200 bp for those families with a repeat length smaller than 100 bp. Satellite DNA landscapes (abundance vs. divergence) were created by applying the Kimura 2-parameter (K-2p) model using the perl script calcDivergenceFromAlign.pl from the RepeatMasker suite. The plot was made in R version 4.2.0 [56], with the ggplot2 package [57].

For a deeper comparison with the sister species *T. infestans*, a re-analysis of its satellitome was performed using the available data from Pita et al. [26] but applying the same parameters used here for *T. delpontei*. In the new RepeatExplorer2 analysis, a custom database with all the satDNA families previously described in *T. infestans* [26], together with all satDNAs found in *T. delpontei*, was used.

For comparative analysis between species, the profile of each satDNA family was generated in both species over raw paired-end reads, using the RepeatProfiler tool [58]. This tool is used to create the coverage and base pair composition profile using the data from Illumina sequencing. As reference, we concatenated monomers into trimers (or at least monomers up to 200 bp). The default options were selected, with the “-p” option in order to take as input the paired-end reads [58].

### 2.3. Chromosome Preparation and Physical Mapping by Fluorescence In Situ Hybridization

Chromosome plates were obtained from fixed testes of *T. delpontei*, as described in Pita et al. [26]. The consensus sequences from the selected satDNA families were used in order to design a set of oligonucleotides that were biotin-16-dUTP (Roche, Manheim, Germany) labeled using terminal transferase (Roche). Labeled oligonucleotides (Table 1) were used as probes for FISH (final concentration of 5 ng/mL in 50% formamide) [26,59]. The fluorescent immunological detection was performed using the avidin-FITC/anti-avidin-biotin system with two amplification rounds. After that, the slides were counterstained using Vectashield-DAPI (Vector Laboratories, Burlingame, CA, USA). Images were taken with a BX51 Olympus^®^ fluorescence microscope (Olympus, Hamburg, Germany) equipped with a CCD camera (Olympus^®^ DP70) and processed using Adobe^®^ Photoshop^®^ software (Adobe Systems, San Jose, CA, USA).

## 3. Results

**Figure 1:**
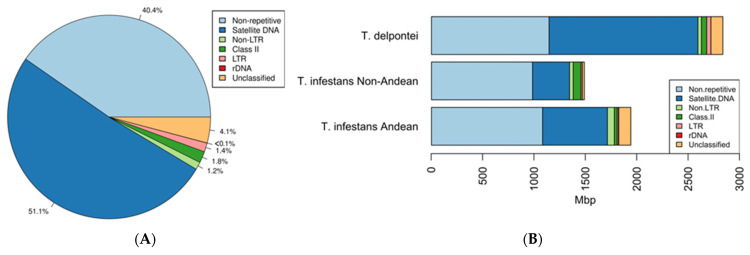
(**A**) *T. delpontei* genome composition. Percentages were calculated according to RepeatExplorer2 results. (**B**) Comparative chart with the composition between *T. delpontei* and both *T.a infestans* lineages genomes. All values were calculated according to RepeatExplorer2 results. The *T. infestans* data were taken from Pita et al. [26].

Low-coverage sequencing of the *T. delpontei* genome was performed, obtaining 8 M paired-end 101 bp reads after quality filtering and trimming the pipeline. RepeatExplorer2 clustering analysis retrieved 1047 clusters, with at least 20 reads within them. Clusters identified as mitochondrial DNA sequences were left aside from the final annotation. Highly repetitive or top clusters corresponded to 59.64% of the total genome. Cluster annotation led to the classification into five categories: long terminal repeats (LTR), non-long terminal repeats (non-LTR), class II elements, satellite DNA (satDNA) and ribosomal DNA (rDNA). Those unable to determine their identities were labeled as undetermined repeat (unclassified) (Figure 1A). In order of abundance, the most frequent category was satDNA (51.07%), followed by class II elements (1.81%), LTR (1.42%), non-LTR (including Penelope elements to simplify 1.21%) and rDNA (0.02%). Comparative analysis with *T. infestans* (Andean and non-Andean lineages) showed that the repeatome of all taxa is built with satDNA as the major repeat fraction, being these sequences responsible for the differentiation in genome size between species (Figure 1B).

Due to the great amount of satDNA, special emphasis was placed on the satellitome determination. A deeper search on clusters led to the identification of 160 satDNA families in the *T. delpontei* genome (Table 2, Supplementary Table S1). Satellite DNA consensus sequences were deposited in the NCBI database (Acc. Numbers OQ82080-OQ82236). The satDNA families were named following Ruiz-Ruano et al. [45], numbered according to their abundance in the genome, and including the length of the repeat sequence (TdelSat01-79 to TdelSat160-49). Monomeric repeat length in *T. delpontei* showed variation from 4 bp (TdelSat13-4-CATA) to 1000 bp (TdelSat05-1000), with most of them having less than 200 bp (Supplementary Table S1). The A+T content in the consensus satDNA families ranged between 41% (TdelSat59-63) and 86% (TdelSat111-7), and the A+T richness in most of them was higher than 50%. Comparative analysis among consensus sequences from all satDNAs families revealed that most of the satDNA families did not show similarities among them. However, between several satDNAs, it was possible to find the existence of regions with high similarity, suggesting that they are evolutionarily related (Supplementary Figure S1). Most of the similarities were found when comparing TdelSat05-1000, which has conserved regions with the other 11 satDNA families.

The deeper *T. infestans* satellitome analysis was able to characterize 54 new families (Acc. Numbers OQ82237-OQ82289), together with the 42 previously already described [26], resulting in a total of 96 satDNA families in the *T. infestans* genome (Supplementary Table S2). In addition, the amount of all satDNAs in *T. infestans* Andean and non-Andean lineages were now quantified using RepeatMasker. Previously, the proportion of each satellite was calculated based on the number of reads in the clusters obtained with RepeatExplorer [26]. The new satDNAs were numbered according to their abundance in the Andean lineage, according to the RepeatMasker results. The new analysis showed some differences in the estimation for the previously identified 42 satDNAs, but we have kept the previous names in order to avoid pitfalls. Most of the 96 satDNAs were present in both lineages (Supplementary Table S2). Two satDNAs were detected only in the Andean lineage, and three families only in the non-Andean lineage. The use of RepeatMasker allowed the determination that 51 of these 96 *T. infestans* satDNAs were also present in *T. delpontei* (Table 2), although their abundance in both species may be different.

The satDNA families shared between *T. delpontei* and *T. infestans* are highly conserved in the nucleotide sequence. In fact, for many satDNAs, the consensus sequences were the same in both species or had very high similarity. The greatest differences were observed in three satDNA families: TinfSat03-4 vs. TdelSat02-8, TinfSat09-113 vs. TdelSat06-25 and TdelSat14-94 vs. TinfSat45-94 (Supplementary Figure S2). In *T. infestans*, the TinfSat03-4 satDNA is organized as (GATA)_n_ arrays where it is possible to find some variants of this sequence, such as GATAGTTA, GATAGATTA or GATAGGTA (Pita et al. 2017a). However, in *T. delpontei*, the satDNA TdelSat02-8 is mainly organized by tandem repetition of the sequence (GATAGTTA)_n_. Therefore, this sequence of eight nucleotides, GATAGTTA, should be considered the consensus sequence of this satDNA in *T. delpontei* (TdelSat02-8). Notwithstanding, since GATA and GATAGTTA repeats are intermixed in the reads, and the similarity between the sequences is 80% (GATAGATA vs. GATAGTTA), it could be considered as the same satDNA. TinfSat09-113 is a higher-order repeat (HOR) originating from a 25 bp sequence with duplications and insertions [26]. In *T. delpontei*, TdelSat06-25 forms arrays with this 25 bp sequence, also with variations by duplications and insertions, but does not form an HOR as in *T. infestans*. TdelSat14-94 vs. TinfSat45-94 have only 60% similarity but contain a 38 bp region with a 100% similarity. The presence of this sequence could indicate that they are evolutionarily related, although their sequences have diverged significantly between the two species.

Both satellitomes presented here were compared with another triatomine species *R. prolixus* [31], which belongs to the Rhodniini tribe. Instead, species from the genus *Triatoma* are included in the Triatomini tribe. A previous study showed that this species shared six satDNAs with *T. infestans* [31], among them the telomeric (TTAGG)_n_ and (GATA)_n_ repeats. The new satellitome analysis carried out in *T. infestans* has determined the existence of a new shared satDNA family between both species (TinfSat43-242 vs. RproSat05-208). The seven satDNAs are also present in *T. delpontei* (Table 2).

**Figure 2:**
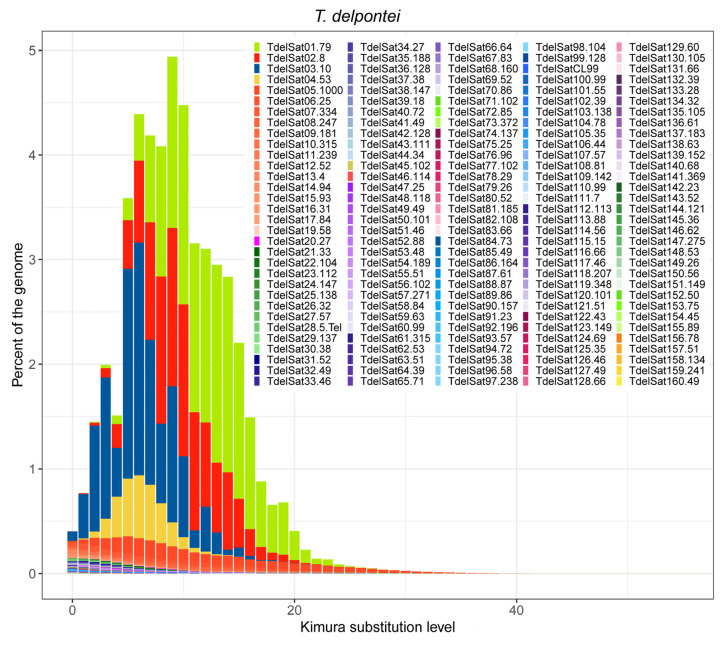
*T. delpontei* satellite DNA landscape.

**Figure 3:**
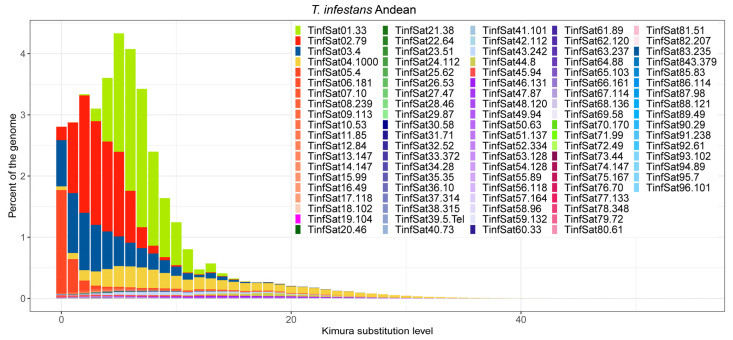

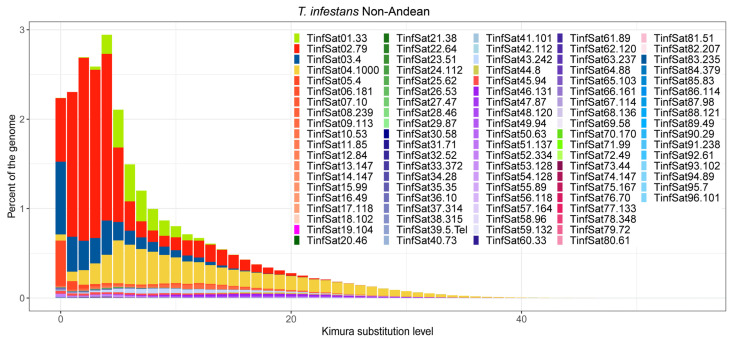
Satellite DNA landscapes for Andean and non-Andean *T. infestans*.

**Figure 4:**
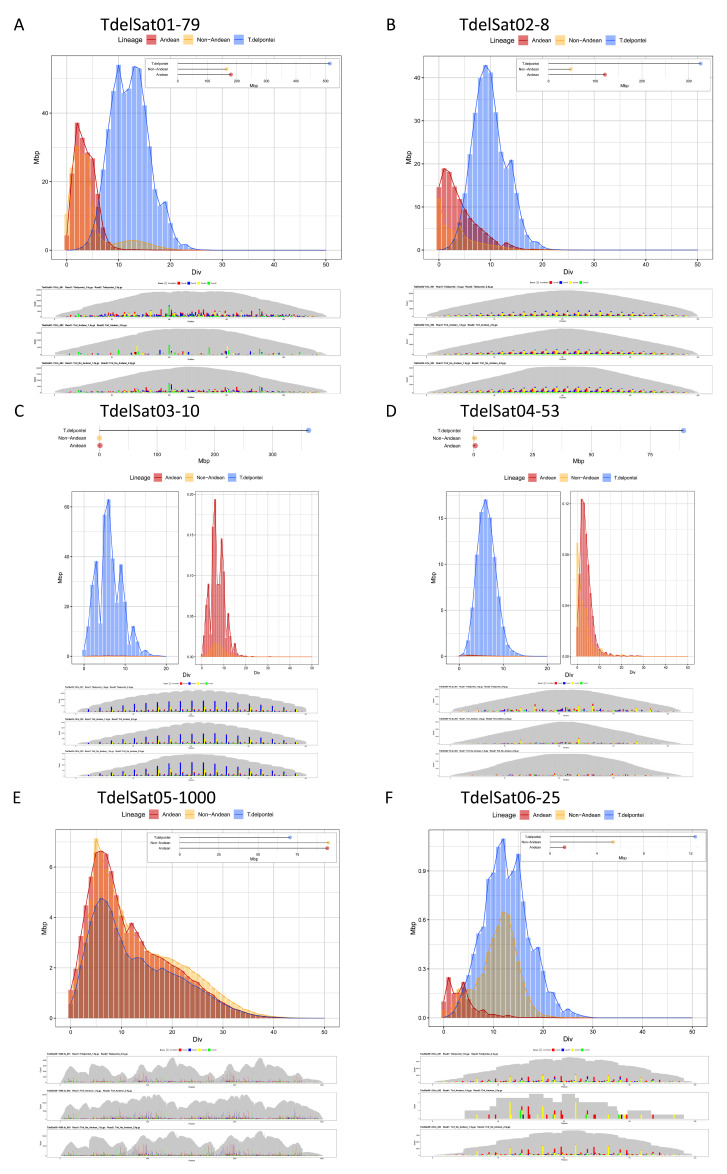
Results of the most abundant satDNAs in *T. delpontei*, Andean and non-Andean *T. infestans* lineages: (**A**) TdelSat01-79, (**B**) TdelSat02-8, (**C**) TdelSat03-10, (**D**) TdelSat04-53, (**E**) TdelSat05-1000 and (**F**) TdelSat06-25. Above: satellite abundance (Mb) estimated by RepeatMasker using each species consensus in the three analyzed genomes, and satellite DNA landscape graphics (abundance in Mb vs. divergence in percentage). Each calculation was performed as above using each species’ consensus over their own genome reads. Bottom: RepeatProfiler variation analyses over the three genomes analyzed. The consensus sequences of *T. delpontei* were used.

The divergence of each satDNA family was calculated using RepeatMasker. In *T. delpontei*, divergence values ranged from 0.04% (TdelSat85-49) to 24.02% (TdelSat25-138), showing a median value of 5.62% (Supplementary Table S1). A similar range was found for Andean and non-Andean *T. infestans* (Supplementary Table S2) as well as in *R. prolixus* [31]. In non-Andean *T. infestans*, the values ranged from 1.29% to 23.45%, with a median of 9.29%. The lowest value corresponds to TinfSat05-4, and the highest one was for the TinfSat18-102 family. While in the Andean *T. infestans* lineage, the values ranged from 0.64% (TinfSat05-4) to 19.53% (TinfSat12-84), the median value is almost the same as the non-Andean lineage, at 8.55%. In *R. prolixus*, the nucleotide divergence of all satDNA families ranged between 0.88% (RproSat32-59) and 28.28% (RproSat13-293), with a median value of 10.03% [31]. Notwithstanding, the satellite DNA landscape curve reflects that overall, *T. delpontei* has a peak at 9% diversity (x-axis on Figure 2), while *T. infestans* shows the curve peak at 4% and 5% in non-Andean and Andean, respectively (Figure 3). In addition, the curve shape is completely different since *T. infestans* has a positively skewed distribution. This could be easily explained by looking at the most abundant family in *T. delpontei*, TdelSat01-79, also one of the highest abundances in *T. infestans* (TinfSat02-79). While in *T. delpontei*, this family has a great divergence (12.73%), with a peak at 10% in the satellite DNA landscape (Figure 4), in *T. infestans*, the divergence was 4.79% and 3.71%, with satellite DNA landscape peaks of 2% and 6% in non-Andean and Andean *T. infestans*, respectively, showing a much more conserved family (Figure 4). The data for all satDNA families are depicted in Supplementary Tables S1 and S2. Individual satellite DNA landscapes for each satDNA family are shown in Supplementary Figures S3–S5.

As commented above, the satDNAs shared between *T. delpontei* and *T. infestans* present very different amounts in the genomes of both species. Figure 4 shows the satellite DNA landscape graphics and absolute amounts for the most abundant satDNAs in *T. delpontei*, as well as the same data for these satDNAs in Andean and non-Andean *T. infestans* lineages. The main satDNA of *T. delpontei* (TdelSat01-79, 18.16% genome) was also present in *T. infestans* (TinfSat02-79), where it also represents an important part of the Andean lineage genome (9.30%), being also the main satDNA in the non-Andean lineage (11.13%). Due to the big differences in the C-value between these genomes (*T. delpontei* 2836 Mb, Andean *T. infestans* 1936 Mb, non-Andean *T. infestans* 1487 Mb), the comparisons between absolute amounts of each satDNA were performed (Figure 4A). In both *T. infestans* lineages, the amounts of TinfSat02-79 are between 150 and 200 Mb. However, TdelSat01-79 satDNA has been significantly amplified in the *T. delpontei* genome, with more than 500 Mb. GATA repeats (TdelSat02-8 vs. TinfSat03-4) are highly amplified in the *Triatoma* species, representing 14.21% of the *T. delpontei* genome and 3.20% and 6.24% of the *T. infestans* genome (non-Andean and Andean, respectively). These results indicate that GATA repeats were intensely amplified in the Andean *T. infestans* genome (above 150 Mb) in relation to non-Andean *T. infestans* (above 50 Mb), and especially in *T. delpontei* (above 250 Mb) (Figure 4B). Other highly amplified satDNAs in *T. delpontei* appear in very low proportion in *T. infestans*. For example, TdelSat03-10 and TdelSat04-53 represent 12.79% and 3.15% of the *T. delpontei* genome (about 360 and 90 Mb, respectively), but their equivalents in *T. infestans* do not reach 0.1% of the genome (Figure 4C,D). There is not much difference, in terms of absolute amount, for TdelSat05-1000 vs. TinfSat04-1000. This satDNA is slightly amplified in the *T. infestans* genome in comparison with *T. delpontei*. In *T. delpontei*, this satDNA family represents 2.50% genome (71 Mb) and 6.47–4.93% (96 and 95 Mb) in *T. infestans* (non-Andean and Andean, respectively) (Figure 4E). For the next most abundant satDNA (TdelSat06-25 vs. Tinf09-113), there are important differences between the two species (Figure 4F). The amount of this satDNA in *T. delpontei* (0.437%, 12.4 Mb) is 10-fold the amount in Andean *T. infestans* (0.064%, 1.23 Mb) and more than 2-fold in non-Andean *T. infestans* (0.358%, 5.32 Mb).

*T. delpontei* consensus sequences from all satDNA families were included in the RepeatProfiler analysis. This analysis allows the visualization of coverage depth profiles for repetitive sequences and the profile sequence variation in relation to a consensus sequence. Profiles for all satDNAs are shown in Supplementary Figure S6. This analysis determined that the number of shared satDNAs between *T. delpontei* and *T. infestans* is much higher than the 51 families detected with RepeatExplorer. In fact, most of the satellites are present in both species and within *T. infestans* in both lineages. RepeatProfiler results determined that another additional 68 satDNA families are also shared between *T. delpontei* and *T. infestans*, raising the number of shared families to 119. RepeatProfiler analysis showed that for these 68 satDNA families, there are strong differences in their abundance between both species. The low abundance of these satDNAs in *T. infestans* may explain why they have not been detected with RepeatExplorer, where only repeats with abundances higher than 0.001% were selected. RepeatMasker analysis failure could be due to the use of a small reads sample to evaluate the genome. In addition, software differences in the performance to identify repeats in the genome could be affecting this. It cannot be ruled out that the 41 satDNA families that seem to be present only in *T. delpontei* could be, in fact, also present in *T. infestans*, but in such a small abundance that it was not possible to detect them with RepeatProfiler either. Figure 4 shows the satellite DNA landscape and RepeatProfiler results for the six most abundant satDNAs of *T. delpontei* and for the equivalent satDNAs in *T. infestans*. The satellite DNA landscape of TdelSat01-79 vs. TinfSat02-79 shows that this satDNA is more variable in *T. delpontei* than in *T. infestans*. RepeatProfiler shows the existence of a large number of variable positions along the repeat sequence for *T. delpontei*, while the sequence in *T. infestans* was more conserved, with both lineages showing a similar pattern of variation. These results are in concordance with the diversity values estimated for these satDNAs: 12.73% for TdelSat01-79, 3.71% and 4.79% for Andean and non-Andean TinfSat02-79 (Supplementary Tables S1 and S2). Similar results were obtained for other satDNA families, such as TdelSat04-53 (Figure 4D). The RepeatProfiler analyses showed that for some satDNAs, the nucleotide variation is similar in both species, as happens with TdelSat02-8, TdelSat03-10, TdelSat05-1000 or TdelSat06-25 (Figure 4B,C,E,F), in spite of the different amounts in both species.

**Figure 5:**
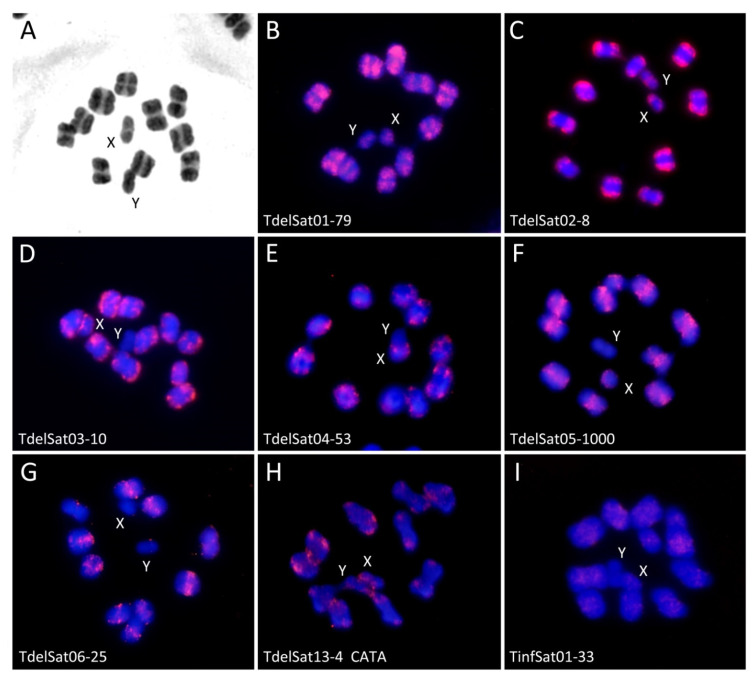
C-banding patterns and satDNA families’ hybridization observed in first meiotic metaphases of *T. delpontei*. (**A**) C-banding with Giemsa, showing the presence of heterochromatic blocks at one chromosome end of all autosomes and both sex chromosomes. (**B**–**I**) Meiotic metaphases I after hybridization using different satDNAs as probes: TdelSat01-79 (**B**), TdelSat02-8 (**C**), TdelSat03-10 (**D**), TdelSat04-53 (**E**), TdelSat05-1000 (**F**), TdelSat06-25 (**G**), TdelSat13-4 (**H**) and TinfSat01-33 (**I**).

As previously reported [15], the C-banding pattern in *T. delpontei* showed the presence of heterochromatic blocks in one chromosomal end of all autosomes and the X chromosome, which reach almost half of the chromosome length (Figure 5A), while the Y chromosome is entirely heterochromatic. However, *T. infestans* autosomes showed prominent C-heterochromatic regions at both ends. This pattern varies depending on the lineage. Andean lineage bears 7 to 10 autosomal pairs plus the X chromosome with C-heterochromatin. Non-Andean lineage has only three pairs of autosomes with positive C-banded regions. The Y chromosome is entirely heterochromatic in both lineages [6,7,26]. Major satDNA families (TdelSat01-79 to TdelSat06-25) were physically mapped on *T. delpontei* chromosomes. The two main satDNA families (TdelSat01-79, TdelSat02-4) were localized on the heterochromatin in both autosomes and sex chromosomes (Figure 5B,C). TdelSat03-10 and TdelSat04-53 were also the building blocks of the autosomes heterochromatin and the X chromosome, but were not present in the Y chromosome (Figure 5D,E). TdelSat05-1000 and TdelSat06-25 were restricted to the euchromatic regions (Figure 5F,G). In addition, FISH with the other two satDNAs was carried out. The first one was TinfSat01-33, the main satDNA in *T. infestans*. Results showed that this satDNA was located on the euchromatic regions of the autosomes and on the X chromosome (Figure 5I). The second one was CATA repeats (TdelSat13-4). In *T. infestans*, this repeat (TinfSat05-4) is located on the heterochromatic regions of the autosomes. In *T. delpontei*, this satDNA is also present on the autosome heterochromatin, but also on the heterochromatin of both sex chromosomes (Figure 5H). It is noteworthy that, for satDNAs located mainly on the heterochromatin, less intense hybridization signals were also observed in the autosomal euchromatic regions.

## 4. Discussion

Hitherto, repetitive DNA in triatomines has been analyzed in just a few species [26,31,60,61]. The *T. delpontei* repeatome analysis showed that the most abundant component in its genome is repetitive DNA. A similar situation is observed in other invertebrate genomes where the repetitive fraction represents nearly or more than half of their genomes, such as *Tenebrio molitor* Linnaeus, 1758 (Coleoptera: Tenebrionidae) [62], *Pontastacus leptodactylus* Eschscholtz, 1823 (Decapoda: Astacidae) [49], *Octopus vulgaris* Cuvier, 1797, *Octopus bimaculoides* Pickford & McConnaughey, 1949 or *Architeuthis dux* Steenstrup, 1857 (Cephalopoda: Coleoidea) [63]. In the case of *T. delpontei*, this scenario was expected, since C-banding reveals that half of the autosomal chromosomes bear heterochromatin, as well as half of the X and the entire Y chromosome. However, a striking and unique result was that more than half of the genome is represented only by satDNA sequences (Figure 1). As said before, four satDNA families, included in the heterochromatin, represent more than 48% of the *T. delpontei* genome.

Regarding the *Triatoma* genus, the present results depicted the great importance of the satDNA sequences in genome evolution. Comparison between sister species, such as between *T. delpontei* and both lineages of *T. infestans*, showed that satDNA is the main repetitive fraction of their genomes. Additionally, a detailed analysis of this fraction allowed the determination that within the infestans sub-complex species, the heterochromatin is mainly formed by only a few different satDNA families. This trend has also been reported for other Heteroptera species, such as *Holhymenia histrio* Fabricius, 1803 [41], as well as in other insect groups, such as Coleoptera [38,39,64] or Hymenoptera [44,65]. The results show that the large genome size of *T. delpontei* is mainly due to the significant increase in some families of satDNA sequences, which turned out to be the largest genome so far reported in Heteroptera (Sadilek et al. [22] and references there). A similar situation was observed in *T. infestans*, where few satDNAs families mainly located in the heterochromatin were responsible for the variation in genomic DNA content between *T. infestans* lineages [26]. Overall, whether this pattern is universal for the *Triatoma* genus or not is yet to be determined. Future analyses of other species are needed to test this hypothesis.

*T. delpontei* heterochromatin is formed mainly by just four satDNA families. Two of these satDNAs are present in the heterochromatin of both species, TdelSat01-79 and TdelSat02-8, although their abundances is significantly higher in *T. delpontei* with respect to their homologs in *T. infestans* (TinfSat02-79 and TinfSat03-4) (Figure 4). The other two abundant satDNAs in the *T. delpontei* heterochromatin (TdelSat03-10, TdelSat04-53) were also found in the genome of *T. infestans* (TinfSat07-10/36-10 and TinfSat10-53), although in the latter species, their abundances were much lower and they were located on the euchromatin. Conversely, there are also satDNAs located in the euchromatin in *T. delpontei* that were part of the heterochromatin in *T. infestans*, as TinfSat01-33. It is important to note that neither RepeatExplorer, RepeatMasker nor RepeatProfiler were able to detect TinfSat01-33 in the *T. delpontei* NSG reads. However, we were able to map this satDNA family by FISH (Figure 5I). This could be due to inter-population variability within *T. delpontei*, but we cannot rule out the hypothesis that the amount or the divergence within this satDNA in the *T. delpontei* genome could hamper the detection by the software. For example, this satDNA could be in a scarce amount in the genome, which would be under the detection limit, or within the limit of the low-pass genome sequencing. It is also possible that the existence of an elevated nucleotide divergence in *T. delpontei* in relation to *T. infestans* might avoid the detection of satDNA by the used software. Lastly, it could also be possible that a similar sequence to TinfSat01-33, belonging to another repetitive element (i.e., a TE), was responsible for this FISH result.

Interestingly, CATA repeats are present in the genomes of *T. infestans* and *T. delpontei* (TdelSat13-4 vs. TinfSat05-4), but there were important differences with respect to their abundance and chromosomal location. In *T. infestans*, this satDNA was located on the heterochromatin of the autosomal bivalents [26]. In *T. delpontei*, CATA repeats have also spread in the heterochromatin of all autosomes. Interestingly, it is noteworthy that in *T. delpontei*, the extension of CATA repeats has also involved the sex chromosomes.

*T. delpontei* and *T. infestans* share most of their satDNA families, although in each species their abundances are different. The number of shared families is lower when it is compared with genomes of species from other genera, such as *R. prolixus*. These results are in accordance with the postulations of the “library hypothesis”, which states that related species share a set of ancestral satDNA families. Hence, closely related species would tend to share a larger proportion of satDNA families than more distant ones [66,67]. According to the FISH results in triatomines, the satDNAs with low abundance are dispersed in the euchromatin. In addition, even satDNAs that build up the heterochromatin are located in euchromatic regions. These “euchromatic” satDNAs would probably be organized in arrays with fewer copies. Hence, it is probable that this set of repetitive sequences dispersed in the euchromatin would form the satDNA “library”. Stochastic mechanisms would move some of these satDNAs to the heterochromatin, where they could be highly amplified. This might have occurred with the TdelSat03-10 and TdelSat04-53 families, which are abundant in the *T. delpontei* heterochromatin but are in low proportion in the euchromatin of *T. infestans*. The other way around happened with TinfSat01-33, which is the main satDNA of *T. infestans*, but is in a much lower proportion in the euchromatin of *T. delpontei*.

It is also possible to observe large differences in the amount of satDNA located on the chromosomal euchromatic regions, as happens with TdelSat05-1000 vs. TinfSat04-1000 or TdelSat06-25 vs. TinfSat09-113 (Figure 4E,F). There are no data to explain the cause of why a given satDNA family increases or decreases its proportion in the genome. It is possible that mechanisms that generate abundance variations for other tandem repeat sequences, such as unequal crossing over or replication slippage, were responsible for such variation. It cannot be ruled out that mechanisms such as ectopic recombination are in relation to the observation of stick chromatin during *T. delpontei* and *T. infestans* meiosis (Panzera et al. 1995). Variations in the amount of a particular satDNA family may occur between species, but also at the population level, as was observed between the Andean and non-Andean *T. infestans* lineages [26]. These intraspecific differences have been observed in other insect groups [39,45,68,69,70].

In order to obtain a better picture of satDNA dynamics between species, DNA landscape comparisons lead to the observation that the *T. delpontei* genome possesses a higher peak of divergence (9%) than *T. infestans* (4% and 5% in non-Andean and Andean, respectively). The most abundant satDNA family in *T. delpontei* (TdelSat01-79) is highly responsible for this trait (see results section). In spite of presenting clustered repeats that tend to be homogenized, extremely large arrays in *T. delpontei* could be hampering the homogenization process. Therefore, mutations could be accumulated at the extremes of the arrays and increment the divergence. Notwithstanding, the median divergence value of *T. delpontei* (5.62%) is lower than other insects [38,39,45,71,72], including both *T. infestans* lineages (9.29% and 8.55% non-Andean and Andean, respectively) and the variable *R. prolixus* satellitome, with a median value of 10.03% [31]. The divergence values of satDNAs are directly related to mutation rate and inversely related to amplification and homogenization [73,74]; hence, the lower value present in the *T. delpontei* satellitome means that the *T. delpontei* genome would be more prone to homogenization processes than both *T. infestans* lineages, probably due to the presence of larger arrays within the euchromatic regions.

*T. delpontei* genome is then constructed by a huge amount of satDNA sequences, constituting more than half of it. The difference with other *Triatoma* species is around double the C-value. Most *Triatoma* species have a genome size of around 1222 Mb, while that of *T. delpontei* is 2836 Mb [20,21]. Within this scenario, satellitome determination and analysis could lead to a hypothesis that explains how satDNA sequences have grown. Interestingly, the *T. infestans* genome presented a singular variability in its DNA content, presenting a non-Andean 1487 Mb genome and an Andean 1936 Mb genome. Hence, *T. infestans* variation could be taken as an intermediate step to the configuration of the outstanding *T. delpontei* genome. As commented above, all three genomes share a common trait in which satDNA is the major component of the repetitive DNA. Furthermore, satDNA families are mostly shared and are responsible for the size variation. Interestingly, satDNA families that invaded the heterochromatin and were highly amplified led to the hypothesis that the genomic environment on subtelomeric regions of *Triatoma* could be influencing this phenomenon. Therefore, repeats that reach subtelomeres are prone to be involved in an expansion process. Ectopic recombination between non-homologous subtelomeres could enhance this process, spreading the repeats. Given the importance of repetitive sequences on the *Triatoma* karyotypic evolution, further investigation is essential. What is more, this topic could shed light to elucidate the mechanisms involved in the speciation processes within *Triatoma*, a specious genus within Triatominae, encompassing 82 out of the more than 150 described species.

## Figures and Tables

**Table 1:** Oligonucleotides used for FISH analyses in *T. delpontei*.

SatDNA Family	Oligonucleotide *	Sequence
TdelSat01-79	TinfSat02-79-F	5′-TTGTAAGGTTCAAGAAAATCCC
	TinfSat02-79-R	5′-CTCACTCTTACGGTTGAAACGC
TdelSat02-8	(GATA)5	5′-GATAGATAGATAGATAGATA
TdelSat03-10	TinfSat07-10	5′-RCATACTCGKRCATACTCGK
TdelSat04-53	TinfSat10-53-F	5′-CGGTTTTGGTTATACTATTTTTCCA
	TinfSat10-53-R	5′-GAAGGGGCAAACGTGTATT
TdelSat05-1000	TinfSat04-1000-F	5′-GATATCGAAAATTTGACACG
	TinfSat04-1000-R	5′-ATGTATGTGAACAGCATAGC
TdelSat06-25	TinfSat09-113	5′-AGAATGTAKAACTTTG
TdelSat13-4	(CATA)5	5′-CATACATACATACATACATA
TinfSat01-33	TinfSat01-33	5′-TTTCCATAAGTCTATTACTTCGTAATTACTGCG

* Previously designed primers for *T. infestans* [26] were used.

**Table 2:** Data of the satDNA families found in *T. delpontei*: name, genome abundance in *T. delpontei*, presence in *T. infestans*, and genome abundance in the Andean and non-Andean *T. infestans* lineages.

Name	Genome Abundance (%)	Similarity with *T. infestans*	Genome Abundance in *T. infestans* (%)	
			Andean	Non-Andean
TdelSat01-79	18.158	TinfSat02-79	9.300	11.127
TdelSat02-8 (GATAGTTA)	14.21	TinfSat03-4	6.236	3.202
TdelSat03-10	12.786	TinfSat07-10 /36-10	0.066	0.01
TdelSat04-53	3.146	TinfSat10-53	0.033	0.024
TdelSat05-1000	2.502	TinfSat04-1000	4.927	6.473
TdelSat06-25	0.437	TinfSat09-113	0.064	0.358
TdelSat07-334	0.410	TinfSat52-334	0.028	0.150
TdelSat08-247	0.303	TinfSat43-242	0.697	0.697
TdelSat09-181	0.173	TinfSat06-181	0.204	0.129
TdelSat10-315	0.117	TinfSat37-314	N.D.	0.037
TdelSat11-239	0.092	TinfSat08-239	0.196	0.184
TdelSat12-52	0.086	TinfSat32-52	0.008	0.012
TdelSat13-4 (CATA)	0.056	TinfSat05-4	2.627	0.734
TdelSat14-94	0.053	TinfSat45-94	0.318	0.342
TdelSat15-93	0.053	TinfSat49-94	0.033	0.045
TdelSat16-31	0.053	N.D.		
TdelSat17-84	0.045	TinfSat12-84	0.049	0.047
TdelSat18-62	0.040	TinfSat25-62	0.012	0.019
TdelSat19-58	0.040	TinfSat30-58	0.013	0.005
TdelSat20-27	0.035	R.P.		
TdelSat21-33	0.034	TinfSat60-33	0.014	0.015
TdelSat22-104	0.033	TinfSat19-104	0.020	0.002
TdelSat23-112	0.031	TinfSat42-112	0.014	0.017
TdelSat24-147	0.030	TinfSat14-147	0.031	0.032
TdelSat25-138	0.028	N.D.		
TdelSat26-32	0.028	N.D.		
TdelSat27-57	0.026	N.D.		
TdelSat28-5 telomere	0.024	TinfSat39-5	0.021	0.011
TdelSat29-137	0.023	TinfSat51-137	0.032	0.041
TdelSat30-38	0.022	N.D.		
TdelSat31-52	0.022	R.P.		
TdelSat32-49	0.022	TinfSat16-49	0.015	0.004
TdelSat33-46	0.022	N.D.		
TdelSat34-27	0.019	R.P.		
TdelSat35-188	0.018	N.D.		
TdelSat36-128	0.018	TinfSat53-128	0.027	0.035
TdelSat37-38	0.017	R.P.		
TdelSat38-147	0.016	TinfSat13-147	0.025	0.014
TdelSat39-18	0.016	R.P.		
TdelSat40-72	0.016	R.P.		
TdelSat41-49	0.015	N.D.		
TdelSat42-128	0.015	N.D.		
TdelSat43-111	0.015	R.P.		
TdelSat44-34	0.014	R.P.		
TdelSat45-102	0.014	TinfSat18-102	0.014	0.006
TdelSat46-114	0.014	TinfSat67-114	0.008	0.007
TdelSat47-25	0.014	N.D.		
TdelSat48-118	0.014	TinfSat56-118	0.023	0.026
TdelSat49-49	0.014	R.P.		
TdelSat50-101	0.013	TinfSat96-101	<0.001	0.01
TdelSat51-46	0.013	TinfSat28-46	0.008	0.009
TdelSat52-88	0.013	TinfSat64-88	0.009	0.003
TdelSat53-48	0.013	R.P.		
TdelSat54-189	0.013	R.P.		
TdelSat55-51	0.012	N.D.		
TdelSat56-102	0.012	N.D.		
TdelSat57-271	0.012	R.P.		
TdelSat58-84	0.011	N.D.		
TdelSat59-63	0.011	TinfSat50-63	0.032	0.011
TdelSat60-99	0.011	TinfSat15-99	0.013	0.011
TdelSat61-315	0.010	TinfSat38-315	N.D.	0.031
TdelSat62-53	0.009	N.D.		
TdelSat63-51	0.009	N.D.		
TdelSat64-39	0.009	R.P.		
TdelSat65-71	0.008	N.D.		
TdelSat66-64	0.008	TinfSat22-64	0.012	0.002
TdelSat67-83	0.008	TinfSat85-83	0.001	0.002
TdelSat68-160	0.008	TinfSat66-161	0.008	0.011
TdelSat69-52	0.007	N.D.		
TdelSat70-86	0.007	TinfSat47-87	0.133	0.135
TdelSat71-102	0.007	R.P.		
TdelSat72-85	0.007	R.P.		
TdelSat73-372	0.007	TinfSat33-372	0.015	0.017
TdelSat74-137	0.007	N.D.		
TdelSat75-25	0.007	R.P.		
TdelSat76-96	0.007	TinfSat58-96	0.016	0.010
TdelSat77-102	0.007	R.P.		
TdelSat78-29	0.006	R.P.		
TdelSat79-26	0.006	R.P.		
TdelSat80-52	0.006	R.P.		
TdelSat81-185	0.006	R.P.		
TdelSat82-108	0.006	R.P.		
TdelSat83-66	0.006	R.P.		
TdelSat84-73	0.006	R.P.		
TdelSat85-49	0.006	N.D.		
TdelSat86-164	0.005	TinfSat57-164	0.017	0.006
TdelSat87-61	0.005	N.D.		
TdelSat88-87	0.005	TinfSat55-89	0.026	0.031
TdelSat89-86	0.005	N.D.		
TdelSat90-157	0.005	R.P.		
TdelSat91-23	0.005	R.P.		
TdelSat92-196	0.005	N.D.		
TdelSat93-57	0.004	R.P.		
TdelSat94-72	0.004	TinfSat79-72	0.003	0.001
TdelSat95-38	0.004	R.P.		
TdelSat96-58	0.004	R.P.		
TdelSat97-238	0.004	R.P.		
TdelSat98-104	0.004	R.P.		
TdelSat99-128	0.004	N.D.		
TdelSat100-99	0.004	N.D.		
TdelSat101-55	0.004	N.D.		
TdelSat102-39	0.004	R.P.		
TdelSat103-138	0.004	R.P.		
TdelSat104-78	0.004	N.D.		
TdelSat105-35	0.004	R.P.		
TdelSat106-44	0.004	TinfSat27-47	0.008	0.008
TdelSat107-57	0.004	R.P.		
TdelSat108-81	0.004	R.P.		
TdelSat109-142	0.004	R.P.		
TdelSat110-99	0.004	R.P.		
TdelSat111-7	0.004	TinfSat95-7	<0.001	0.005
TdelSat112-113	0.004	R.P.		
TdelSat113-88	0.004	N.D.		
TdelSat114-56	0.004	R.P.		
TdelSat115-15	0.004	N.D.		
TdelSat116-66	0.003	R.P.		
TdelSat117-46	0.003	N.D.		
TdelSat118-207	0.003	TinfSat82-207	0.002	0.002
TdelSat119-348	0.003	TinfSat78-348	0.004	0.004
TdelSat120-101	0.003	R.P.		
TdelSat121-51	0.003	R.P.		
TdelSat122-43	0.003	R.P.		
TdelSat123-149	0.003	R.P.		
TdelSat124-69	0.003	N.D.		
TdelSat125-35	0.003	R.P.		
TdelSat126-46	0.003	R.P.		
TdelSat127-49	0.003	R.P.		
TdelSat128-66	0.003	N.D.		
TdelSat129-60	0.002	R.P.		
TdelSat130-105	0.002	R.P.		
TdelSat131-66	0.002	R.P.		
TdelSat132-39	0.002	R.P.		
TdelSat133-28	0.002	R.P.		
TdelSat134-32	0.002	R.P.		
TdelSat135-105	0.002	R.P.		
TdelSat136-61	0.002	R.P.		
TdelSat137-183	0.002	N.D.		
TdelSat138-63	0.002	R.P.		
TdelSat139-152	0.002	N.D.		
TdelSat140-68	0.002	N.D.		
TdelSat141-369	0.002	R.P.		
TdelSat142-23	0.002	N.D.		
TdelSat143-52	0.002	N.D.		
TdelSat144-121	0.002	N.D.		
TdelSat145-36	0.002	R.P.		
TdelSat146-62	0.002	N.D.		
TdelSat147-275	0.002	R.P.		
TdelSat148-53	0.001	TinfSat26-53	0.011	0.002
TdelSat149-26	0.001	R.P.		
TdelSat150-56	0.001	N.D.		
TdelSat151-149	0.001	N.D.		
TdelSat152-50	0.001	R.P.		
TdelSat153-75	0.001	N.D.		
TdelSat154-45	0.001	R.P.		
TdelSat155-89	0.001	R.P.		
TdelSat156-78	0.001	R.P.		
TdelSat157-51	0.001	R.P.		
TdelSat158-134	0.001	R.P.		
TdelSat159-241	0.001	TinfSat63-237	0.010	0.002
TdelSat160-49	0.001	R.P.		
TOTAL	53.92			

R.P. = Detected in *T. infestans* by RepeatProfiler; N.D. = Not detected in *T. infestans* genome.

## Data Availability

All satDNA consensus sequences were submitted to the NCBI database (Acc. Numbers OQ82080-OQ82289).

## Supplementary Materials

The following are available online at https://www.mdpi.com/article/10.3390/genes14020371/s1. Table S1: data of the satDNA families found in *T. delpontei*; Table S2: data of the satDNA families found in *T. infestans*; Figure S1: alignments of the consensus sequences between different satDNA families of *T. delpontei*; Figure S2: alignments of the consensus sequences for the shared satDNA families between *T. delpontei* and *T. infestans*; Figure S3: satellite DNA landscapes for all satDNA families found in *Triatoma delpontei*; Figure S4: satellite DNA landscapes for all satDNA families found in Andean *Triatoma infestans*; Figure S5: satellite DNA landscapes for all satDNA families found in non-Andean *T. infestans*; Figure S6: RepeatProfiler results for the analyses of all satDNAs found in *T. delpontei* over three genomes: *T. delpontei*, Andean and non-Andean *T. infestans* lineages.
