# Supplementary material for: Making the Genome Huge: The Case of Triatoma delpontei, a Triatominae Species with More than 50% of Its Genome Full of Satellite DNA
Source: Genes (Basel). 2023 Jan 31;14(2):371. doi: 10.3390/genes14020371 (PMC9957312; doi:10.3390/genes14020371)
Supplement: Supplementary file 1 [file genes-14-00371-s001.zip › Figure S1 Consensus comparison.pdf]

Figure S1. Alignments of the consensus sequences between different satDNA families of *Triatoma delpontei*. The regions with the highest similarity are marked.

TdelSat05-1000 vs TdelSat23-112, TdelSat12-52, TdelSat51-46

```
TdelSat05-1000    ATCGTCGGAGCCATTTTTGAGAAAATCGCAAAAAAGTAAAAAAAAACAACTAGTAAAGTG        60

TdelSat05-1000    GTACTTCCGGTTGAGGAATTTTGACAGATAACGTACTGTCAGATCCCATGGTGATACCTA        120
TdelSat23-112                                              GAACCCACGGTGATATCTA        19
                                                           ** **** ******* ***

TdelSat05-1000    AACGGAATATCAAGTTTCAACTTTCTACGGTTTTTCGTTTTTGAGCTATGCTGTTCACAT        180
TdelSat23-112     AACAGAATGTCAAGTTTGAATTTTCTGCGATTTTTGTTTTTGTTGTGGTTGTGTTTGTTC        79
                  *** **** ******** ** ***** ** *****  ****   *   *  ****

TdelSat05-1000    ACATACATACACACACACACACACACACCATTTGCTAAAAACCACTTTTTTGGACTCAGG        240
TdelSat23-112     CGGTTGTGGAATTTTGACAGATAACTTAGTGTC                                   112
                     *      *     *** * *        *

TdelSat05-1000    GGACCTCAAAACGGATATTTCCGGTGAAAACTCGATATCGAAAATTTGACACGATTACAA        300

TdelSat05-1000    TACTTCCTCTTACTAGGAGTAAGAGAAAGTAAAAAAAAAAAATCTGGTGTGAAACACTCA        360
TdelSat12-52                                         ATATAAATCTGATGT---GTAATCA        22
                                                     * * ******* ***     * ***

TdelSat05-1000    CACAACTTTCTCTTACTCCAGTTCTCAAAATTATAATTGACAAATTTTATAGTTTGTGTA        420
TdelSat12-52      CACGACTTTCTCTTAGTGCTGTAGGAGGTG                                      52
                  *** *********** * * **

TdelSat05-1000    CCAAATTTAATGAAATTCGTCGAAAACTGAAAAAAACTGTTCAGTAAAAGCGCACTTCCG        480
TdelSat05-1000    GTTTACTAATTTTCTTGAAACTCGGAATTTTGGCCATCTATTTATTTCTAATTAATTTTG        540
TdelSat05-1000    TTTGGATGATAGAATTGTATAATCAACTATGATAGCACTTTAGAGAAAAACATAACCCCA        600
TdelSat05-1000    CCCCTCCCCTAAAAGTGCCCTTAAATGAATTTTCCCAGAGAAATGTTTTAAATAAAAGTT        660
TdelSat05-1000    GTAGATCTTTGTATGTGTAGTTTATATACGAAGTTTCAAGAAAATCGTCGGAGCCATTTT        720
TdelSat05-1000    TGAGAAAATCGCGAAAAAGTGAAAAAAATCATTTAGTAAAGTGGTAAAGTAAACGCACTT        780
TdelSat05-1000    CCGGTTATCCGATTTTTTTCAAACTCGGAATTTCAACAACATATTTAATTCTAATTAACA        840
TdelSat05-1000    TTATTTAGATGACAGAATTGTCTAATAAACTGAAATAGCACTTTAGATGAAAACCTAACC        900

TdelSat05-1000    CCACCCCTCCCCCAAAAGTGCCCTTAAATGAATTTTCCCGGAGAAATGTTTCAAATAAAA        960
TdelSat51-46                                          CCGATACAGAAAGTGTAAATAAA-        31
                                                      **   * * *   *  *******

TdelSat05-1000    GTTGTAGCTCTTTGTATGTGTAGTTGATATACCAAGTTTAAAGAAA          1006
TdelSat51-46      GTTGTCGCTCTTTGTATGTACAG                                 46
                  ***** *************  **
```

TdelSat05-1000 vs TdelSat70-86, TdelSat29-137

```
TdelSat05-1000    ATCGTCGGAGCCATTTTTGAGAAAATCGCAAAAAAGTAAAAAAAAACAACTAGTAAAGTG        60

TdelSat05-1000    GTACTTCCGGTTGAGGAATTTTGACAGATAACGTACTGTCAGATCCCATGGTGATACCTA        120
TdelSat70-86              GTACCACTGCTTCTAACACGAACGGAAGGTTAGTGGCAGTTTGTGATACCTA        52
                          *    *    ** * ***   *  * *   *     *   * **********

TdelSat05-1000    AACGGAATATCAAGTTTCAACTTTCTACGGTTTTTCGTTTTTGAGCTATGCTGTTCACAT        180
TdelSat70-86      AACGGAATGTCAAACTTGGTATTCCGTTTATGTA                                  86
                  ******** ****  **    ** *     * *

TdelSat05-1000    ACATACATACACACACACACACACACACCATTTGCTAAAAACCACTTTTTTGGACTCAGG        240

TdelSat05-1000    GGACCTCAAAACGGATAT--TTCCGGTGAAAACTCGATATCGAAAATTTGACACGATTAC        298
TdelSat29-137         CTACAATGAGATATATATAAAGTGACGAATTTACTT--TAAACTT---CCAATTTT        51
                      **  **   *****   *   ****  * *  *  *   *** **    * ***

TdelSat05-1000    AATACTTCCTCTTACTAGGAGTAAGAGAAAGTAAAAAAAAAAAAATCTGGTGTGAAACACT        358
TdelSat29-137     ACTCATTTGTCCTAACATATAGTTTGGTGATGTTAGGAGGGGGAGGTGGTGGGGGACACT        111
                  * *  **  ** **  *         *  *    *  *     *  ***** *  *****

TdelSat05-1000    CACACAACTTTCTCTTACTCCAGTTCTCAAAATTATAATTGACAAATTTTATAGTTTGTG        418
TdelSat29-137     CACACGACTTTCTCTTACTCCTGTTA                                          137
                  ***** *************** ***

TdelSat05-1000    CGGTTTACTAATTTTCTTGAAACTCGGAATTTTGGCCATCTATTTATTTCTAATTAATTT        538
TdelSat05-1000    TGTTTGGATGATAGAATTGTATAATCAACTATGATAGCACTTTAGAGAAAAACATAACCC        598
TdelSat05-1000    CACCCCTCCCCTAAAAGTGCCCTTAAATGAATTTTCCCAGAGAAATGTTTTAAATAAAAG        658
TdelSat05-1000    TTGTAGATCTTTGTATGTGTAGTTTATATACGAAGTTTCAAGAAAATCGTCGGAGCCATT        718
TdelSat05-1000    TTTGAGAAAATCGCGAAAAAGTGAAAAAAATCATTTAGTAAAGTGGTAAAGTAAACGCAC        778
TdelSat05-1000    TTCCGGTTATCCGATTTTTTTCAAACTCGGAATTTCAACAACATATTTAATTCTAATTAA        838
TdelSat05-1000    CATTATTTAGATGACAGAATTGTCTAATAAACTGAAATAGCACTTTAGATGAAAACCTAA        898
```

```
TdelSat05-1000      CCCCACCCCTCCCCCAAAAGTGCCCTTAAATGAATTTTCCCGGAGAAATGTTTCAAATAA       958
TdelSat05-1000      AAGTTGTAGCTCTTTGTATGTGTAGTTGATATACCAAGTTTAAAGAAA        1006
```

TdelSat05-1000 vs TdelSat43-111

```
TdelSat05-1000      ATCGTCGGAGCCATTTTTGAGAAAATCGCAAAAAAGTAAAAAAAAACAACTAGTAAAGTG       60
TdelSat05-1000      GTACTTCCGGTTGAGGAATTTTGACAGATAACGTACTGTCAGATCCCATGGTGATACCTA       120
TdelSat05-1000      AACGGAATATCAAGTTTCAACTTTCTACGGTTTTTCGTTTTTGAGCTATGCTGTTCACAT       180
TdelSat05-1000      ACATACATACACACACACACACACACACCATTTGCTAAAAACCACTTTTTTGGACTCAGG       240
TdelSat05-1000      GGACCTCAAAACGGATATTTCCGGTGAAAACTCGATATCGAAAATTTGACACGATTACAA       300

TdelSat05-1000      TACTTCCTCTTACTAGGAGTAAGAGAAAGTAAAAAAAAAAAATCTGGTGTGAAACACTCA       360
TdelSat43-111                                        AAAAAAAAATCTGGTGTGAAACACTAA       27
                                                     ************************* *

TdelSat05-1000      CACAACTTTCTCTTACTCCAGTTCTCAAAATTATAATTGACAAATTTTATAGTTTGTGTA       420
TdelSat43-111       CACCTT--TCTCTTACAGCTAGCATAGGAAGTACTGTGATCGTGTC--------------       71
                    ***     ********  *     *   ** **   *   *   *

TdelSat05-1000      CCAAATTTAATGAAATTCGTCGAAAACTGAAAAAAACTGTTCAGTAAAAGCGCACTTCCG       480
TdelSat43-111       --ATTTTTTTTAAAATTGAGTTTACACCGGAAAGACATATTT                         111
                      *  ***  * *****      * ** * *** *  * **

TdelSat05-1000      GTTTACTAATTTTCTTGAAACTCGGAATTTTGGCCATCTATTTATTTCTAATTAATTTTG       540
TdelSat05-1000      TTTGGATGATAGAATTGTATAATCAACTATGATAGCACTTTAGAGAAAAACATAACCCCA       600
TdelSat05-1000      CCCCTCCCCTAAAAGTGCCCTTAAATGAATTTTCCCAGAGAAATGTTTTAAATAAAAGTT       660
TdelSat05-1000      GTAGATCTTTGTATGTGTAGTTTATATACGAAGTTTCAAGAAAATCGTCGGAGCCATTTT       720
TdelSat05-1000      TGAGAAAATCGCGAAAAAGTGAAAAAAATCATTTAGTAAAGTGGTAAAGTAAACGCACTT       780
TdelSat05-1000      CCGGTTATCCGATTTTTTTCAAACTCGGAATTTCAACAACATATTTAATTCTAATTAACA       840
TdelSat05-1000      TTATTTAGATGACAGAATTGTCTAATAAACTGAAATAGCACTTTAGATGAAAACCTAACC       900
TdelSat05-1000      CCACCCCTCCCCCAAAAGTGCCCTTAAATGAATTTTCCCGGAGAAATGTTTCAAATAAAA       960
TdelSat05-1000      GTTGTAGCTCTTTGTATGTGTAGTTGATATACCAAGTTTAAAGAAA          1006
```

TdelSat05-1000 vs TdelSat48-118

```
TdelSat05-1000      ATCGTCGGAGCCATTTTTGAGAAAATCGCAAAAAAGTAAAAAAAAACAACTAGTAAAGTG       60
TdelSat05-1000      GTACTTCCGGTTGAGGAATTTTGACAGATAACGTACTGTCAGATCCCATGGTGATACCTA       120
TdelSat05-1000      AACGGAATATCAAGTTTCAACTTTCTACGGTTTTTCGTTTTTGAGCTATGCTGTTCACAT       180
TdelSat05-1000      ACATACATACACACACACACACACACACCATTTGCTAAAAACCACTTTTTTGGACTCAGG       240

TdelSat05-1000      GGACCTCAAAACGGATATTTCCGGTGAAAACTCGATATCGAAAATTTGACACGATTACAA       300
TdelSat48-118                                                     CGCTTGTACAGCAG       14
                                                                         *   **

TdelSat05-1000      TACTTCCTCTTACTAGGAGTAAGAGAAAGTAAAAAAAAAAAATCTGGTGTGAAACACTCA       360
TdelSat48-118       TAAATGGAACCATTAACTTGCTACCAGGTGTTTCAAAGTAAAACTGGTGTGAAATATTCA       74
                    **  *      * **          *        ***  *** *********** * ***

TdelSat05-1000      CACAACTTTCTCTTACTCCAGTTCTCAAAATTATAATTGACAAATTTTATAGTTTGTGTA       420
TdelSat48-118       CACGACTTTCTCTTACTCCAGTTAATACAATT-TAACTGTTCAAT                      118
                    *** *******************   * **** *** **   ***

TdelSat05-1000      CCAAATTTAATGAAATTCGTCGAAAACTGAAAAAAACTGTTCAGTAAAAGCGCACTTCCG       480
TdelSat05-1000      GTTTACTAATTTTCTTGAAACTCGGAATTTTGGCCATCTATTTATTTCTAATTAATTTTG       540
TdelSat05-1000      TTTGGATGATAGAATTGTATAATCAACTATGATAGCACTTTAGAGAAAAACATAACCCCA       600
TdelSat05-1000      CCCCTCCCCTAAAAGTGCCCTTAAATGAATTTTCCCAGAGAAATGTTTTAAATAAAAGTT       660
TdelSat05-1000      GTAGATCTTTGTATGTGTAGTTTATATACGAAGTTTCAAGAAAATCGTCGGAGCCATTTT       720
TdelSat05-1000      TGAGAAAATCGCGAAAAAGTGAAAAAAATCATTTAGTAAAGTGGTAAAGTAAACGCACTT       780
TdelSat05-1000      CCGGTTATCCGATTTTTTTCAAACTCGGAATTTCAACAACATATTTAATTCTAATTAACA       840
TdelSat05-1000      TTATTTAGATGACAGAATTGTCTAATAAACTGAAATAGCACTTTAGATGAAAACCTAACC       900
TdelSat05-1000      CCACCCCTCCCCCAAAAGTGCCCTTAAATGAATTTTCCCGGAGAAATGTTTCAAATAAAA       960
TdelSat05-1000      GTTGTAGCTCTTTGTATGTGTAGTTGATATACCAAGTTTAAAGAAA          1006
```

TdelSat05-1000 vs TdelSat57-271

```
TdelSat05-1000      ATCGTCGGAGCCATTTTTGAGAAAATCGCAAAAAAGTAAAAAAAAACAACTAGTAAAGTG       60
TdelSat05-1000      GTACTTCCGGTTGAGGAATTTTGACAGATAACGTACTGTCAGATCCCATGGTGATACCTA       120
TdelSat05-1000      AACGGAATATCAAGTTTCAACTTTCTACGGTTTTTCGTTTTTGAGCTATGCTGTTCACAT       180
TdelSat05-1000      ACATACATACACACACACACACACACACCATTTGCTAAAAACCACTTTTTTGGACTCAGG       240

TdelSat05-1000      GGACCTCAAAACGGATATTTCCGGTGAAAACTCGATATCGAAAATTTGACACGATTACAA       300
TdelSat57-271       ---------------------------------------------ACAAAAAAAATAAAA       15
                                                                    * *  * ** **

TdelSat05-1000      TACTTCCTCTTACTAGGAGTAAGAGAAAGTAAAAAAAAAAAATCTGGTGTGAAACACTCA       360
TdelSat57-271       TACTTCCTGTTAGTAGGAGTAAGAGAAAGTAAAAAAGTAGGATAGTAGAAGTGATGACAA       75
                    ******** *** ***********************  *  **       *  *     *

TdelSat05-1000      CACA------ACTTTCTCTTACTCCAGTTCTCAAAATTATAATTGACAAATTTTATAGTT       414
TdelSat57-271       GTCGGCCAGAAGAGTGTGCCAATAACATTCAACCATCA-------AGCCCATATAAAATT       128
                      *       *   * *   * *    ***    *          *     * ** * **
```

```
TdelSat05-1000      TGTGTACCAAATTTAATGAAATTCGTCGAAAACTGAAAAAAACTGTTCAGTAAAAGCGCA       474
TdelSat57-271       TGAATTTCA---------------------------------------------------       137
                    **  *  **

TdelSat05-1000      CTTCCGGTTTACTAATTTTCTTGAAACTCGGAATTTTGGCCATCTATTTATTTCTAATTA       534
TdelSat05-1000      ATTTTGTTTGGATGATAGAATTGTATAATCAACTATGATAGCACTTTAGAGAAAAACATA       594

TdelSat05-1000      ACCCCACCCCTCCCCTAAAAGTGCCCTTAAATGAATTTTCC-CAGAGAAATGTTTTAAAT       653
TdelSat57-271       ACGCTACAGATTCCGCAAAACTACATTAAAATCCATGTTTTTCAAAAAAAATTTACAAAT       197
                    ** * **   * **  **** * *  * ****  ** **   ** * ***  **  ****

TdelSat05-1000      AAAA-GTTGT-AGATCTTTGTATGTGTAGTTTATATACGAAGTTTCAAGAAAATCGTCGG       711
TdelSat57-271       AAAAAGTGAAAGGGATTTTATATGTGTAGATAGTATACAAAAATTTGAAGCC--------       249
                    **** **     *   *** ********* *  ***** **  **  *

TdelSat05-1000      AGCCATTTTTGAGAAAATCGCGAAAAAGTGAAAAAAATCATTTAGTAAAGTGGTAAAGTA       771
TdelSat57-271       ---ATTGTTTGAGGAAATCGCAAAA                                          271
                         * ****** ******* ***

TdelSat05-1000      AACGCACTTCCGGTTATCCGATTTTTTTCAAACTCGGAATTTCAACAACATATTTAATTC       831
TdelSat05-1000      TAATTAACATTATTTAGATGACAGAATTGTCTAATAAACTGAAATAGCACTTTAGATGAA       891
TdelSat05-1000      AACCTAACCCCACCCCTCCCCCAAAAGTGCCCTTAAATGAATTTTCCCGGAGAAATGTTT       951
TdelSat05-1000      CAAATAAAAGTTGTAGCTCTTTGTATGTGTAGTTGATATACCAAGTTTAAAGAAA  1006
```

TdelSat05-1000 vs TdelSat74-137

```
TdelSat05-1000      ATCGTCGGAGCCATTTTTGAGAAAATCGCAAAAAAGTAAAAAAAAACAACTAGTAAAGTG       60
TdelSat05-1000      GTACTTCCGGTTGAGGAATTTTGACAGATAACGTACTGTCAGATCCCATGGTGATACCTA       120
TdelSat05-1000      AACGGAATATCAAGTTTCAACTTTCTACGGTTTTTCGTTTTTGAGCTATGCTGTTCACAT       180

TdelSat05-1000      ACATACATACACACACACACACACACACCATTTGCTAAAAACCACTTTTTTGG-ACTCAG       239
TdelSat74-137                                                  TCAATATTATGATGGTCGC       19
                                                              ** * ** **    **

TdelSat05-1000      GGGACCTCAAAACGGATATTTCCGGTGAAAACTCGATATCGAAAATTTGACACGATTACA       299
TdelSat74-137       GGCT-------ACAGAT-TCTGTAAAAATTCCATGATTGAAGAAAATTTGTCCCGTCGCA       71
                    **         ** *** * *      *   *  ***     *** **    *  *  **

TdelSat05-1000      ATACTTCCTCTTACTAGGAGTAAGAGAAAGTAAAAAAAAAAAATCTGGTGTGAAACACTC       359
TdelSat74-137       AGTCTTCCTCTTACGAGGAGTAAAAGGAAATAAAAAACTAATAATGTACAATTATGGCCC       131
                    *  *********** ******** ** ** *******  ** *          *   * *

TdelSat05-1000      ACACAACTTTCTCTTACTCCAGTTCTCAAAATTATAATTGACAAATTTTATAGTTTGTGT       419
TdelSat74-137       CCCCCA                                                             137
                     * * *

TdelSat05-1000      ACCAAATTTAATGAAATTCGTCGAAAACTGAAAAAAACTGTTCAGTAAAAGCGCACTTCC       479
TdelSat05-1000      GGTTTACTAATTTTCTTGAAACTCGGAATTTTGGCCATCTATTTATTTCTAATTAATTTT       539
TdelSat05-1000      GTTTGGATGATAGAATTGTATAATCAACTATGATAGCACTTTAGAGAAAAACATAACCCC       599
TdelSat05-1000      ACCCCTCCCCTAAAAGTGCCCTTAAATGAATTTTCCCAGAGAAATGTTTTAAATAAAAGT       659
TdelSat05-1000      TGTAGATCTTTGTATGTGTAGTTTATATACGAAGTTTCAAGAAAATCGTCGGAGCCATTT       719
TdelSat05-1000      TTGAGAAAATCGCGAAAAAGTGAAAAAAATCATTTAGTAAAGTGGTAAAGTAAACGCACT       779
TdelSat05-1000      TCCGGTTATCCGATTTTTTTCAAACTCGGAATTTCAACAACATATTTAATTCTAATTAAC       839
TdelSat05-1000      ATTATTTAGATGACAGAATTGTCTAATAAACTGAAATAGCACTTTAGATGAAAACCTAAC       899
TdelSat05-1000      CCCACCCCTCCCCCAAAAGTGCCCTTAAATGAATTTTCCCGGAGAAATGTTTCAAATAAA       959
TdelSat05-1000      AGTTGTAGCTCTTTGTATGTGTAGTTGATATACCAAGTTTAAAGAAA          1006
```

TdelSat05-1000 vs TdelSat81-185

```
TdelSat05-1000      ATCGTCGGAGCCATTTTTGAGAAAATCGCAAAAAAGTAAAAAAAAACAACTAGTAAAGTG       60
TdelSat05-1000      GTACTTCCGGTTGAGGAATTTTGACAGATAACGTACTGTCAGATCCCATGGTGATACCTA       120

TdelSat05-1000      AACGGAATATCAAGTTTCAACTTTCTACGGTTTTTCGTTTTTGAGCTATGCTGTTCACAT       180
TdelSat81-185                                                   CACACACCACACACAC       16
                                                                   *  *    ****

TdelSat05-1000      ACATACATACACACACA-CACACACACACCATTTGCTAAAAACCACTTTTTTGGACTCAG       239
TdelSat81-185       ACATATATATATAGAAAGAGAAAGAAAACAATTTTATAAAAATCACATTTTTGGACTCAG       76
                    ***** *** * * * *    *   * ** ****  ****** *** *************

TdelSat05-1000      GGGACCTCAAAACGGATATTTCCGGTGAAAACTCGATATCGAAAATTTGACACGATTACA       299
TdelSat81-185       GGGATCTCAAAACATACACTTGCAATTTTATTTAATTGAAACTGCGTGTCGTATTCCCTA      137
                    **** ********  * * ** *  *   *  *   *                   *

TdelSat05-1000      ATACTTCCTCTTACTAGGAGTAAGAGAAAGTAAAAAAAAAAAATCTGGTGTGAAACACTC       359
TdelSat81-185       GAGATATCCATAATATGAGAGTTATATTGTTTTCCCTCTCACACGCATA                  185
                        *  *  * *   *             *

TdelSat05-1000      ACACAACTTTCTCTTACTCCAGTTCTCAAAATTATAATTGACAAATTTTATAGTTTGTGT       419
TdelSat05-1000      ACCAAATTTAATGAAATTCGTCGAAAACTGAAAAAAACTGTTCAGTAAAAGCGCACTTCC       479
TdelSat05-1000      GGTTTACTAATTTTCTTGAAACTCGGAATTTTGGCCATCTATTTATTTCTAATTAATTTT       539
TdelSat05-1000      GTTTGGATGATAGAATTGTATAATCAACTATGATAGCACTTTAGAGAAAAACATAACCCC       599
TdelSat05-1000      ACCCCTCCCCTAAAAGTGCCCTTAAATGAATTTTCCCAGAGAAATGTTTTAAATAAAAGT       659
```

```
TdelSat05-1000      TGTAGATCTTTGTATGTGTAGTTTATATACGAAGTTTCAAGAAAATCGTCGGAGCCATTT      719
TdelSat05-1000      TTGAGAAAATCGCGAAAAAGTGAAAAAAATCATTTAGTAAAGTGGTAAAGTAAACGCACT      779
TdelSat05-1000      TCCGGTTATCCGATTTTTTTCAAACTCGGAATTTCAACAACATATTTAATTCTAATTAAC      839
TdelSat05-1000      ATTATTTAGATGACAGAATTGTCTAATAAACTGAAATAGCACTTTAGATGAAAACCTAAC      899
TdelSat05-1000      CCCACCCCTCCCCCAAAAGTGCCCTTAAATGAATTTTCCCGGAGAAATGTTTCAAATAAA      959
TdelSat05-1000      AGTTGTAGCTCTTTGTATGTGTAGTTGATATACCAAGTTTAAAGAAA          1006
```

TdelSat05-1000 vs TdelSat-138-63

```
TdelSat05-1000      ATCGTCGGAGCCATTTTTGAGAAAATCGCAAAAAAGTAAAAAAAAACAACTAGTAAAGTG      60
TdelSat05-1000      GTACTTCCGGTTGAGGAATTTTGACAGATAACGTACTGTCAGATCCCATGGTGATACCTA      120
TdelSat05-1000      AACGGAATATCAAGTTTCAACTTTCTACGGTTTTTCGTTTTTGAGCTATGCTGTTCACAT      180
TdelSat05-1000      ACATACATACACACACACACACACACCATTTGCTAAAAACCACTTTTTTGGACTCAGG      240
TdelSat05-1000      GGACCTCAAAACGGATATTTCCGGTGAAAACTCGATATCGAAAATTTGACACGATTACAA      300

TdelSat05-1000      TACTTCCTCTTACTAGGAGTAAGAGAAAGTAAAAAAAAAAAATCTGGTGTGAAACACTCA      360
TdelSat-138-63      ---TTGCCACTAACAGCAATAAGAGAAAGTAAAAAAGAAGAAAATTTGCCATTTTACATA
                       ** *   **  ** * ***************** ** **  *          **  *

TdelSat05-1000      CACAACTTTCTCTTACTCCAGTTCTCAAAATTATAATTGACAAATTTTATAGTTTGTGTA      420
TdelSat-138-63      TAATTA                                                            63
                     *

TdelSat05-1000      CCAAATTTAATGAAATTCGTCGAAAACTGAAAAAAACTGTTCAGTAAAAGCGCACTTCCG      480
TdelSat05-1000      GTTTACTAATTTTCTTGAAACTCGGAATTTTGGCCATCTATTTATTTCTAATTAATTTTG      540
TdelSat05-1000      TTTGGATGATAGAATTGTATAATCAACTATGATAGCACTTTAGAGAAAAACATAACCCCA      600
TdelSat05-1000      CCCCTCCCCTAAAAGTGCCCTTAAATGAATTTTCCCAGAGAAATGTTTTAAATAAAAGTT      660
TdelSat05-1000      GTAGATCTTTGTATGTGTAGTTTATATACGAAGTTTCAAGAAAATCGTCGGAGCCATTTT      720
TdelSat05-1000      TGAGAAAATCGCGAAAAAGTGAAAAAAATCATTTAGTAAAGTGGTAAAGTAAACGCACTT      780
TdelSat05-1000      CCGGTTATCCGATTTTTTTCAAACTCGGAATTTCAACAACATATTTAATTCTAATTAACA      840
TdelSat05-1000      TTATTTAGATGACAGAATTGTCTAATAAACTGAAATAGCACTTTAGATGAAAACCTAACC      900
TdelSat05-1000      CCACCCCTCCCCCAAAAGTGCCCTTAAATGAATTTTCCCGGAGAAATGTTTCAAATAAAA      960
TdelSat05-1000      GTTGTAGCTCTTTGTATGTGTAGTTGATATACCAAGTTTAAAGAAA           1006
```

TdelSat12-52 vs TdelSat48-118

```
TdelSat48-118       ATACAATTTAACTGTTCAATCGCTTGTACAGCAGTAAATGGAACCATTAACTTGCTACCA        60


TdelSat12-52                 ATATAAA---TCTGATGTGTAATCACACGACTTTCTCTTAGTGCTGTAGGA        48
TdelSat48-118       GGTGTTTCAAAGTAAAACTGGTGTGAAATATTCACACGACTTTCTCTTACTCCAGTTA      118
                             *  ****     **     ** ****************** * * **

TdelSat12-52        GGTG          52
```

TdelSat12-52 vs TdelSat29-137

```
TdelSat29-137       CTACAATGAGATATATATAAAGTGACGAATTTACTTTAAACTTCCAATTTTACTCATTTG      60

TdelSat12-52                                       ATATAAATCTGATGTGTAATCACACGACT      31
TdelSat29-137       TCCTAACATATAGTTTGGTGATGTTAGGAGGGGGAGGTGGGGGGACACTCACACGACT      120
                                                            **  *   * **********


TdelSat12-52        TTCTCTTAGTGCTGTAGGAGGTG 52
TdelSat29-137       TTCTCTTACTCCTGTTA      137
                    ******** * ****
```

TdelSat21-33 vs TdelSat57-271

```
TdelSat21-33        TCTACACACATAAATACC-TATCAC-----------------------------------        23
TdelSat57-271       TCTACACATATAAAATCCCTTTCACTTTTTATTTGTAAATTTTTTTTGAAAAACATGGAT        60
                    ******** *****  ** * ****
TdelSat21-33        ------------------------------------------------------------        23
TdelSat57-271       TTTAATGTAGTTTTGCGGAATCTGTAGCGTTGAAATTCAAATTTTATATGGGCTTGATGG        120

TdelSat21-33        ------------------------------------------------------------        23
TdelSat57-271       TTGAATGTTATTGGCACACTCTTCTGGCCGACTTGTCATCACTTCTACTATCCTACTTTT        180

TdelSat21-33        ------------------------------------------------------------        23
TdelSat57-271       TTACTTTCTCTTACTCCTACTAACAGGAAGTATTTTATTTTTTTTGTTTTTGCGATTTCC        240

TdelSat21-33        ----------------------TGTATACTA  33
TdelSat57-271       TCAAACAATGGCTTCAAATTTTTGTATACTA  271
                                          *********
```

TdelSat21-33 vs TdelSat64-39

```
TdelSat21-33      ACTATCTACACACATAAATA------CCTATCACTGTAT   33
TdelSat64-39      ACTAACTACACACATAATTATTTAAATTATATATCGTAT   39
                  **** ************ **            *  ****
```

TdelSat24-147 vs TdelSat122-43

```
TdelSat24-147      CCAAGAAAGTTCATTTTATGGCGTTTTGGGTGGGGGGTAAGGGGCAAGCTTTTCCCAAAA       60


TdelSat24-147      TAGTGGTTTTTATTGTTTAGATTCTCCCACTGATGTAAAATTGAAGACAACTAAACATCT       120
TdelSat122-43                             ACTTAAGTATTGTAAAATTGAAGACAAGCAAACGAAA       37
                                               * *  *****************  ****

TdelSat24-147      AGTGAGAGAATTAGACTTTTGAAAGTT     147
TdelSat122-43      TTGAAG                          43
                       **
```

TdelSat29-137 vs TdelSat48-118

```
TdelSat29-137      CTACAATGAGATATATATAAAGTGACGAATTTACTTTAAACTTCCAATTTTACTCATTTG       60
TdelSat48-118      ----------ATACAATTTAACTGTTCAATCGCTTG---------TACAGCAGTAAATGG       41
                             *** *  * ** **   ***    *           *    * * * * *

TdelSat29-137      TCCTAACATATAGTTTGGTGATGTTAGGAGGGGGAGGTGGTGGGGGACACTCACACGACT       120
TdelSat48-118      AACCATTAACTTGCTACCAGGTGTTTCAAAGTAAAACTGGTGTGAAATATTCACACGACT       101
                     * *  *  * * *    * ****   * *   *  ***** *  * * **********

TdelSat29-137      TTCTCTTACTCCTGTTA       137
TdelSat48-118      TTCTCTTACTCCAGTTA       118
                   ************ ****
```

TdelSat36-128 vs TdelSat54-189

```
TdelSat36-128         TCTCAATTACTCATATGTCCTAATATAAATATACAGGTGGTGGGAGACAAG-----T       52
TdelSat54-189      TTTCAAATTTTGCTCATATATCCCAACATAAATATCCAGGTGGTGGGGGGAGGGACGAGT       60
                            ** ******* *** ** ******** *********** *    *     *

TdelSat36-128      GCCAGCCTAGCAACACAGACAGGTACAGAATGATTACATTGTAGACAGCCAATAATATTG       112
TdelSat54-189      GCCAATGTACCACCAGGGCAGGTAGATAACGGATTGCACGGTAGAGA-GCGTCAATAATG       119
                   ****   ** ** **  *   *      *  **** **  ***** *  *   **** **

TdelSat36-128      AACAAACACATTTGAA                                                   128
TdelSat54-189      AACAAACACATTTGAATTTTTAACGTCTCTTGATTCCTCAGAGAACCACCTAAAAACAAT       179
                   ****************

TdelSat54-189      TTTTTTTAAA     189
```

TdelSat36-128 vs TdelSat72-85

```
TdelSat36-128      TCTCAATTACTCATATGTCCTAATATAAATATACAGGTGGTGGGAGACAAGTGCCAGCCT       60
TdelSat72-85                          ATAACATAAATATGGAGGTGGTGGGAGATAGGTAAATACCT       41
                                       *** ********  ************* * **     ***

TdelSat36-128      AGCAACACAGACAGGTACAG---AATG-ATTACATTGTAGACAGCCAATAATATTGAACA       116
TdelSat72-85       CTCTACTCAGCTGGCAGGTAGACAAGTGTTTATGTATTTGTCCT                       85
                     * ** ***   *         **    ***  *  * * *

TdelSat36-128      AACACATTTGAA   128
```

TdelSat48-118 vs TdelSat88-87 (reverse)

```
TdelSat48-118      ATACAATTTAACTGTTCAATCGCTTGTACAGCAGTAAATGGAACCATTAACTTGCTACCA        60
TdelSat88-87 (rev) ATACAATTACAATTTAC-------------------------------AAATTTTCTTTA        29
                   ********  * * * *                               ** **      *

TdelSat48-118      GGTGTTTCAAAGTAAAACTGGTGTGAAATATTCACACGACTTTCTCTTACTCCAGTTA          118
TdelSat88-87 (rev) TATTGTAATGTTACTCAATGGTGTGAAACATTCAGACGACTTGGTCTCATTGCAGTTA          87
                     *  *          * ********** ***** *******  *** * * ******
```

TdelSat54-189 vs TdelSat72-85

```
TdelSat54-189      TTTCAAATTTTGCTCATATATCCCAACATAAATATCCAGGTGGTGGGGGGAGGGACGAGT        60
TdelSat72-85                             ATAACATAAATATGGAGGTGGTGGGAGATAGGTA-AAT        37
                                           ***********  ********** *   **   * *

TdelSat54-189      GCCAATGTACCACCAGGGCAGGTAGATAACGGATTGCACGGTAGAGAGCGTCAATAATGA        120
TdelSat72-85       ACCTCTCTACTCAGCTGGCAGGTAGACAAGTGTTTATGTATTTGTCCT                    85
                    **  * ***      ********** **  * **      * *

TdelSat54-189      ACAAACACATTTGAATTTTTAACGTCTCTTGATTCCTCAGAGAACCACCTAAAAACAATT        180

TdelSat54-189      TTTTTTAAA     189
```

TdelSat68-160 vs TdelSat73-372

The sequence of TdelSat73-372 was separated into two regions: the first one between positions 1 to 237 (TdelSat73-372-1) and the second one between positions 238 and 372 (TdelSat73-372-2). Ast

```
                  ************************************* **********************
TdelSat73-372-1   TAGTGCTGCGTTTGTGCCGGCGATTCACCAAATTTCAACGTTTGTGCCGTAAAATTGACA       60
TdelSat68-160     TAGTGCTGCGTTTGTGCCGGCGATTCACCAAATTTCATCGTTTGTGCCGTAAAATTGACA       60
TdelSat73-372-2   AAGTGATTCATTTGTGCCAGCGACCCACCAAATTTCAGCGTTTGTGCTGCAAAATTGACA       297
                   **** * * ******** ****  ************ ********* * **********

                  ******** * ******  **     ** *      *
TdelSat73-372-1   AAGATATTGACGAAAAACTGTTCGTCGTACTTGAAAACCAAAGGCCATGGCTGCGAGTAA       120
TdelSat68-160     AAGATATTAAAGAAAAAAAGTGGCCAGTCCAACTTGA-----------------------       97
TdelSat73-372-2   AAGTTATTGAGG------------------------------------------------       309
                  *** **** * *

                                                                            **
TdelSat73-372-1   CATTTGAATGTGCGCGTGCTCCACTGACCGCCAACGGCCCTTATCAGGGCCAGCAACTAA       180
TdelSat68-160     ------------------------------------------------------CGTCAA       103
TdelSat73-372-2   ------------------------------------------------------CGTCAA       315
                                                                        ******

                    *  *******  * ******* ***   ** *  ****** * **  * * ** *
TdelSat73-372-1   AATGTACTGGCCCTCGATTTCTAATCAAAATTCTGGAATTTTTTCTTCGCTATTCTT          237
TdelSat68-160     GTTAGACTGGCCACCAATTTCTATTCACTTTTTTAAAATTTTCTTTTTACCAATCGT          160
TdelSat73-372-2   GTTAGACTGGCCACCAATTTCTATTCACTTTTTTAAACTTTTCTCTTTGCCAATCGT          372
                  ************************************* ****** *** ********
```
