# Supplementary material for: Making the Genome Huge: The Case of Triatoma delpontei, a Triatominae Species with More than 50% of Its Genome Full of Satellite DNA
Source: Genes (Basel). 2023 Jan 31;14(2):371. doi: 10.3390/genes14020371 (PMC9957312; doi:10.3390/genes14020371)
Supplement: Supplementary file 1 [file genes-14-00371-s001.zip › Figure S2 Consensus alignments.pdf]

Figure S2. Alignments of the consensus sequences for the shared satDNA families between *Triatoma delpontei* and *Triatoma infestans*.

```
TdelSat01-79      CCCTAAAATCGGCGTTTCAAACGTAAGAGTGAGTTTTCGAGCAACACTCACTTCAGTT 60
TinfSat02-79      CCCTAAAATCGGCGTTTCAAACGTAAGAGTGAGTTTTCGAGCAACACTCACTTCAGTT 60
                  **********************************************************

TdelSat01-79      GGTTGTAAGGTTCAAGAAAAT     79
TinfSat02-79      GGTTGTAAGGTTCAAGAAAAT     79
                  *********************
```

```
TdelSat02-8       GATAGTTA       8
TinfSat03-4 (2X)  GATAGATA       8
                  ***** **
```

```
TdelSat03-10      RCATACTCGK     10
TinfSat07-10      RCATACTCGK     10
TinfSat36-10-rev  ACATACTCGK     10
                   *********
```

```
TdelSat04-53      AACGGTTTTGGTTATACTATTTTTCCAAACCCGCAATACACGTTTGCCCCTTC      53
TinfSat10-53      AACGGTTTTGGTTATACTATTTTTCCAAACCCGCAATACACGTTTGCCCCTTC      53
                  *****************************************************
```

```
TdelSat05-1000     ATCGTCGGAGCCATTTTTGAGAAAATCGCAAAAAAGTAAAAAAAAACAACTAGTAAAGTG       60
TinfSat04-1000     ATCGTCGGAGCCATTTTTGAGAAAATCGCAAAAAAGTAAAAAAAAACAACTAGTAAAGTG       60
                   ************************************************************

TdelSat05-1000     GTACTTCCGGTTGAGGAATTTTGACAGATAACGTACTGTCAGATCCCATGGTGATACCTA       120
TinfSat04-1000     GTACTTCCGGTTGAGGAATTTTGACAGATAACGTACTGTCAGATCCCATGGTGATACCTA       120
                   ************************************************************

TdelSat05-1000     AACGGAATATCAAGTTTCAACTTTCTACGGTTTTTCGTTTTTGAGCTATGCTGTTCACAT       180
TinfSat04-1000     AACGGAATATCAAGTTTCAACTTTCTACGGTTTTTCGTTTTTGAGCTATGCTGTTCACAT       180
                   ************************************************************

TdelSat05-1000     ACATACATACACACACACACACACACACCATTTGCTAAAAACCACTTTTTTGGACTCAGG       240
TinfSat04-1000     ACATACATAC--------------------TTTGCTAAAAACCACTTTTTTGGACTCAGG       220
                   **********                    ******************************

TdelSat05-1000     GGACCTCAAAACGGATATTTCCGGTGAAAACTCGATATCGAAAATTTGACACGATTACAA       300
TinfSat04-1000     GGACCTCAAAACGGATATTTCCGGTGAAAACTCGATATCGAAAATTTGACACGATTACAA       280
                   ************************************************************

TdelSat05-1000     TACTTCCTCTTACTAGGAGTAAGAGAAAGTAAAAAAAAAAAAATCTGGTGTGAAACACTCA       360
TinfSat04-1000     TACTTCCTCTTACTAGGAGTAAGAGAAAGTAAAAAAAAAAAAATCTGGTGTGAAACACTCA       340
                   ************************************************************

TdelSat05-1000     CACAACTTTCTCTTACTCCAGTTCTCAAAATTATAATTGACAAATTTTATAGTTTGTGTA       420
TinfSat04-1000     CACAACTTTCTCTTACTCCAGTTCTCAAAATTATAATTGACAAATTTTATAGTTTGTGTA       400
                   ************************************************************

TdelSat05-1000     CCAAATTTAATGAAATTCGTCGAAAACTGAAAAAAACTGTTCAGTAAAAGCGCACTTCCG       480
TinfSat04-1000     CCAAATTTAATGAAATTCGTCGAAAACTGAAAAAAACTGTTCAGTAAAAGCGCACTTCCG       460
                   ************************************************************

TdelSat05-1000     GTTTACTAATTTTCTTGAAACTCGGAATTTTGGCCATCTATTTATTTCTAATTAATTTTG       540
TinfSat04-1000     GTTTACTAATTTTCTTGAAACTCGGAATTTTGGCCATCTATTTATTTCTAATTAATTTTG       520
                   ************************************************************

TdelSat05-1000     TTTGGATGATAGAATTGTATAATCAACTATGATAGCACTTTAGAGAAAAACATAACCCCA       600
TinfSat04-1000     TTTGGATGATAGAATTGTATAATCAACTATGATAGCACTTTAGAGAAAAACATAACCCCA       580
                   ************************************************************

TdelSat05-1000     CCCCTCCCCTAAAAGTGCCCTTAAATGAATTTTCCCAGAGAAATGTTTTAAATAAAAGTT       660
TinfSat04-1000     CCCCTCCCCYAAAAGTGCCCTTAAATGAATTTTCCCAGAGAAATGTTTTAAATAAAAGTT       640
                   ********* **************************************************

TdelSat05-1000     GTAGATCTTTGTATGTGTAGTTTATATACGAAGTTTCAAGAAAATCGTCGGAGCCATTTT       720
TinfSat04-1000     GTAGATCTTTGTATGTGTAGTTTATATAGGAAGTTTCAAGAAAATCGTCGGAGCCATTTT       700
                   **************************** *******************************

TdelSat05-1000     TGAGAAAATCGCGAAAAAGTGAAAAAAATCATTTAGTAAAGTGGTAAAGTAAACGCACTT       780
TinfSat04-1000     TGAGAAAATCGCGAAAAAGTRRAAAAAATCATTTAGTAAAGTGGTAAAGTAAACGCACTT       760
                   ********************  **************************************

TdelSat05-1000     CCGGTTATCCGATTTTTTTCAAACTCGGAATTTCAACAACATATTTAATTCTAATTAACA       840
TinfSat04-1000     CCGGTTATCCGATTTTTTTCAAACTCGGAATTTCAACAACATATTTAATTCTAATTAACA       820
                   ************************************************************
```

```
TdelSat05-1000      TTATTTAGATGACAGAATTGTCTAATAAACTGAAATAGCACTTTAGATGAAAACCTAACC      900
TinfSat04-1000      TTATTTAGATGAYAGAATTGTCTAATAAACTGAAATAGCACTTTAGATGAAAACCTAACC      880
                    ************ ***********************************************

TdelSat05-1000      CCACCCCTCCCCCAAAAGTGCCCTTAAATGAATTTTCCCGGAGAAATGTTTCAAATAAAA      960
TinfSat04-1000      CCACCCCTCCCCCAAAAGTGCCCTTAAATGAATTTTCCCRGAGAAATGTTTCAAATAAAA      940
                    *************************************** ********************

TdelSat05-1000      GTTGTAGCTCTTTGTATGTGTAGTTGATATACCAAGTTTAAAGAAA          1006
TinfSat04-1000      GTTGTAGCTCTTTGTATGTGTAGTTGATATACCAAGTTTAAAGAAA          986
                    **********************************************
```

```
TdelSat06-25 (4x)  AGAATGTATAACTTTGACAAATGTTAGAATGTATAACTTTGACAAATGTTAGAATGTATA      60
TinfSat09-113      AGAATGTAGAACTTTGAAAAATGTTAGAATGTAGAATTTTGCGTAATATTAGAATGTATA      60
                   ******** ******** *************** ** ****   *** ************

TdelSat06-25 (4x)  ACTTTGACAAA-------TGTTAGAATGTATAACTTTGACAAA-------TGTT   100
TinfSat09-113      ACTTTGGAAAACATGAATTATTAGAATGTAGAACTTT-ACAAATACTAATTATT   113
                   ******  ***       * ********** ****** *****       * **
```

```
TdelSat07-334      TAAACAGGCATAGGATATTATGCGCTAAAAAACATGTTACTTTTTAATCAAAGAATATTG      60
TinfSat52-334      TAAACAGGCATAGGATATTATGCGCTAAAAAACATGTTACTTTTTAATCGAAGAATATTG      60
                   ************************************************* **********

TdelSat07-334      GAACAAGTTAAACCATTATAAGTTTCTAATCTGGGGAAATGATTGCATTATATCCAAATT      120
TinfSat52-334      GAACAAGTTAAACCATTATAAGTTTCTAATCTGGGGAAATGATTGCATTATATCCAAATT      120
                   ************************************************************

TdelSat07-334      CGAAATCTTCTGTTTTGTAGCCATTGTCTAACGTCTATTAGTCCAAGAAAAACACAGATA      180
TinfSat52-334      CGAAATCTTCTGTTTTGTAGCCATTGTCTAACGTCTATTAGTCCAAGAAAAACACAGATA      180
                   ************************************************************

TdelSat07-334      TACAATAGAATATAAACATATATTGCACTAAAACATTCTTAAGTTATCGCATGGCAATTA      240
TinfSat52-334      TACAATAGAATATAAACATATATTGCACTAAAACATTCTTAAGTTATCGCATGGCAATTA      240
                   ************************************************************

TdelSat07-334      TTGTGGATTGCAATAACAGTTTTGCTAGTAATTTCCAAGTTCTTTCCTCAACCTTTCTAA      300
TinfSat52-334      TTGTGGATTGCAATAACAGTTTTGCTAGTAATTTCCAAGTTCTTTCCTCAACCTTTCTAA      300
                   ************************************************************

TdelSat07-334      ATATGTAACAACTTCTTCTGCCATCTTACATTCT  334
TinfSat52-334      ACATGTAACAACTTGTTCTACTGTCTTACATTCT  334
                   * ************ **** *  ***********
```

```
TdelSat08-247      GGCGCGTCAACATCTATGGTCATTGGCGCCCGGAGCCTTCTCAGTGCACTGCCGGCCTCC      60
TinfSat43-242      GGCGCGTCAACATCTATGGTCATTGGCGCCCGGAGCCTTCTCAGTGCACTGCCGGCCTCC      60
                   ************************************************************

TdelSat08-247      GGACAGTGTTCTCTCGTTACAGACAACACTGTCCATCACCGGACAGTGAGCTGTAGAGGC      120
TinfSat43-242      GGACAGTGTTCTCTCGTTACAGACAACACTGTCCATCACCGGACAGTGAGCTGTAGAGGC      120
                   ************************************************************

TdelSat08-247      TCCTAGGAAGAGAAAATCTAAGGGTAGCACAGCTGGACTCGAACCCGGGACCTCCGCGTG      180
TinfSat43-242      TCCTAGGAAGAAAAAATCTAAGGGCAGCAC-GCTGGACTCGAACCCGGGACCTCCGCGT-      179
                   *********** ************ ***** ****************************

TdelSat08-247      GGAGTCCAGTACGCTAACCACTTAGCTATTCCCTTCCCTTTATTATTATTATTATTTTTT      240
TinfSat43-242      GGAGTCCAGT-CGCTAACCACTGAGCTAATCCCTTCCCTTT--TTTTTTTTTTTTTTTTT      235
                   ********** *********** ***** ************  * ** ** ** ******

TdelSat08-247      AATTTAA       247
TinfSat43-242      AATTTAA       242
                   *******
```

```
TdelSat09-181      CGGCTCAAAAACAATATTAAAGTCCTTAGAATGTTTTTAGCTAAAATATTATAAGTGTAA      60
TinfSat06-181      CGGCTCAAAAACAATATTAAAGTCCTTAGAATGTGTTTAGCTAAAATATTATAAGTGTAA      60
                   ********************************** *************************

TdelSat09-181      ACATAATTTAAATATACATAAAAAAAAACAAAGTCTTTGTTCATAGTATTGGATGATAAT      120
TinfSat06-181      ACATAATTTAAACATACATAAAAAAAAACAAAGTCTTTGTTCATAGTATTGGATGATAAT      120
                   ************ ***********************************************

TdelSat09-181      ATATTTAATGTTTCTAGCTAAATATTATAAAATATTAGCCCTAGTCTTGTATGTCATCTC      180
TinfSat06-181      ATATTTAATGTTTTTAGCTAAATATTATAAAATATTAGCCCTCGTCTTGGATGTCATCTC      180
                   ************* **************************** ****** **********

TdelSat09-181      A  181
TinfSat06-181      A  181
                   *
```

```
TdelSat10-315     GCAGATATATGGCTAGCCAGAGTGGTAGTGGGCTGAAAGCCCGCTTAAAACTGGCTGATT        60
TinfSat37-314     GCATATATATGGCTAGCCAGAGTGGTCGTGGGCTGAAAGCCCGCTTAAAACTGGCTGATT        60
                  *** ********************** *********************************

TdelSat10-315     GGGCAACTGAGATTCAATCTGCTGACTATGCCATCACTGCCGGGGCGGCGGTGTCGGAGA        120
TinfSat37-314     GGGCTACTGAGGTTCAATCTGCTGACTATGCCATCACTGCCGGGGCGGCGGTGCCGGAGA        120
                  **** ****** ***************************************** ******

TdelSat10-315     AATCGGCGATTAAGGTCGATAACGCCACCTATACGGCAGGTAATGACATGACCATTAGTG        180
TinfSat37-314     AATCGGCGATTAAGGTCGATAACACCACCTATACGGCAGGTAATGACATGACCGTTAGTG        180
                  *********************** ***************************** ******

TdelSat10-315     TGACGCTGAAGGATGCGCAGGGTAATGGGGTTACTGGCCAAGCTGCTGCCTTAACGTCCC        240
TinfSat37-314     TGACGCTGAAGGATGCGCAGGGTAATGGGGTTACTGGCCAAGCTGCTGCCTTAACGTCCC        240
                  ************************************************************

TdelSat10-315     AGGCGGTAACGGTGGCCAATGCCAGTGAGAAATCCGGTGTCACGTGGATCGATAACGGCG        300
TinfSat37-314     AGGCGGTAACGGTGGCCAATGCCAGTGAGAAAGACGGTGTCACGTGGATCGATAACGGCG        300
                  ********************************  **************************

TdelSat10-315     ACGGCACCTATAGCC          315
TinfSat37-314     A-GGCACCTATAGCC          314
                  * *************
```

```
TdelSat11-239     ACCCGCGCGCACCTATTCTAAAAATTAAATTCTCTGAAATAAAAGTAAAAGTAAAGAATG        60
TinfSat08-239     TCCCGCGCGAACCTATTCTAAAAACTAAATTCTCTGAAATAAAAGTAAAAGTAAAGAGTG        60
                   ******** ************** ******************************** **

TdelSat11-239     CCTATTTTTTCAGGCTGACGTAACATTCAACTAACAATTAGGTATCGTGGACGGGGGCAG        120
TinfSat08-239     CCTATTTTTTCAGGCTGACGTAACATTCAACTAACAATTAGGTATCGTGGTAGGGGGCAC        120
                  **************************************************  ******* 

TdelSat11-239     AAACTCCTACTTCACTATTAACATTTTTCCATACCAATATAAAATTTAGCAACTATACGC        180
TinfSat08-239     AAACTCCTACTTTACTATTAACATTTT-CCATACCGATATAAAATTTAGCAACTCGACGC        179
                  ************ ************** ******* ******************  ****

TdelSat11-239     GGGACTCGAACTGGGGACCTCCCGCGTTCGAGTCCATGCTCACAACCACTAATGAATTAA        240
TinfSat08-239     GGGACCCGAACTGGGGACCTCCCGCGTTCGAGTCCATGCTCACAACCACTAATGAAATAA        239
                  ***** ************************************************** ***
```

```
TdelSat12-52     CACCTCCTACAGCACTAAGAGAAAGTCGTGTGATTACACATCAGATTTATAT      52
TinfSat32-52     CACCTCCTACAGCACTAAGAGAAAGTCGTGTGATTACACATCAGATTTGTAT      52
                 ************************************************ ***
```

```
TdelSat14-94     CGGAAAATGTCTCTGATAAAG---AATATTAT-GAATGTAAAGAATGTAAAGAATATTAG         56
TinfSat45-94     CGGAAAATGTCTCTGATGCTTTCCAGCTCTGCGGAA-CCAACCACCACAAAACGCCCGAA         59
                 *****************       *    *   ***   **  *    ***       *

TdelSat14-94     AATAAAGAATGTAAAAAACAAAATGAAGCAGGCTACCA         94
TinfSat45-94     ACCGGTCGATG---ACCACAAAATGAAGCAGGCTACCA
                 *       ***   *  *********************
```

```
TdelSat15-93     AGAGTGTGTGGTTTCCTCAACAAATCCATTTTATTAAGTTTAGATTCCTCCCAATGACAC         60
TinfSat49-94     GGAGTGTATGGTTTCCTCAAAAAAGTCATTTTATTAAGTTTAGATTCTCCAATTGATATA         60
                  ****** ************ ***  *********************  *

TdelSat15-93     ACAATGTAAACAAGTACACATT-AGGGGAGAGGG   93
TinfSat49-94     AAATTGAAGACAATTAAACATTTTGGAGAGAGGG   94
                 * * ** * **** ** *****  ** *******
```

```
TdelSat17-84     AAAAGACATATGCGAACACATACAGGCGAGAAGCCATATAAATGTAGTGAATGCGATTAC         60
TinfSat12-84     AAAAAACATATGCGAACACATACAGGCGAGAAGCCATATAAATGTAGTGAATGTGATTAC         60
                 **** ************************************************ ******

TdelSat17-84     AGTTGTACAACAACTGGAAATTTA  84
TinfSat12-84     AGTTGTACACAATCTGGAAATCTT  84
                 *********  * ******** *
```

```
TdelSat18-62     TTTTCTGCGATTTTCTCCATAACGGTGGACTAGAAGTGCTACTTCACTGGTGAAGTTCTT         60
TinfSat25-62     TTTTCTGCGATTTTCTCCATAACGGTGGACTAGAAGTGCTACTTCACTGGTGAAGTTCTT         60
                 ************************************************************

TdelSat18-62     TG  62
TinfSat25-62     CG  62
                  *
```

```
TdelSat19-58     TAATCTGAGTTGGAACTATCCATTGTAGTGAACCACATATTTGTTACTATCAGCGTAT 58
TinfSat30-58     TAATCTGAGTTGGAACTATCCATTGTAGTGAACCACATATTTGTTACTATCAGCGTAT 58
                 **********************************************************
```

```
TdelSat21-33     TCTACACACATAAATACCTATCACTGTATACTA    33
TinfSat60-33     TCTACACACATAAATACCTATCACTGTATACTA    33
                 *********************************
```

```
TdelSat22-104     GCTTAGTCAGTTCAATTATACATTTAGTTGGCTGAATGGTGAGCAACCTAATGTTTGTAT        60
TinfSat19-104     GCTTAGTCAGTTCAATTATACATTTAGTTGGCTGAATGGTGAGCAACCTAATGTTTGTAT        60
                  ************************************************************

TdelSat22-104     TTATTAGCGAGAATGTGATGATAGGTTAGAGTGTGCTTGTATGG  104
TinfSat19-104     TTATTAGCGAGAATGTGATGATAGGTTAGAGTGTGCTTGTATGG  104
                  ********************************************
```

```
TdelSat23-112     GACACTAAGTTATCTGTCAAAATTCCACAACCGGAACAAACACAACCACAACAAAAACAA        60
TinfSat42-112     GACACTAAGTTATCTGTCAAAATTCCACAACCGGAACAAACACAACCACAACAAAAACAA        60
                  ************************************************************

TdelSat23-112     AAATCGCAGAAAATTCAAACTTGACATTCTGTTTAGATATCACCGTGGGTTC     112
TinfSat42-112     AAATCGCAGAAAATTCAAACTTGACATTCTGTTTAGGTATCACCGTGGGTTC     112
                  ************************************ ***************
```

```
TdelSat24-147     CCAAGAAAGTTCATTTTATGGCGTTTTGGGTGGGGGGTAAGGGGCAAGCTTTTCCCAAAA        60
TinfSat14-147     CCAAGAAAGTTCATTTTATGGCGTTTTGGGTGGGGGGTAAGAGGCAAGCTTTTCCCAAAA        60
                  ***************************************** ******************

TdelSat24-147     TAGTGGTTTTTATTGTTTAGATTCTCCCACTGATGTAAAATTGAAGACAACTAAACATCT        120
TinfSat14-147     TAGTGGTTTTTATTGTTTAGATTCTCCCACTGATGTAAAATTGAAGACAACTAAACATCT        120
                  ************************************************************

TdelSat24-147     AGTGAGAGAATTAGACTTTTGAAAGTT        147
TinfSat14-147     AGTGAGAGAATTAGACTTTTGAAAGTT        147
                  ***************************
```

```
TdelSat29-137     TAACAGGAGTAAGAGAAAGTCGTGTGAGTGTCCCCCACCACCTCCCCCTCCTAACATCAC        60
TinfSat51-137     TAACAGGAGTAAGAGAAAGGCGTGTGAGTGTCCCCCACCACCTCCCCCTCCTAACATCAC        60
                  ******************* ****************************************

TdelSat29-137     CAAACTATATGTTAGGACAAATGAGTAAAATTGGAAGTTTAAAGTAAATTCGTCACTTTA        120
TinfSat51-137     CAAACTATATGTTAGGACAAATGAGTAAAATTGGAAGTTTAAAGTAAATTCGTCACTTTA        120
                  ************************************************************

TdelSat29-137     TATATATCTCATTGTAG       137
TinfSat51-137     TATATATCTCATTGTAG       137
                  *****************
```

```
TdelSat32-49     GAAGCTGTACAATAGTCAATTAGAATTAACAGAAAATTATGTGAAAGGT          49
TinfSat16-49     GAAGTTGTACAATAGTCAATTAGAATTAACAGAAAATTATGTGAAAGGT          49
                 **** ********************************************
```

```
TdelSat36-128     TCTCAATTACTCATATGTCCTAATATAAATATACAGGTGGTGGGAGACAAGTGCCAGCCT        60
TinfSat53-128     TTTCAATTACTCATATGTCCTAATATAAATATACAGGTGGTGGGAGACAAGTGCCAGCCT        60
                  * **********************************************************

TdelSat36-128     AGCAACACAGACAGGTACAGAATGATTACATTGTAGACAGCCAATAATATTGAACAAACA        120
TinfSat53-128     AGCAACACAGACAGGTACAGAATGATTACATTGTAGATAGCCAATAATATTGAACAAACA        120
                  ************************************* **********************

TdelSat36-128     CATTTGAA      128
TinfSat53-128     CATTTGAA      128
                  ********
```

```
TdelSat38-147     GTTATCTGGTTGGCTTTTTTCCTGCGTTTCAGATTTCGATTCCTTTTCTGCCGCAGTTTT        60
TinfSat13-147     GTTATCTGGTTGGCTTTTTTCCTGCGTTTCAGATTTTGATTCCTTTTCTGCCGCAGTTTT        60
                  ************************************ ***********************

TdelSat38-147     TGATCCCGCTTGAATGAATGGTGGGATTGGTGGAACATTGTCTATTTCAGATATCGATTT        120
TinfSat13-147     TGATCCCGCTTGAATGAATGGTGGGATTGGCGGAACATCGTCTATTTCAGATATCGATTT        120
                  ****************************** ******* *********************

TdelSat38-147     GTCAGCATCTTCACCTTCATTACTTGA        147
TinfSat13-147     GTCAGCATCTTCACCTTCATTACTTGA        147
                  ***************************
```

```
TdelSat45-102     TTCGCGGAAAAAAGTTCCCAATAAAAGTGACGGCATTTGCATTCACAGTTACTGTAGGAC        60
TinfSat18-102     GTCGCGGAAAAAAGTTCCCAATAAAAGTGACGGCATTTGCATTCACAGTTACTGTAGGAC        60
                   ***********************************************************
```

```
TdelSat45-102     GTTTAGTGAAATCCGTTGCCATTTTTACTAAATAAATCAACT    102
TinfSat18-102     GTTTAGTGAAATCCGTTGCCATTTTTACTAAATAAATCAACT    102
                  ******************************************
```

```
TdelSat46-114     TATCGTTACAACTATTATTAATCTCATTACAGAGAATCTCTATATCGTTTTTAGCAGAAC        60
TinfSat67-114     TATCGTTACAACTATTATTAATCTCATTACAGAGAATCTCTATATCGTTTTTAGCAGAAC        60
                  ************************************************************

TdelSat46-114     ATTCTACAATAACTCCGCCATTAGAGATTCCCTTAATGTTTTCTACACCCAATT   114
TinfSat67-114     ATTCTACAATAACTCCGCCATTAGAGATTCCCTTAATGTTTTCTACACCCAATT   114
                  ******************************************************
```

```
TdelSat48-118     TAACTGGAGTAAGAGAAAGTCGTGTGAATATTTCACACCAGTTTTACTTTGAAACACCTG        60
TinfSat56-118     TAACTGGAGTAAGAGAAAGTCGTGTGAATATTTCCCACCAGTTTTACTTTGAAACACCTG        60
                  ********************************** *************************

TdelSat48-118     GTAGCAAGTTAATGGTTCCATTTACTGCTGTACAAGCGATTGAACAGTTAAATTGTAT          118
TinfSat56-118     GTAGCAAGTTAATGGTTCCGTTTACTGCTGTACAAGCGATTTAACAGTTAAATTGTAT          118
                  ******************* ******************** *****************
```

```
TdelSat50-101     ATATTTGTAGCAAAATAGTTAATTCTAAAGTAGGGGGGGGAATGCAGAGAGCAGTCAACT        60
TinfSat96-101     ATATTTGTAGCAAAATAGTTAATTCTAAAGTAGGGGGAGGAATGCAGAGAGCAGTCAAAT        60
                  ************************************* ******************** *

TdelSat50-101     TCATTTGAACATAAACATCATTTATTATGTTAACTTACACT      101
TinfSat96-101     TCATTTGAACATAAACATCATTTATTATGTTAACTTACACT      101
                  *****************************************
```

```
TdelSat51-46     ATGTACAGCCGATACAGAAAGTGTAAATAAAGTTGTCGCTCTTTGT  46
TinfSat28-46     ATGTACAGCCGATACAGAAAGTTTAAATAAAGTTGTCGCTCTTTGT  46
                 ********************** ***********************
```

```
TdelSat52-88     AGGTGAGTGTTGTACTAAAATGAAATTTTTAGATGAATTTTGGTGAAAATAGTGGATCTA         60
TinfSat64-88     AGGTGAGTGTTGTACTAAAATGAAATTTTTAGATGAATTTTGGTGAAAATAGTGGATTTA         60
                 ********************************************************* **

TdelSat52-88     GTATTTTATTGTACATTAAACACAACTG        88
TinfSat64-88     GTATTTTATTGTACATTAAACGCAACTG        88
                 ********************* ******
```

```
TdelSat59-63     CGTGGCCTAAGTGCTCGTCCAGTTCGATGATTGGTCAGTTGGAGAGGGGTGGGGGTAAGT         60
TinfSat50-63     CGTGGCTTAAGTGCTCGTCCAGTTCGATGATTGGTCAGTTGGAGAGGGGTGGGGGTAAGT         60
                 ****** *****************************************************

TdelSat59-63     GGG 63
TinfSat50-63     GGG 63
                 ***
```

```
TdelSat60-99     TACGTATTGCGTCATACCGTGCAACATGACATGTCTCAACATGTTCATTTTAATTTATTT         60
TinfSat15-99     TACGTATTGCGTCATACCGTGCAACATGACATGTCTCAACATGTTCATTTTAATTTATTT         60
                 ************************************************************

TdelSat60-99     CTTACTCTTGCTAAAACATCGAAAAAAATACTAGATTTG        99
TinfSat15-99     CTTACTCTTGCTAAAACATCGAAAAAAATACTAGATTTG        99
                 ***************************************
```

```
TdelSat61-315     ACAATTTATAAGGCCGAGACTGTAGGTGAAAACCTGAAAGCGACTGTAAAGCTTCCAGAT        60
TinfSat38-315     ACAATTTATAAGGCCGAGACTGTAGGTGAAAACCTGAAAGCGACTGTAAAGCTTCCAGAT        60
                  ************************************************************

TdelSat61-315     TGGAAGGGTACAACTGAATCAGCTAATTACGCAATTACTACCGGTAACCCTGCGTATATG        120
TinfSat38-315     TGGAAGGGTACAACTGAATCGGCTAATTACGCAATTACTACCGGTAACCCTGCGTATATG        120
                  ******************** ***************************************

TdelSat61-315     AAGTCAACAATTACGTTAGATAAAGACATCTATCCCGCAAATAGTGATATGAAGGTTACG        180
TinfSat38-315     AAGTCAACGATTACGTTAGATAAAGACATCTATCCCGCAAATAGTGATATGAAGGTTACG        180
                  ******** ***************************************************

TdelSat61-315     GTAACCTTGAAAGATTTGTATGGTAATAGCGTAACTGGAAAAGTTGCAGAATTAACTGAT        240
TinfSat38-315     GTAACCTTGAAAGATTTGTATGGTAATAGCGTAACTGGAAAAGTTGCAGAATTAACTGAT        240
                  ************************************************************

TdelSat61-315     GCGGTAGTAACAGTACCAAATGCAAAAATGAAAATAGGTGAAAAATGGAAAGAAAATGAA        300
TinfSat38-315     GCGGTAGTAACAGTACCAAATGCAAAAATGAAAATAGGTGAAAAATGGAAAGAAAATGAA        300
                  ************************************************************
```

```
TdelSat61-315      GCTGGGACCTATACT       315
TinfSat38-315      GCTGGGACCTATACT       315
                   ***************
```

```
TdelSat66-64       AATGTGCAAAATCTAAGTAATATTTATGACAAAGTTCTACATTCTAATACTAATATAAAA       60
TinfSat22-64       AATGTGCAAAATCTAAGTAATATTTATGACAAAGTTCTACATTCTAATACTAATATAAAA       60
                   ************************************************************

TdelSat66-64       TGGA       64
TinfSat22-64       TGGA       64
                   ****
```

```
TdelSat67-83       AAAAACATATACATATCACCCCTGTACTATTGATTAAGCTCCCAAATCTCTTTAACATAA       60
TinfSat85-83       AAAAACATATACATATCACCCCTGTACTATTGATTAAGCTCCCAAATCTCTTTAACATAA       60
                   ************************************************************

TdelSat67-83       CTTTACACTATTACTATACTTAG   83
TinfSat85-83       CTTTACACTATTACTATACTTAG   83
                   ***********************
```

```
TdelSat68-160      TAGTGCTGCGTTTGTGCCGGCGATTCACCAAATTTCATCGTTTGTGCCGTAAAATTGACA       60
TinfSat66-161      TAGTGCTGCGTTTGTGCCGGCGATTCACCAAATTTCATCGTTTGTGCCGTAAAATTGACA       60
                   ************************************************************

TdelSat68-160      AAGATATTAAAGAAAAAAAGTGGCCAGTCCAACTTGACGTCAAGTTAGACTGGCCACCAA       120
TinfSat66-161      AAGATATTGAAGAAAAAAAGTGGCCAGTCCAACTTGACGTCAAGTTAGACTGGCCACAAA       120
                   ******** ************************************************* **

TdelSat68-160      -TTTCTATTCACTTTTTTAAAATTTTCTTTTTACCAATCGT      160
TinfSat66-161      ATTTCTATTCACTTTTTTAAACTTTTCTCTTTGCCAATCGT      161
                    *** ***************** ****** *** ********
```

```
TdelSat70-86       TACATAAACGGAATACCAAGTTTGACATTCCGTTTAGGTATCACAAACTGCCACTAACCT       60
TinfSat47-87       TACCTAAACGGAATACCAAGTTTGAGCTTCCTGCAATTTTCGTTTTTGGATTTTTCCCGA       60
                   *** *********************  ****    *  *               *  *

TdelSat70-86       TCCGTTCGTGT-TAGAAGCAGTGGTAC         86
TinfSat47-87       AATCTTCGTGTCTAGATGCGGTGGTAG         87
                       ******* **** ** ******
```

```
TdelSat73-372      TAGTGCTGCGTTTGTGCCGGCGATTCACCAAATTTCAACGTTTGTGCCGTAAAATTGACA       60
TinfSat33-372      TAGTGCTGCGTTTGTGCCGGCGATTCACCAAATTTCATCGTTTGTGCCGTAAAATTGACA       60
                   ************************************* **********************

TdelSat73-372      AAGATATTGACGAAAAACTGTTCGTCGTACTTGAAAACCAAAGGCCATGGCTGCGAGTAA       120
TinfSat33-372      AAGATATTAAGGAAAAACTGTTCGTCGTACTTGAAAACCAAAGGCCATGGCTGCGAGTAA       120
                   ******** * *************************************************

TdelSat73-372      CATTTGAATGTGCGCGTGCTCCACTGACCGCCAACGGCCCTTATCAGGGCCAGCAACTAA       180
TinfSat33-372      CATTTGAATGTGCGCGTGCTCCACTGACCGCCAACGGCCCTTATCAGGGCCAGCAACTAA       180
                   ************************************************************

TdelSat73-372      AATGTACTGGCCCTCGATTTCTAATCAAAATTCTGGAATTTTTTCTTCGCTATTCTTAAG       240
TinfSat33-372      AATGTACTGGCCCTCGATTTCTAATCAAAATTCTGGAATTTTTTCTTCGCTATTCTTAAG       240
                   ************************************************************

TdelSat73-372      TGATTCATTTGTGCCAGCGACCCACCAAATTTCAGCGTTTGTGCTGCAAAATTGACAAAG       300
TinfSat33-372      TGTTTCATTTGTGCCAGCGACCCACCAAATTTCAGCGTTTGTGCTGCAAAATTGACAAAG       300
                   ** *********************************************************

TdelSat73-372      TTATTGAGGCGTCAAGTTAGACTGGCCACCAATTTCTATTCACTTTTTTAAACTTTTCTC       360
TinfSat33-372      TTATTGAGGCGTCAAGTTAGACTGGCCACCAATTTCTATTCACTTTTTTAAACTTTTCTC       360
                   ************************************************************

TdelSat73-372      TTTGCCAATCGT  372
TinfSat33-372      TTTGCCAATCGT  372
                   ************
```

```
TdelSat76-96       GGCAAGAAAGGTAATGAGTACAAAGTTTATTTTTACATAATAGGATTAAGTATTACTAA       60
TinfSat58-96       GGCAAGAAAGGTAATGAGTACAAAGTTTAATTTTTACATAATAGGATTAAGTATTACTAA       60
                   **************************** *******************************

TdelSat76-96       ACATGTTAATAAGACCTGTGGTAACATGTCCGAACA 96
TinfSat58-96       ACATGCTAATAAGACCTGTGGTAACATGTCCGGACA 96
                   ***** ************************** ***
```

```
TdelSat86-164     AATTGTTTGGAGTTAAATAAGGTGAGTGGCCAGTGCTGCTGTGCTGGTCACGTTTGTAGC        60
TinfSat57-164     AATTGTTTGGAGTTAAATAAGGTGAGTGGCCAGTGCTGCTGTGCTGGTCACGTTTGTAGC        60
                  ************************************************************

TdelSat86-164     TTTGTTTAACATAATCCTTCAATTCTACCCACAGCTTGCCGTTATGTTAGTTCTTAGGAA        120
TinfSat57-164     TTTGTTTAACATAATCCTTCAATTCTACCCACAGCTTGCCGTTATGTTAGTTCTTAGGAA        120
                  ************************************************************

TdelSat86-164     TTAACATATATATGCGGCGCCTCTTTTATTTTATTTTAGCGTAG  164
TinfSat57-164     TTAGCATATATATGCGGCGCCCTTTTATATATATATTAGTGTAG  164
                  *** *****************  ***   * *** **** ****
```

```
TdelSat88-87      TAACTGCAATGAGACCAAGTCGTCTGAATGTTT--CACACCATTGAGTAACATTACAATA        58
TinfSat55-89      TAACTGCAGTGAGAGTAAGTTGTGTGAATGTTTTTCACACCATTGAGTAACATTCCATTA        60
                  ******** *****  **** ** *********  ******************* ** **

TdelSat88-87      TAAAGAAAATTTGTAAATTGTAATTGTAT       87
TinfSat55-89      TAAAGAAAATTTGTCAATTGTAATTGTAT       89
                  ************** **************
```

```
TdelSat94-72      TCCAGCCACACTAACTTCAAATATTAGAATAGTTTTCGCCATTAAACCATATCACCAATC        60
TinfSat79-72      TCCAGCCACACTAACTTCAAATATTAGAATAGTTTTCGCCATTAAACCATATCACCAATC        60
                  ************************************************************

TdelSat94-72      AACGTTCTAACA   72
TinfSat79-72      AACGTTCTAACA   72
                  ************
```

```
TdelSat106-44     TAGGTAGTGCTAACACCAACAGAAT---CAATATTACCCCACCACTC          44
TinfSat27-47      TAGGTAGTGCTAACACCAACAGAATAATTAATATTACCCCACCACTC          47
                  *************************    ******************
```

```
TdelSat111-7      ACAATTT       7
TinfSat95-7       ACAATTT       7
                  *******
```

```
TdelSat118-207    TCAGTGGGCTTTTCGGTTACTCTAGTTGTAGCTTCAGTATCATGAATTGTAGATTGTTCT        60
TinfSat82-207     TCAGTGGGCTTTTCGGTTACTCTAGTTGTAAATTCAGTATCATGAATTGTAGATTCTTCT        60
                  ******************************  *********************** ****

TdelSat118-207    GTGATAAGTGTAGTCGGTTGCTCAGTGTTTGTTGTAAATTCATCAGCGGTTTGTGCATCC        120
TinfSat82-207     GTGATATGTGTAGTCGGTTGCTCAGTGTTTGTTGTAAATTCTTCAGCGGTTTGTGCATCC        120
                  ****** ********************************** ******************

TdelSat118-207    GTGCTAGATCCTATGTAAGATGCAGTATGGTCATTTGTGTAATATTTCGGGTTATTTGGT        180
TinfSat82-207     GTGCTAGATCCTATGTAAGATGCAGTATGGTCATTTGTGTAATATTTCGGGTTATTTGGT        180
                  ************************************************************

TdelSat118-207    GTATTCGGTTTGAATGTTGTTATAATA       207
TinfSat82-207     GTATTCGGTTTGAATGTTGTTGTAATA       207
                  ********************* *****
```

```
TdelSat119-348    GTGAAGCCTATCACAACTTCGGCAACCTACACCGAAGAGTTCTCAACAATACAATCTTCA        60
TinfSat78-348     GTGAAGCCTATCACAACTTCGGCAACCTACACCGAAGAGTTCTCAACAATACAATCTTCA        60
                  ************************************************************

TdelSat119-348    TCACCTCATTCCACAGAACCAACATCAGAAAGTTATACTACTAACAGCCATCGCACAACT        120
TinfSat78-348     TCACCTCATTCCACAGAACCAACATCAGAAAGTTATACTACTAACAGCCATCGCACAACT        120
                  ************************************************************

TdelSat119-348    GAATCAGAATCTACACCAATCACACAATCAAGCCCAGAGATACATACCATATTTACGTCC        180
TinfSat78-348     GAATCAGAATCTACACCAATCACACAATCAAGCCCAGAGATACGTACCACATTTACGTCC        180
                  ******************************************* ***** **********

TdelSat119-348    GCGTCCCCATCTACTGAGAACAGTGCAACAACAACCGCCACCGCGCACACATCACAGCCT        240
TinfSat78-348     GCGTCCCCATCTACTGAGAACAGTGCAACAACAACCGCCACCGCGCACACATCACAGCCT        240
                  ************************************************************

TdelSat119-348    AGCTCTGAAACATTCAATACAATTAAATACACTACGGGTCCGGGCACTGAAACCTATTCA        300
TinfSat78-348     AGCTCTGAAACATTCAATACAATTAAATACACTACGGGTCCGGGCACTGAAACCTATTCA        300
                  ************************************************************

TdelSat119-348    ACAATAGTGGGTTCAGGCTCGACATCTCACAGCGAAGGTTATACTTCC          348
TinfSat78-348     ACAATAGTGGGTTCAGGCTCGACATCTCACAGCGAAGGTTATACTTCC          348
                  ************************************************
```

```
TdelSat148-53     CAGTAAGAGAAAGTGGGGAAATGTATCACATTCAGTTGGCAAGAACTAAATTG     53
TinfSat26-53      CAGTAAGAGAAAGTGGGGAAATGTATCACATTCAGTTGGCAAGAACTAAATTG     53
                  *****************************************************
```

```
TdelSat159-241      AATGTATTGATAATGAATTTCTAAACGAAAATTCAACAATAAACGTGTTAGGATTTACGG       60
TinfSat63-237       AATATATTTATAATGAATTTCTAAAGAAAATTCGGAAAACACAATGCGTTAGGTTAAAGG       60
                    *** **** ****************  *** *   * ** * *     *  * ** * **

TdelSat159-241      CAAATATTTTA-CAATTAAATGCAACACGAAATCCAACAAAA-TTCAATAATTCTGTTTAC      119
TinfSat63-237       CAAATATTTAACCAA---AATGCAACACGAAATCTAACAAAAGAATAATTCTGTTTACTAC      118
                    ********* * ***   **************** *******    ***  *  *   ***

TdelSat159-241      AACGAAACAGACAAAAATACATTAGATTTACGGCAAATATTTAACAAAAATGTATAGCAA       179
TinfSat63-237       GAAACAGACGACAAAAATACATTAGATTTACGGCAAATATTTAACAAAAATGTATAGCAA       178
                     *   *   ***************************************************

TdelSat159-241      ATAAATGCAACACGAAATCTAACAAAA-GAATTAATAATTCTGTTTACAACGAAACAGACA      239
TinfSat63-237       ATAAATGCAACACGAAATCTAACAAAAAGAA----TAATTCTGTTTACTACGAAACAGACA      235
                    *************************** ***    ************* ************

TdelSat159-241      AA           241
TinfSat63-237       AA           237
                    **
```
