# Supplementary material for: Making the Genome Huge: The Case of Triatoma delpontei, a Triatominae Species with More than 50% of Its Genome Full of Satellite DNA
Source: Genes (Basel). 2023 Jan 31;14(2):371. doi: 10.3390/genes14020371 (PMC9957312; doi:10.3390/genes14020371)
Supplement: Supplementary file 1 [file genes-14-00371-s001.zip › Figure S3 T delpontei landscapes.pdf]

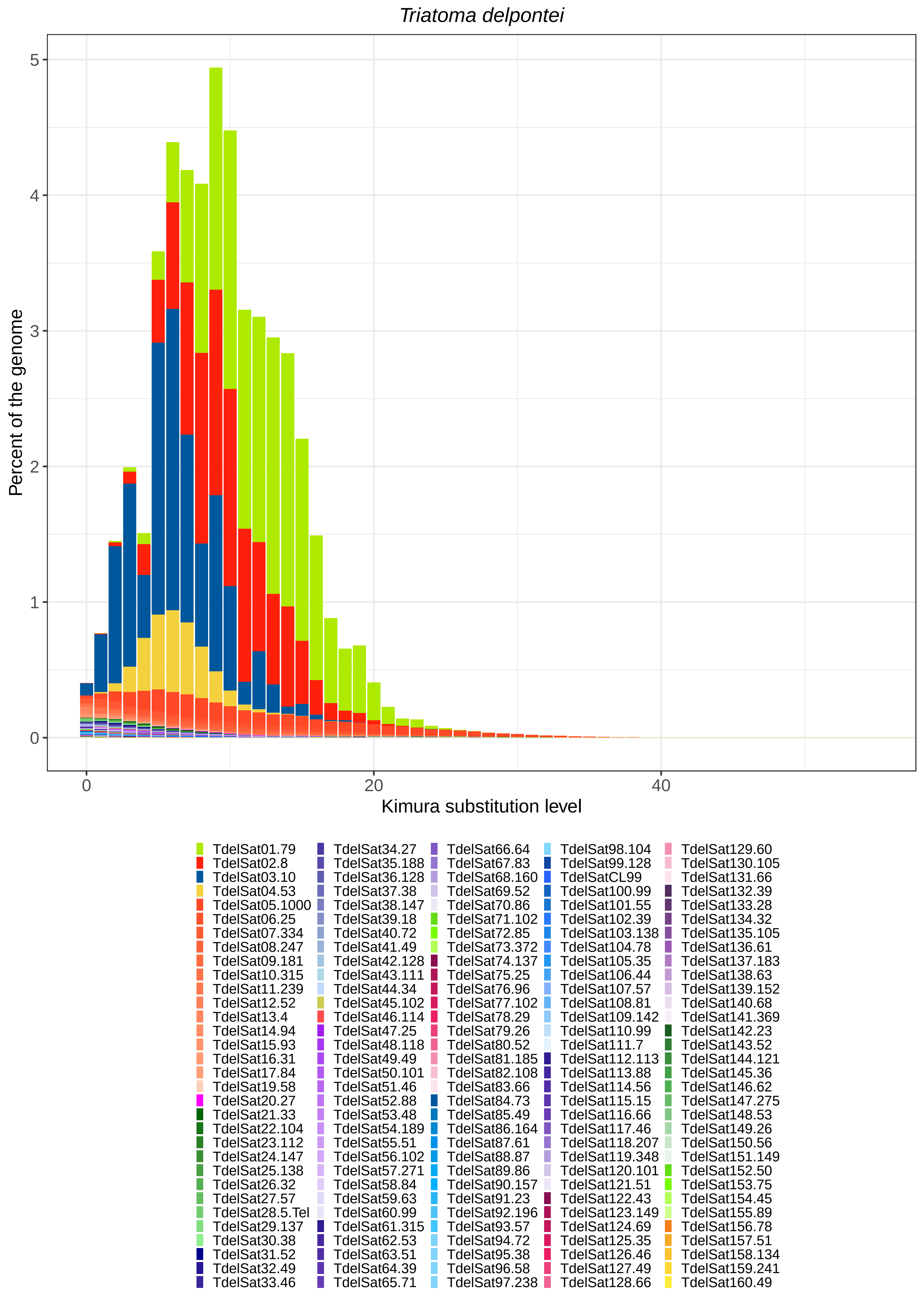

Triatoma delpontei
Percent of the genome
0
1
2
3
4
5
Kimura substitution level
0
20
40
TdelSat01.79
TdelSat02.8
TdelSat03.10
TdelSat04.53
TdelSat05.1000
TdelSat06.25
TdelSat07.334
TdelSat08.247
TdelSat09.181
TdelSat10.315
TdelSat11.239
TdelSat12.52
TdelSat13.4
TdelSat14.94
TdelSat15.93
TdelSat16.31
TdelSat17.84
TdelSat19.58
TdelSat20.27
TdelSat21.33
TdelSat22.104
TdelSat23.112
TdelSat24.147
TdelSat25.138
TdelSat26.32
TdelSat27.57
TdelSat28.5.Tel
TdelSat29.137
TdelSat30.38
TdelSat31.52
TdelSat32.49
TdelSat33.46
TdelSat34.27
TdelSat35.188
TdelSat36.128
TdelSat37.38
TdelSat38.147
TdelSat39.18
TdelSat40.72
TdelSat41.49
TdelSat42.128
TdelSat43.111
TdelSat44.34
TdelSat45.102
TdelSat46.114
TdelSat47.25
TdelSat48.118
TdelSat49.49
TdelSat50.101
TdelSat51.46
TdelSat52.88
TdelSat53.48
TdelSat54.189
TdelSat55.51
TdelSat56.102
TdelSat57.271
TdelSat58.84
TdelSat59.63
TdelSat60.99
TdelSat61.315
TdelSat62.53
TdelSat63.51
TdelSat64.39
TdelSat65.71
TdelSat66.64
TdelSat67.83
TdelSat68.160
TdelSat69.52
TdelSat70.86
TdelSat71.102
TdelSat72.85
TdelSat73.372
TdelSat74.137
TdelSat75.25
TdelSat76.96
TdelSat77.102
TdelSat78.29
TdelSat79.26
TdelSat80.52
TdelSat81.185
TdelSat82.108
TdelSat83.66
TdelSat84.73
TdelSat85.49
TdelSat86.164
TdelSat87.61
TdelSat88.87
TdelSat89.86
TdelSat90.157
TdelSat91.23
TdelSat92.196
TdelSat93.57
TdelSat94.72
TdelSat95.38
TdelSat96.58
TdelSat97.238
TdelSat98.104
TdelSat99.128
TdelSatCL99
TdelSat100.99
TdelSat101.55
TdelSat102.39
TdelSat103.138
TdelSat104.78
TdelSat105.35
TdelSat106.44
TdelSat107.57
TdelSat108.81
TdelSat109.142
TdelSat110.99
TdelSat111.7
TdelSat112.113
TdelSat113.88
TdelSat114.56
TdelSat115.15
TdelSat116.66
TdelSat117.46
TdelSat118.207
TdelSat119.348
TdelSat120.101
TdelSat121.51
TdelSat122.43
TdelSat123.149
TdelSat124.69
TdelSat125.35
TdelSat126.46
TdelSat127.49
TdelSat128.66
TdelSat129.60
TdelSat130.105
TdelSat131.66
TdelSat132.39
TdelSat133.28
TdelSat134.32
TdelSat135.105
TdelSat136.61
TdelSat137.183
TdelSat138.63
TdelSat139.152
TdelSat140.68
TdelSat141.369
TdelSat142.23
TdelSat143.52
TdelSat144.121
TdelSat145.36
TdelSat146.62
TdelSat147.275
TdelSat148.53
TdelSat149.26
TdelSat150.56
TdelSat151.149
TdelSat152.50
TdelSat153.75
TdelSat154.45
TdelSat155.89
TdelSat156.78
TdelSat157.51
TdelSat158.134
TdelSat159.241
TdelSat160.49

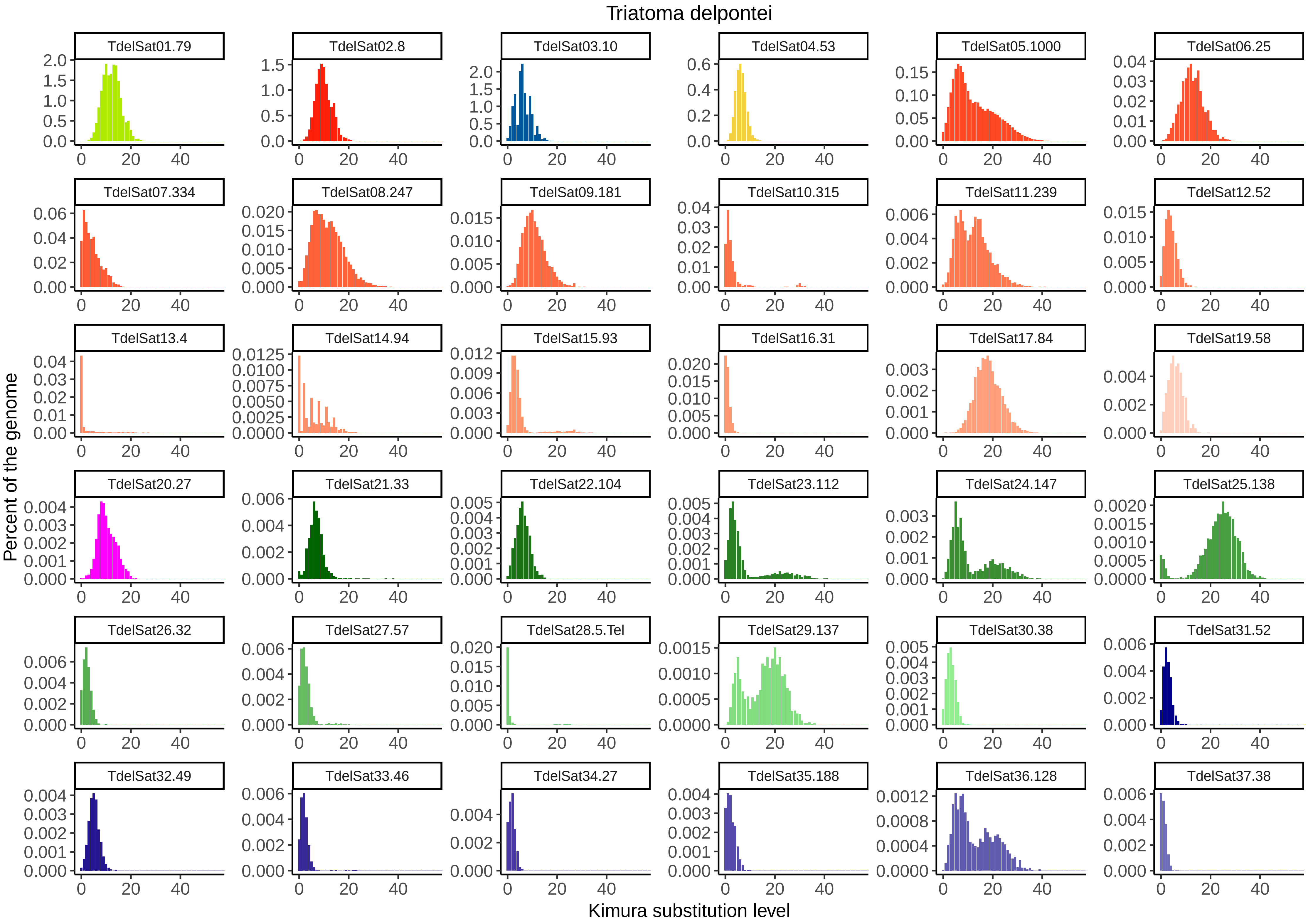
Triatoma delpontei
TdelSat01.79
TdelSat02.8
TdelSat03.10
TdelSat04.53
TdelSat05.1000
TdelSat06.25
TdelSat07.334
TdelSat08.247
TdelSat09.181
TdelSat10.315
TdelSat11.239
TdelSat12.52
TdelSat13.4
TdelSat14.94
TdelSat15.93
TdelSat16.31
TdelSat17.84
TdelSat19.58
TdelSat20.27
TdelSat21.33
TdelSat22.104
TdelSat23.112
TdelSat24.147
TdelSat25.138
TdelSat26.32
TdelSat27.57
TdelSat28.5.Tel
TdelSat29.137
TdelSat30.38
TdelSat31.52
TdelSat32.49
TdelSat33.46
TdelSat34.27
TdelSat35.188
TdelSat36.128
TdelSat37.38
Percent of the genome
Kimura substitution level

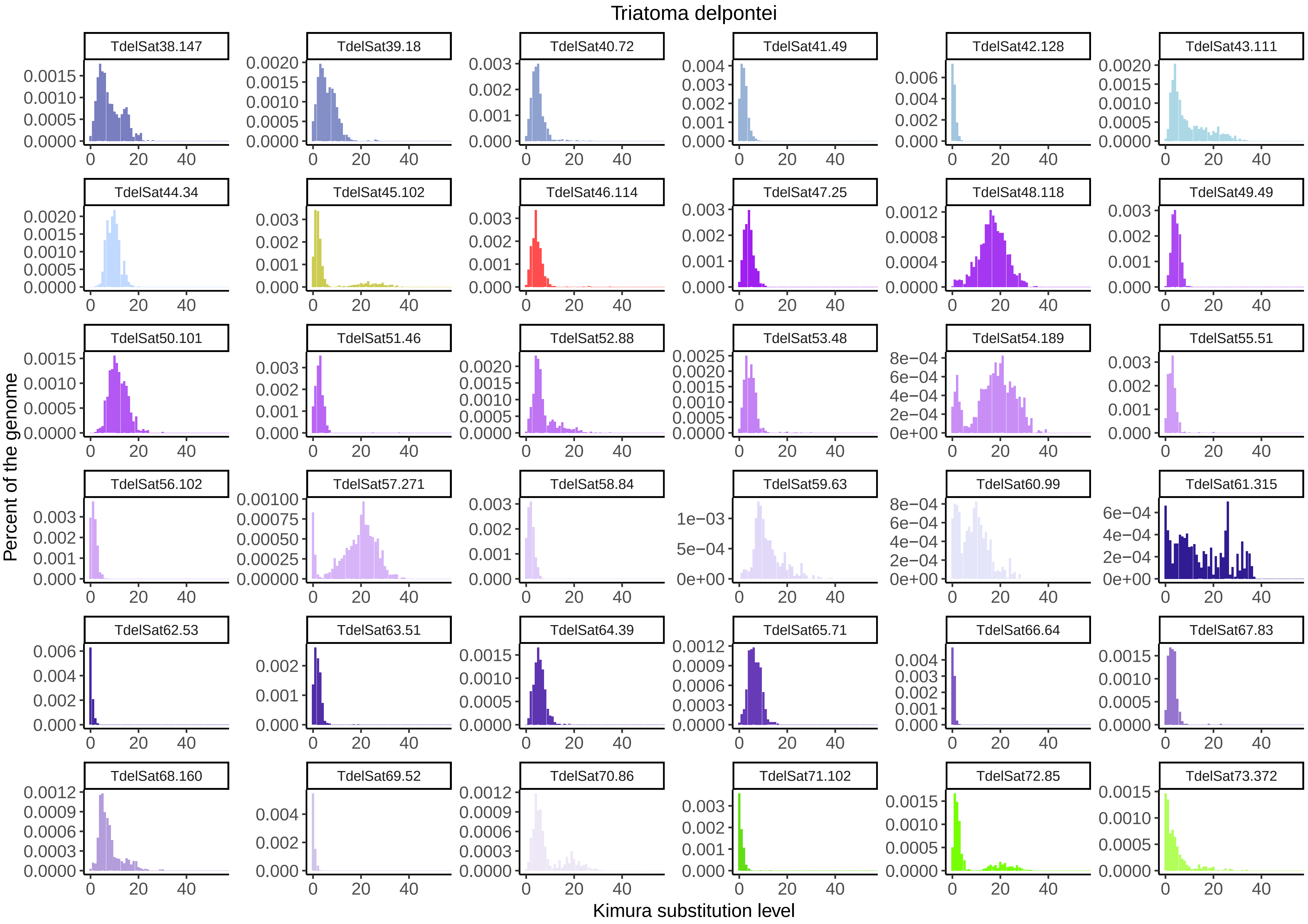
Triatoma delpontei
TdelSat38.147
TdelSat39.18
TdelSat40.72
TdelSat41.49
TdelSat42.128
TdelSat43.111
TdelSat44.34
TdelSat45.102
TdelSat46.114
TdelSat47.25
TdelSat48.118
TdelSat49.49
TdelSat50.101
TdelSat51.46
TdelSat52.88
TdelSat53.48
TdelSat54.189
TdelSat55.51
TdelSat56.102
TdelSat57.271
TdelSat58.84
TdelSat59.63
TdelSat60.99
TdelSat61.315
TdelSat62.53
TdelSat63.51
TdelSat64.39
TdelSat65.71
TdelSat66.64
TdelSat67.83
TdelSat68.160
TdelSat69.52
TdelSat70.86
TdelSat71.102
TdelSat72.85
TdelSat73.372
Percent of the genome
Kimura substitution level

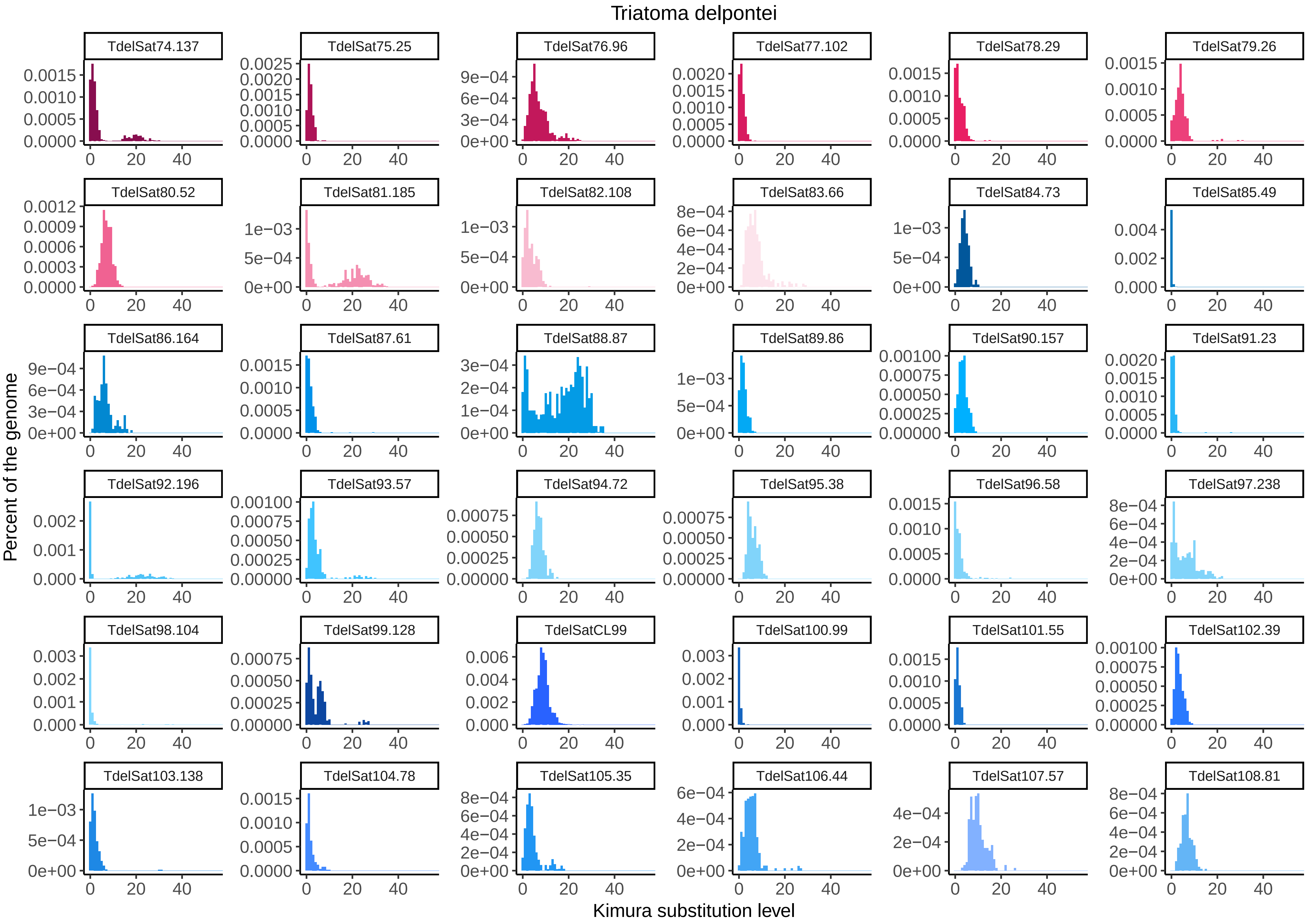

Triatoma delpontei
TdelSat74.137
TdelSat75.25
TdelSat76.96
TdelSat77.102
TdelSat78.29
TdelSat79.26
TdelSat80.52
TdelSat81.185
TdelSat82.108
TdelSat83.66
TdelSat84.73
TdelSat85.49
TdelSat86.164
TdelSat87.61
TdelSat88.87
TdelSat89.86
TdelSat90.157
TdelSat91.23
TdelSat92.196
TdelSat93.57
TdelSat94.72
TdelSat95.38
TdelSat96.58
TdelSat97.238
TdelSat98.104
TdelSat99.128
TdelSatCL99
TdelSat100.99
TdelSat101.55
TdelSat102.39
TdelSat103.138
TdelSat104.78
TdelSat105.35
TdelSat106.44
TdelSat107.57
TdelSat108.81
Percent of the genome
Kimura substitution level

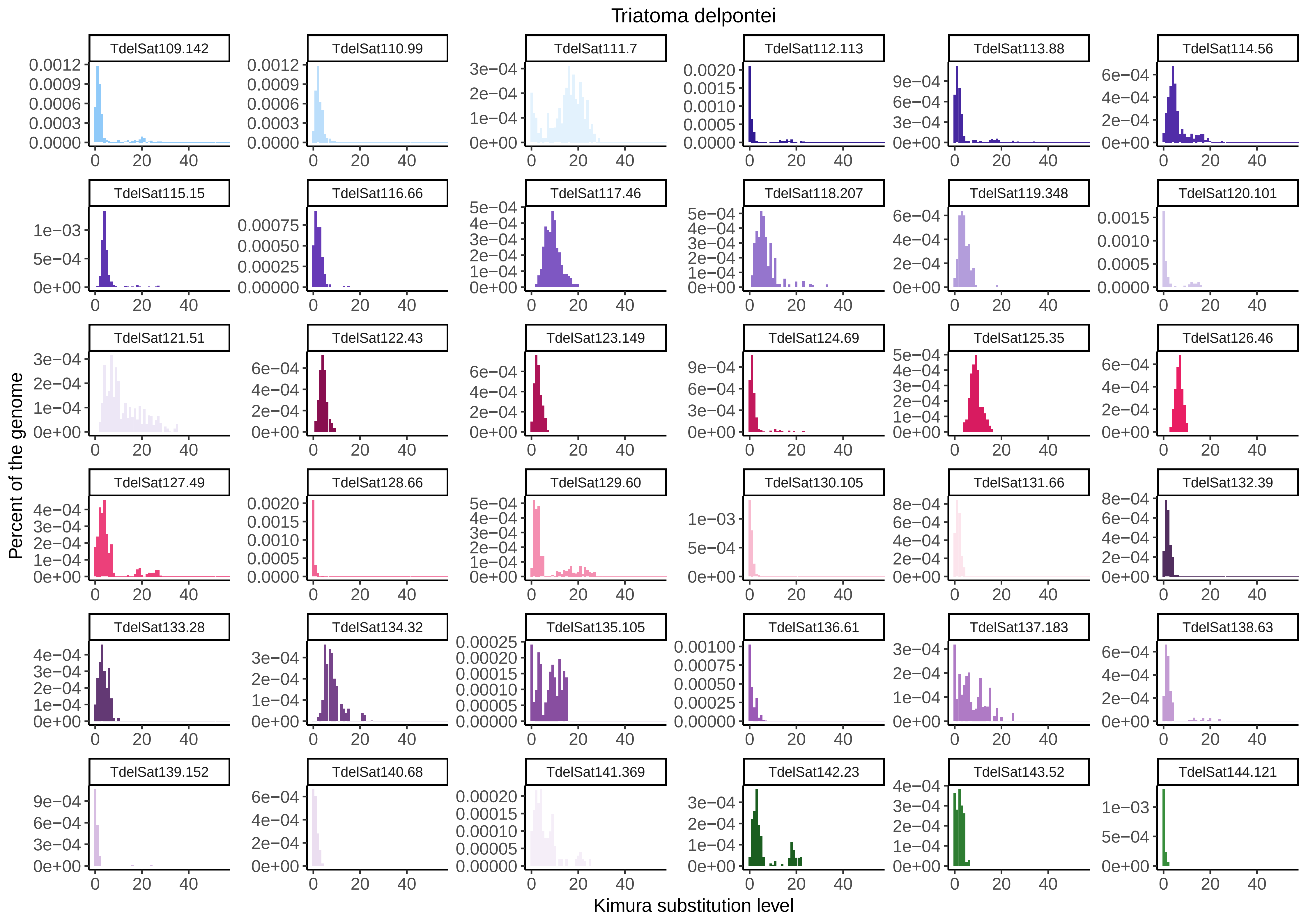

Triatoma delpontei
TdelSat109.142
TdelSat110.99
TdelSat111.7
TdelSat112.113
TdelSat113.88
TdelSat114.56
TdelSat115.15
TdelSat116.66
TdelSat117.46
TdelSat118.207
TdelSat119.348
TdelSat120.101
TdelSat121.51
TdelSat122.43
TdelSat123.149
TdelSat124.69
TdelSat125.35
TdelSat126.46
TdelSat127.49
TdelSat128.66
TdelSat129.60
TdelSat130.105
TdelSat131.66
TdelSat132.39
TdelSat133.28
TdelSat134.32
TdelSat135.105
TdelSat136.61
TdelSat137.183
TdelSat138.63
TdelSat139.152
TdelSat140.68
TdelSat141.369
TdelSat142.23
TdelSat143.52
TdelSat144.121
Percent of the genome
Kimura substitution level

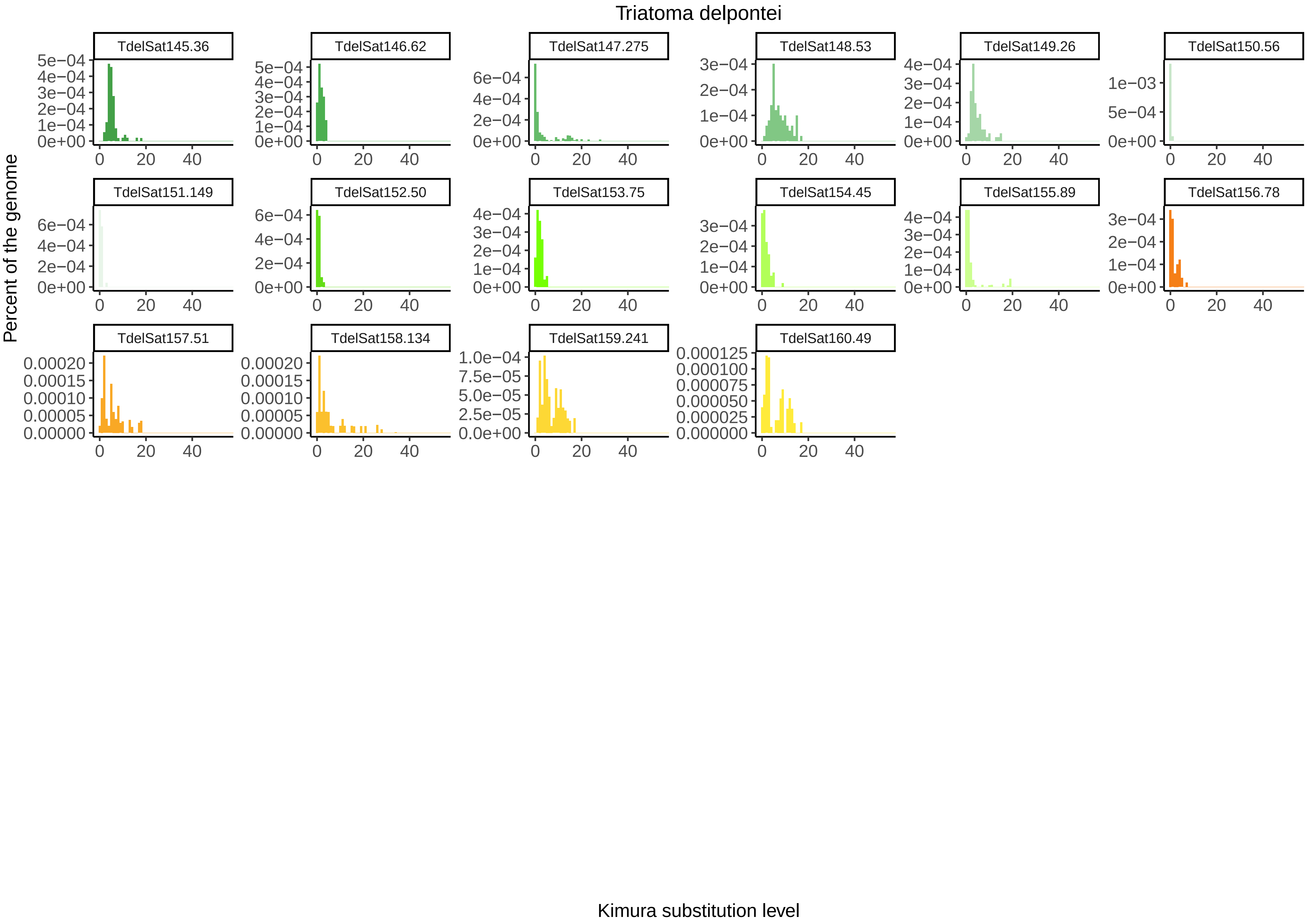

Triatoma delpontei
TdelSat145.36
TdelSat146.62
TdelSat147.275
TdelSat148.53
TdelSat149.26
TdelSat150.56
TdelSat151.149
TdelSat152.50
TdelSat153.75
TdelSat154.45
TdelSat155.89
TdelSat156.78
TdelSat157.51
TdelSat158.134
TdelSat159.241
TdelSat160.49
Percent of the genome
Kimura substitution level
