# Supplementary material for: Making the Genome Huge: The Case of Triatoma delpontei, a Triatominae Species with More than 50% of Its Genome Full of Satellite DNA
Source: Genes (Basel). 2023 Jan 31;14(2):371. doi: 10.3390/genes14020371 (PMC9957312; doi:10.3390/genes14020371)
Supplement: Supplementary file 1 [file genes-14-00371-s001.zip › Figure S4 T infestans Andean landscapes.pdf]

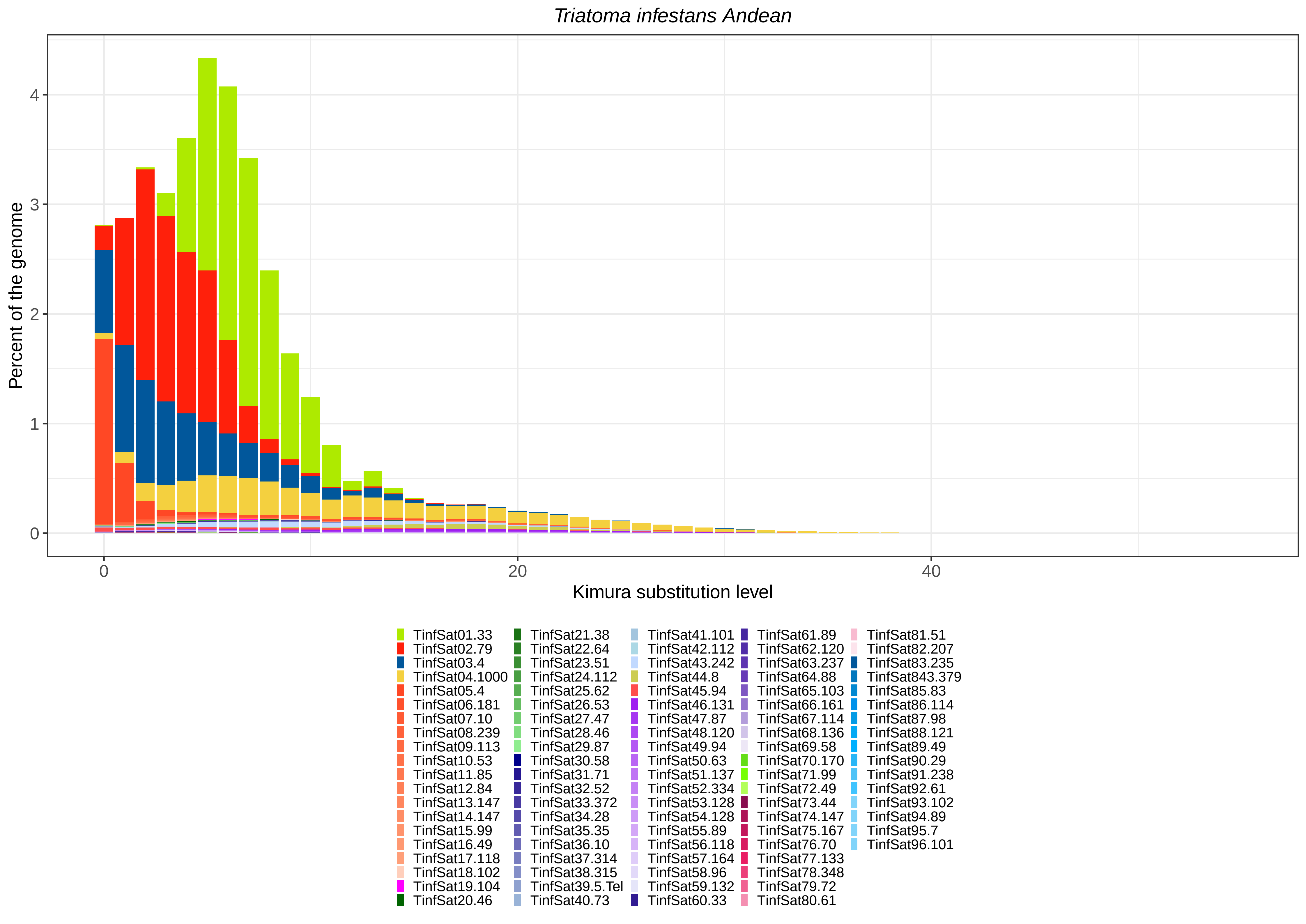

Triatoma infestans Andean
Percent of the genome
0
1
2
3
4
0
20
40
Kimura substitution level
TinfSat01.33
TinfSat02.79
TinfSat03.4
TinfSat04.1000
TinfSat05.4
TinfSat06.181
TinfSat07.10
TinfSat08.239
TinfSat09.113
TinfSat10.53
TinfSat11.85
TinfSat12.84
TinfSat13.147
TinfSat14.147
TinfSat15.99
TinfSat16.49
TinfSat17.118
TinfSat18.102
TinfSat19.104
TinfSat20.46
TinfSat21.38
TinfSat22.64
TinfSat23.51
TinfSat24.112
TinfSat25.62
TinfSat26.53
TinfSat27.47
TinfSat28.46
TinfSat29.87
TinfSat30.58
TinfSat31.71
TinfSat32.52
TinfSat33.372
TinfSat34.28
TinfSat35.35
TinfSat36.10
TinfSat37.314
TinfSat38.315
TinfSat39.5.Tel
TinfSat40.73
TinfSat41.101
TinfSat42.112
TinfSat43.242
TinfSat44.8
TinfSat45.94
TinfSat46.131
TinfSat47.87
TinfSat48.120
TinfSat49.94
TinfSat50.63
TinfSat51.137
TinfSat52.334
TinfSat53.128
TinfSat54.128
TinfSat55.89
TinfSat56.118
TinfSat57.164
TinfSat58.96
TinfSat59.132
TinfSat60.33
TinfSat61.89
TinfSat62.120
TinfSat63.237
TinfSat64.88
TinfSat65.103
TinfSat66.161
TinfSat67.114
TinfSat68.136
TinfSat69.58
TinfSat70.170
TinfSat71.99
TinfSat72.49
TinfSat73.44
TinfSat74.147
TinfSat75.167
TinfSat76.70
TinfSat77.133
TinfSat78.348
TinfSat79.72
TinfSat80.61
TinfSat81.51
TinfSat82.207
TinfSat83.235
TinfSat843.379
TinfSat85.83
TinfSat86.114
TinfSat87.98
TinfSat88.121
TinfSat89.49
TinfSat90.29
TinfSat91.238
TinfSat92.61
TinfSat93.102
TinfSat94.89
TinfSat95.7
TinfSat96.101

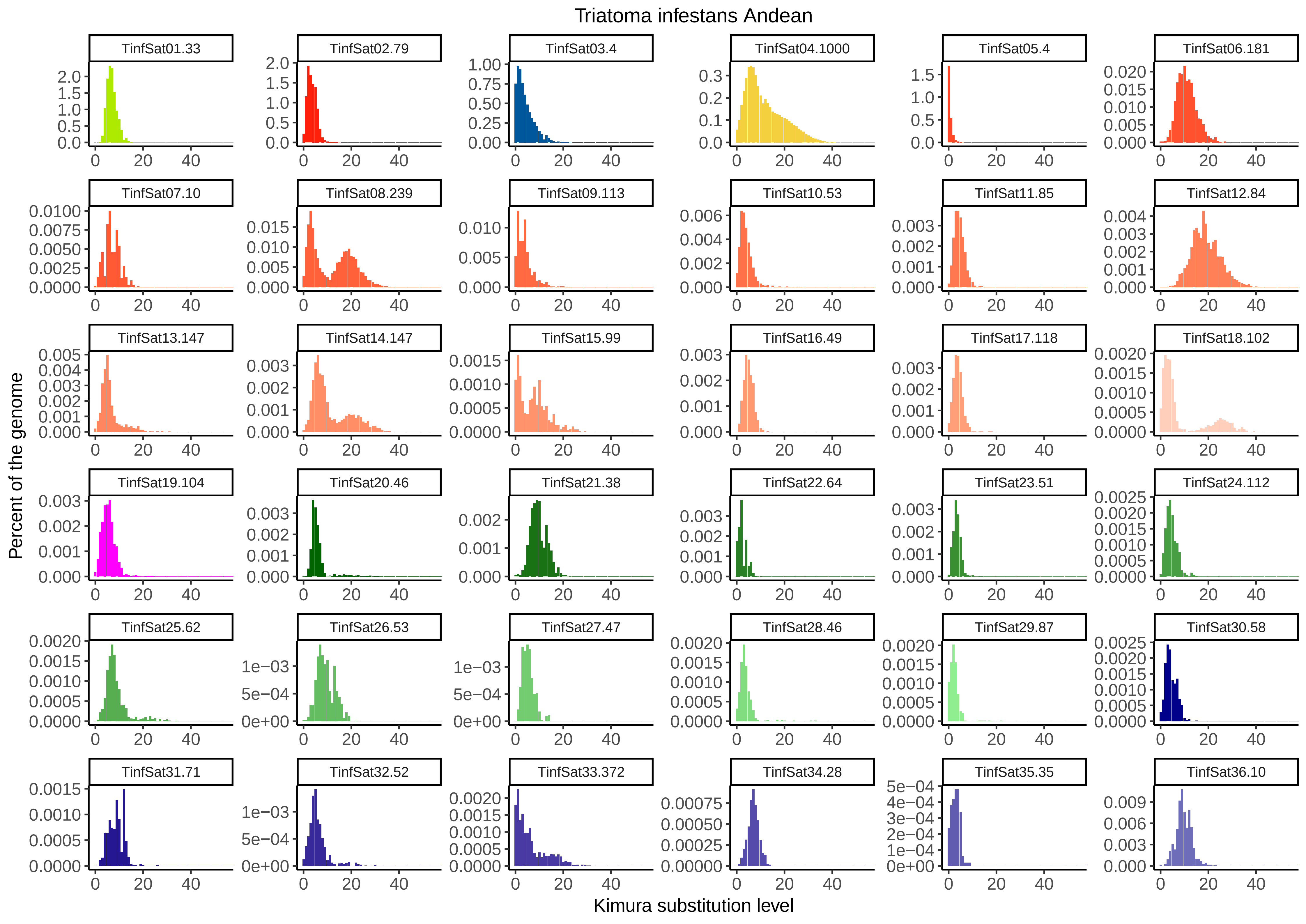

Triatoma infestans Andean
TinfSat01.33
TinfSat02.79
TinfSat03.4
TinfSat04.1000
TinfSat05.4
TinfSat06.181
TinfSat07.10
TinfSat08.239
TinfSat09.113
TinfSat10.53
TinfSat11.85
TinfSat12.84
TinfSat13.147
TinfSat14.147
TinfSat15.99
TinfSat16.49
TinfSat17.118
TinfSat18.102
TinfSat19.104
TinfSat20.46
TinfSat21.38
TinfSat22.64
TinfSat23.51
TinfSat24.112
TinfSat25.62
TinfSat26.53
TinfSat27.47
TinfSat28.46
TinfSat29.87
TinfSat30.58
TinfSat31.71
TinfSat32.52
TinfSat33.372
TinfSat34.28
TinfSat35.35
TinfSat36.10
Percent of the genome
Kimura substitution level

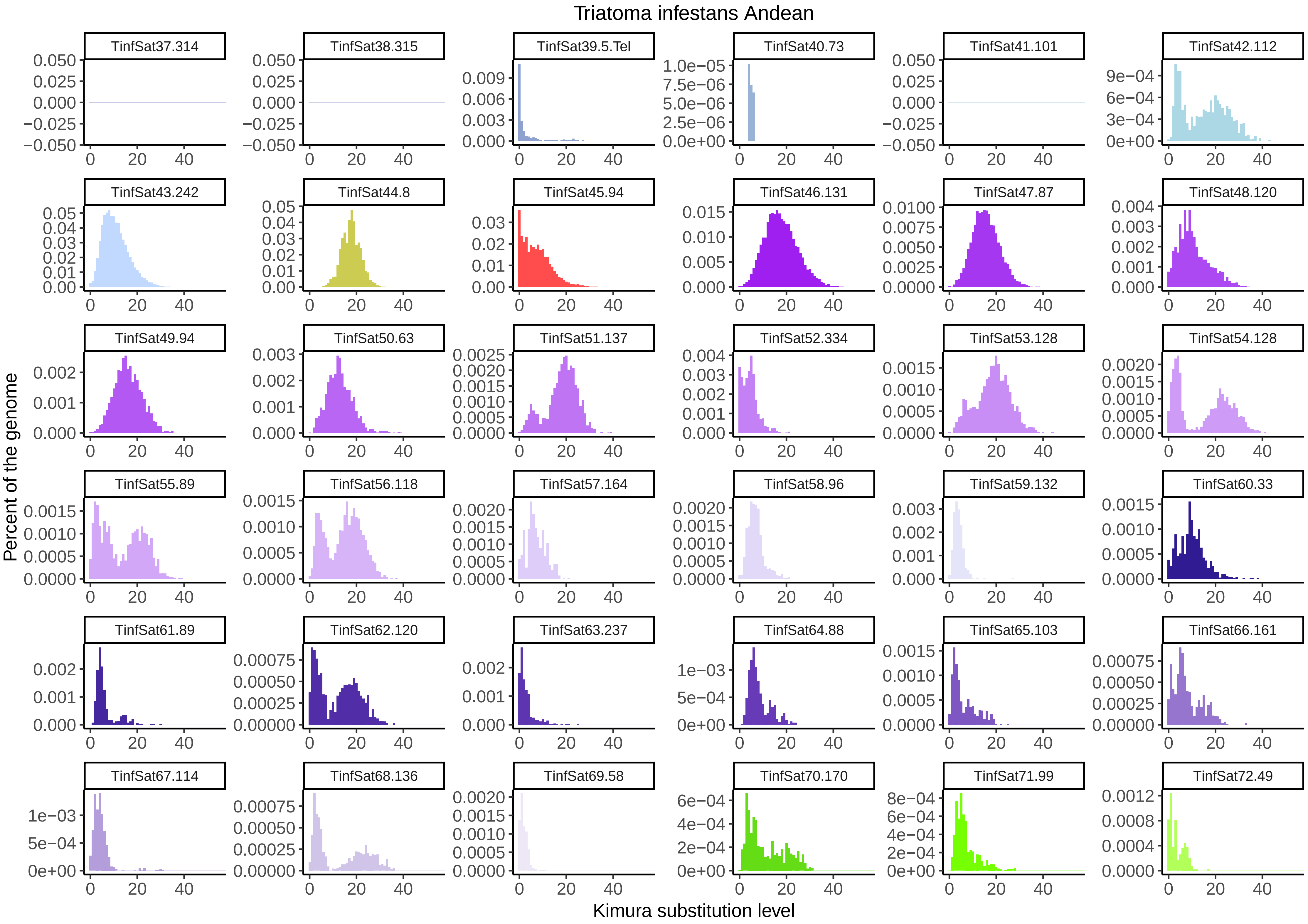
Triatoma infestans Andean
TinfSat37.314
TinfSat38.315
TinfSat39.5.Tel
TinfSat40.73
TinfSat41.101
TinfSat42.112
TinfSat43.242
TinfSat44.8
TinfSat45.94
TinfSat46.131
TinfSat47.87
TinfSat48.120
TinfSat49.94
TinfSat50.63
TinfSat51.137
TinfSat52.334
TinfSat53.128
TinfSat54.128
TinfSat55.89
TinfSat56.118
TinfSat57.164
TinfSat58.96
TinfSat59.132
TinfSat60.33
TinfSat61.89
TinfSat62.120
TinfSat63.237
TinfSat64.88
TinfSat65.103
TinfSat66.161
TinfSat67.114
TinfSat68.136
TinfSat69.58
TinfSat70.170
TinfSat71.99
TinfSat72.49
Percent of the genome
Kimura substitution level

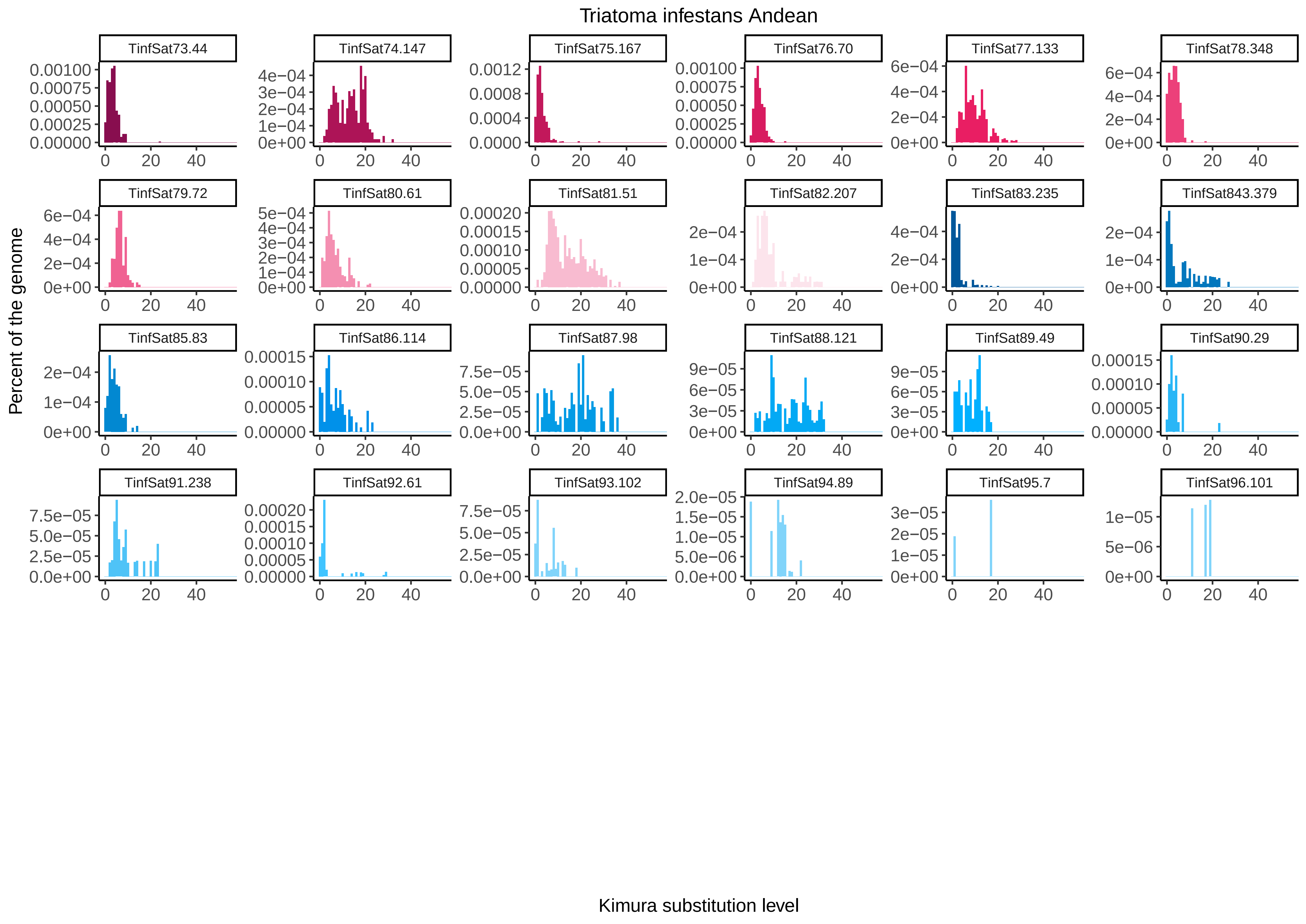
Triatoma infestans Andean
Percent of the genome
Kimura substitution level
TinfSat73.44
TinfSat74.147
TinfSat75.167
TinfSat76.70
TinfSat77.133
TinfSat78.348
TinfSat79.72
TinfSat80.61
TinfSat81.51
TinfSat82.207
TinfSat83.235
TinfSat843.379
TinfSat85.83
TinfSat86.114
TinfSat87.98
TinfSat88.121
TinfSat89.49
TinfSat90.29
TinfSat91.238
TinfSat92.61
TinfSat93.102
TinfSat94.89
TinfSat95.7
TinfSat96.101
