# Supplementary material for: Making the Genome Huge: The Case of Triatoma delpontei, a Triatominae Species with More than 50% of Its Genome Full of Satellite DNA
Source: Genes (Basel). 2023 Jan 31;14(2):371. doi: 10.3390/genes14020371 (PMC9957312; doi:10.3390/genes14020371)
Supplement: Supplementary file 1 [file genes-14-00371-s001.zip › Figure S6 Tdelpontei_Profiler.pdf]

Figure S6. RepeatProfiler results for the analyses of all satDNAs found in *T. delpontei* over three genomes: *T. delpontei*, Andean and non-Andean *T. infestans* lineages

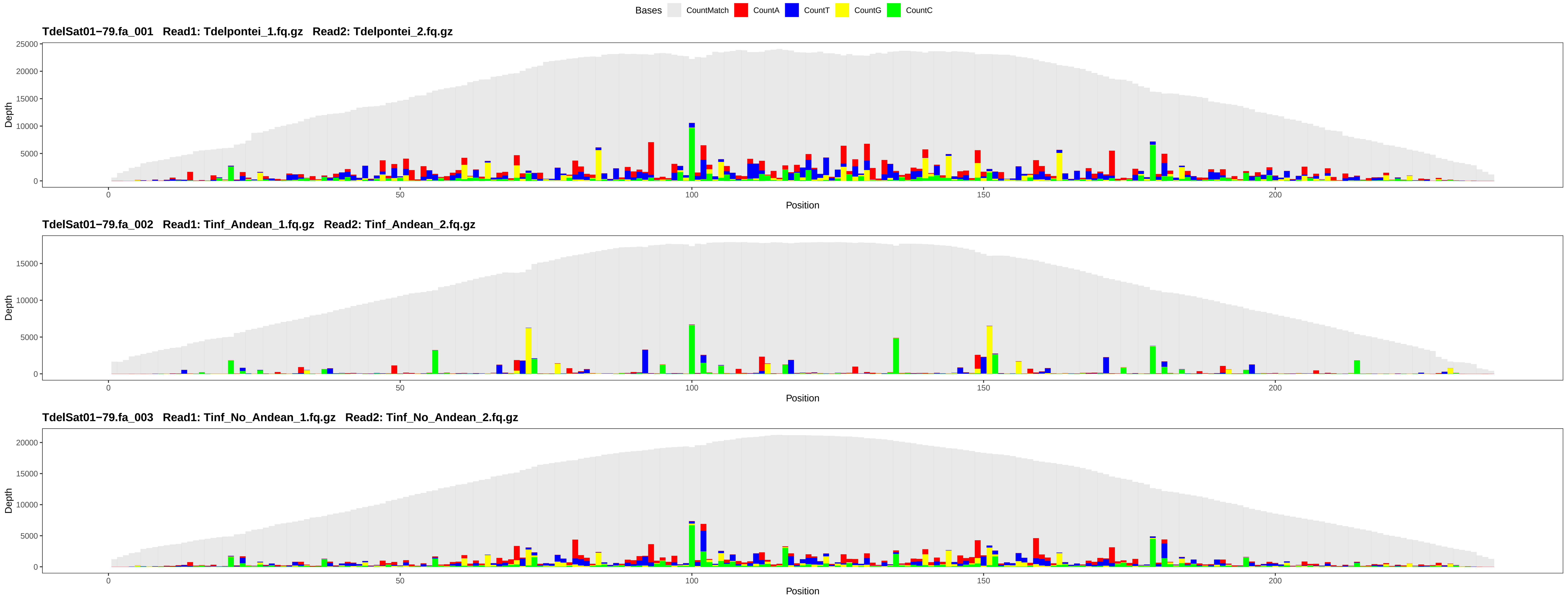
Bases
CountMatch
CountA
CountT
CountG
CountC
TdelSat01−79.fa_001 Read1: Tdelpontei_1.fq.gz Read2: Tdelpontei_2.fq.gz
Depth
Position
TdelSat01−79.fa_002 Read1: Tinf_Andean_1.fq.gz Read2: Tinf_Andean_2.fq.gz
Depth
Position
TdelSat01−79.fa_003 Read1: Tinf_No_Andean_1.fq.gz Read2: Tinf_No_Andean_2.fq.gz
Depth
Position

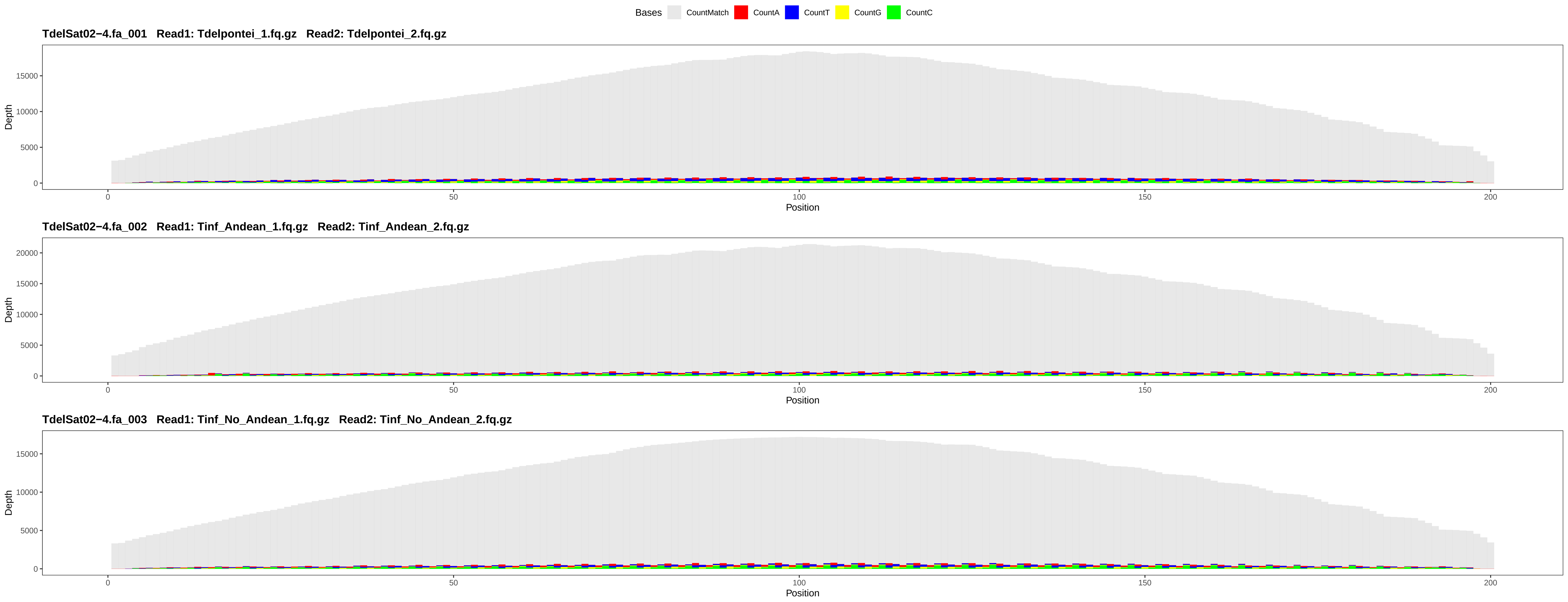
Bases CountMatch CountA CountT CountG CountC
TdelSat02−4.fa_001 Read1: Tdelpontei_1.fq.gz Read2: Tdelpontei_2.fq.gz
Depth
15000
10000
5000
0
Position
0 50 100 150 200
TdelSat02−4.fa_002 Read1: Tinf_Andean_1.fq.gz Read2: Tinf_Andean_2.fq.gz
Depth
20000
15000
10000
5000
0
Position
0 50 100 150 200
TdelSat02−4.fa_003 Read1: Tinf_No_Andean_1.fq.gz Read2: Tinf_No_Andean_2.fq.gz
Depth
15000
10000
5000
0
Position
0 50 100 150 200

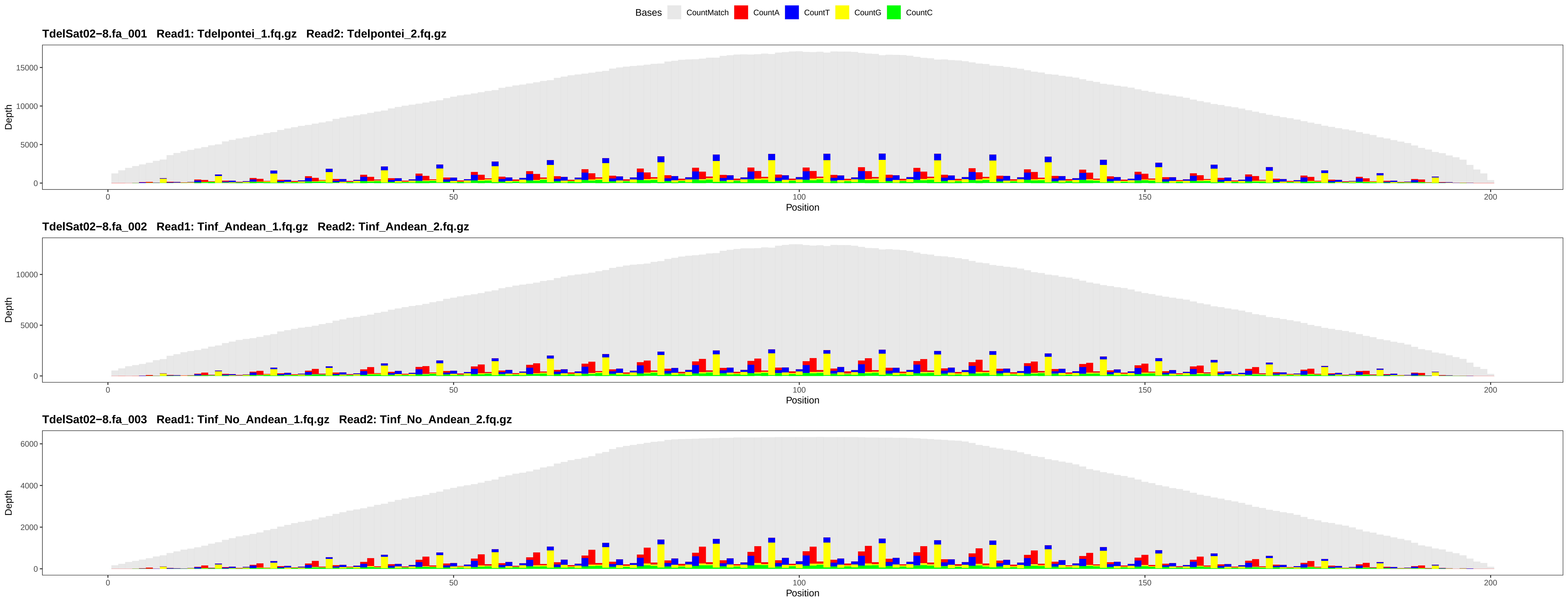
Bases CountMatch CountA CountT CountG CountC
TdelSat02−8.fa_001 Read1: Tdelpontei_1.fq.gz Read2: Tdelpontei_2.fq.gz
Depth
15000
10000
5000
0
0 50 100 150 200
Position
TdelSat02−8.fa_002 Read1: Tinf_Andean_1.fq.gz Read2: Tinf_Andean_2.fq.gz
Depth
10000
5000
0
0 50 100 150 200
Position
TdelSat02−8.fa_003 Read1: Tinf_No_Andean_1.fq.gz Read2: Tinf_No_Andean_2.fq.gz
Depth
6000
4000
2000
0
0 50 100 150 200
Position

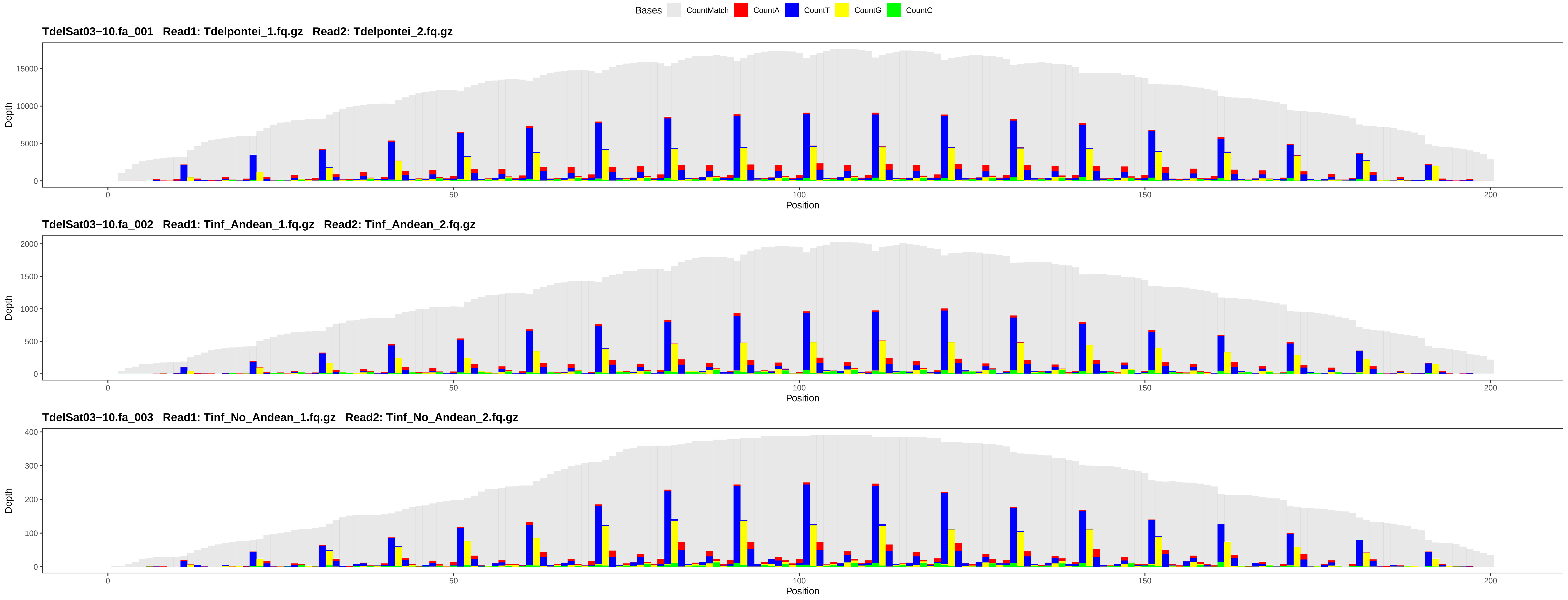
Bases
CountMatch
CountA
CountT
CountG
CountC
TdelSat03−10.fa_001   Read1: Tdelpontei_1.fq.gz   Read2: Tdelpontei_2.fq.gz
Depth
15000
10000
5000
0
Position
0
50
100
150
200
TdelSat03−10.fa_002   Read1: Tinf_Andean_1.fq.gz   Read2: Tinf_Andean_2.fq.gz
Depth
2000
1500
1000
500
0
Position
TdelSat03−10.fa_003   Read1: Tinf_No_Andean_1.fq.gz   Read2: Tinf_No_Andean_2.fq.gz
Depth
400
300
200
100
0
Position

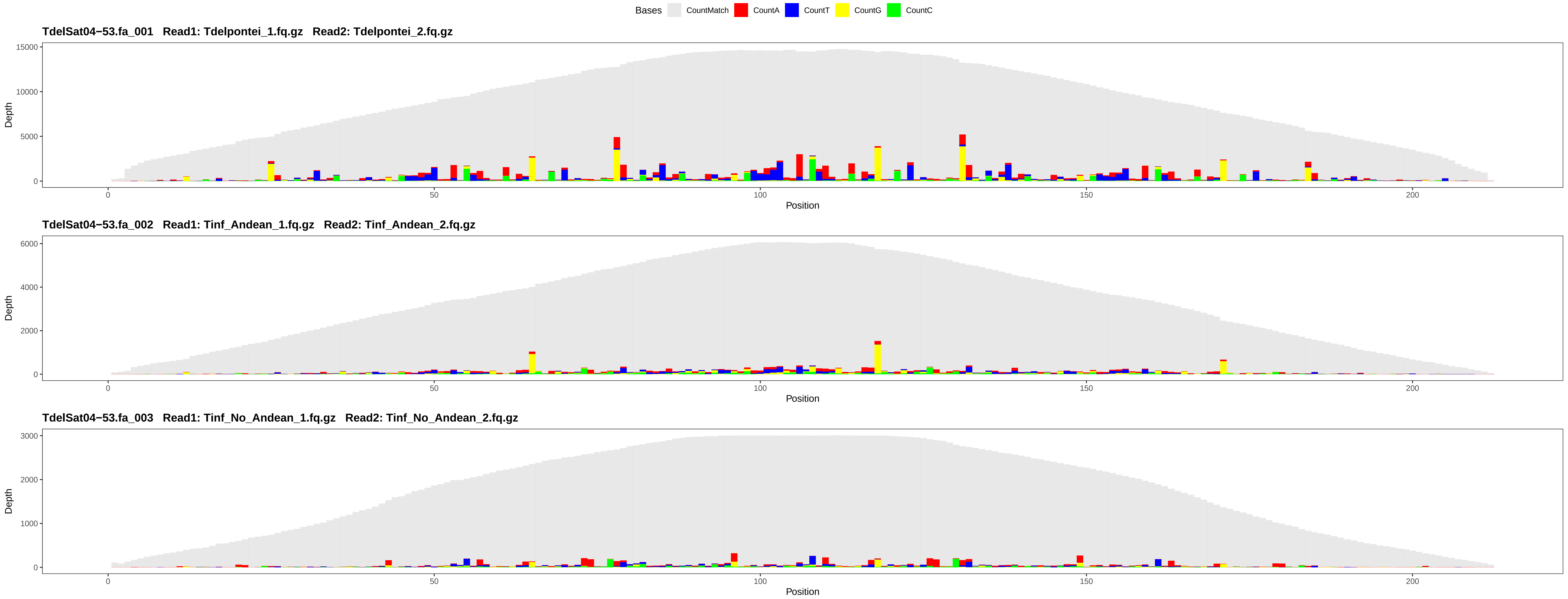
Bases
CountMatch
CountA
CountT
CountG
CountC
TdelSat04−53.fa_001 Read1: Tdelpontei_1.fq.gz Read2: Tdelpontei_2.fq.gz
15000
10000
5000
0
Depth
0
50
100
150
200
Position
TdelSat04−53.fa_002 Read1: Tinf_Andean_1.fq.gz Read2: Tinf_Andean_2.fq.gz
6000
4000
2000
0
Depth
0
50
100
150
200
Position
TdelSat04−53.fa_003 Read1: Tinf_No_Andean_1.fq.gz Read2: Tinf_No_Andean_2.fq.gz
3000
2000
1000
0
Depth
0
50
100
150
200
Position

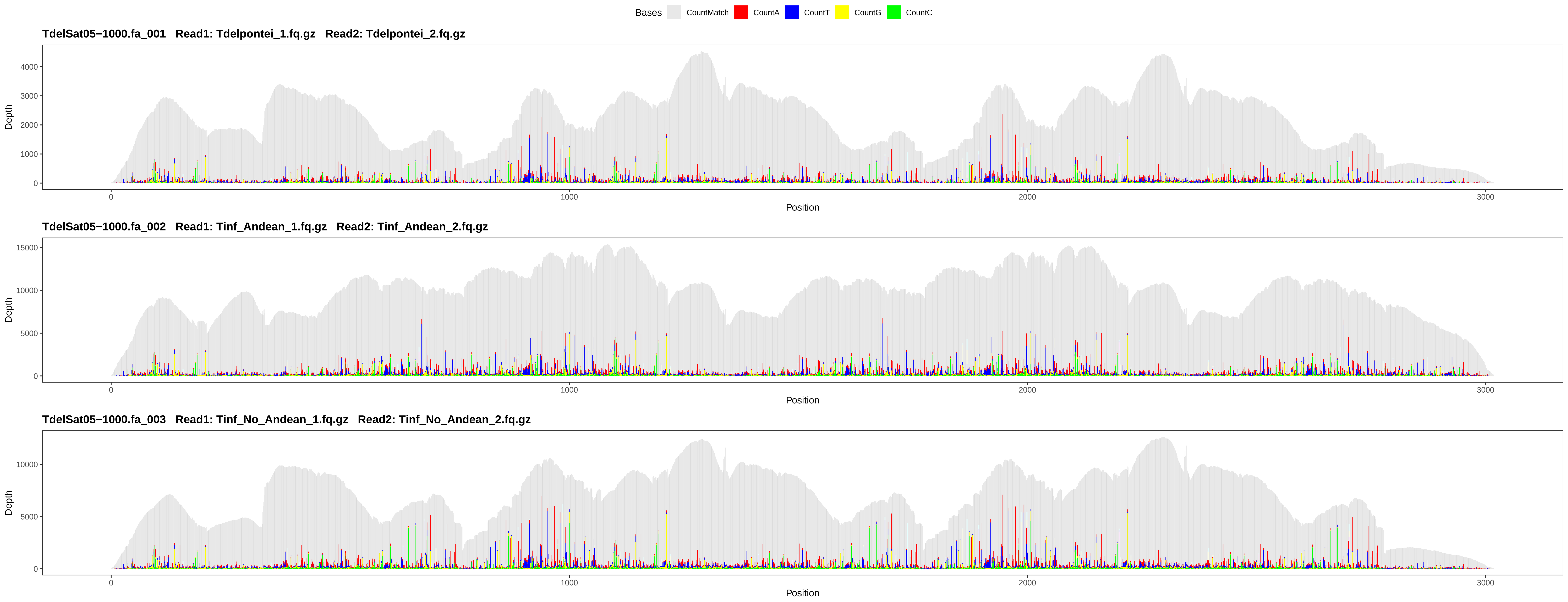
Bases
CountMatch
CountA
CountT
CountG
CountC
TdelSat05−1000.fa_001 Read1: Tdelpontei_1.fq.gz Read2: Tdelpontei_2.fq.gz
TdelSat05−1000.fa_002 Read1: Tinf_Andean_1.fq.gz Read2: Tinf_Andean_2.fq.gz
TdelSat05−1000.fa_003 Read1: Tinf_No_Andean_1.fq.gz Read2: Tinf_No_Andean_2.fq.gz
Depth
Position
0
1000
2000
3000
4000
5000
10000
15000

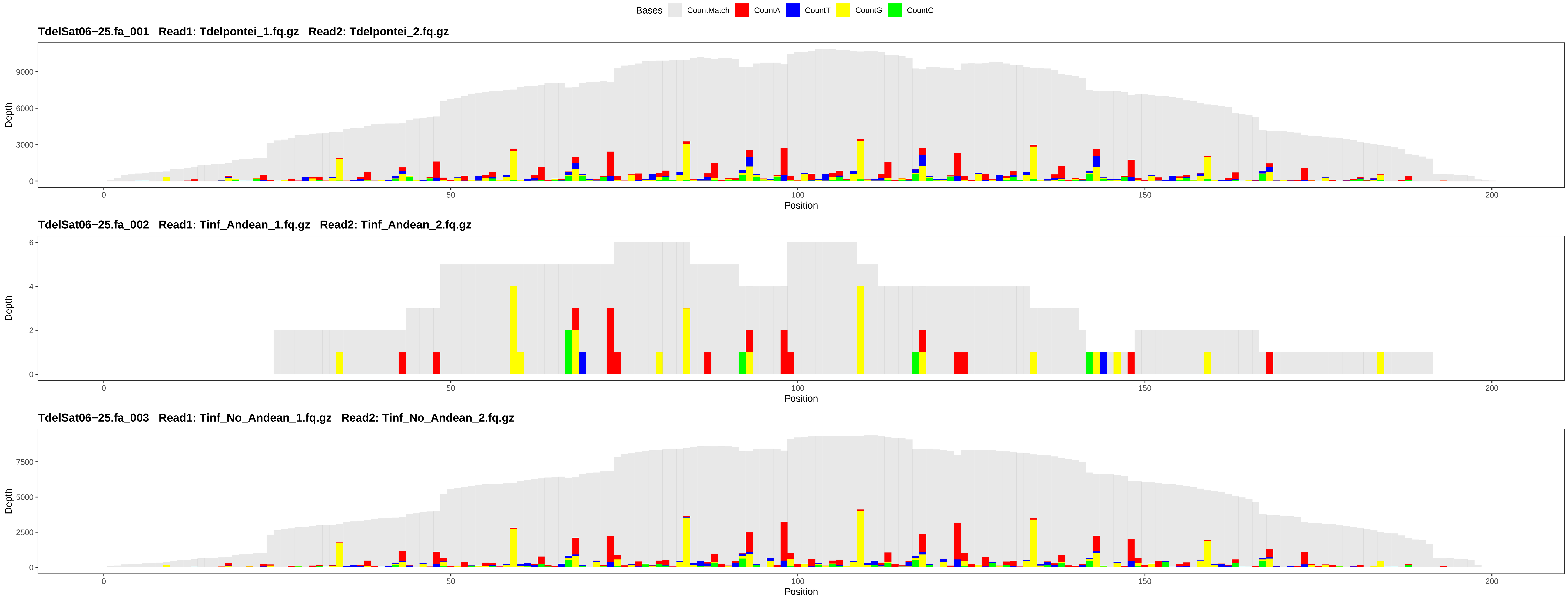
Bases CountMatch CountA CountT CountG CountC
TdelSat06−25.fa_001 Read1: Tdelpontei_1.fq.gz Read2: Tdelpontei_2.fq.gz
Depth
9000
6000
3000
0
0 50 100 150 200
Position
TdelSat06−25.fa_002 Read1: Tinf_Andean_1.fq.gz Read2: Tinf_Andean_2.fq.gz
Depth
6
4
2
0
0 50 100 150 200
Position
TdelSat06−25.fa_003 Read1: Tinf_No_Andean_1.fq.gz Read2: Tinf_No_Andean_2.fq.gz
Depth
7500
5000
2500
0
0 50 100 150 200
Position

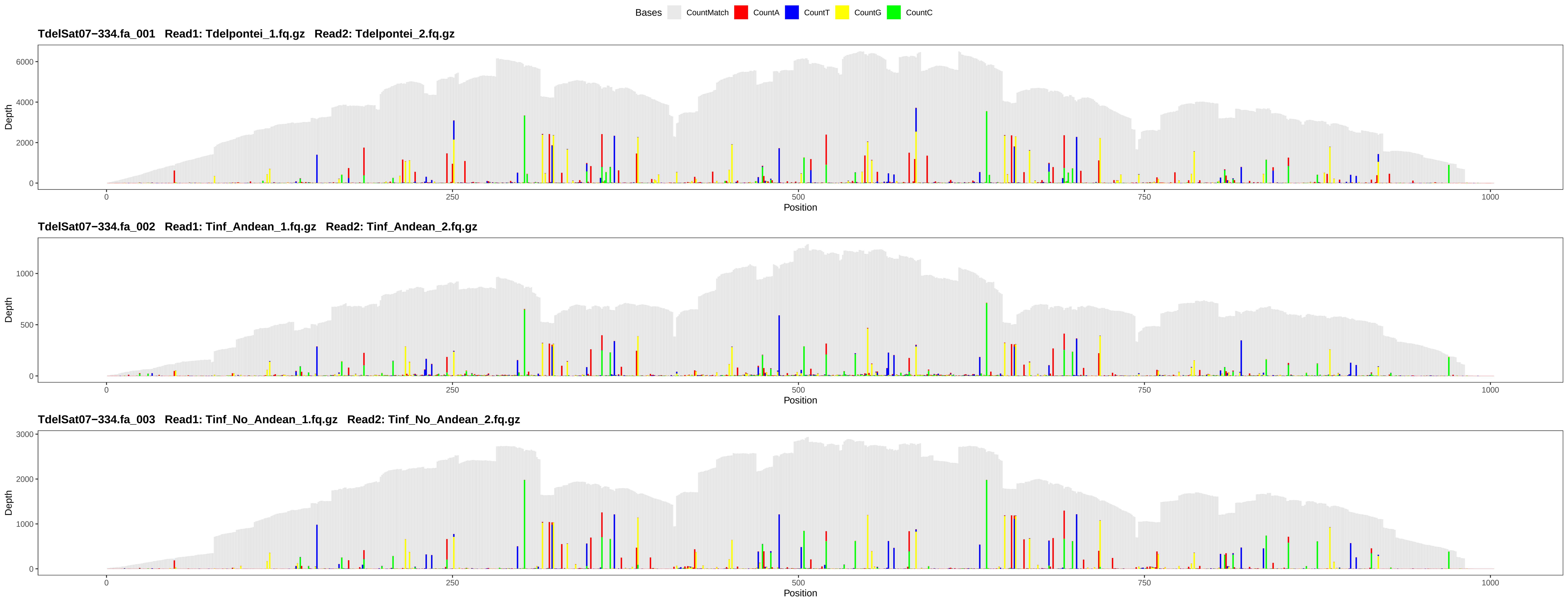
Bases
CountMatch
CountA
CountT
CountG
CountC
TdelSat07−334.fa_001 Read1: Tdelpontei_1.fq.gz Read2: Tdelpontei_2.fq.gz
Depth
6000
4000
2000
0
0
250
500
750
1000
Position
TdelSat07−334.fa_002 Read1: Tinf_Andean_1.fq.gz Read2: Tinf_Andean_2.fq.gz
Depth
1000
500
0
0
250
500
750
1000
Position
TdelSat07−334.fa_003 Read1: Tinf_No_Andean_1.fq.gz Read2: Tinf_No_Andean_2.fq.gz
Depth
3000
2000
1000
0
0
250
500
750
1000
Position

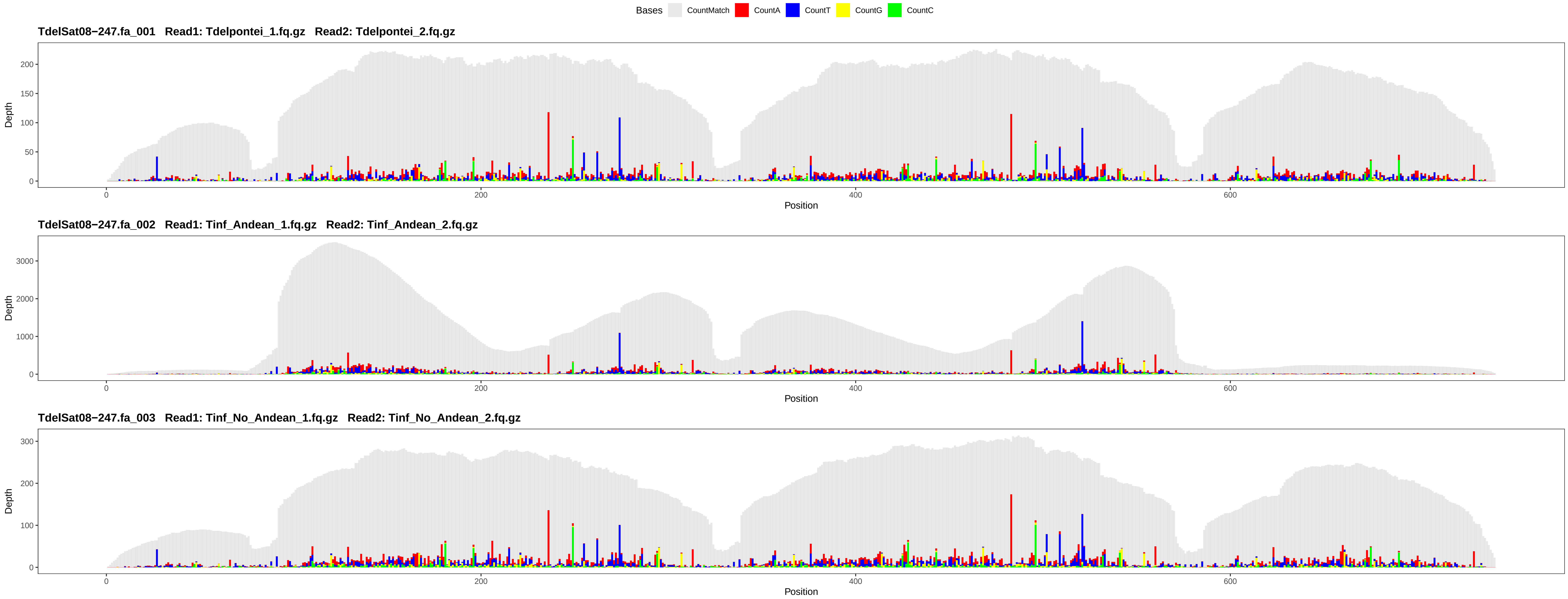
Bases CountMatch CountA CountT CountG CountC
TdelSat08−247.fa_001 Read1: Tdelpontei_1.fq.gz Read2: Tdelpontei_2.fq.gz
Depth
0
50
100
150
200
0
200
400
600
Position
TdelSat08−247.fa_002 Read1: Tinf_Andean_1.fq.gz Read2: Tinf_Andean_2.fq.gz
Depth
0
1000
2000
3000
0
200
400
600
Position
TdelSat08−247.fa_003 Read1: Tinf_No_Andean_1.fq.gz Read2: Tinf_No_Andean_2.fq.gz
Depth
0
100
200
300
0
200
400
600
Position

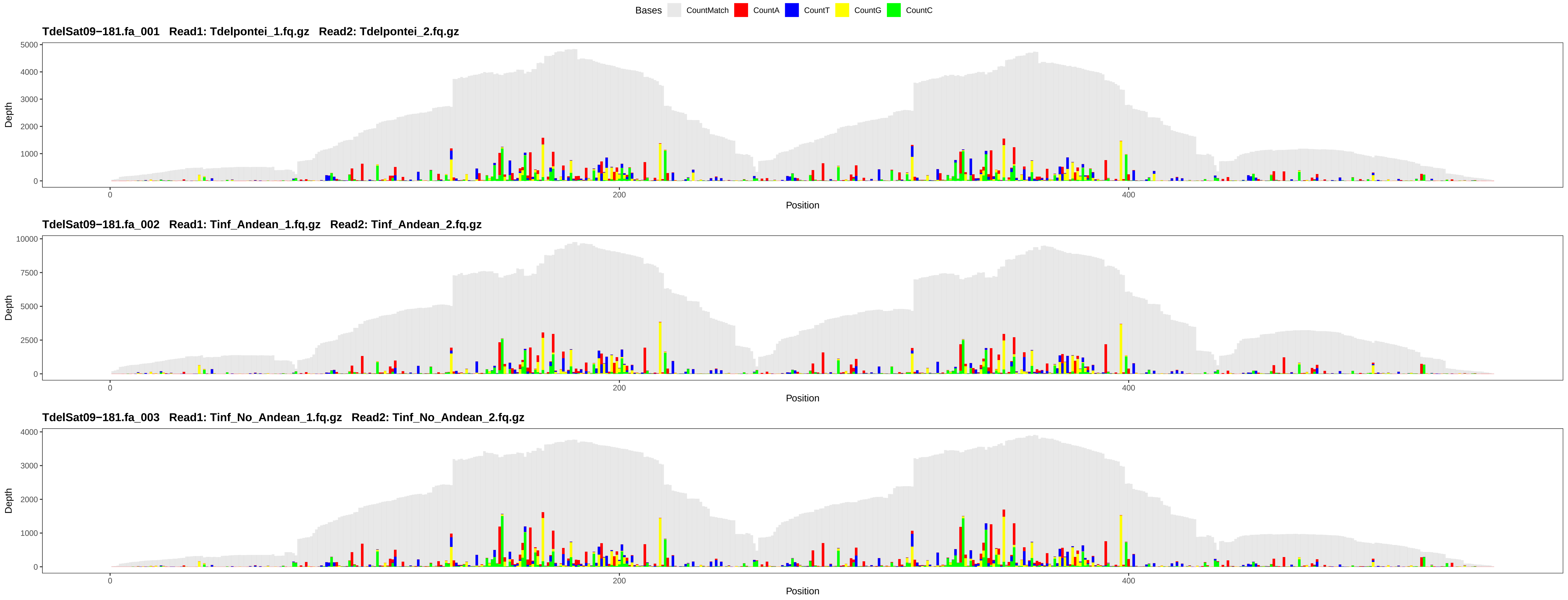
Bases
CountMatch
CountA
CountT
CountG
CountC
TdelSat09−181.fa_001 Read1: Tdelpontei_1.fq.gz Read2: Tdelpontei_2.fq.gz
Depth
5000
4000
3000
2000
1000
0
0
200
400
Position
TdelSat09−181.fa_002 Read1: Tinf_Andean_1.fq.gz Read2: Tinf_Andean_2.fq.gz
Depth
10000
7500
5000
2500
0
0
200
400
Position
TdelSat09−181.fa_003 Read1: Tinf_No_Andean_1.fq.gz Read2: Tinf_No_Andean_2.fq.gz
Depth
4000
3000
2000
1000
0
0
200
400
Position

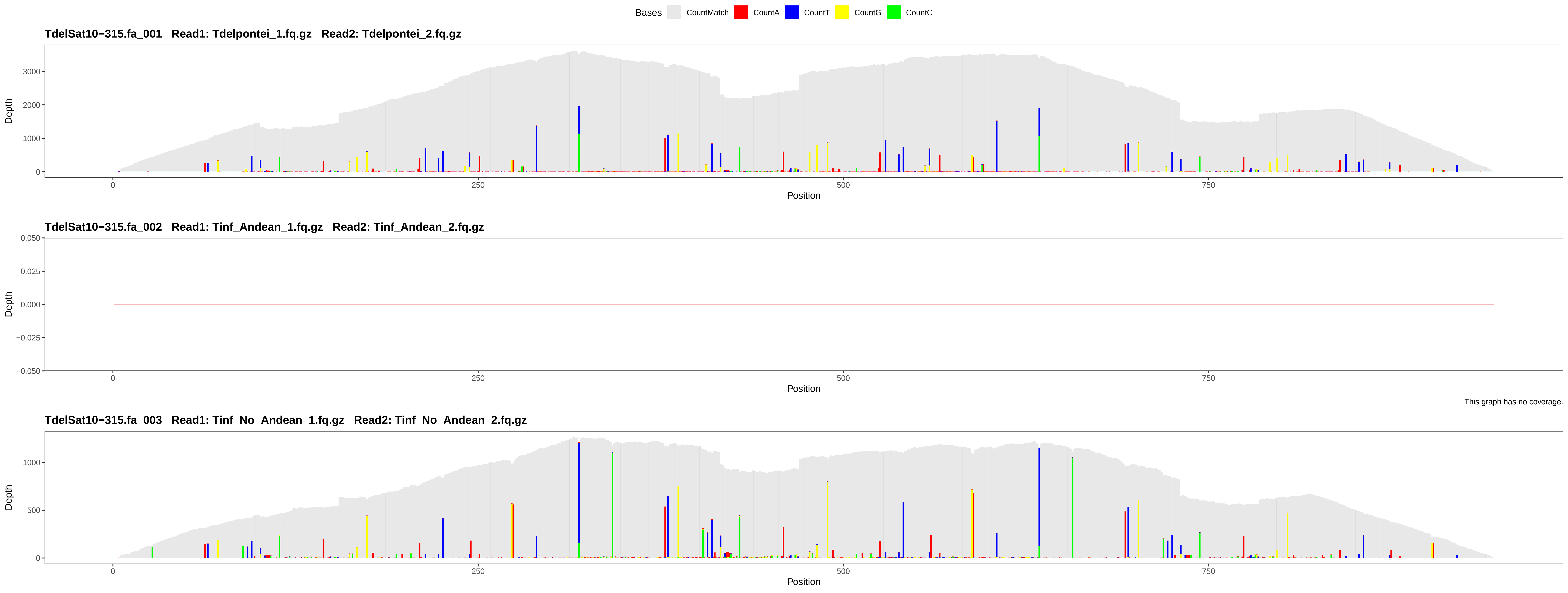
Bases CountMatch CountA CountT CountG CountC
TdelSat10−315.fa_001 Read1: Tdelpontei_1.fq.gz Read2: Tdelpontei_2.fq.gz
Depth
0
1000
2000
3000
0
250
500
750
Position
TdelSat10−315.fa_002 Read1: Tinf_Andean_1.fq.gz Read2: Tinf_Andean_2.fq.gz
Depth
0.050
0.025
0.000
−0.025
−0.050
0
250
500
750
Position
This graph has no coverage.
TdelSat10−315.fa_003 Read1: Tinf_No_Andean_1.fq.gz Read2: Tinf_No_Andean_2.fq.gz
Depth
0
500
1000
0
250
500
750
Position

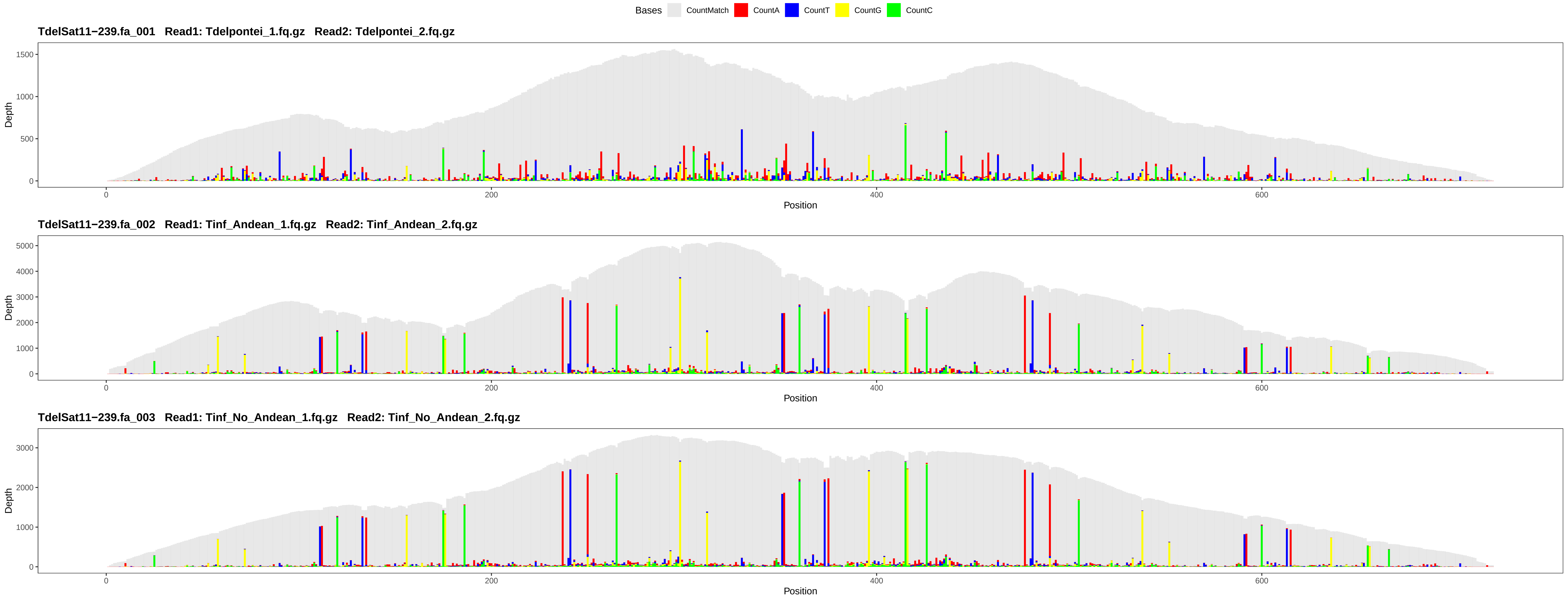
Bases
CountMatch
CountA
CountT
CountG
CountC
TdelSat11−239.fa_001 Read1: Tdelpontei_1.fq.gz Read2: Tdelpontei_2.fq.gz
Depth
0
500
1000
1500
Position
0
200
400
600
TdelSat11−239.fa_002 Read1: Tinf_Andean_1.fq.gz Read2: Tinf_Andean_2.fq.gz
Depth
0
1000
2000
3000
4000
5000
Position
0
200
400
600
TdelSat11−239.fa_003 Read1: Tinf_No_Andean_1.fq.gz Read2: Tinf_No_Andean_2.fq.gz
Depth
0
1000
2000
3000
Position
0
200
400
600

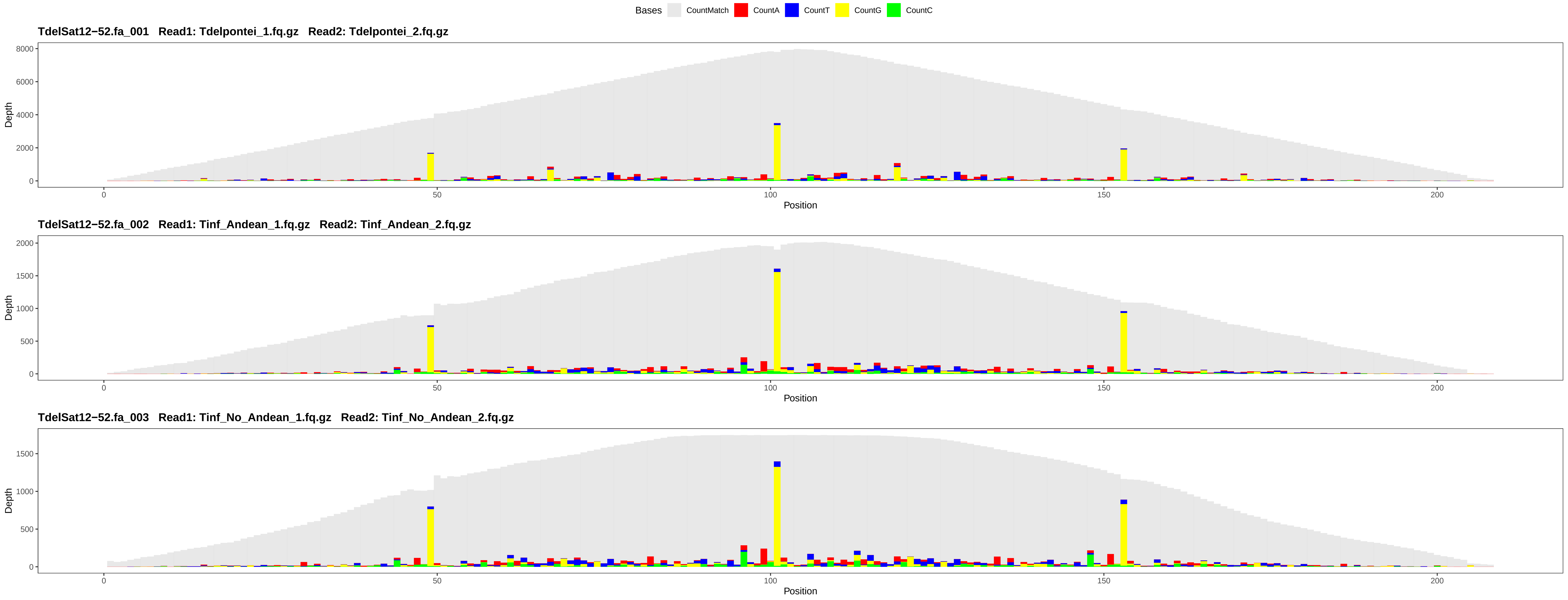
Bases CountMatch CountA CountT CountG CountC
TdelSat12−52.fa_001 Read1: Tdelpontei_1.fq.gz Read2: Tdelpontei_2.fq.gz
TdelSat12−52.fa_002 Read1: Tinf_Andean_1.fq.gz Read2: Tinf_Andean_2.fq.gz
TdelSat12−52.fa_003 Read1: Tinf_No_Andean_1.fq.gz Read2: Tinf_No_Andean_2.fq.gz
Depth
Position
0
50
100
150
200
8000
6000
4000
2000
1500
1000
500

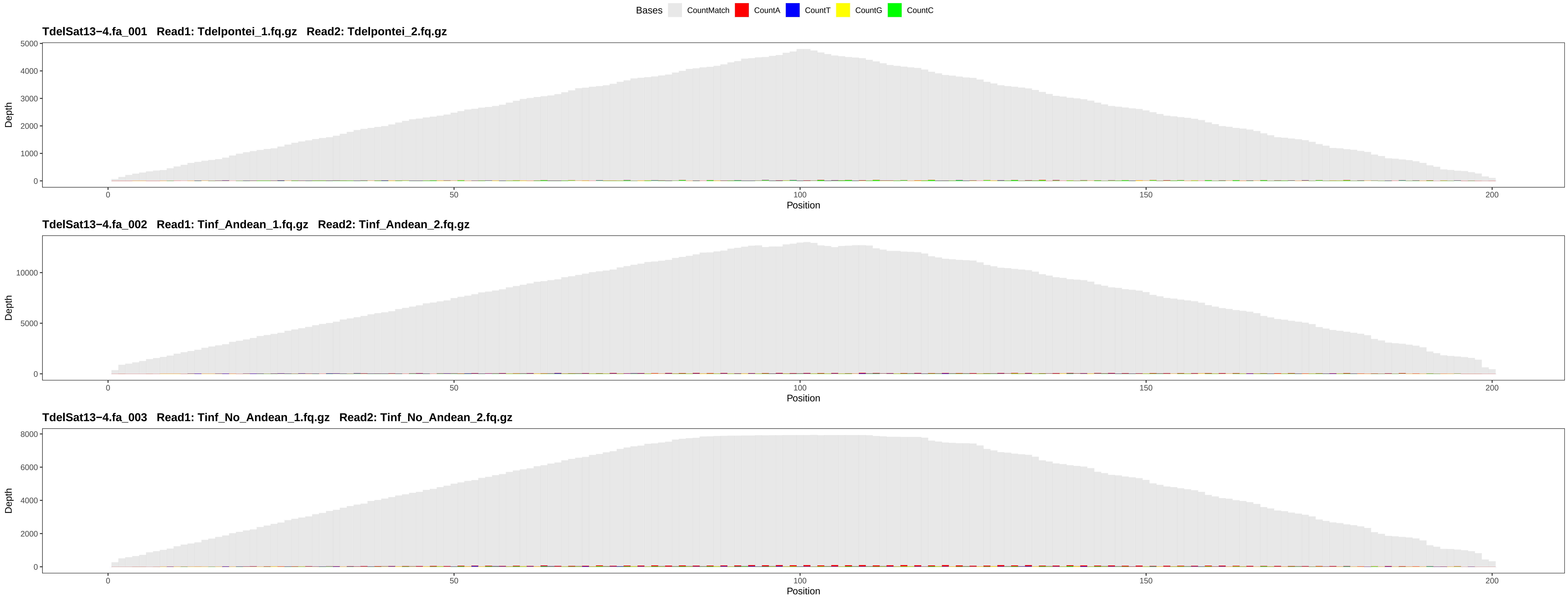
Bases
CountMatch
CountA
CountT
CountG
CountC
TdelSat13−4.fa_001 Read1: Tdelpontei_1.fq.gz Read2: Tdelpontei_2.fq.gz
Depth
Position
TdelSat13−4.fa_002 Read1: Tinf_Andean_1.fq.gz Read2: Tinf_Andean_2.fq.gz
Depth
Position
TdelSat13−4.fa_003 Read1: Tinf_No_Andean_1.fq.gz Read2: Tinf_No_Andean_2.fq.gz
Depth
Position

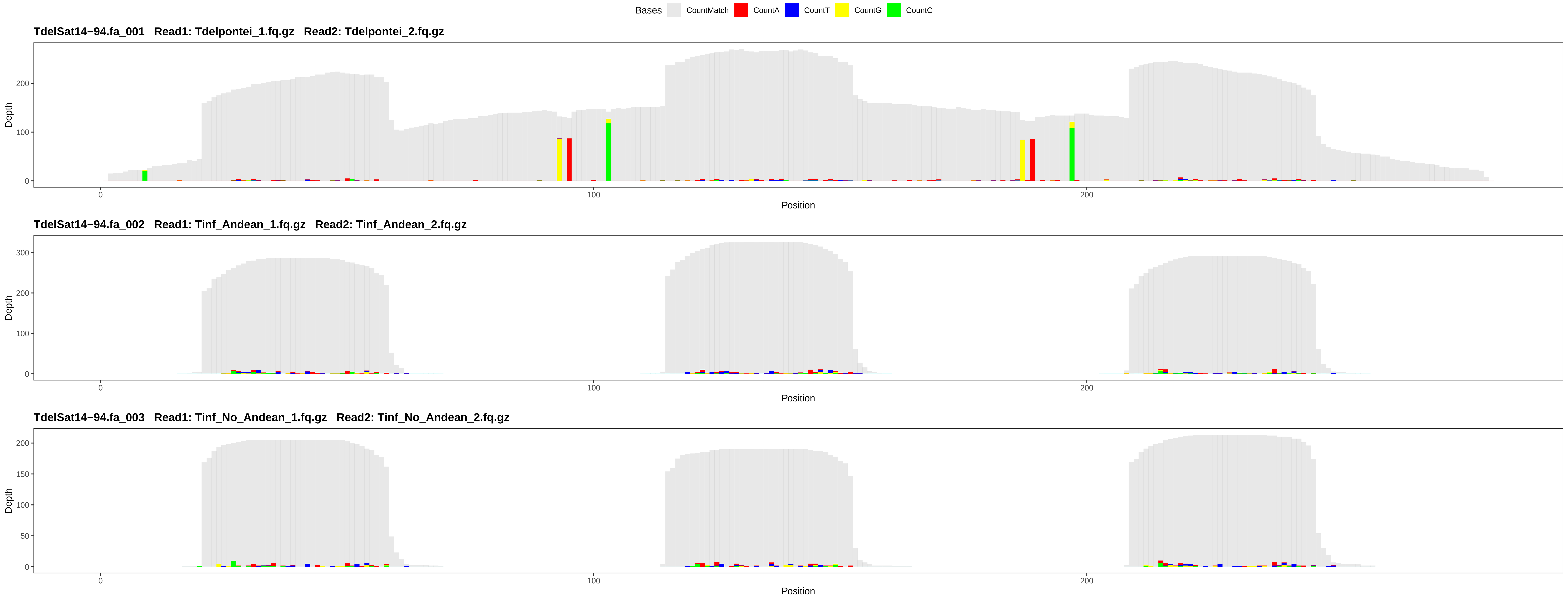

Bases
CountMatch
CountA
CountT
CountG
CountC
TdelSat14−94.fa_001 Read1: Tdelpontei_1.fq.gz Read2: Tdelpontei_2.fq.gz
Depth
0
100
200
Position
TdelSat14−94.fa_002 Read1: Tinf_Andean_1.fq.gz Read2: Tinf_Andean_2.fq.gz
Depth
300
TdelSat14−94.fa_003 Read1: Tinf_No_Andean_1.fq.gz Read2: Tinf_No_Andean_2.fq.gz
50
150

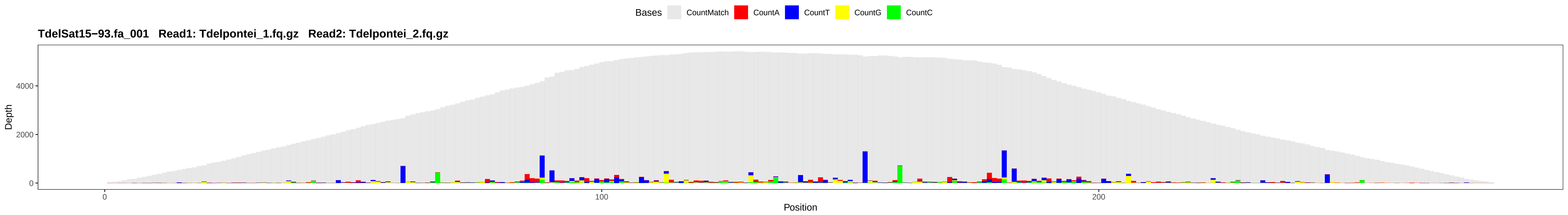
Bases
CountMatch
CountA
CountT
CountG
CountC
TdelSat15−93.fa_001 Read1: Tdelpontei_1.fq.gz Read2: Tdelpontei_2.fq.gz
Depth
4000
2000
0
0
100
200
Position

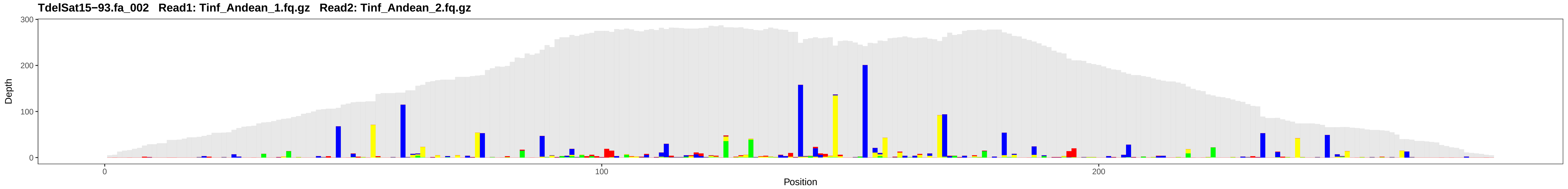
TdelSat15−93.fa_002 Read1: Tinf_Andean_1.fq.gz Read2: Tinf_Andean_2.fq.gz
Depth
300
200
100
0
0
100
200
Position

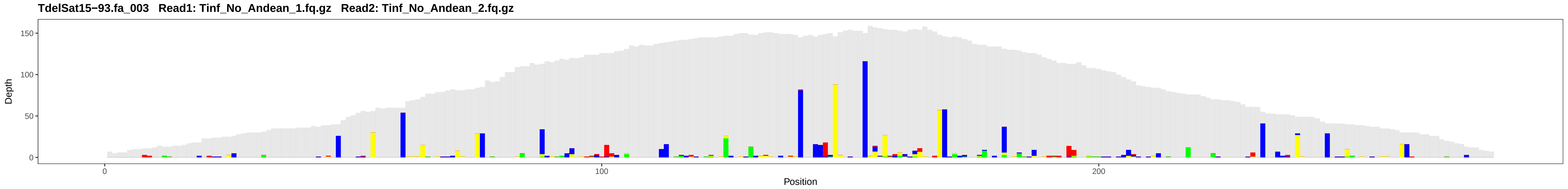
TdelSat15−93.fa_003 Read1: Tinf_No_Andean_1.fq.gz Read2: Tinf_No_Andean_2.fq.gz
Depth
150
100
50
0
0
100
200
Position

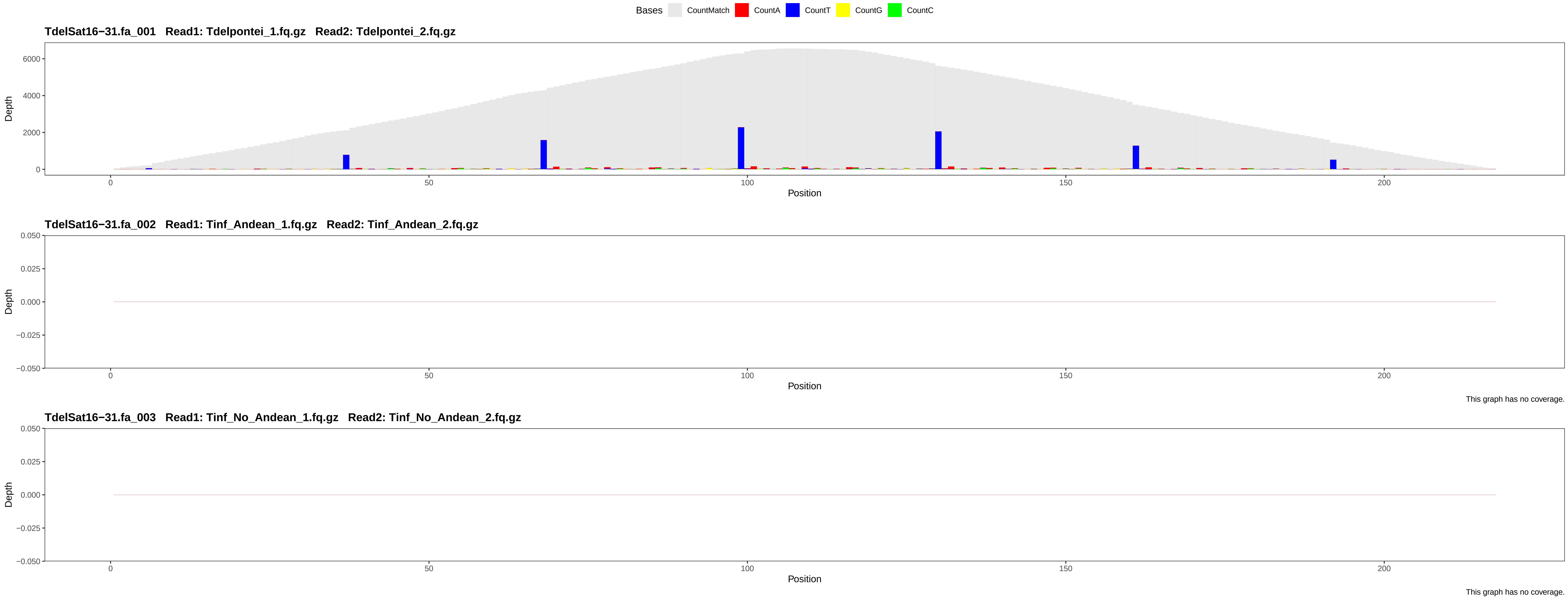
Bases CountMatch CountA CountT CountG CountC
TdelSat16−31.fa_001 Read1: Tdelpontei_1.fq.gz Read2: Tdelpontei_2.fq.gz
Depth
Position
TdelSat16−31.fa_002 Read1: Tinf_Andean_1.fq.gz Read2: Tinf_Andean_2.fq.gz
Depth
Position
This graph has no coverage.
TdelSat16−31.fa_003 Read1: Tinf_No_Andean_1.fq.gz Read2: Tinf_No_Andean_2.fq.gz
Depth
Position
This graph has no coverage.

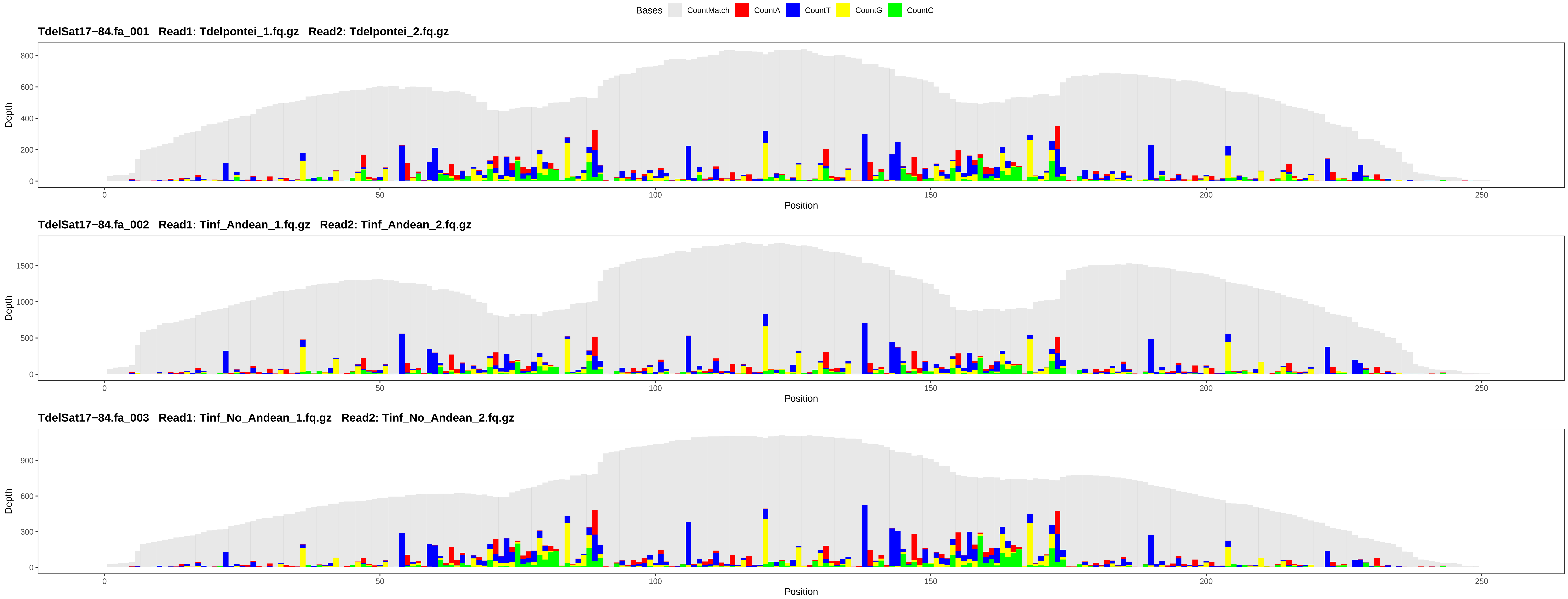
Bases CountMatch CountA CountT CountG CountC
TdelSat17−84.fa_001 Read1: Tdelpontei_1.fq.gz Read2: Tdelpontei_2.fq.gz
Depth
Position
TdelSat17−84.fa_002 Read1: Tinf_Andean_1.fq.gz Read2: Tinf_Andean_2.fq.gz
Depth
Position
TdelSat17−84.fa_003 Read1: Tinf_No_Andean_1.fq.gz Read2: Tinf_No_Andean_2.fq.gz
Depth
Position

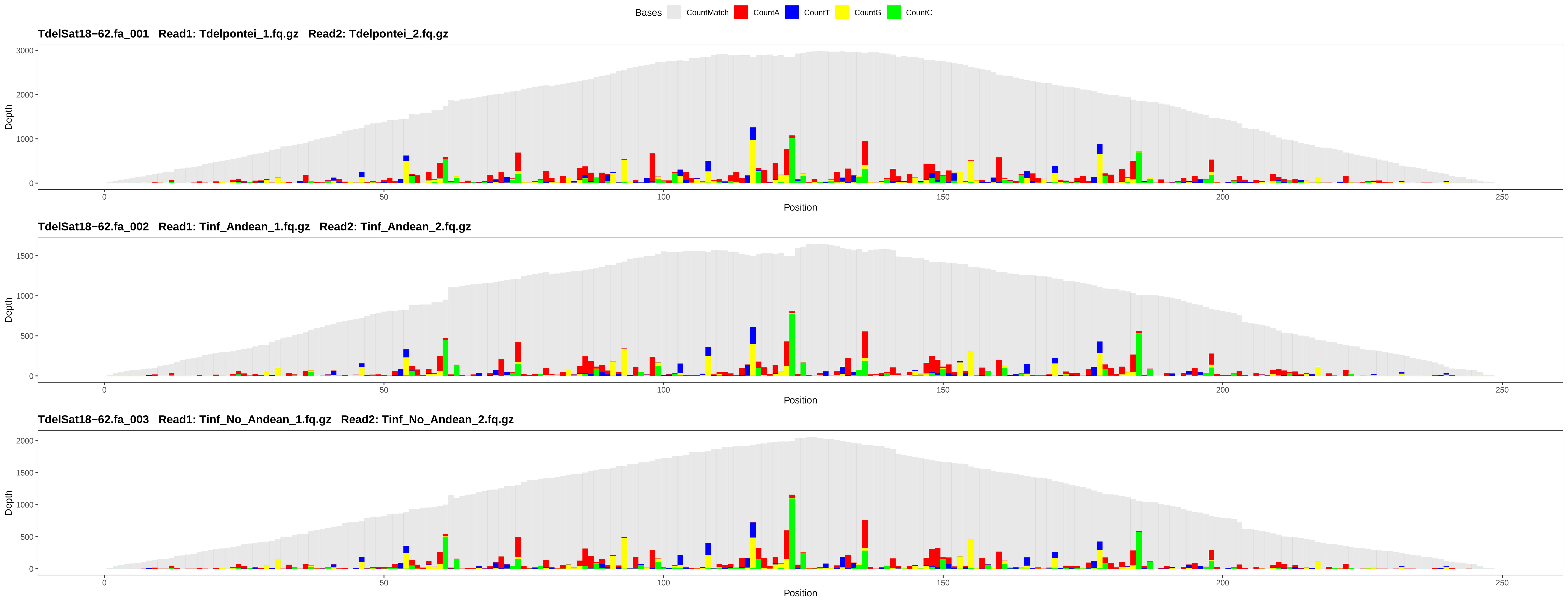
Bases
CountMatch
CountA
CountT
CountG
CountC
TdelSat18−62.fa_001 Read1: Tdelpontei_1.fq.gz Read2: Tdelpontei_2.fq.gz
Depth
0
1000
2000
3000
Position
0
50
100
150
200
250
TdelSat18−62.fa_002 Read1: Tinf_Andean_1.fq.gz Read2: Tinf_Andean_2.fq.gz
Depth
0
500
1000
1500
Position
0
50
100
150
200
250
TdelSat18−62.fa_003 Read1: Tinf_No_Andean_1.fq.gz Read2: Tinf_No_Andean_2.fq.gz
Depth
0
500
1000
1500
2000
Position
0
50
100
150
200
250

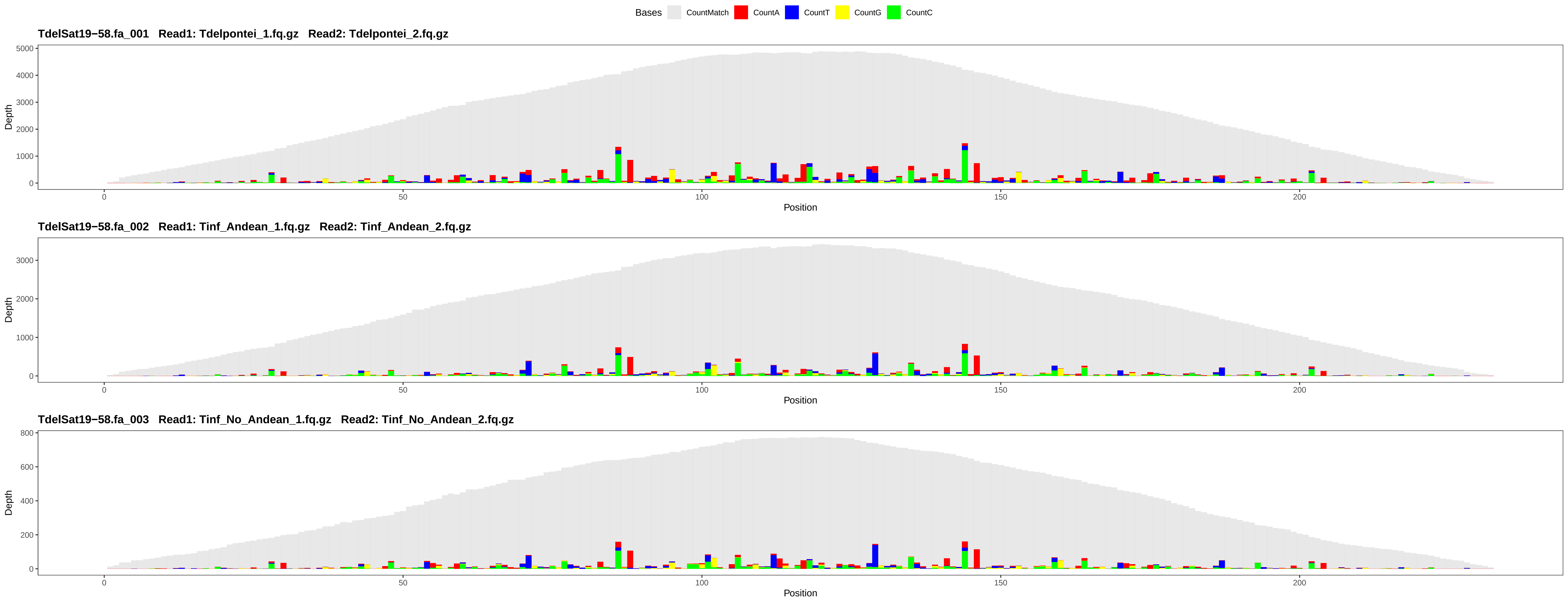
Bases
CountMatch
CountA
CountT
CountG
CountC
TdelSat19−58.fa_001 Read1: Tdelpontei_1.fq.gz Read2: Tdelpontei_2.fq.gz
Depth
0
1000
2000
3000
4000
5000
Position
0
50
100
150
200
TdelSat19−58.fa_002 Read1: Tinf_Andean_1.fq.gz Read2: Tinf_Andean_2.fq.gz
Depth
0
1000
2000
3000
Position
0
50
100
150
200
TdelSat19−58.fa_003 Read1: Tinf_No_Andean_1.fq.gz Read2: Tinf_No_Andean_2.fq.gz
Depth
0
200
400
600
800
Position
0
50
100
150
200

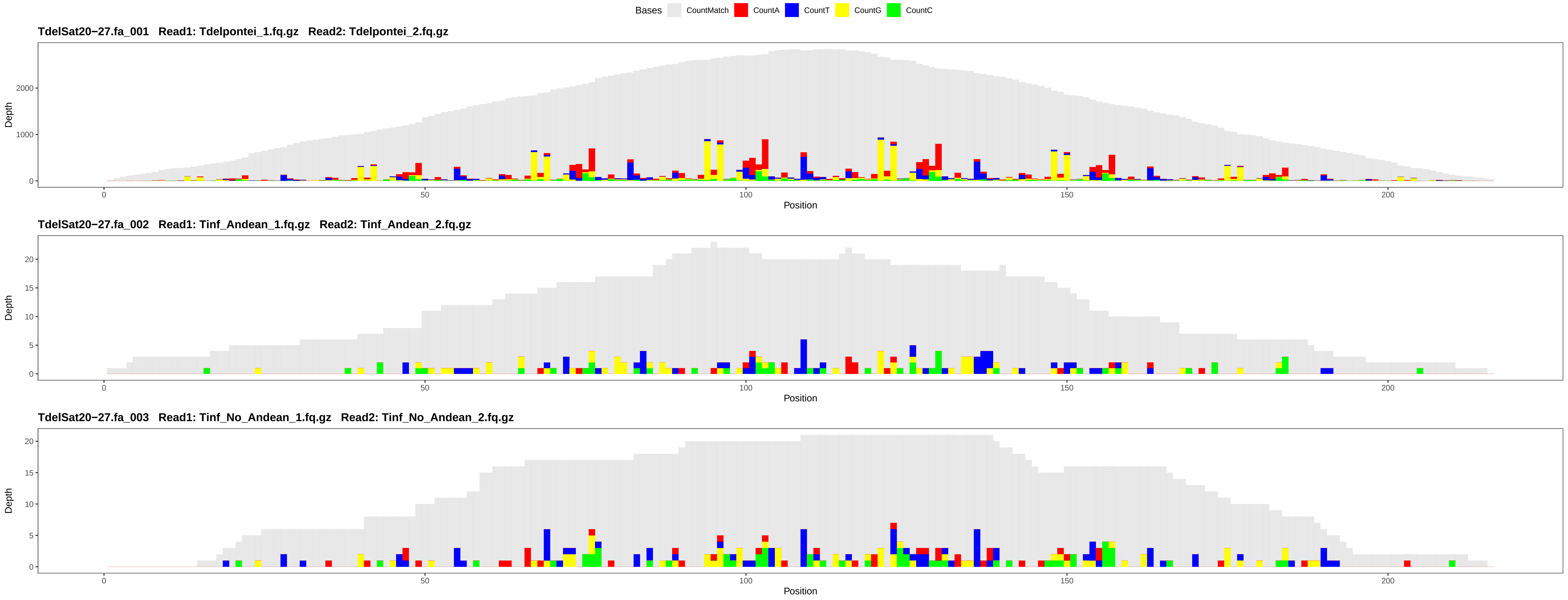
Bases CountMatch CountA CountT CountG CountC
TdelSat20−27.fa_001 Read1: Tdelpontei_1.fq.gz Read2: Tdelpontei_2.fq.gz
Depth
Position
TdelSat20−27.fa_002 Read1: Tinf_Andean_1.fq.gz Read2: Tinf_Andean_2.fq.gz
Depth
Position
TdelSat20−27.fa_003 Read1: Tinf_No_Andean_1.fq.gz Read2: Tinf_No_Andean_2.fq.gz
Depth
Position

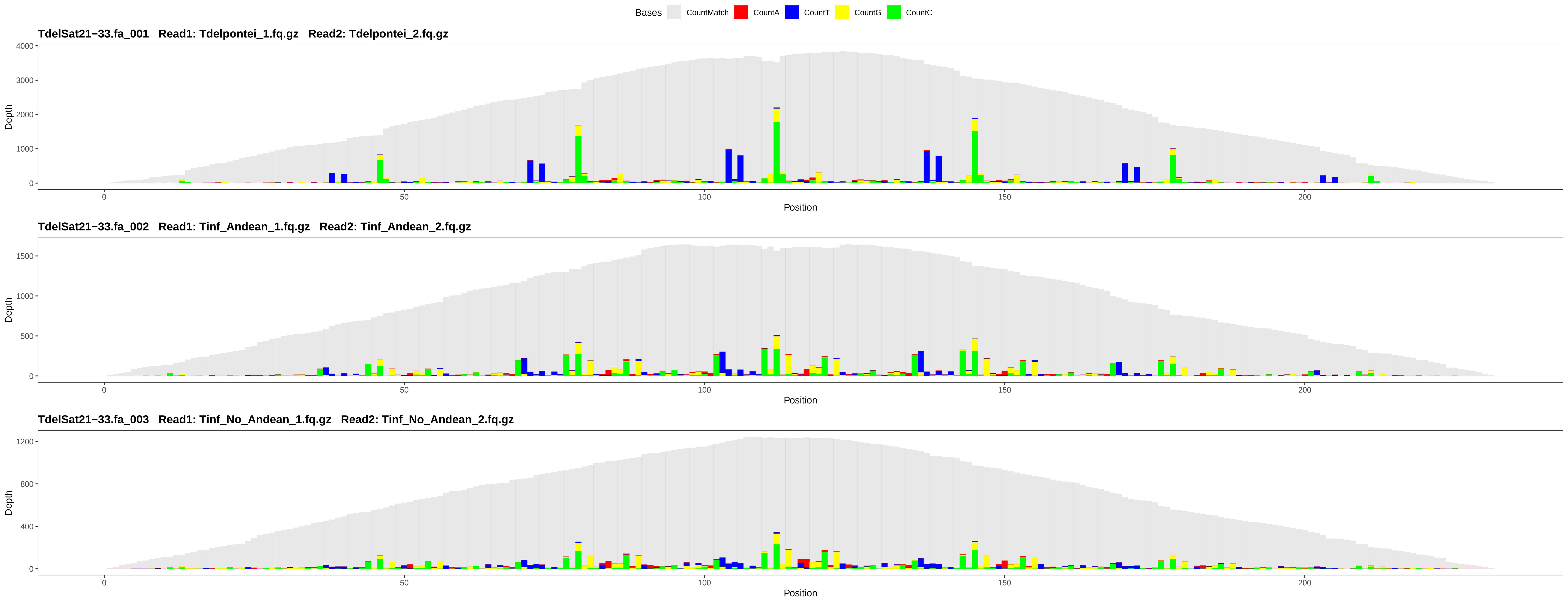
Bases CountMatch CountA CountT CountG CountC
TdelSat21−33.fa_001 Read1: Tdelpontei_1.fq.gz Read2: Tdelpontei_2.fq.gz
TdelSat21−33.fa_002 Read1: Tinf_Andean_1.fq.gz Read2: Tinf_Andean_2.fq.gz
TdelSat21−33.fa_003 Read1: Tinf_No_Andean_1.fq.gz Read2: Tinf_No_Andean_2.fq.gz
Depth
Position
0
50
100
150
200
400
800
1200
500
1000
1500
2000
3000
4000

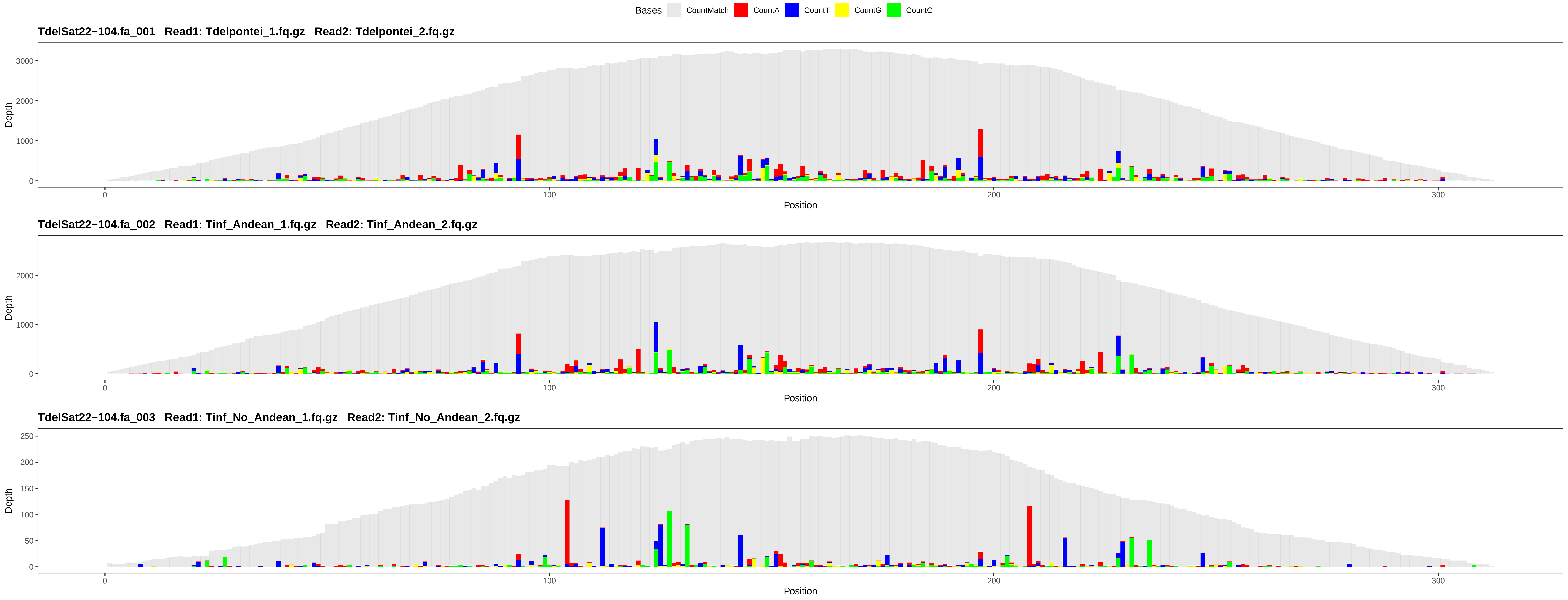
Bases
CountMatch
CountA
CountT
CountG
CountC
TdelSat22−104.fa_001 Read1: Tdelpontei_1.fq.gz Read2: Tdelpontei_2.fq.gz
TdelSat22−104.fa_002 Read1: Tinf_Andean_1.fq.gz Read2: Tinf_Andean_2.fq.gz
TdelSat22−104.fa_003 Read1: Tinf_No_Andean_1.fq.gz Read2: Tinf_No_Andean_2.fq.gz
Depth
Position
0
100
200
300
1000
2000
3000
50
150
250

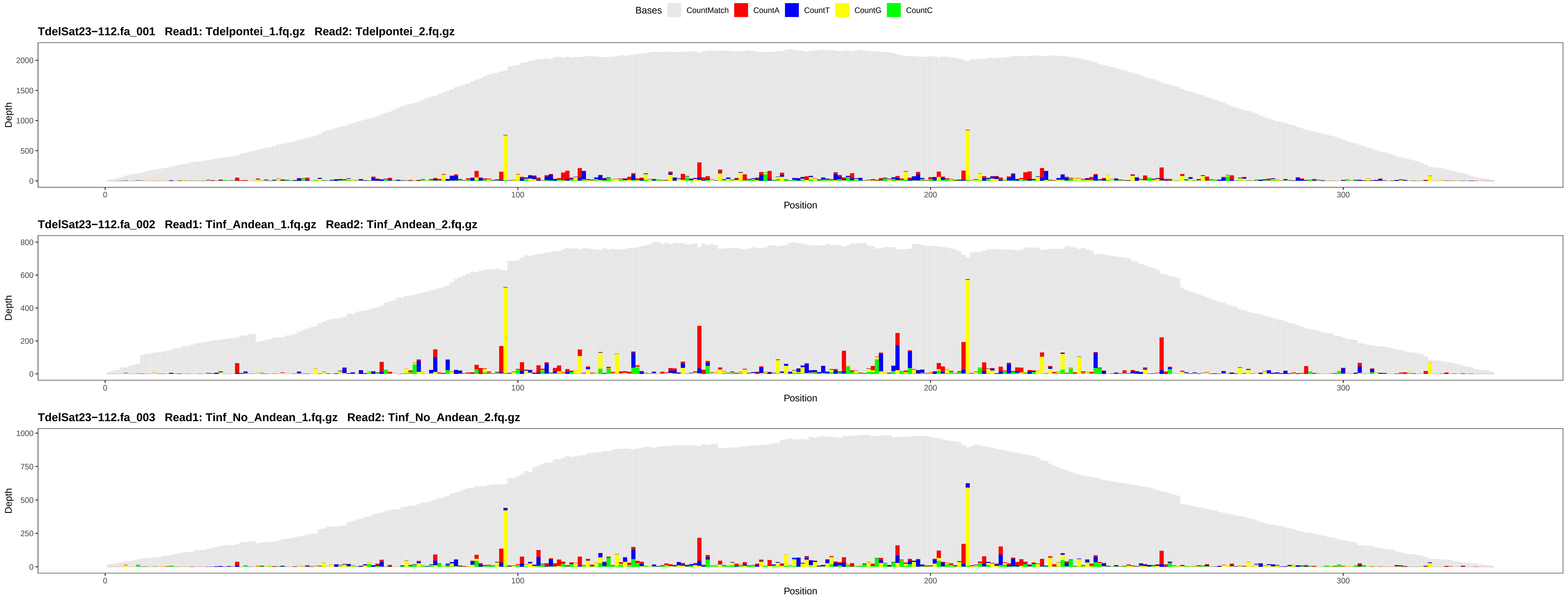
Bases CountMatch CountA CountT CountG CountC
TdelSat23−112.fa_001 Read1: Tdelpontei_1.fq.gz Read2: Tdelpontei_2.fq.gz
Depth
0
500
1000
1500
2000
Position
0
100
200
300
TdelSat23−112.fa_002 Read1: Tinf_Andean_1.fq.gz Read2: Tinf_Andean_2.fq.gz
Depth
0
200
400
600
800
Position
0
100
200
300
TdelSat23−112.fa_003 Read1: Tinf_No_Andean_1.fq.gz Read2: Tinf_No_Andean_2.fq.gz
Depth
0
250
500
750
1000
Position
0
100
200
300

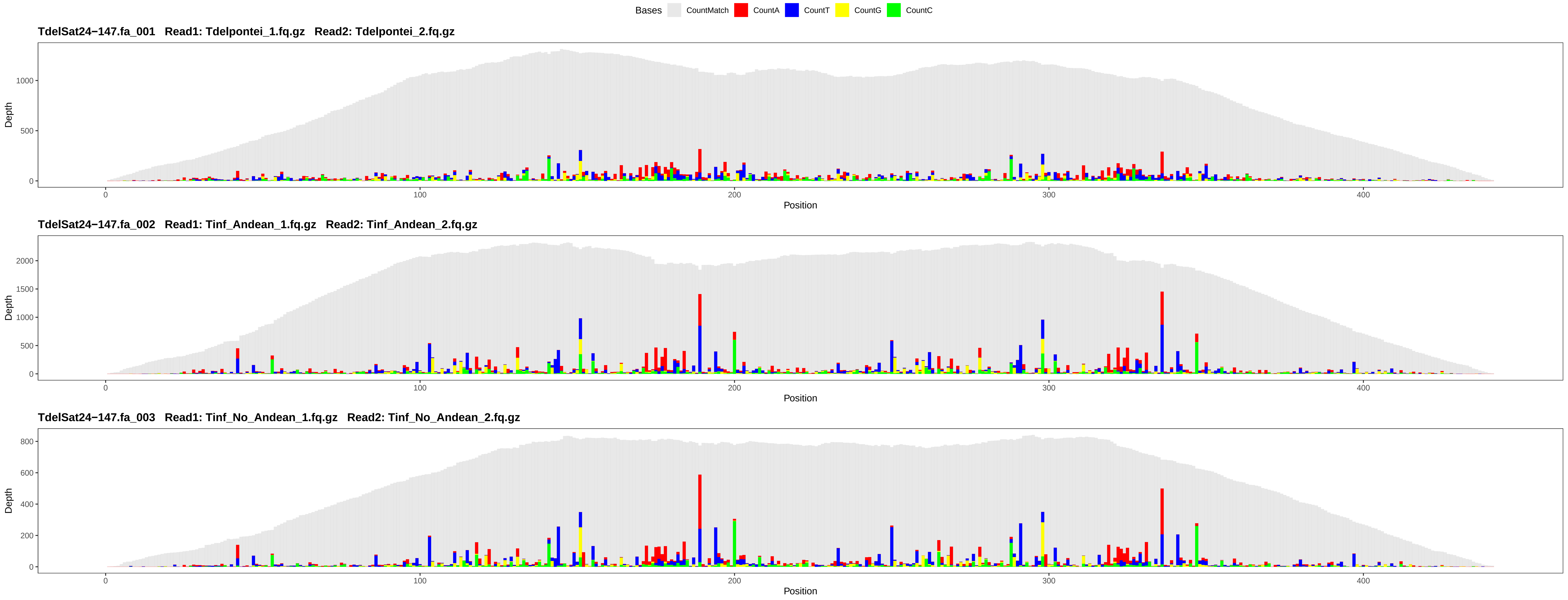

Bases CountMatch CountA CountT CountG CountC
TdelSat24−147.fa_001 Read1: Tdelpontei_1.fq.gz Read2: Tdelpontei_2.fq.gz
Depth
0
500
1000
Position
0
100
200
300
400
TdelSat24−147.fa_002 Read1: Tinf_Andean_1.fq.gz Read2: Tinf_Andean_2.fq.gz
Depth
0
500
1000
1500
2000
Position
0
100
200
300
400
TdelSat24−147.fa_003 Read1: Tinf_No_Andean_1.fq.gz Read2: Tinf_No_Andean_2.fq.gz
Depth
0
200
400
600
800
Position
0
100
200
300
400

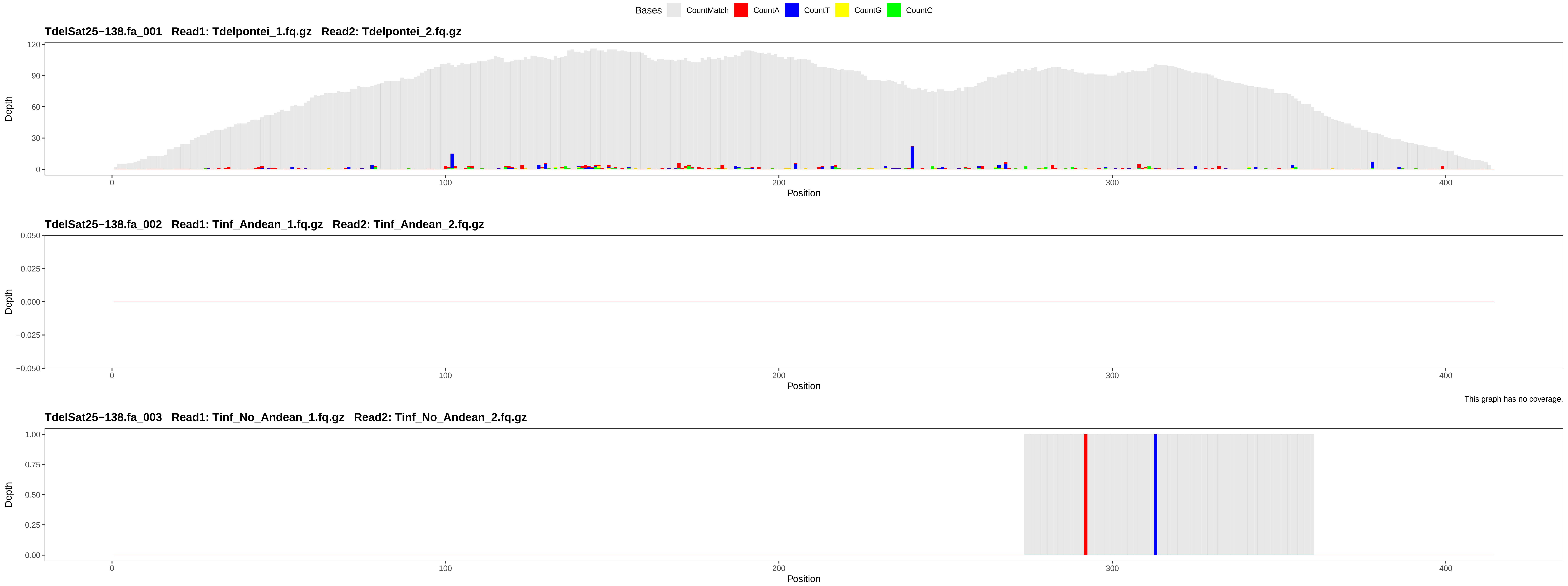

Bases
CountMatch
CountA
CountT
CountG
CountC
TdelSat25−138.fa_001 Read1: Tdelpontei_1.fq.gz Read2: Tdelpontei_2.fq.gz
Depth
Position
TdelSat25−138.fa_002 Read1: Tinf_Andean_1.fq.gz Read2: Tinf_Andean_2.fq.gz
Depth
Position
This graph has no coverage.
TdelSat25−138.fa_003 Read1: Tinf_No_Andean_1.fq.gz Read2: Tinf_No_Andean_2.fq.gz
Depth
Position

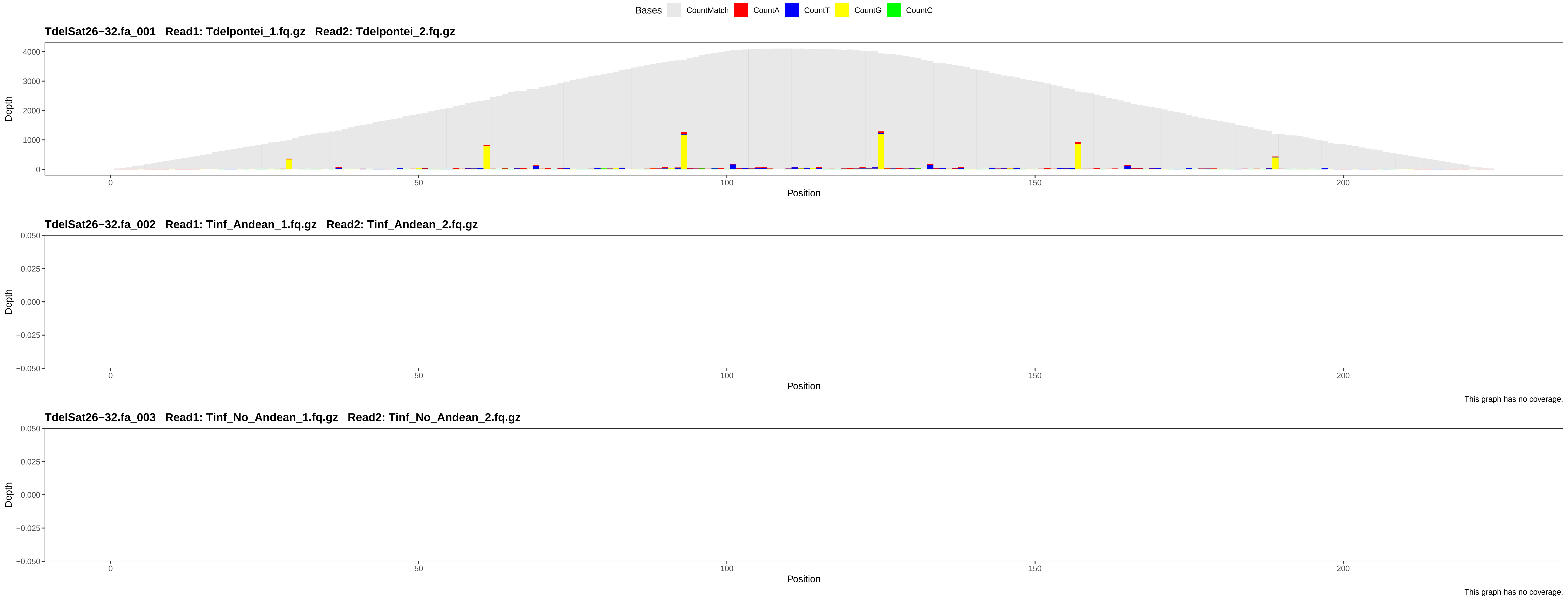
Bases CountMatch CountA CountT CountG CountC
TdelSat26−32.fa_001 Read1: Tdelpontei_1.fq.gz Read2: Tdelpontei_2.fq.gz
Depth
Position
TdelSat26−32.fa_002 Read1: Tinf_Andean_1.fq.gz Read2: Tinf_Andean_2.fq.gz
Depth
Position
This graph has no coverage.
TdelSat26−32.fa_003 Read1: Tinf_No_Andean_1.fq.gz Read2: Tinf_No_Andean_2.fq.gz
Depth
Position
This graph has no coverage.

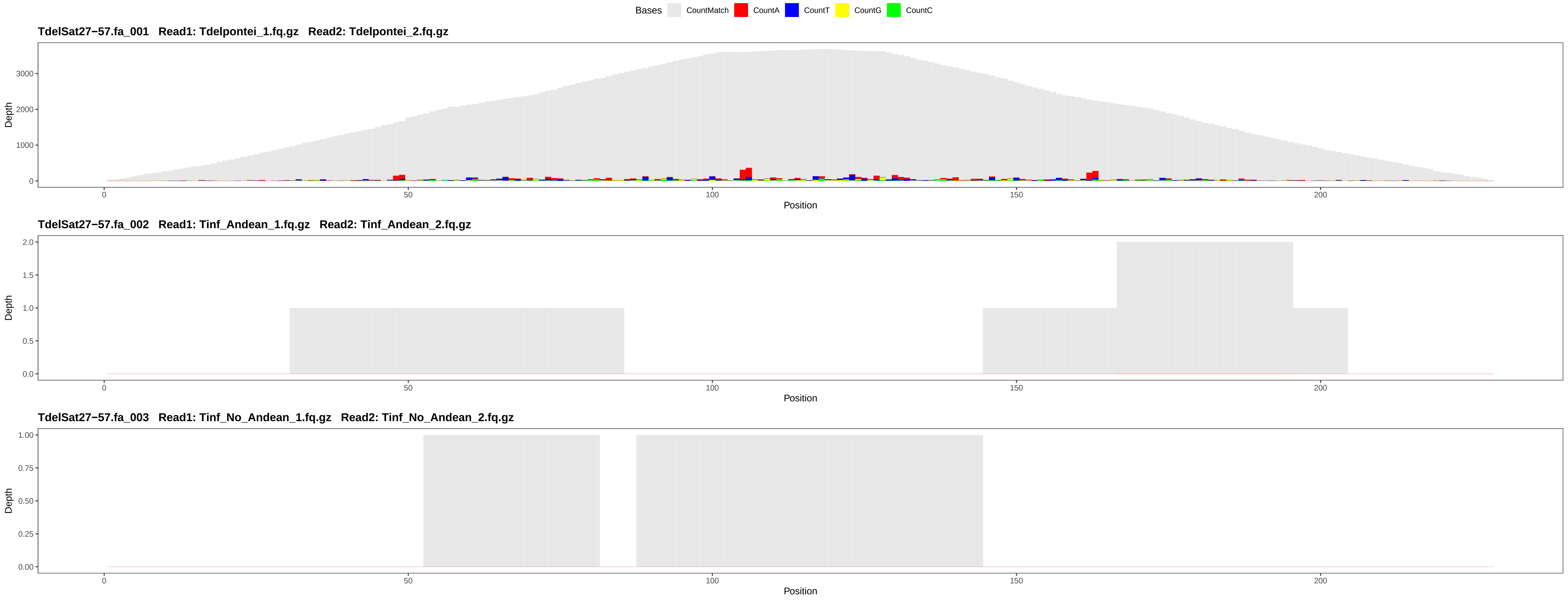
Bases CountMatch CountA CountT CountG CountC
TdelSat27−57.fa_001 Read1: Tdelpontei_1.fq.gz Read2: Tdelpontei_2.fq.gz
Depth
Position
TdelSat27−57.fa_002 Read1: Tinf_Andean_1.fq.gz Read2: Tinf_Andean_2.fq.gz
Depth
Position
TdelSat27−57.fa_003 Read1: Tinf_No_Andean_1.fq.gz Read2: Tinf_No_Andean_2.fq.gz
Depth
Position

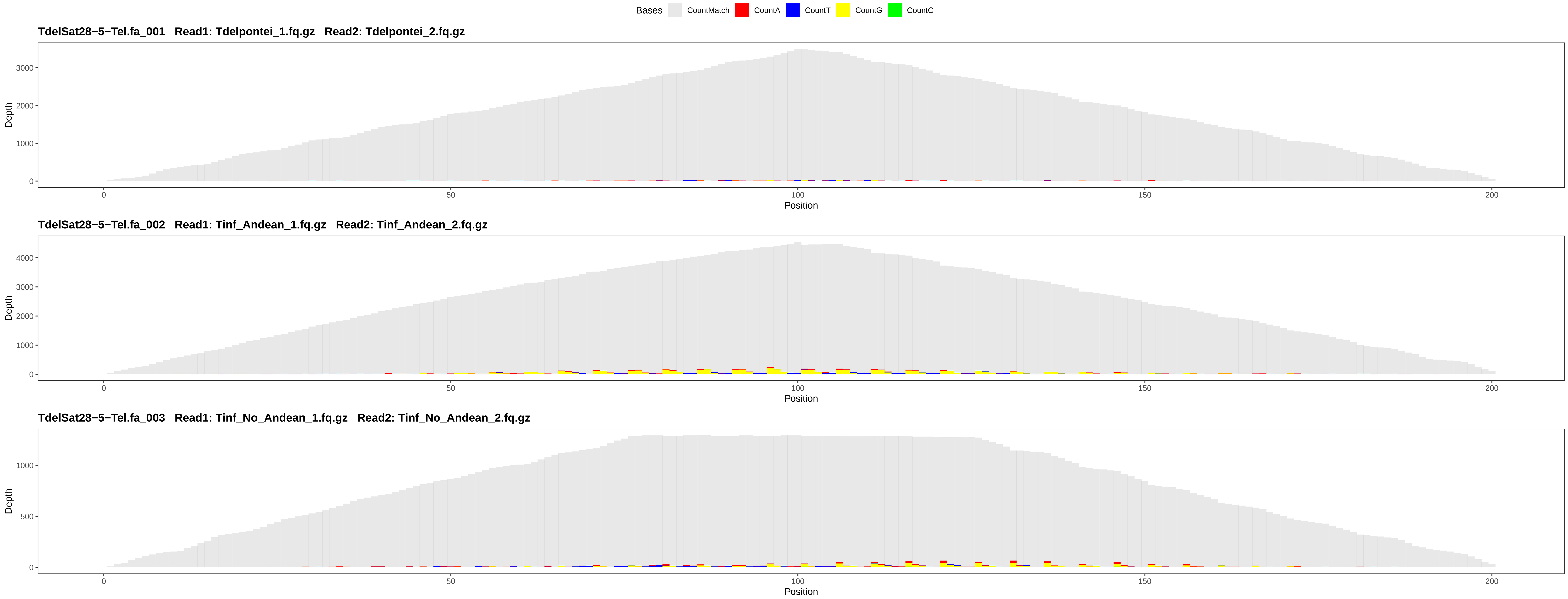

Bases CountMatch CountA CountT CountG CountC
TdelSat28−5−Tel.fa_001 Read1: Tdelpontei_1.fq.gz Read2: Tdelpontei_2.fq.gz
Depth
Position
TdelSat28−5−Tel.fa_002 Read1: Tinf_Andean_1.fq.gz Read2: Tinf_Andean_2.fq.gz
Depth
Position
TdelSat28−5−Tel.fa_003 Read1: Tinf_No_Andean_1.fq.gz Read2: Tinf_No_Andean_2.fq.gz
Depth
Position

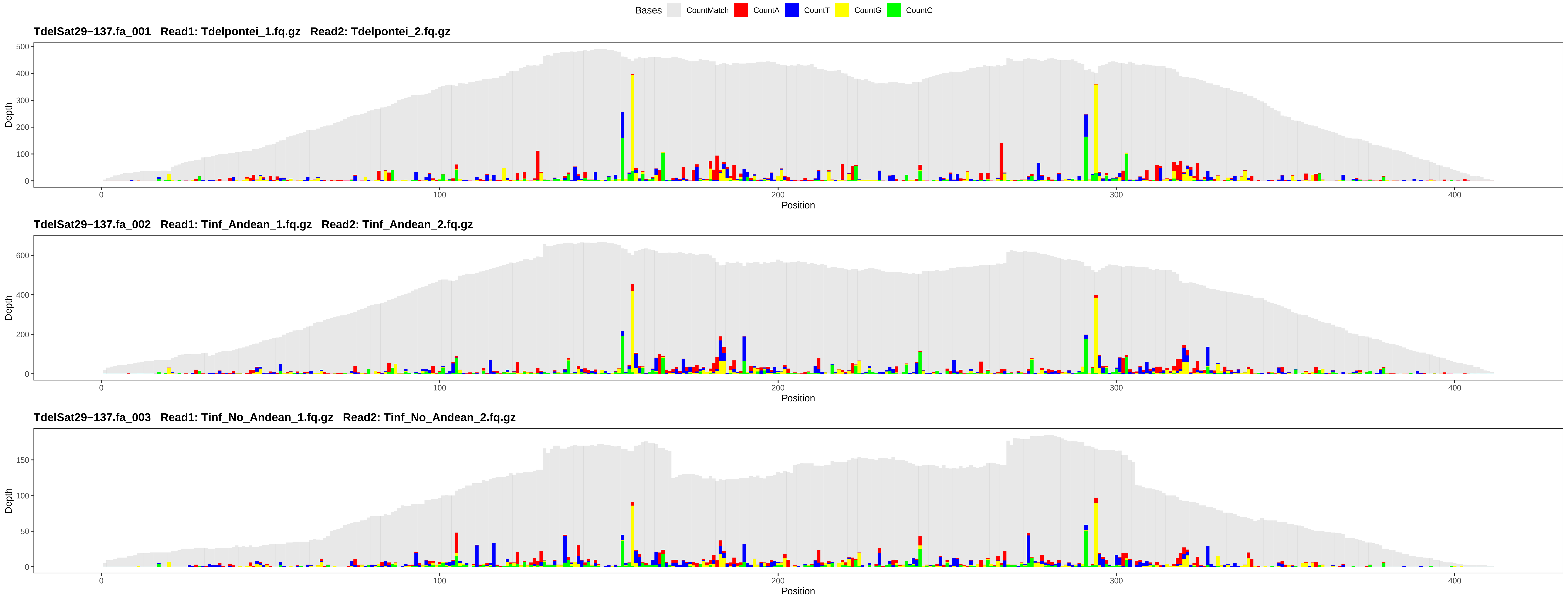

Bases
CountMatch
CountA
CountT
CountG
CountC
TdelSat29−137.fa_001   Read1: Tdelpontei_1.fq.gz   Read2: Tdelpontei_2.fq.gz
Depth
Position
TdelSat29−137.fa_002   Read1: Tinf_Andean_1.fq.gz   Read2: Tinf_Andean_2.fq.gz
Depth
Position
TdelSat29−137.fa_003   Read1: Tinf_No_Andean_1.fq.gz   Read2: Tinf_No_Andean_2.fq.gz
Depth
Position

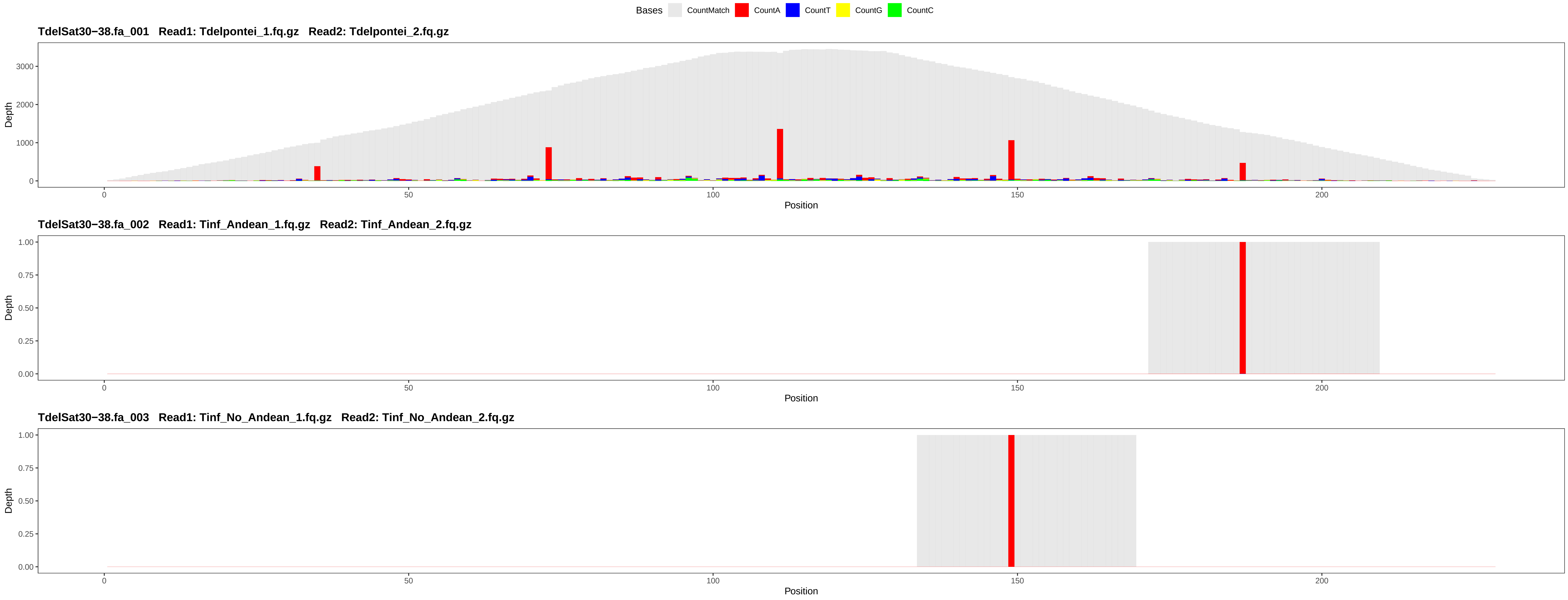
Bases CountMatch CountA CountT CountG CountC
TdelSat30−38.fa_001 Read1: Tdelpontei_1.fq.gz Read2: Tdelpontei_2.fq.gz
Depth
0
1000
2000
3000
0
50
100
150
200
Position
TdelSat30−38.fa_002 Read1: Tinf_Andean_1.fq.gz Read2: Tinf_Andean_2.fq.gz
Depth
0.00
0.25
0.50
0.75
1.00
0
50
100
150
200
Position
TdelSat30−38.fa_003 Read1: Tinf_No_Andean_1.fq.gz Read2: Tinf_No_Andean_2.fq.gz
Depth
0.00
0.25
0.50
0.75
1.00
0
50
100
150
200
Position

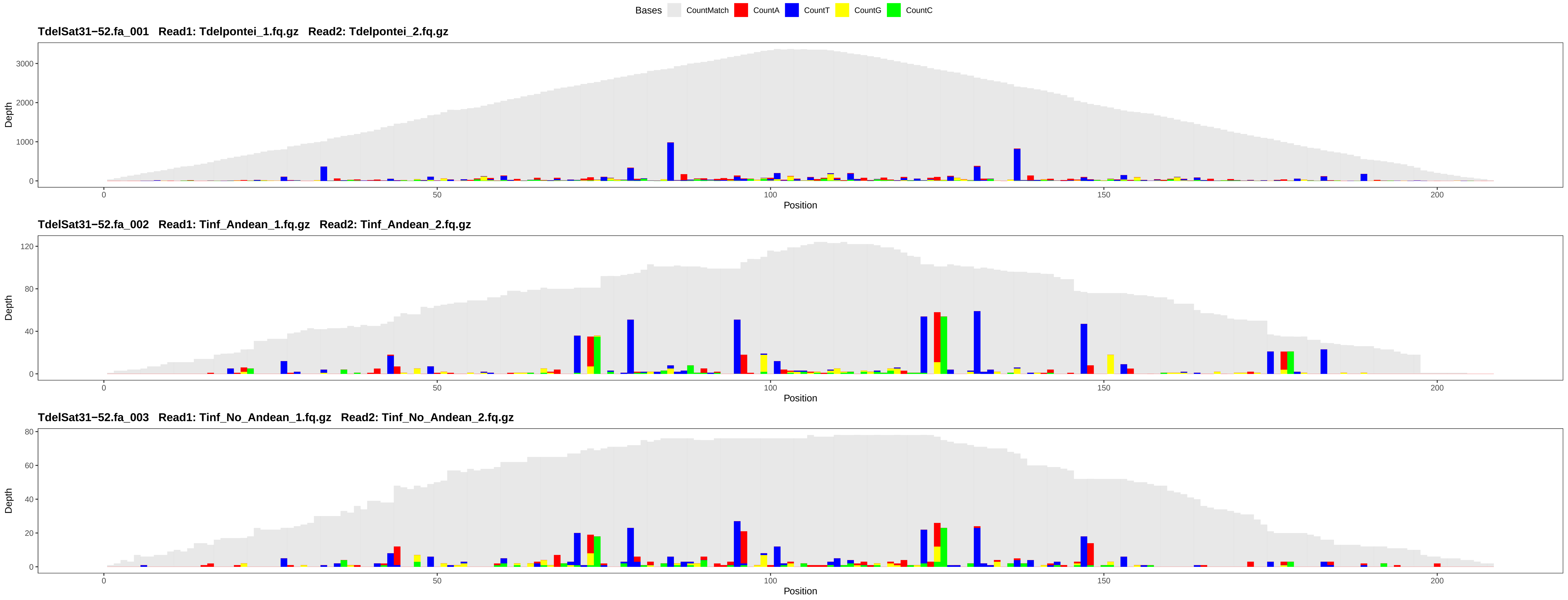

Bases CountMatch CountA CountT CountG CountC
TdelSat31−52.fa_001 Read1: Tdelpontei_1.fq.gz Read2: Tdelpontei_2.fq.gz
Depth
0
1000
2000
3000
Position
0
50
100
150
200
TdelSat31−52.fa_002 Read1: Tinf_Andean_1.fq.gz Read2: Tinf_Andean_2.fq.gz
Depth
0
40
80
120
Position
0
50
100
150
200
TdelSat31−52.fa_003 Read1: Tinf_No_Andean_1.fq.gz Read2: Tinf_No_Andean_2.fq.gz
Depth
0
20
40
60
80
Position
0
50
100
150
200

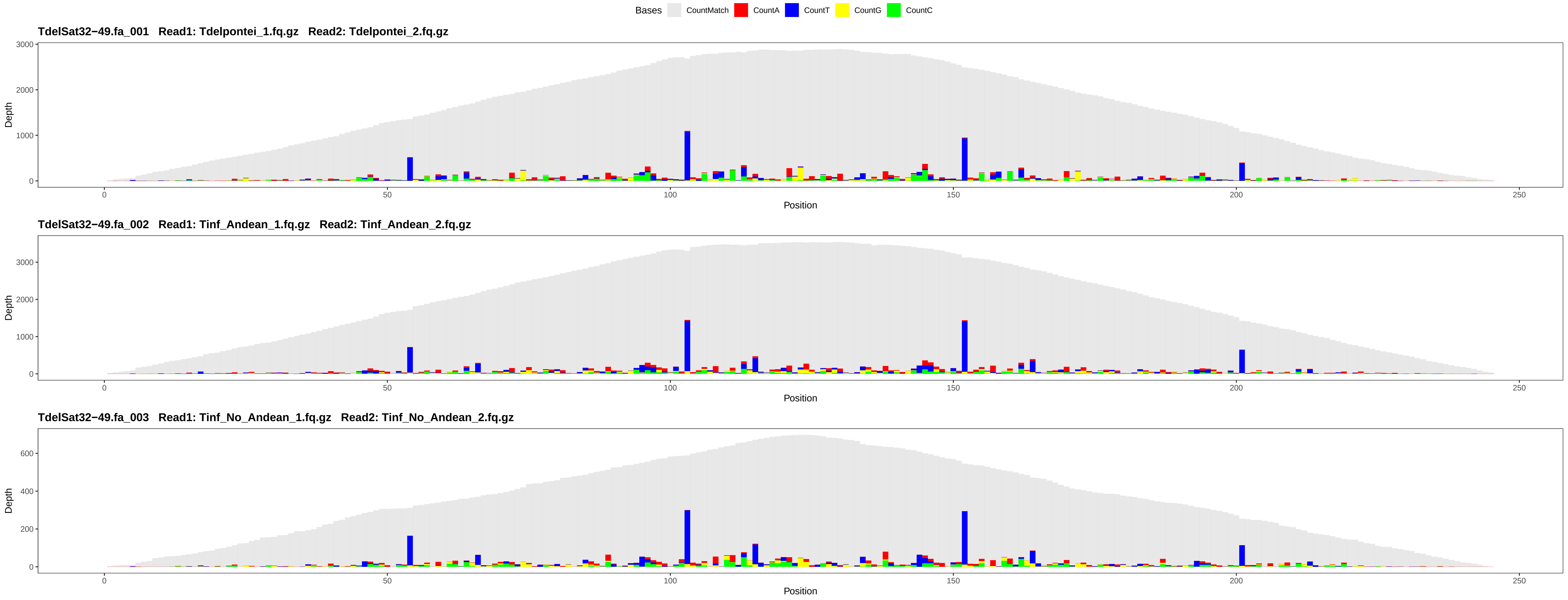
Bases CountMatch CountA CountT CountG CountC
TdelSat32−49.fa_001 Read1: Tdelpontei_1.fq.gz Read2: Tdelpontei_2.fq.gz
Depth
0
1000
2000
3000
0 50 100 150 200 250
Position
TdelSat32−49.fa_002 Read1: Tinf_Andean_1.fq.gz Read2: Tinf_Andean_2.fq.gz
Depth
0
1000
2000
3000
0 50 100 150 200 250
Position
TdelSat32−49.fa_003 Read1: Tinf_No_Andean_1.fq.gz Read2: Tinf_No_Andean_2.fq.gz
Depth
0
200
400
600
0 50 100 150 200 250
Position

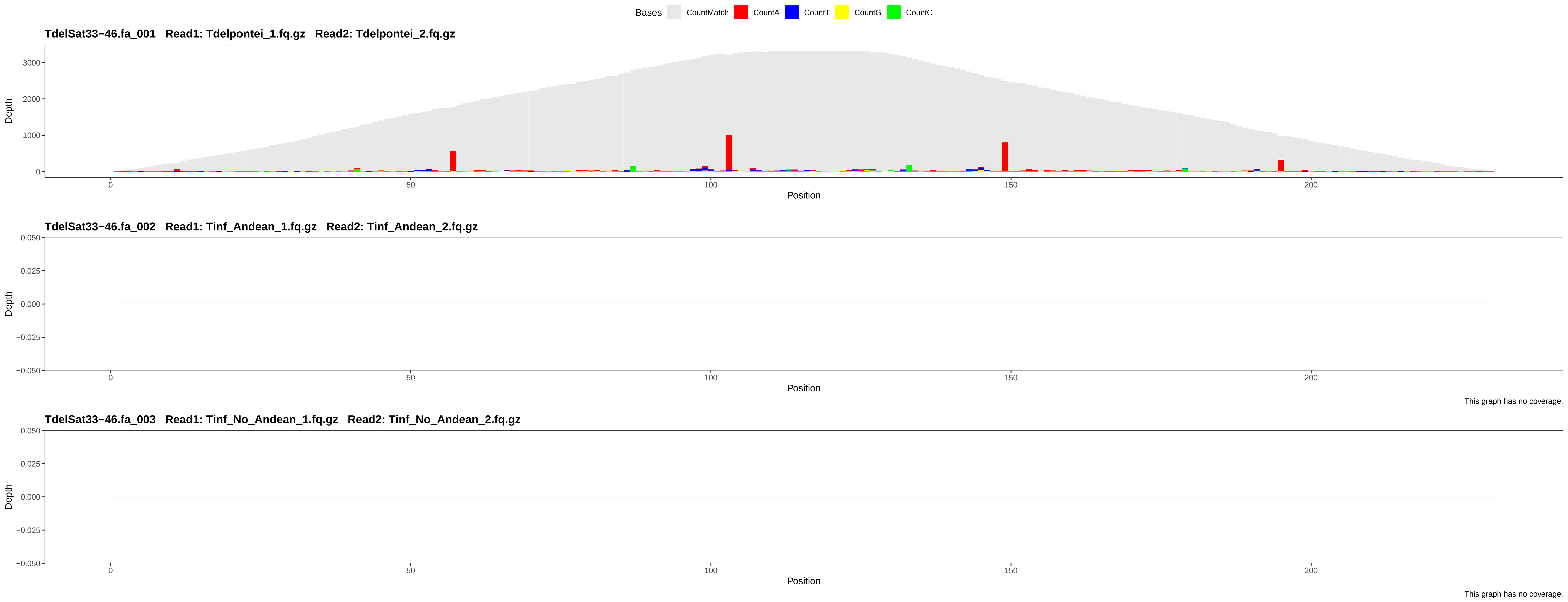

Bases CountMatch CountA CountT CountG CountC
TdelSat33−46.fa_001 Read1: Tdelpontei_1.fq.gz Read2: Tdelpontei_2.fq.gz
Depth
3000
2000
1000
0
0
50
100
150
200
Position
TdelSat33−46.fa_002 Read1: Tinf_Andean_1.fq.gz Read2: Tinf_Andean_2.fq.gz
Depth
0.050
0.025
0.000
−0.025
−0.050
0
50
100
150
200
Position
This graph has no coverage.
TdelSat33−46.fa_003 Read1: Tinf_No_Andean_1.fq.gz Read2: Tinf_No_Andean_2.fq.gz
Depth
0.050
0.025
0.000
−0.025
−0.050
0
50
100
150
200
Position
This graph has no coverage.

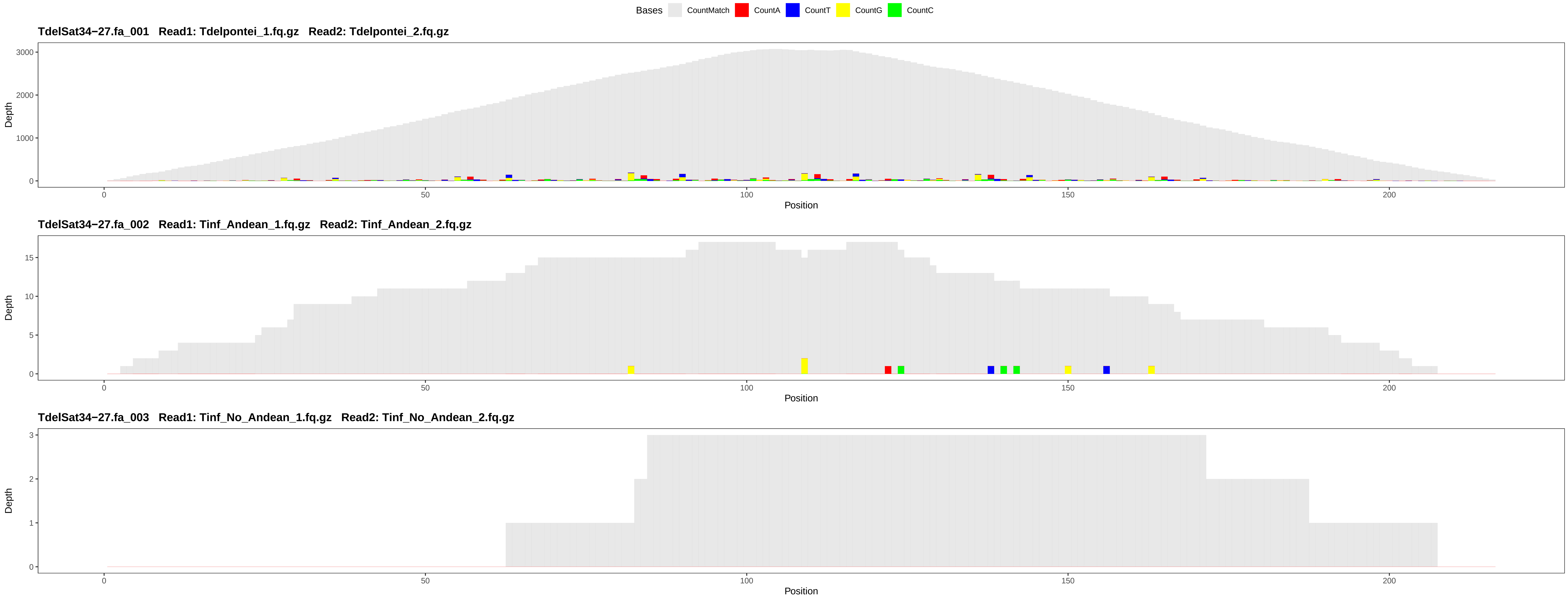

Bases
CountMatch
CountA
CountT
CountG
CountC
TdelSat34−27.fa_001 Read1: Tdelpontei_1.fq.gz Read2: Tdelpontei_2.fq.gz
Depth
Position
TdelSat34−27.fa_002 Read1: Tinf_Andean_1.fq.gz Read2: Tinf_Andean_2.fq.gz
Depth
Position
TdelSat34−27.fa_003 Read1: Tinf_No_Andean_1.fq.gz Read2: Tinf_No_Andean_2.fq.gz
Depth
Position

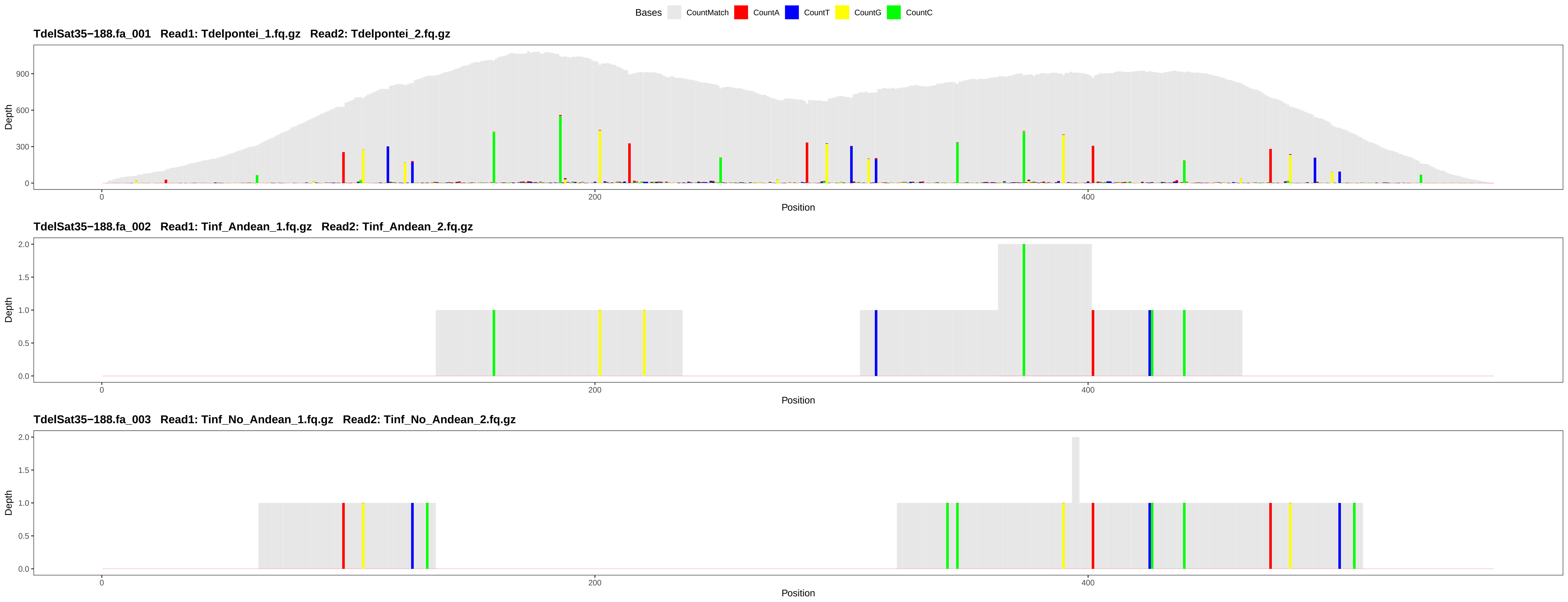

Bases
CountMatch
CountA
CountT
CountG
CountC
TdelSat35−188.fa_001   Read1: Tdelpontei_1.fq.gz   Read2: Tdelpontei_2.fq.gz
Depth
900
600
300
0
0
200
400
Position
TdelSat35−188.fa_002   Read1: Tinf_Andean_1.fq.gz   Read2: Tinf_Andean_2.fq.gz
Depth
2.0
1.5
1.0
0.5
0.0
0
200
400
Position
TdelSat35−188.fa_003   Read1: Tinf_No_Andean_1.fq.gz   Read2: Tinf_No_Andean_2.fq.gz
Depth
2.0
1.5
1.0
0.5
0.0
0
200
400
Position

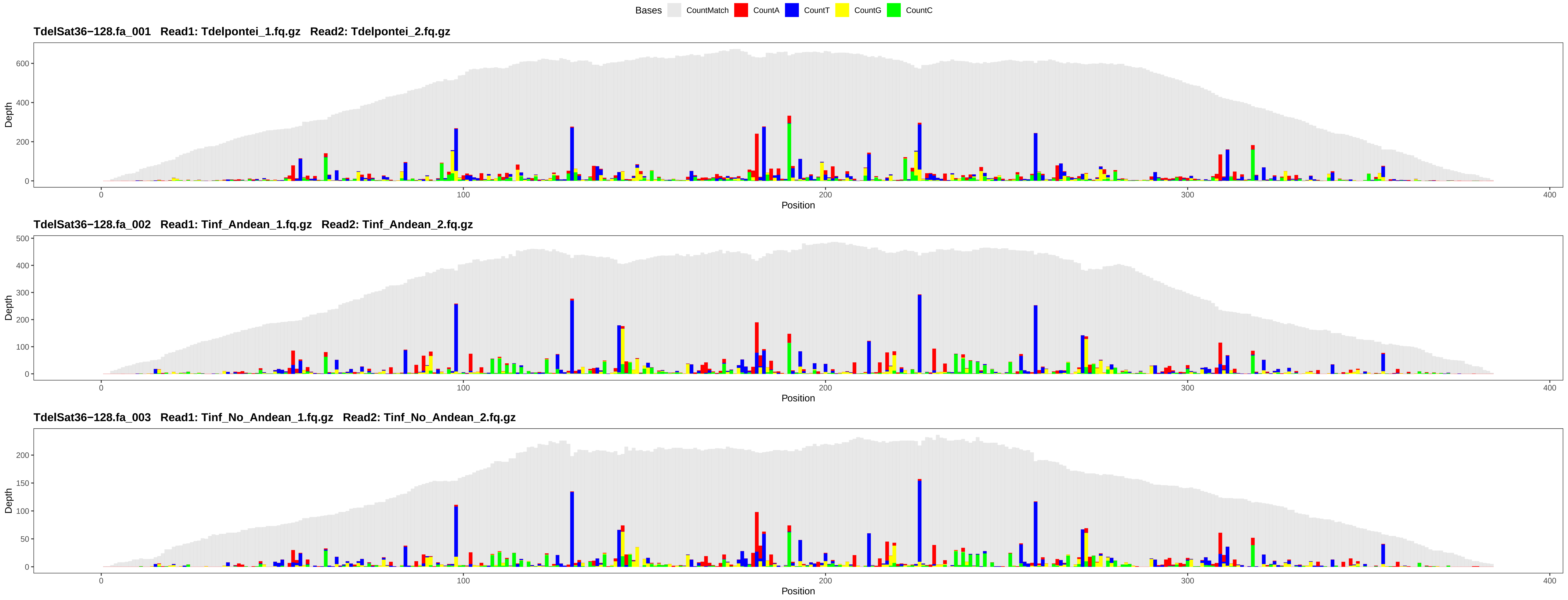
Bases CountMatch CountA CountT CountG CountC
TdelSat36−128.fa_001 Read1: Tdelpontei_1.fq.gz Read2: Tdelpontei_2.fq.gz
Depth
Position
TdelSat36−128.fa_002 Read1: Tinf_Andean_1.fq.gz Read2: Tinf_Andean_2.fq.gz
Depth
Position
TdelSat36−128.fa_003 Read1: Tinf_No_Andean_1.fq.gz Read2: Tinf_No_Andean_2.fq.gz
Depth
Position

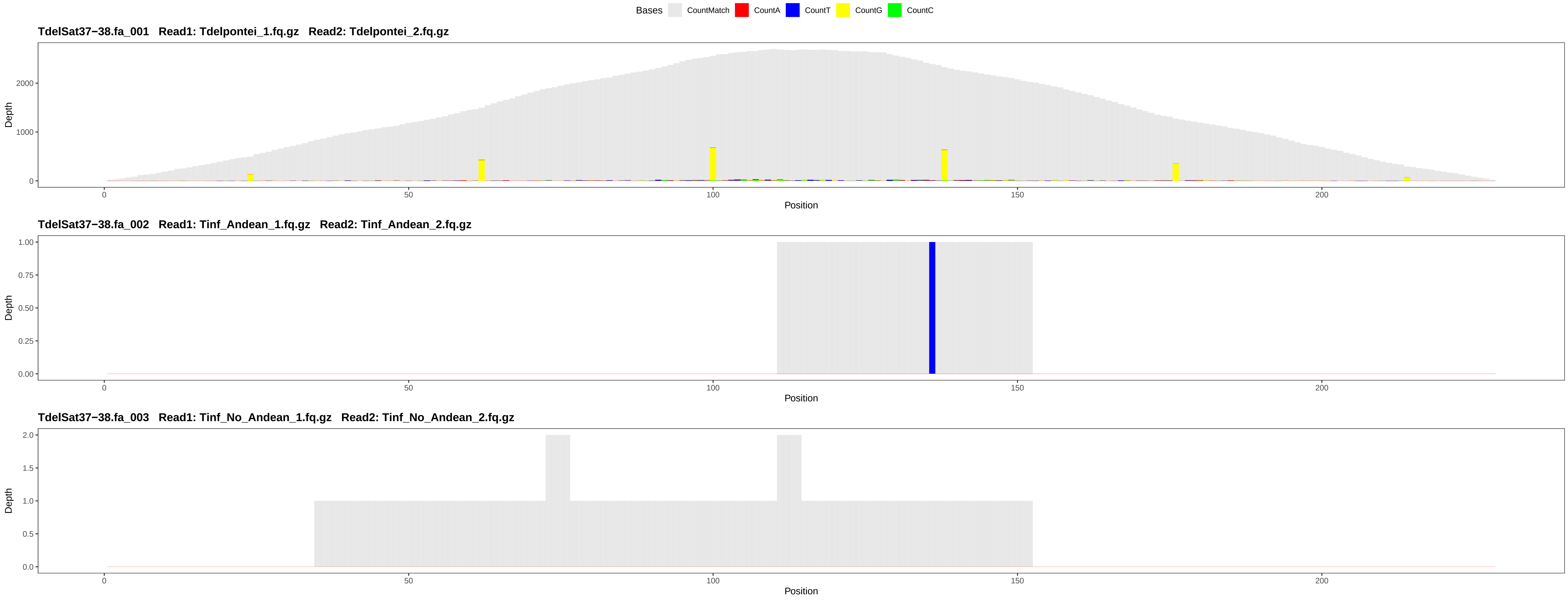
Bases
CountMatch
CountA
CountT
CountG
CountC
TdelSat37−38.fa_001 Read1: Tdelpontei_1.fq.gz Read2: Tdelpontei_2.fq.gz
Depth
0
1000
2000
0
50
100
150
200
Position
TdelSat37−38.fa_002 Read1: Tinf_Andean_1.fq.gz Read2: Tinf_Andean_2.fq.gz
Depth
0.00
0.25
0.50
0.75
1.00
0
50
100
150
200
Position
TdelSat37−38.fa_003 Read1: Tinf_No_Andean_1.fq.gz Read2: Tinf_No_Andean_2.fq.gz
Depth
0.0
0.5
1.0
1.5
2.0
0
50
100
150
200
Position

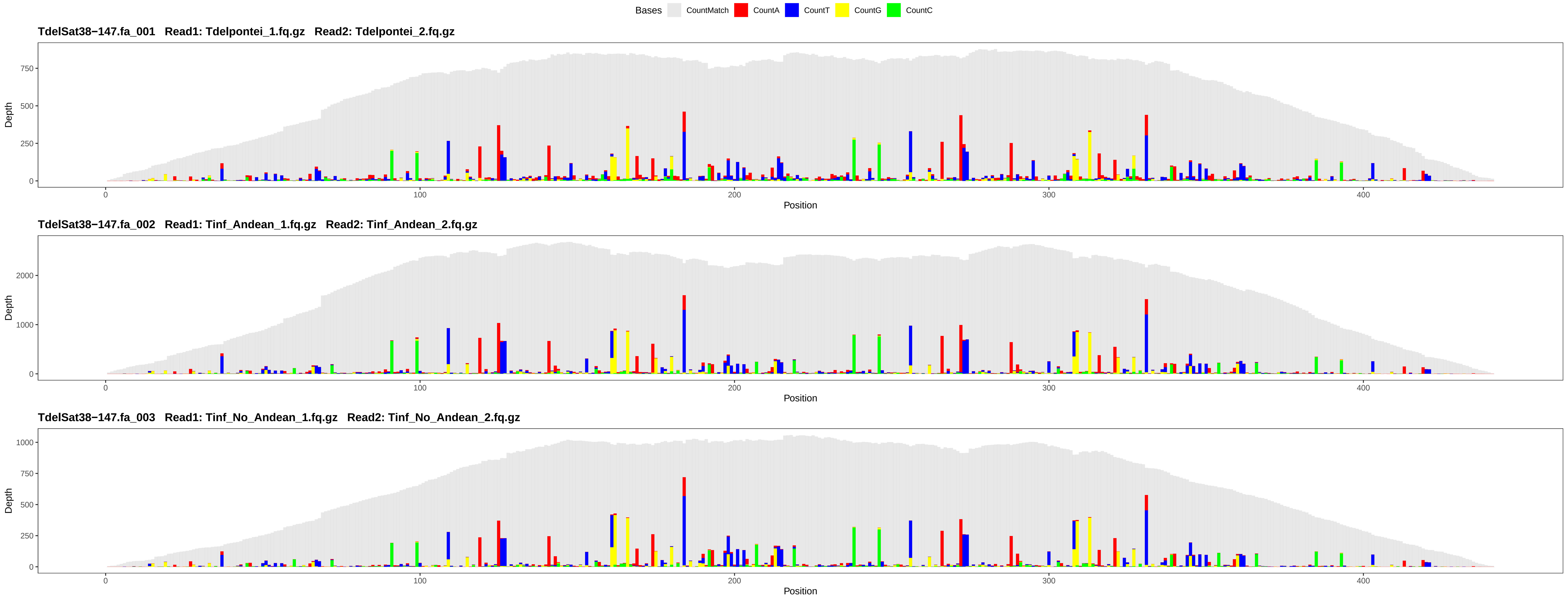
Bases
CountMatch
CountA
CountT
CountG
CountC
TdelSat38−147.fa_001   Read1: Tdelpontei_1.fq.gz   Read2: Tdelpontei_2.fq.gz
Depth
0
250
500
750
0
100
200
300
400
Position
TdelSat38−147.fa_002   Read1: Tinf_Andean_1.fq.gz   Read2: Tinf_Andean_2.fq.gz
Depth
0
1000
2000
0
100
200
300
400
Position
TdelSat38−147.fa_003   Read1: Tinf_No_Andean_1.fq.gz   Read2: Tinf_No_Andean_2.fq.gz
Depth
0
250
500
750
1000
0
100
200
300
400
Position

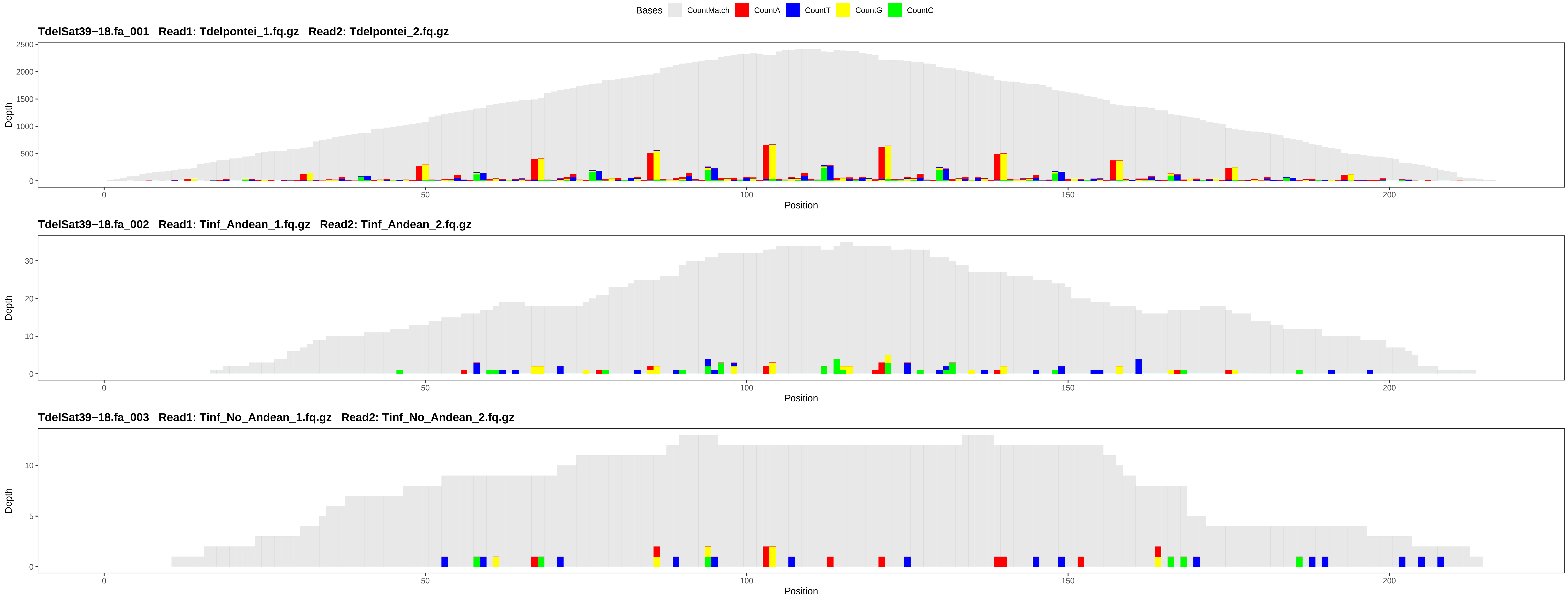

Bases CountMatch CountA CountT CountG CountC
TdelSat39−18.fa_001 Read1: Tdelpontei_1.fq.gz Read2: Tdelpontei_2.fq.gz
Depth
Position
TdelSat39−18.fa_002 Read1: Tinf_Andean_1.fq.gz Read2: Tinf_Andean_2.fq.gz
Depth
Position
TdelSat39−18.fa_003 Read1: Tinf_No_Andean_1.fq.gz Read2: Tinf_No_Andean_2.fq.gz
Depth
Position

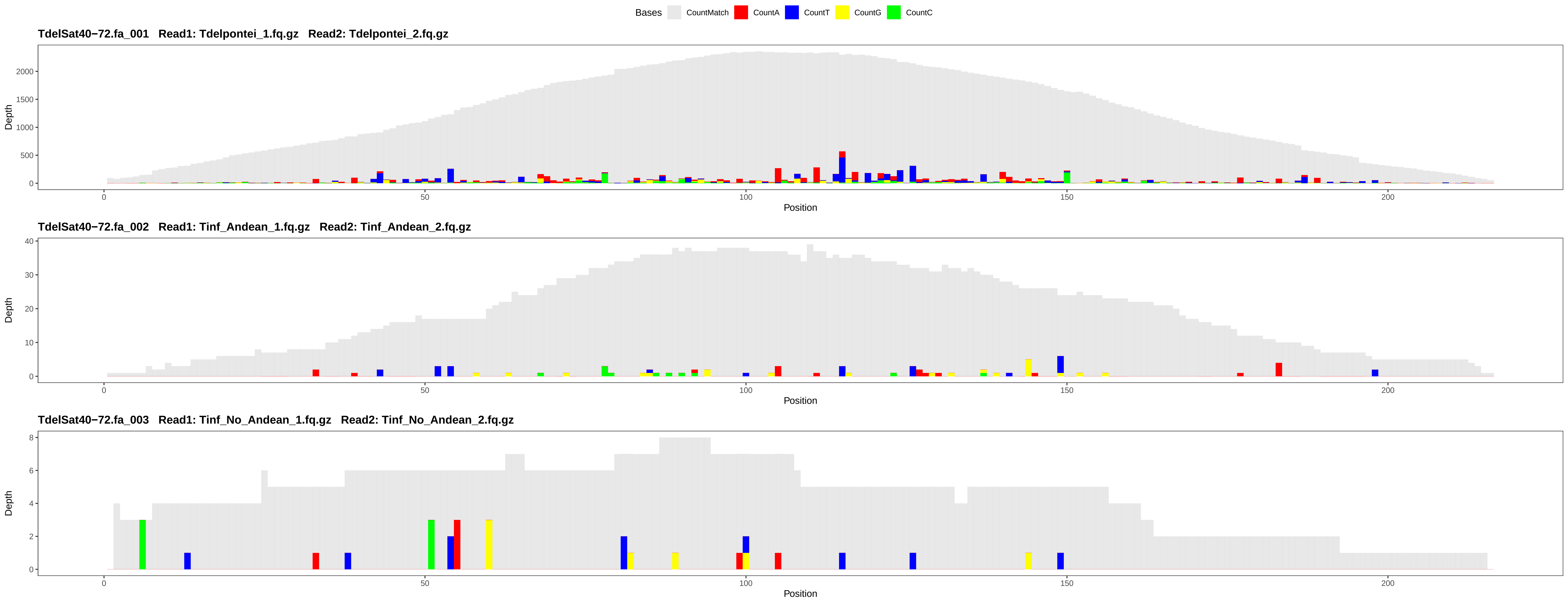
Bases CountMatch CountA CountT CountG CountC
TdelSat40−72.fa_001 Read1: Tdelpontei_1.fq.gz Read2: Tdelpontei_2.fq.gz
Depth
Position
TdelSat40−72.fa_002 Read1: Tinf_Andean_1.fq.gz Read2: Tinf_Andean_2.fq.gz
Depth
Position
TdelSat40−72.fa_003 Read1: Tinf_No_Andean_1.fq.gz Read2: Tinf_No_Andean_2.fq.gz
Depth
Position

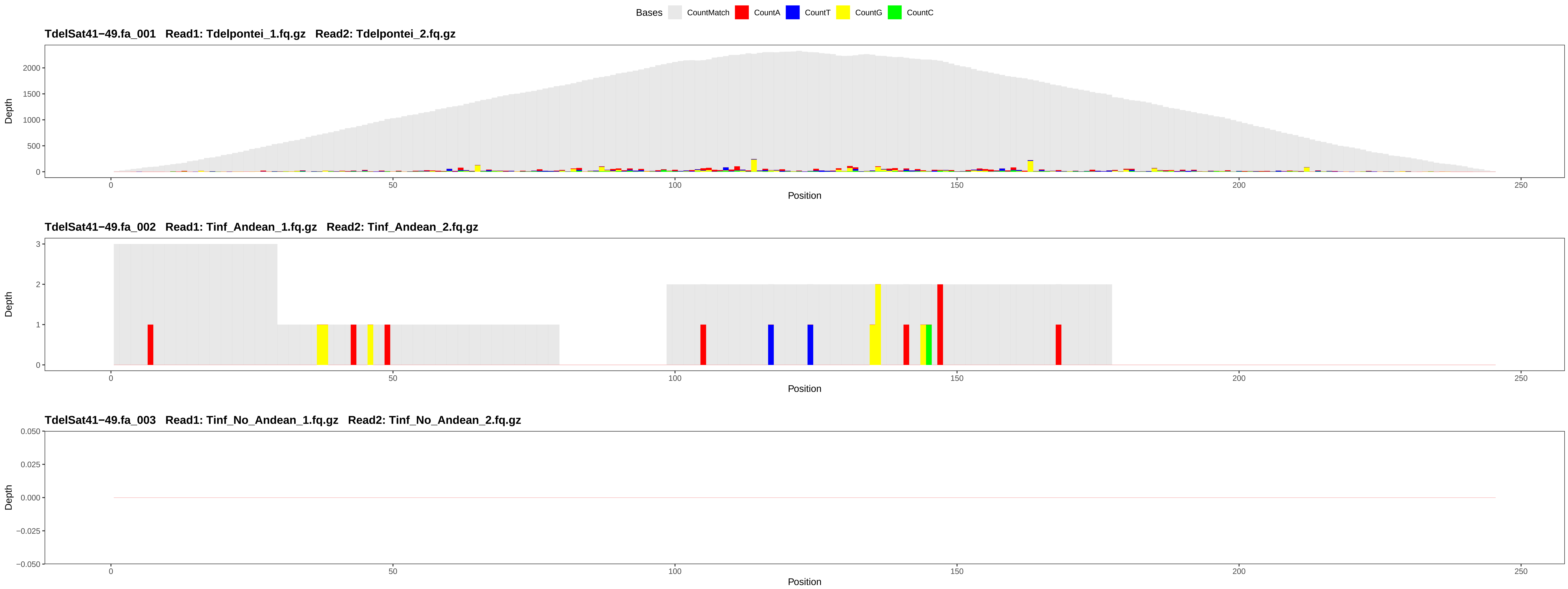

This graph has no coverage.

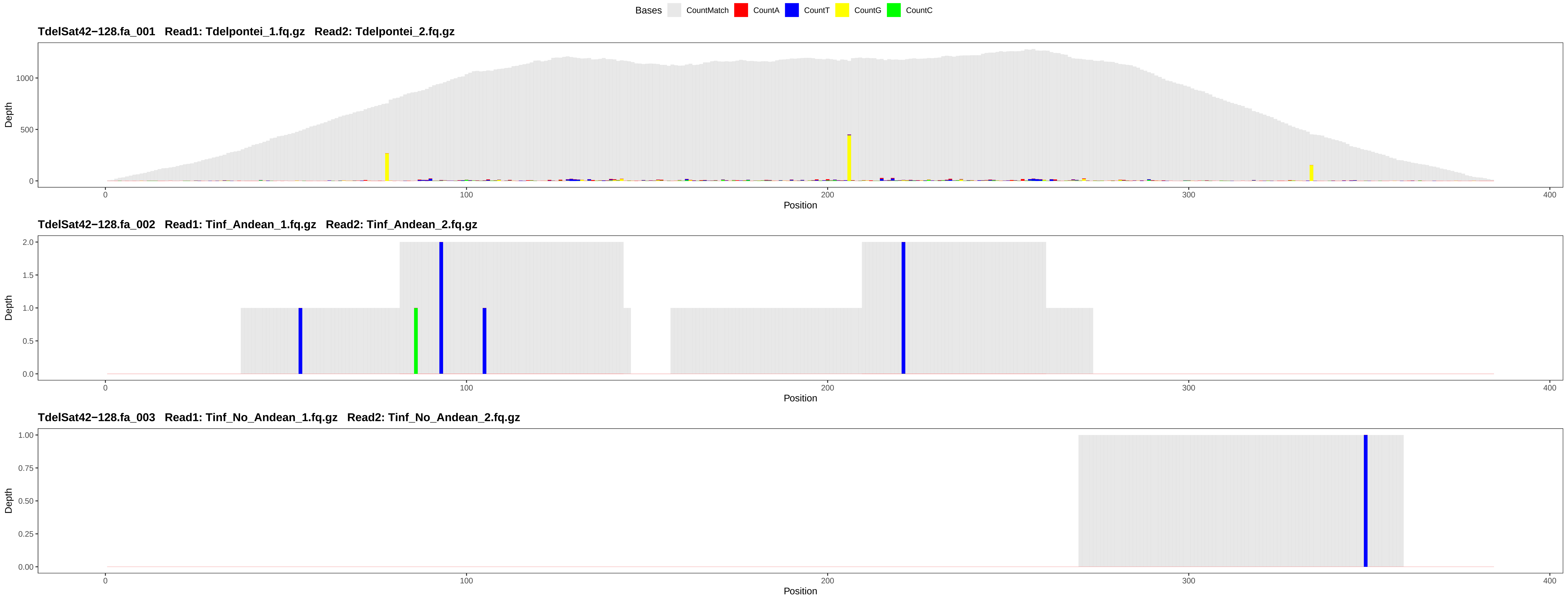

Bases
CountMatch
CountA
CountT
CountG
CountC
TdelSat42−128.fa_001 Read1: Tdelpontei_1.fq.gz Read2: Tdelpontei_2.fq.gz
Depth
Position
TdelSat42−128.fa_002 Read1: Tinf_Andean_1.fq.gz Read2: Tinf_Andean_2.fq.gz
Depth
Position
TdelSat42−128.fa_003 Read1: Tinf_No_Andean_1.fq.gz Read2: Tinf_No_Andean_2.fq.gz
Depth
Position

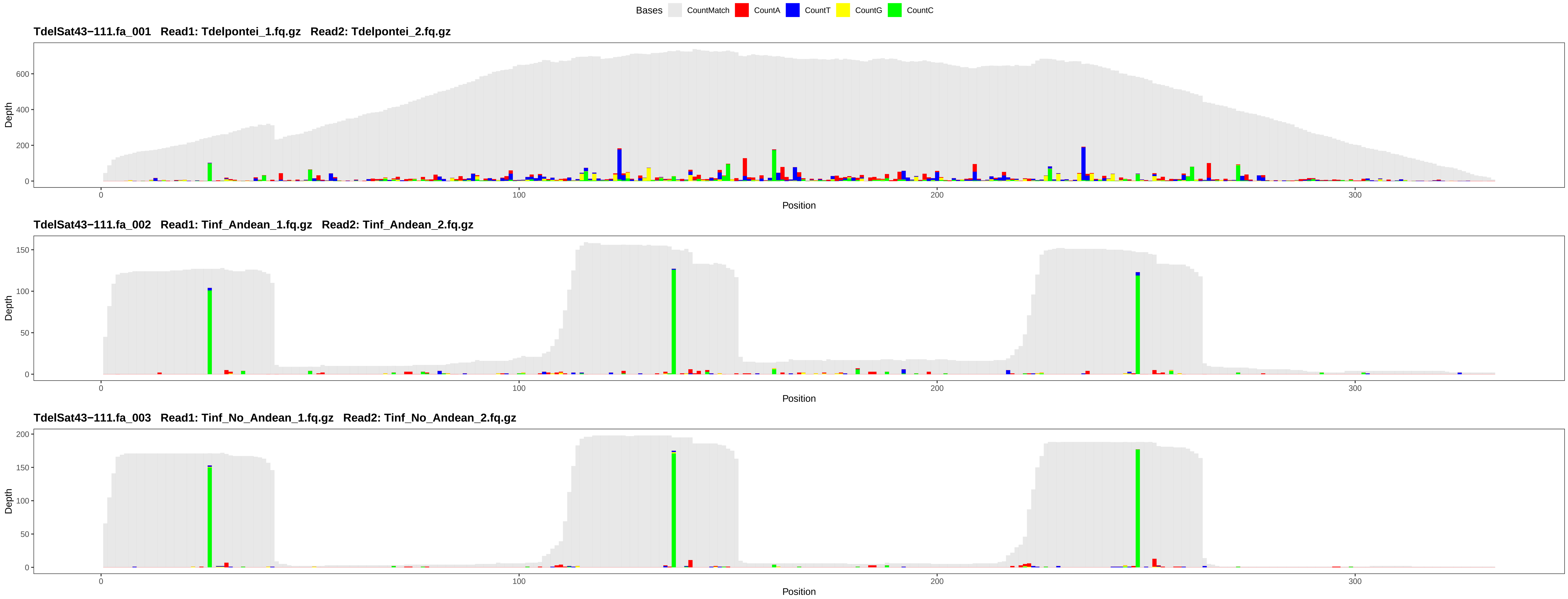
Bases
CountMatch
CountA
CountT
CountG
CountC
TdelSat43−111.fa_001 Read1: Tdelpontei_1.fq.gz Read2: Tdelpontei_2.fq.gz
Depth
Position
TdelSat43−111.fa_002 Read1: Tinf_Andean_1.fq.gz Read2: Tinf_Andean_2.fq.gz
Depth
Position
TdelSat43−111.fa_003 Read1: Tinf_No_Andean_1.fq.gz Read2: Tinf_No_Andean_2.fq.gz
Depth
Position

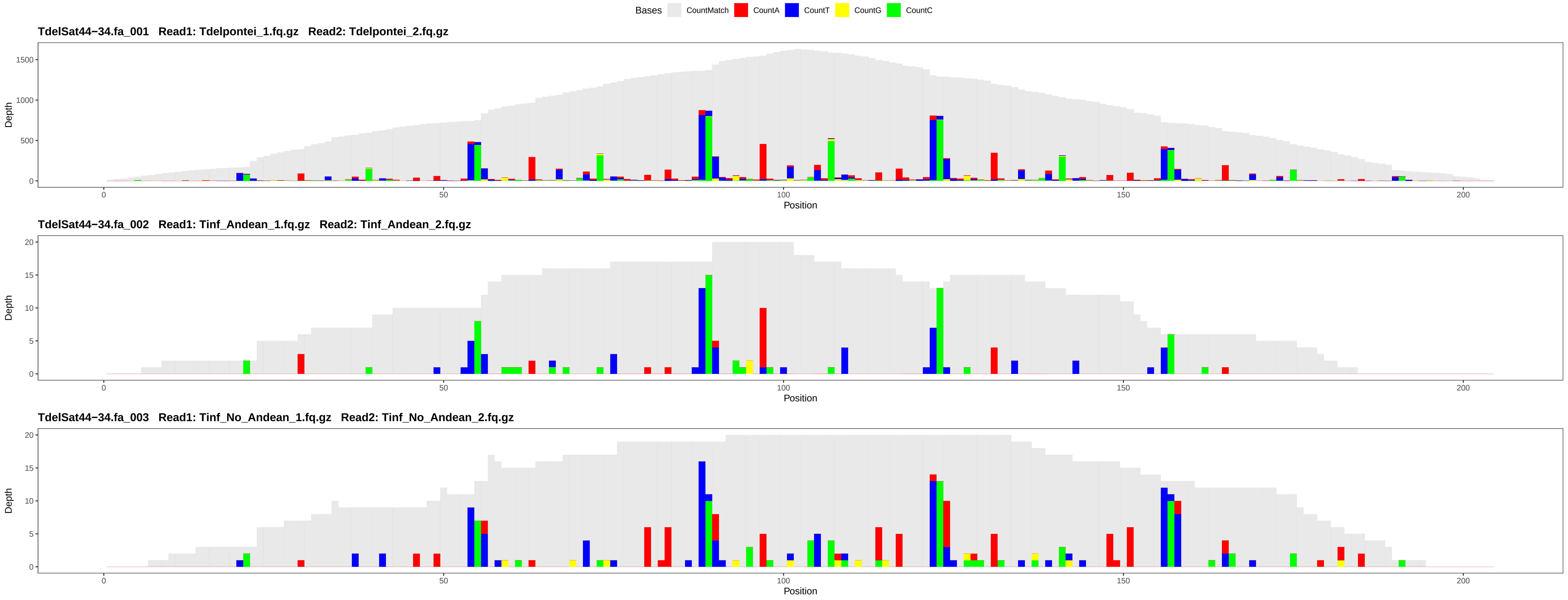

Bases CountMatch CountA CountT CountG CountC
TdelSat44−34.fa_001 Read1: Tdelpontei_1.fq.gz Read2: Tdelpontei_2.fq.gz
Depth
Position
TdelSat44−34.fa_002 Read1: Tinf_Andean_1.fq.gz Read2: Tinf_Andean_2.fq.gz
Depth
Position
TdelSat44−34.fa_003 Read1: Tinf_No_Andean_1.fq.gz Read2: Tinf_No_Andean_2.fq.gz
Depth
Position

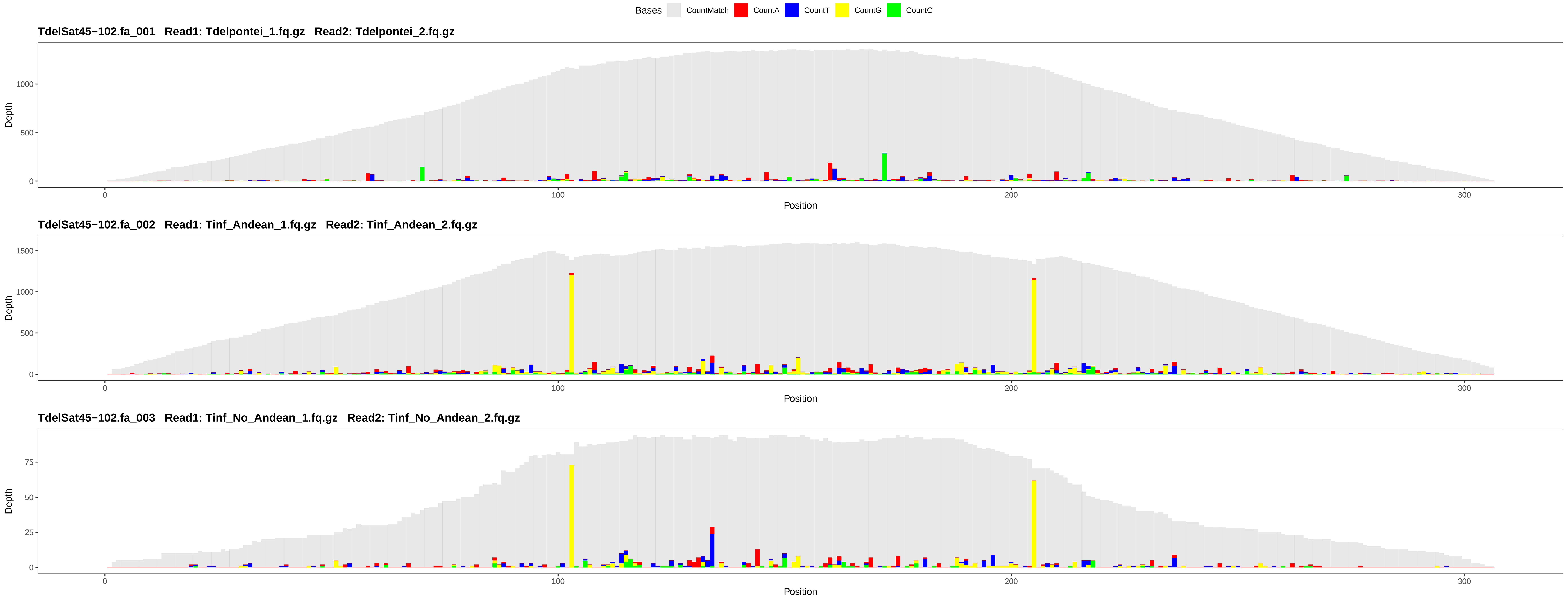
Bases CountMatch CountA CountT CountG CountC
TdelSat45−102.fa_001 Read1: Tdelpontei_1.fq.gz Read2: Tdelpontei_2.fq.gz
Depth
0
500
1000
Position
0
100
200
300
TdelSat45−102.fa_002 Read1: Tinf_Andean_1.fq.gz Read2: Tinf_Andean_2.fq.gz
Depth
0
500
1000
1500
Position
0
100
200
300
TdelSat45−102.fa_003 Read1: Tinf_No_Andean_1.fq.gz Read2: Tinf_No_Andean_2.fq.gz
Depth
0
25
50
75
Position
0
100
200
300

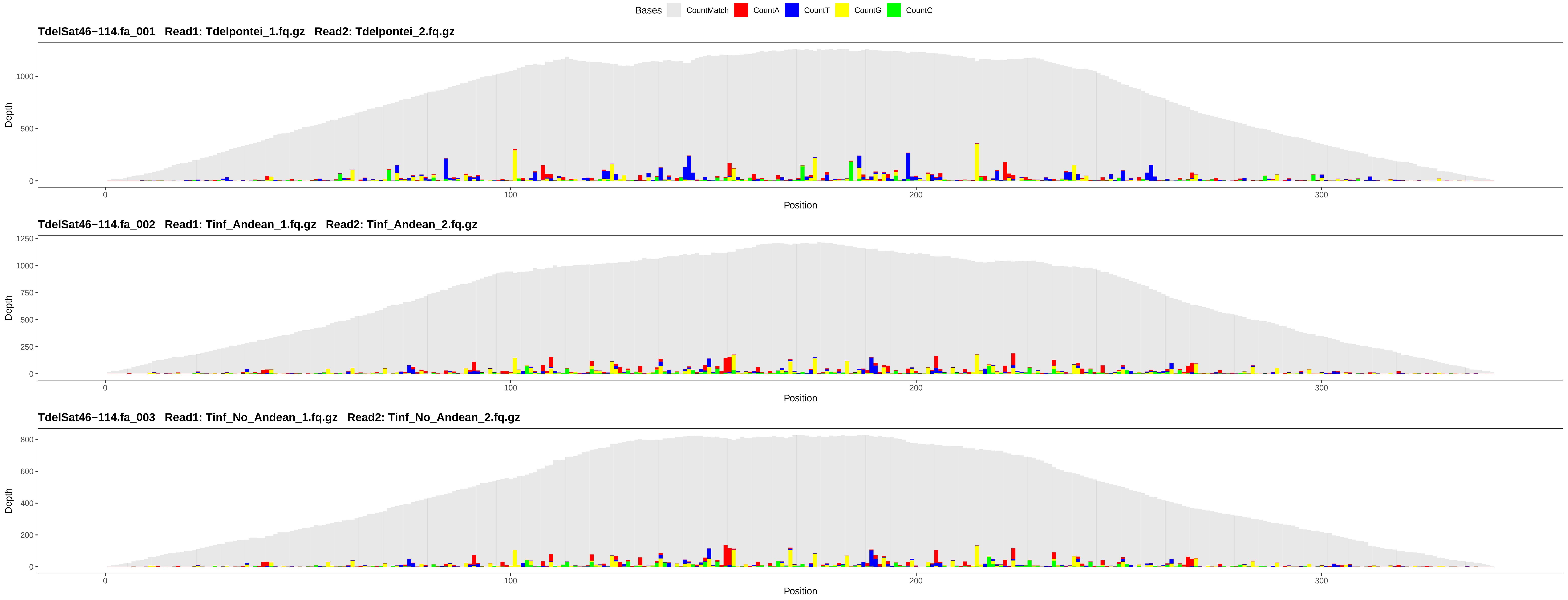
Bases CountMatch CountA CountT CountG CountC
TdelSat46−114.fa_001 Read1: Tdelpontei_1.fq.gz Read2: Tdelpontei_2.fq.gz
Depth
Position
TdelSat46−114.fa_002 Read1: Tinf_Andean_1.fq.gz Read2: Tinf_Andean_2.fq.gz
Depth
Position
TdelSat46−114.fa_003 Read1: Tinf_No_Andean_1.fq.gz Read2: Tinf_No_Andean_2.fq.gz
Depth
Position

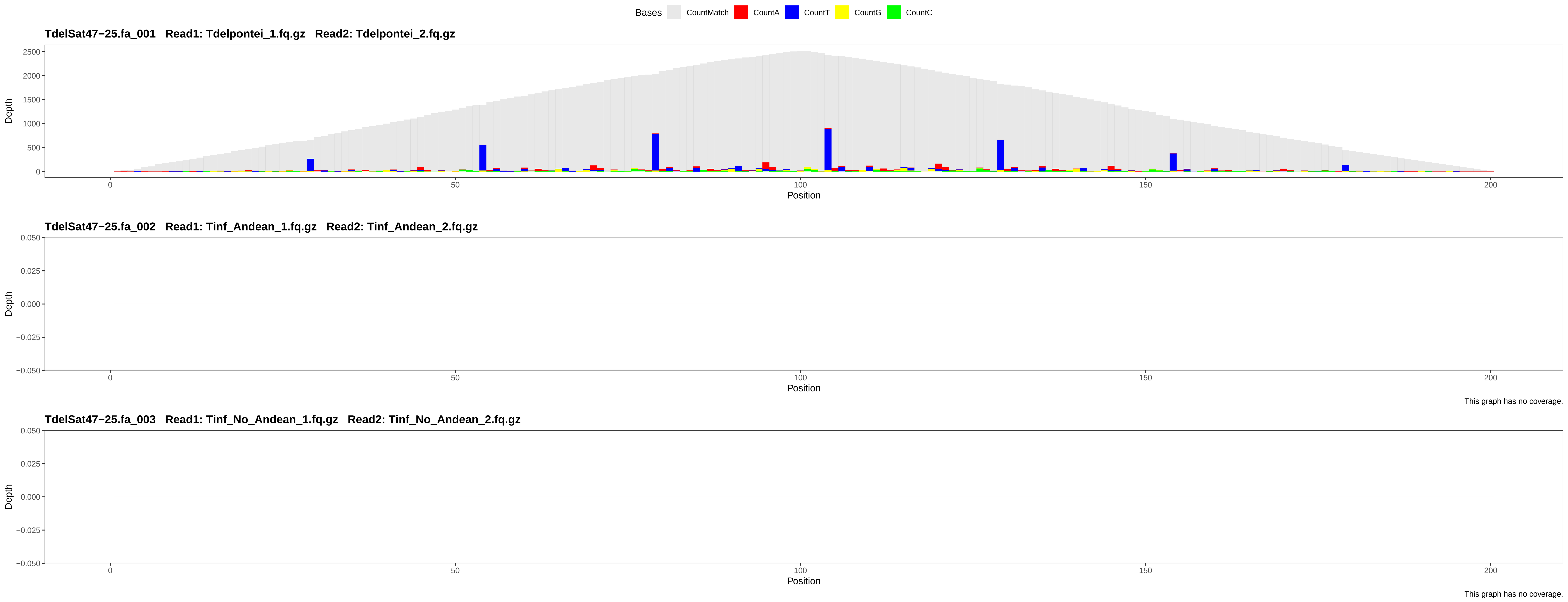
Bases CountMatch CountA CountT CountG CountC
TdelSat47−25.fa_001 Read1: Tdelpontei_1.fq.gz Read2: Tdelpontei_2.fq.gz
Depth
2500
2000
1500
1000
500
0
0
50
100
150
200
Position
TdelSat47−25.fa_002 Read1: Tinf_Andean_1.fq.gz Read2: Tinf_Andean_2.fq.gz
Depth
0.050
0.025
0.000
−0.025
−0.050
0
50
100
150
200
Position
This graph has no coverage.
TdelSat47−25.fa_003 Read1: Tinf_No_Andean_1.fq.gz Read2: Tinf_No_Andean_2.fq.gz
Depth
0.050
0.025
0.000
−0.025
−0.050
0
50
100
150
200
Position
This graph has no coverage.

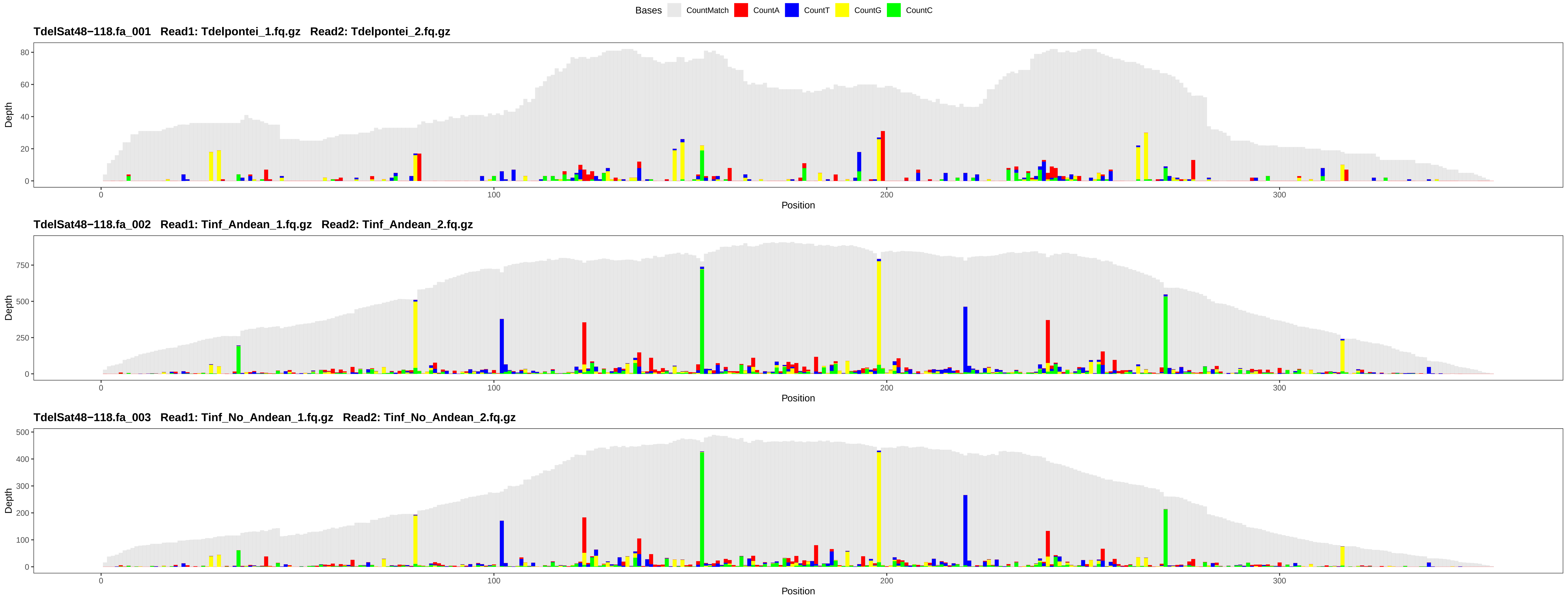
Bases
CountMatch
CountA
CountT
CountG
CountC
TdelSat48−118.fa_001   Read1: Tdelpontei_1.fq.gz   Read2: Tdelpontei_2.fq.gz
Depth
80
60
40
20
0
0
100
200
300
Position
TdelSat48−118.fa_002   Read1: Tinf_Andean_1.fq.gz   Read2: Tinf_Andean_2.fq.gz
Depth
750
500
250
0
0
100
200
300
Position
TdelSat48−118.fa_003   Read1: Tinf_No_Andean_1.fq.gz   Read2: Tinf_No_Andean_2.fq.gz
Depth
500
400
300
200
100
0
0
100
200
300
Position

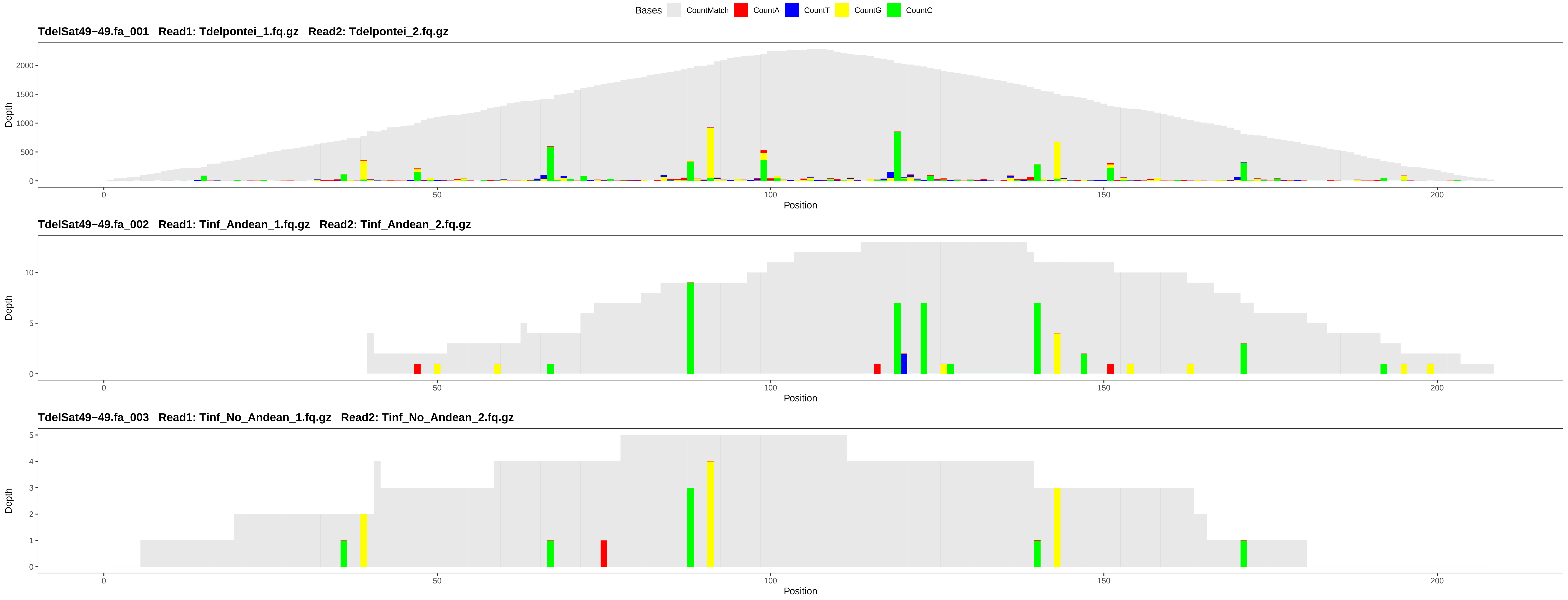

Bases
CountMatch
CountA
CountT
CountG
CountC
TdelSat49−49.fa_001 Read1: Tdelpontei_1.fq.gz Read2: Tdelpontei_2.fq.gz
Depth
Position
TdelSat49−49.fa_002 Read1: Tinf_Andean_1.fq.gz Read2: Tinf_Andean_2.fq.gz
Depth
Position
TdelSat49−49.fa_003 Read1: Tinf_No_Andean_1.fq.gz Read2: Tinf_No_Andean_2.fq.gz
Depth
Position

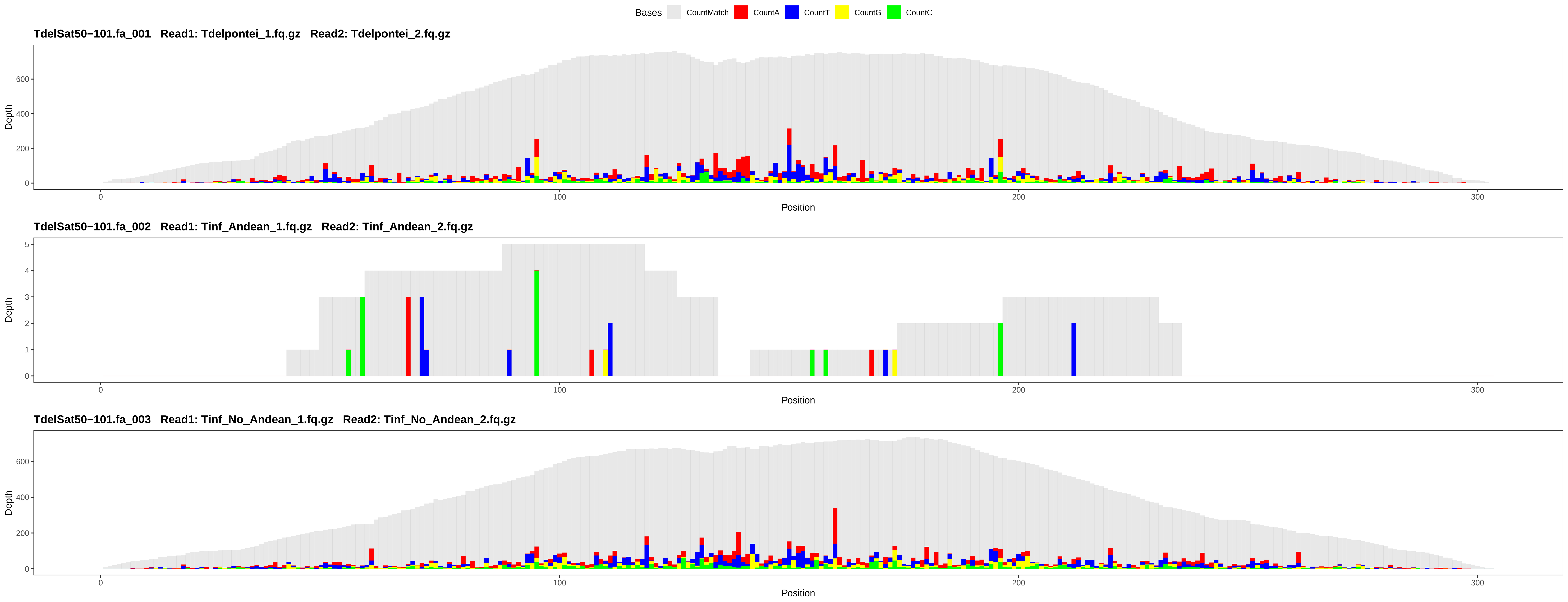

Bases
CountMatch
CountA
CountT
CountG
CountC
TdelSat50−101.fa_001 Read1: Tdelpontei_1.fq.gz Read2: Tdelpontei_2.fq.gz
Depth
0
200
400
600
Position
0
100
200
300
TdelSat50−101.fa_002 Read1: Tinf_Andean_1.fq.gz Read2: Tinf_Andean_2.fq.gz
Depth
0
1
2
3
4
5
Position
0
100
200
300
TdelSat50−101.fa_003 Read1: Tinf_No_Andean_1.fq.gz Read2: Tinf_No_Andean_2.fq.gz
Depth
0
200
400
600
Position
0
100
200
300

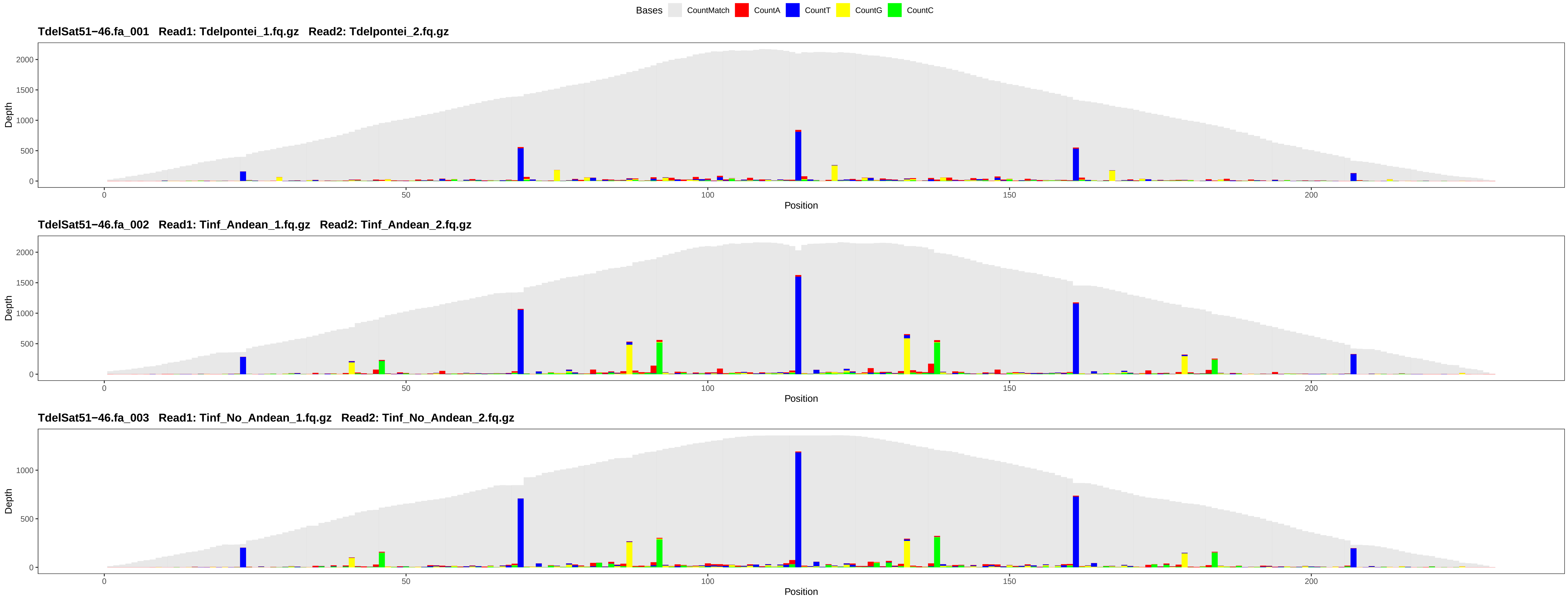
Bases CountMatch CountA CountT CountG CountC
TdelSat51−46.fa_001 Read1: Tdelpontei_1.fq.gz Read2: Tdelpontei_2.fq.gz
Depth
0
500
1000
1500
2000
0
50
100
150
200
Position
TdelSat51−46.fa_002 Read1: Tinf_Andean_1.fq.gz Read2: Tinf_Andean_2.fq.gz
Depth
0
500
1000
1500
2000
0
50
100
150
200
Position
TdelSat51−46.fa_003 Read1: Tinf_No_Andean_1.fq.gz Read2: Tinf_No_Andean_2.fq.gz
Depth
0
500
1000
0
50
100
150
200
Position

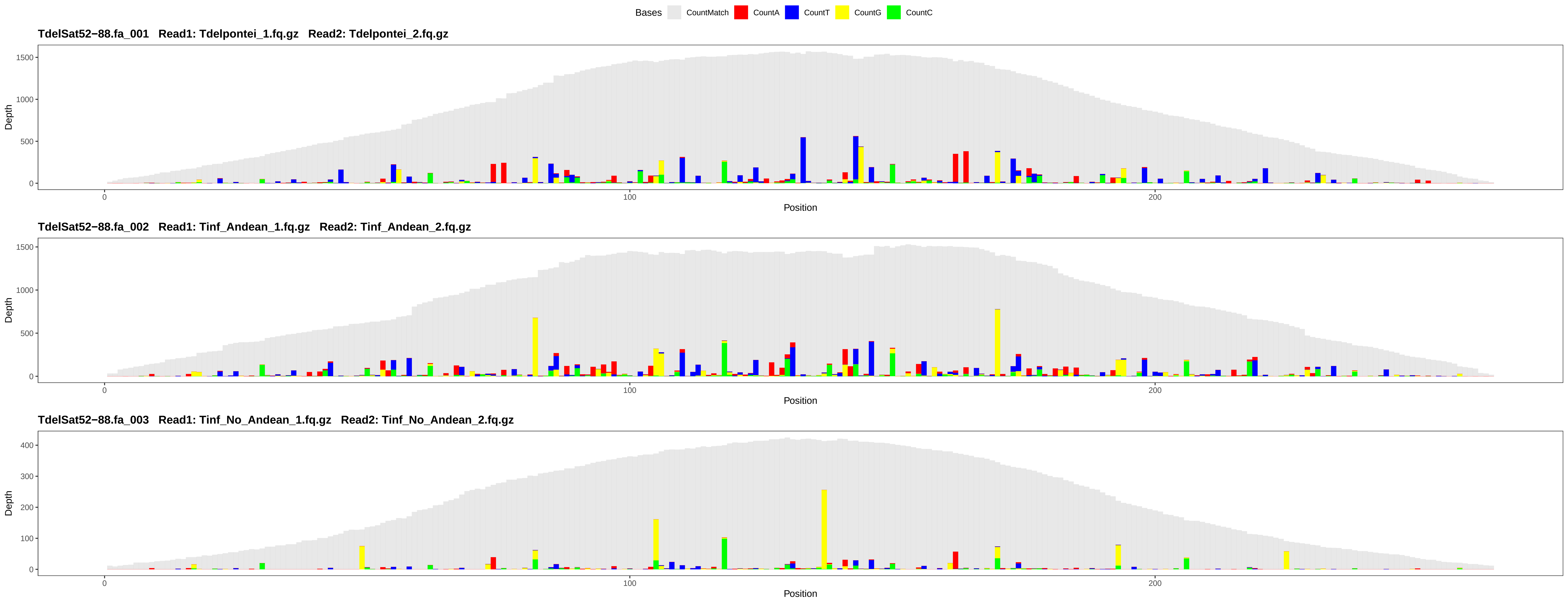
Bases
CountMatch
CountA
CountT
CountG
CountC
TdelSat52−88.fa_001 Read1: Tdelpontei_1.fq.gz Read2: Tdelpontei_2.fq.gz
Depth
0
500
1000
1500
Position
0
100
200
TdelSat52−88.fa_002 Read1: Tinf_Andean_1.fq.gz Read2: Tinf_Andean_2.fq.gz
Depth
0
500
1000
1500
Position
0
100
200
TdelSat52−88.fa_003 Read1: Tinf_No_Andean_1.fq.gz Read2: Tinf_No_Andean_2.fq.gz
Depth
0
100
200
300
400
Position
0
100
200

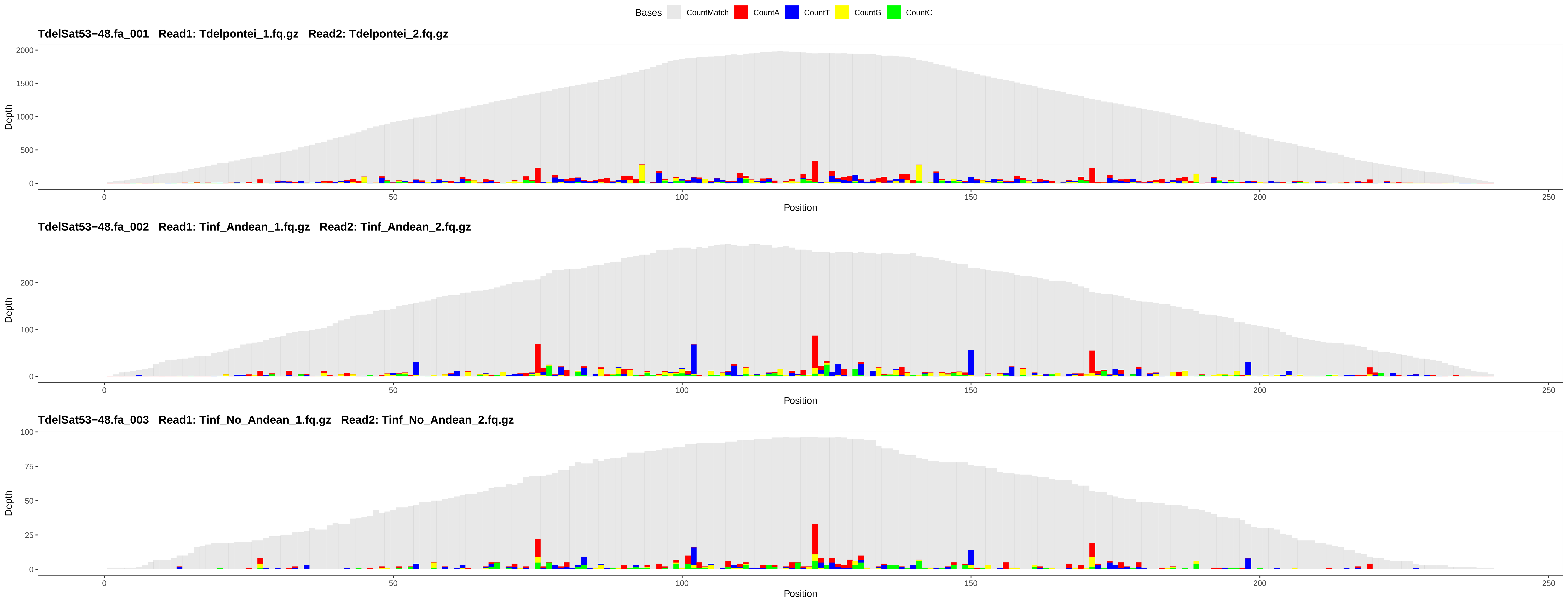
Bases CountMatch CountA CountT CountG CountC
TdelSat53−48.fa_001 Read1: Tdelpontei_1.fq.gz Read2: Tdelpontei_2.fq.gz
Depth
Position
TdelSat53−48.fa_002 Read1: Tinf_Andean_1.fq.gz Read2: Tinf_Andean_2.fq.gz
Depth
Position
TdelSat53−48.fa_003 Read1: Tinf_No_Andean_1.fq.gz Read2: Tinf_No_Andean_2.fq.gz
Depth
Position

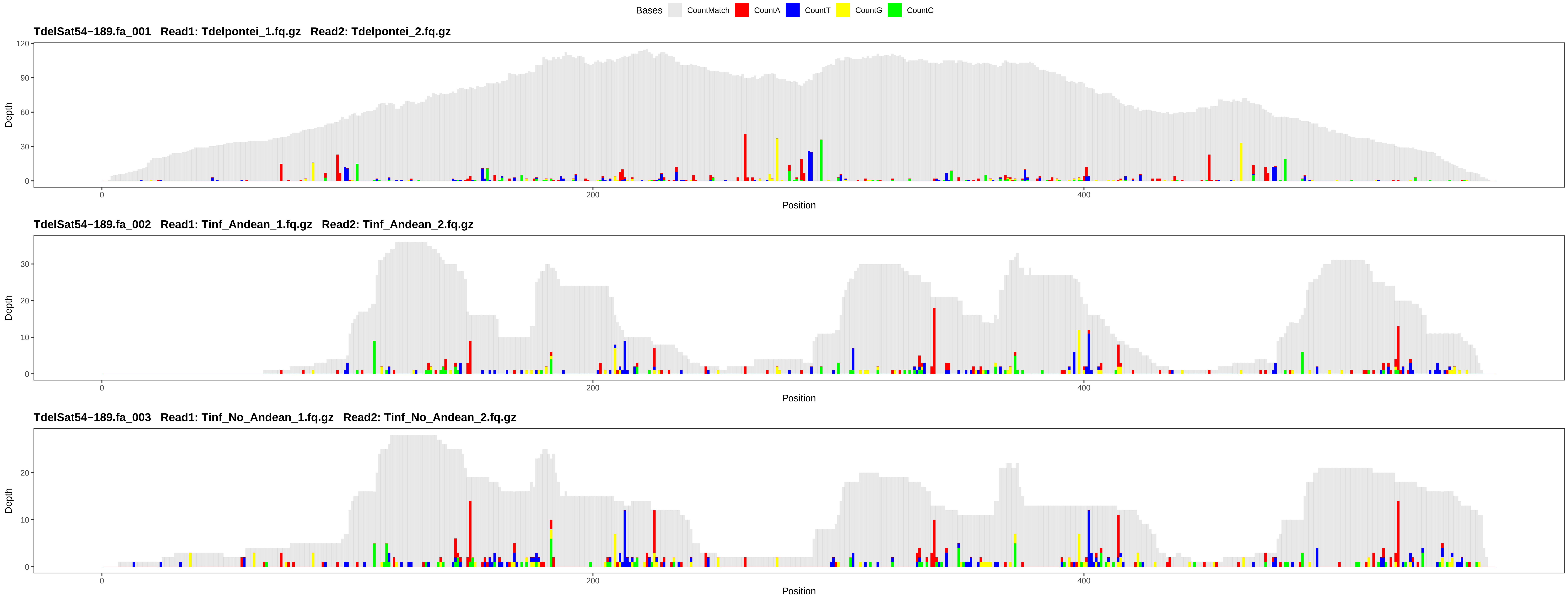

Bases
CountMatch
CountA
CountT
CountG
CountC
TdelSat54−189.fa_001   Read1: Tdelpontei_1.fq.gz   Read2: Tdelpontei_2.fq.gz
Depth
Position
TdelSat54−189.fa_002   Read1: Tinf_Andean_1.fq.gz   Read2: Tinf_Andean_2.fq.gz
Depth
Position
TdelSat54−189.fa_003   Read1: Tinf_No_Andean_1.fq.gz   Read2: Tinf_No_Andean_2.fq.gz
Depth
Position

Bases: CountMatch, CountA, CountT, CountG, CountC

TdelSat55−51.fa_001   Read1: Tdelpontei_1.fq.gz   Read2: Tdelpontei_2.fq.gz

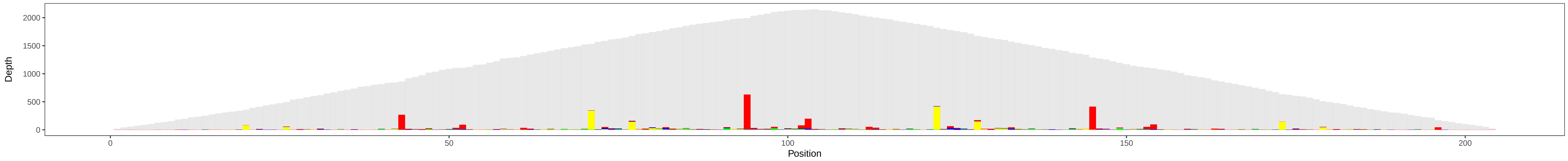

TdelSat55−51.fa_002   Read1: Tinf_Andean_1.fq.gz   Read2: Tinf_Andean_2.fq.gz

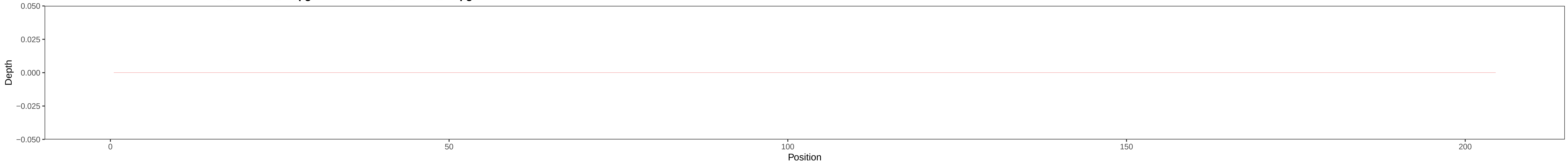

This graph has no coverage.

TdelSat55−51.fa_003   Read1: Tinf_No_Andean_1.fq.gz   Read2: Tinf_No_Andean_2.fq.gz

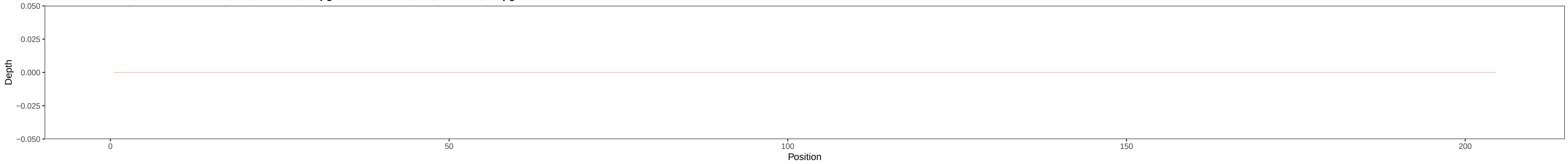

This graph has no coverage.

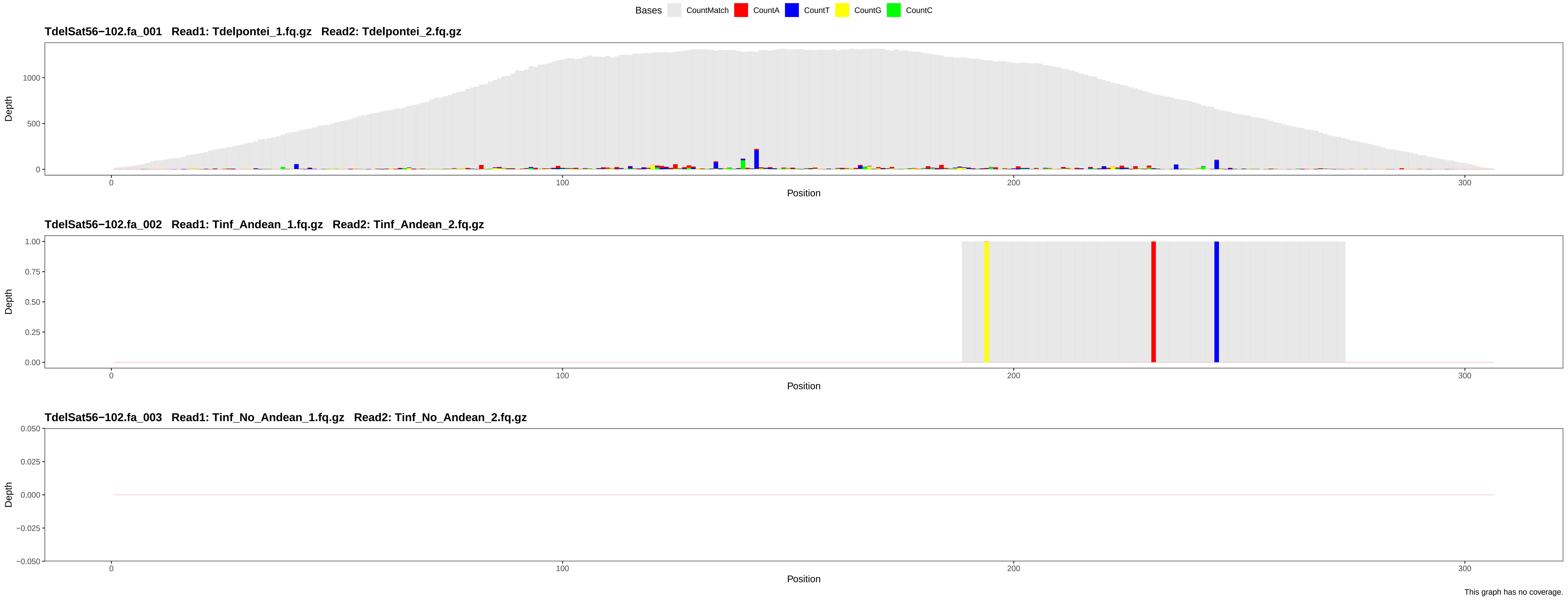
Bases CountMatch CountA CountT CountG CountC
TdelSat56−102.fa_001 Read1: Tdelpontei_1.fq.gz Read2: Tdelpontei_2.fq.gz
Depth
1000
500
0
0
100
200
300
Position
TdelSat56−102.fa_002 Read1: Tinf_Andean_1.fq.gz Read2: Tinf_Andean_2.fq.gz
Depth
1.00
0.75
0.50
0.25
0.00
0
100
200
300
Position
TdelSat56−102.fa_003 Read1: Tinf_No_Andean_1.fq.gz Read2: Tinf_No_Andean_2.fq.gz
Depth
0.050
0.025
0.000
−0.025
−0.050
0
100
200
300
Position
This graph has no coverage.

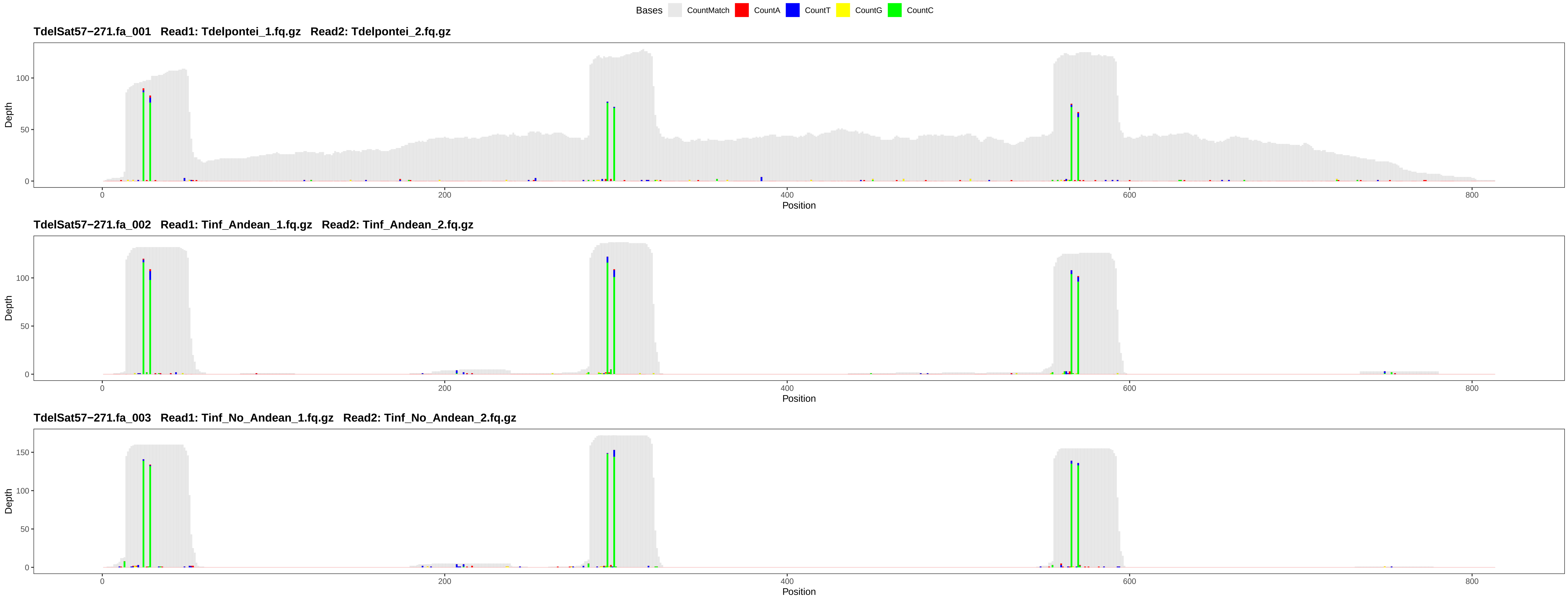

Bases
CountMatch
CountA
CountT
CountG
CountC
TdelSat57−271.fa_001 Read1: Tdelpontei_1.fq.gz Read2: Tdelpontei_2.fq.gz
Depth
0
50
100
0
200
400
600
800
Position
TdelSat57−271.fa_002 Read1: Tinf_Andean_1.fq.gz Read2: Tinf_Andean_2.fq.gz
Depth
0
50
100
0
200
400
600
800
Position
TdelSat57−271.fa_003 Read1: Tinf_No_Andean_1.fq.gz Read2: Tinf_No_Andean_2.fq.gz
Depth
0
50
100
150
0
200
400
600
800
Position

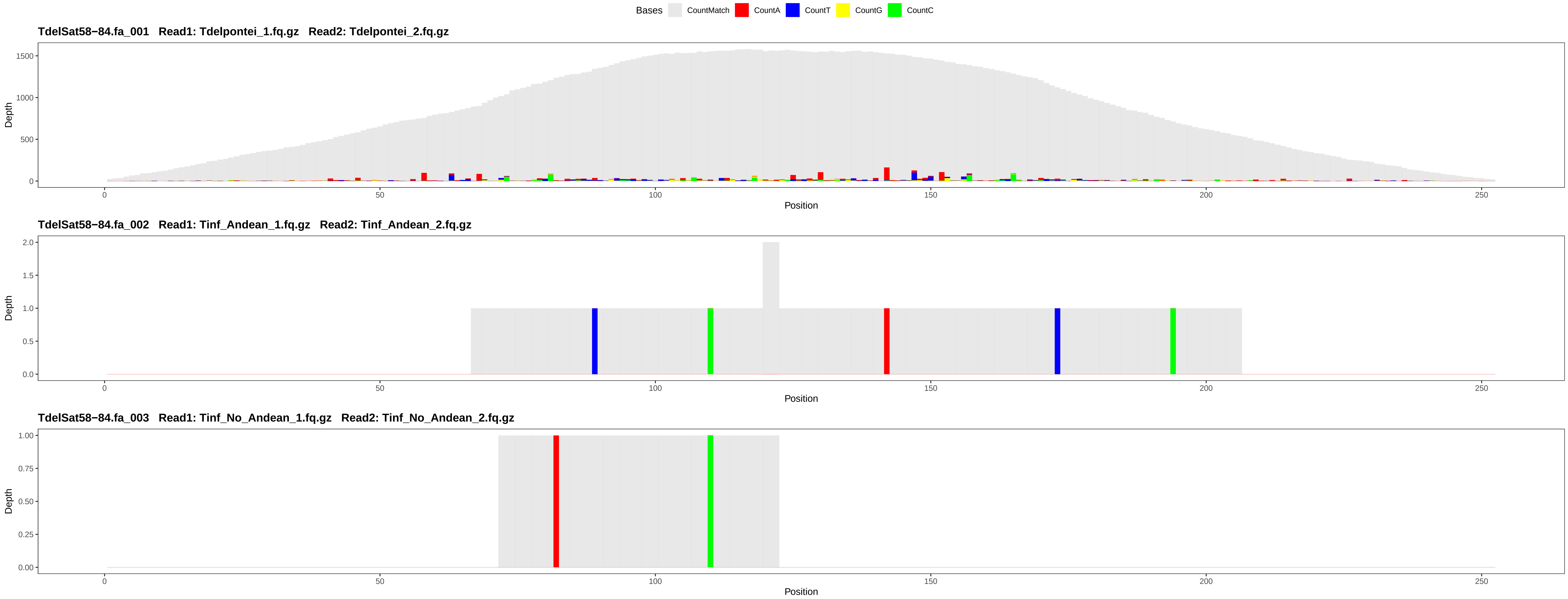

Bases
CountMatch
CountA
CountT
CountG
CountC
TdelSat58−84.fa_001 Read1: Tdelpontei_1.fq.gz Read2: Tdelpontei_2.fq.gz
Depth
0
500
1000
1500
0
50
100
150
200
250
Position
TdelSat58−84.fa_002 Read1: Tinf_Andean_1.fq.gz Read2: Tinf_Andean_2.fq.gz
Depth
0.0
0.5
1.0
1.5
2.0
0
50
100
150
200
250
Position
TdelSat58−84.fa_003 Read1: Tinf_No_Andean_1.fq.gz Read2: Tinf_No_Andean_2.fq.gz
Depth
0.00
0.25
0.50
0.75
1.00
0
50
100
150
200
250
Position

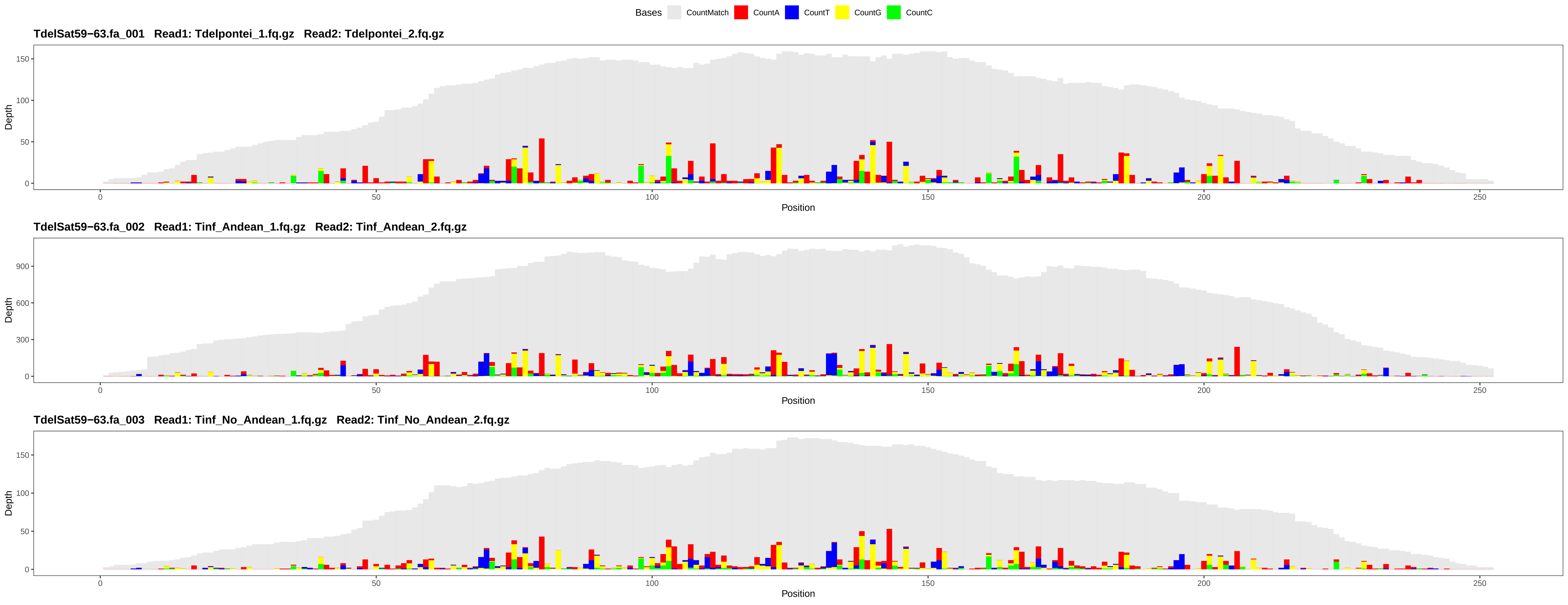

Bases
CountMatch
CountA
CountT
CountG
CountC
TdelSat59−63.fa_001 Read1: Tdelpontei_1.fq.gz Read2: Tdelpontei_2.fq.gz
Depth
Position
TdelSat59−63.fa_002 Read1: Tinf_Andean_1.fq.gz Read2: Tinf_Andean_2.fq.gz
Depth
Position
TdelSat59−63.fa_003 Read1: Tinf_No_Andean_1.fq.gz Read2: Tinf_No_Andean_2.fq.gz
Depth
Position

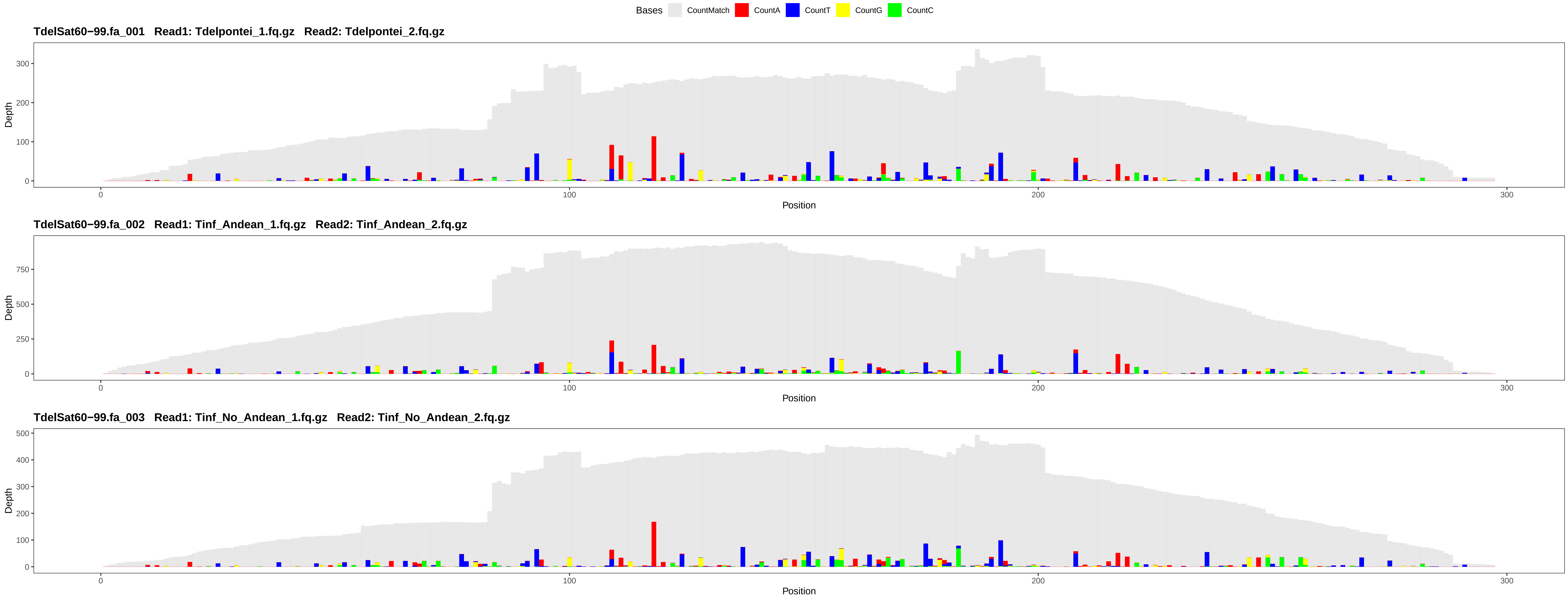
Bases
CountMatch
CountA
CountT
CountG
CountC
TdelSat60−99.fa_001 Read1: Tdelpontei_1.fq.gz Read2: Tdelpontei_2.fq.gz
Depth
Position
TdelSat60−99.fa_002 Read1: Tinf_Andean_1.fq.gz Read2: Tinf_Andean_2.fq.gz
Depth
Position
TdelSat60−99.fa_003 Read1: Tinf_No_Andean_1.fq.gz Read2: Tinf_No_Andean_2.fq.gz
Depth
Position

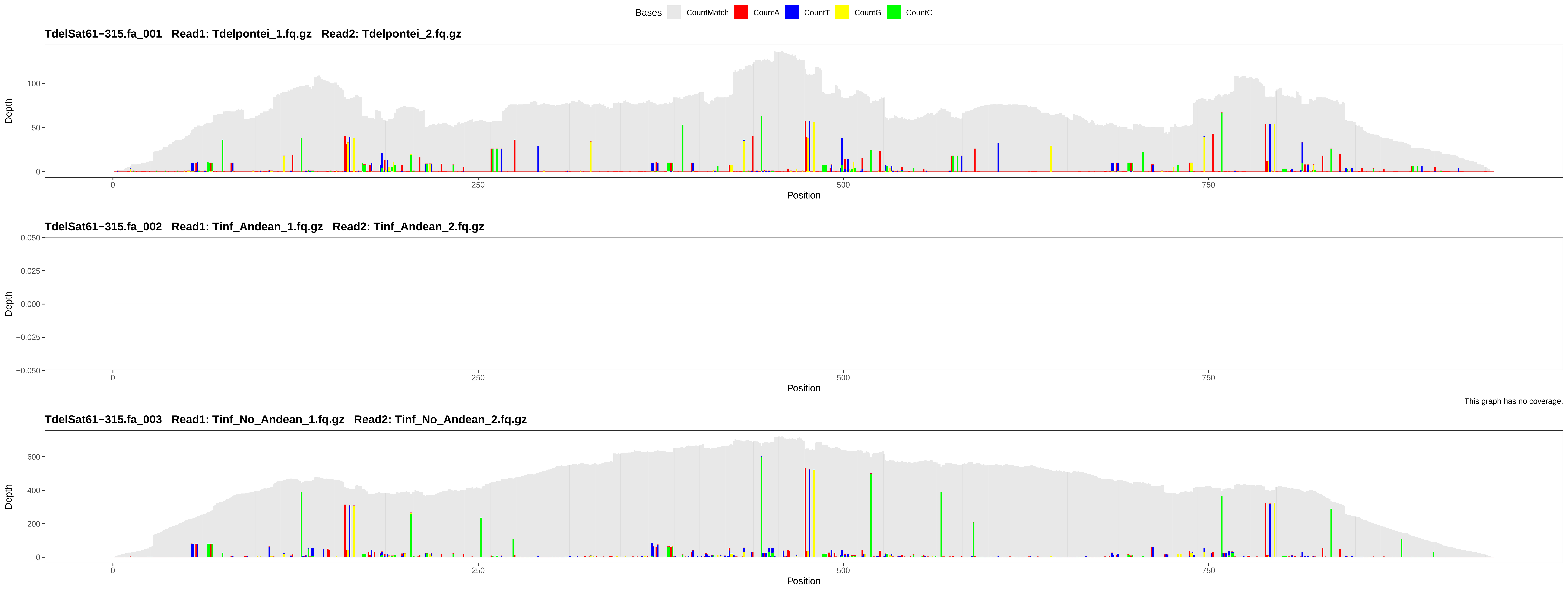
Bases
CountMatch
CountA
CountT
CountG
CountC
TdelSat61−315.fa_001 Read1: Tdelpontei_1.fq.gz Read2: Tdelpontei_2.fq.gz
Depth
100
50
0
0
250
500
750
Position
TdelSat61−315.fa_002 Read1: Tinf_Andean_1.fq.gz Read2: Tinf_Andean_2.fq.gz
Depth
0.050
0.025
0.000
−0.025
−0.050
0
250
500
750
Position
This graph has no coverage.
TdelSat61−315.fa_003 Read1: Tinf_No_Andean_1.fq.gz Read2: Tinf_No_Andean_2.fq.gz
Depth
600
400
200
0
0
250
500
750
Position

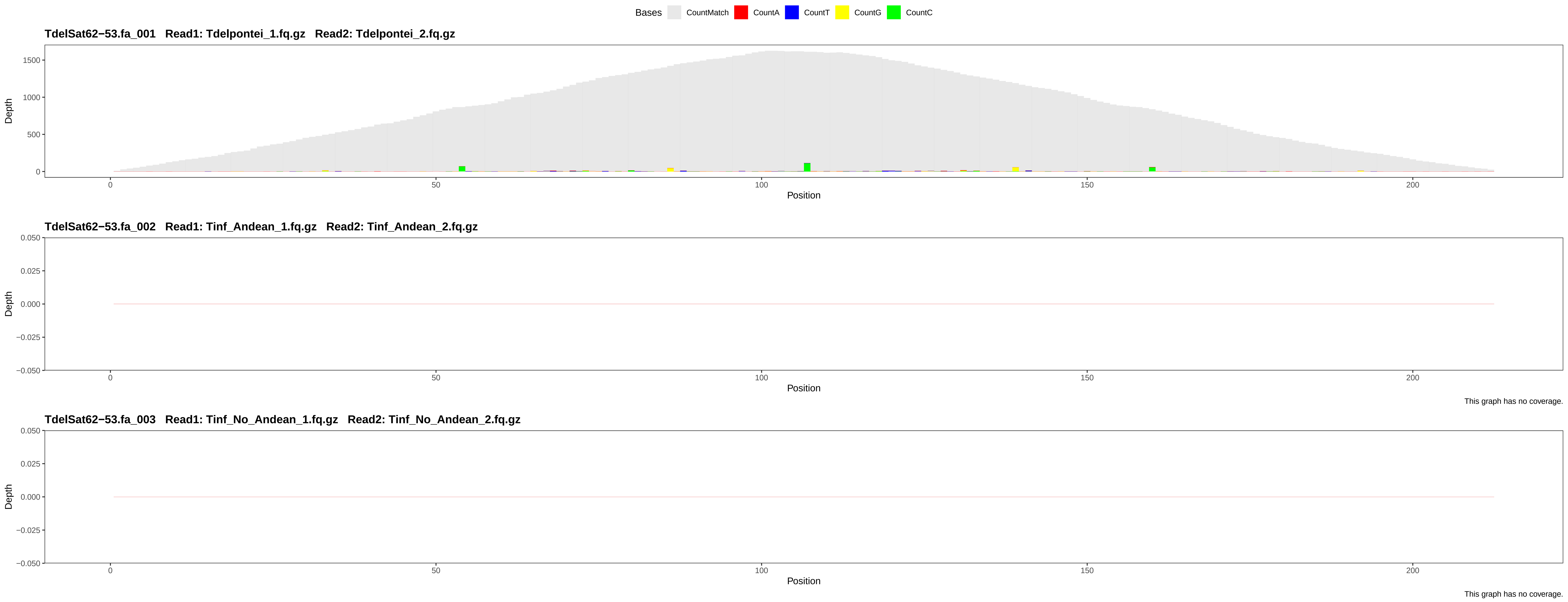

Bases CountMatch CountA CountT CountG CountC
TdelSat62−53.fa_001 Read1: Tdelpontei_1.fq.gz Read2: Tdelpontei_2.fq.gz
Depth
Position
TdelSat62−53.fa_002 Read1: Tinf_Andean_1.fq.gz Read2: Tinf_Andean_2.fq.gz
Depth
Position
This graph has no coverage.
TdelSat62−53.fa_003 Read1: Tinf_No_Andean_1.fq.gz Read2: Tinf_No_Andean_2.fq.gz
Depth
Position
This graph has no coverage.

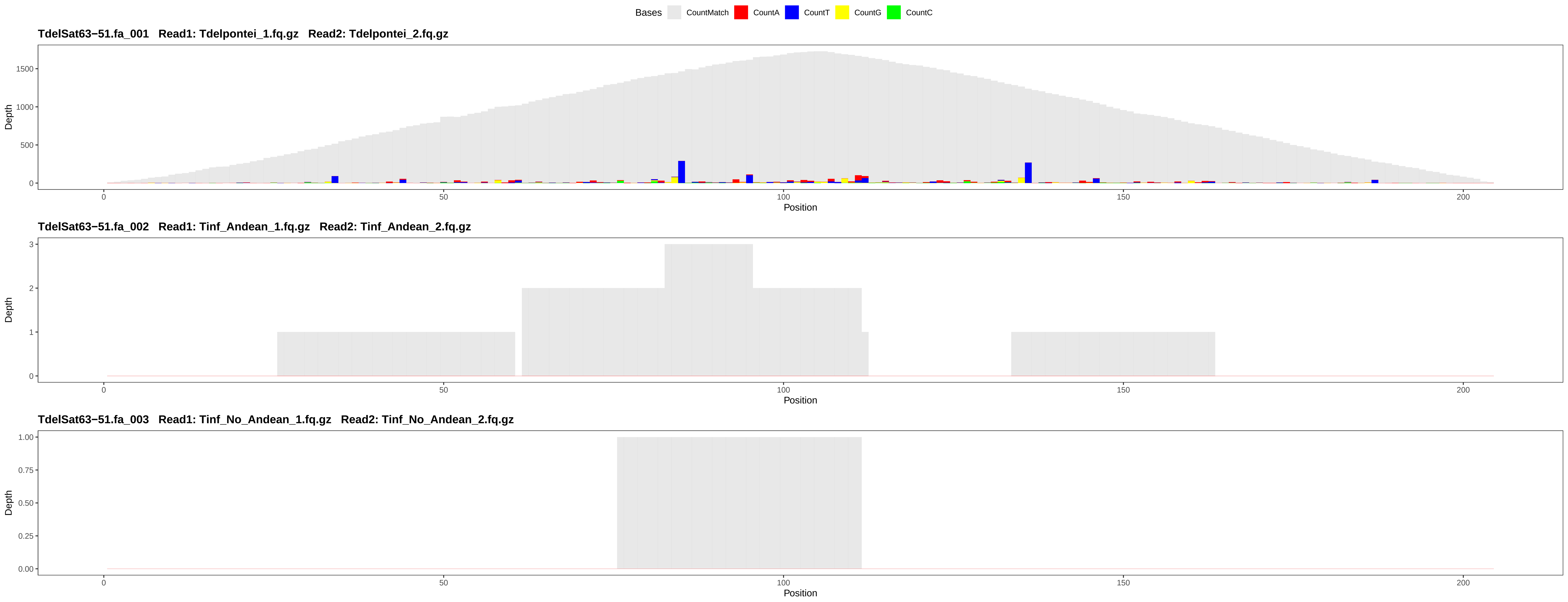

Bases
CountMatch
CountA
CountT
CountG
CountC
TdelSat63−51.fa_001 Read1: Tdelpontei_1.fq.gz Read2: Tdelpontei_2.fq.gz
Depth
0
500
1000
1500
0
50
100
150
200
Position
TdelSat63−51.fa_002 Read1: Tinf_Andean_1.fq.gz Read2: Tinf_Andean_2.fq.gz
Depth
0
1
2
3
0
50
100
150
200
Position
TdelSat63−51.fa_003 Read1: Tinf_No_Andean_1.fq.gz Read2: Tinf_No_Andean_2.fq.gz
Depth
0.00
0.25
0.50
0.75
1.00
0
50
100
150
200
Position

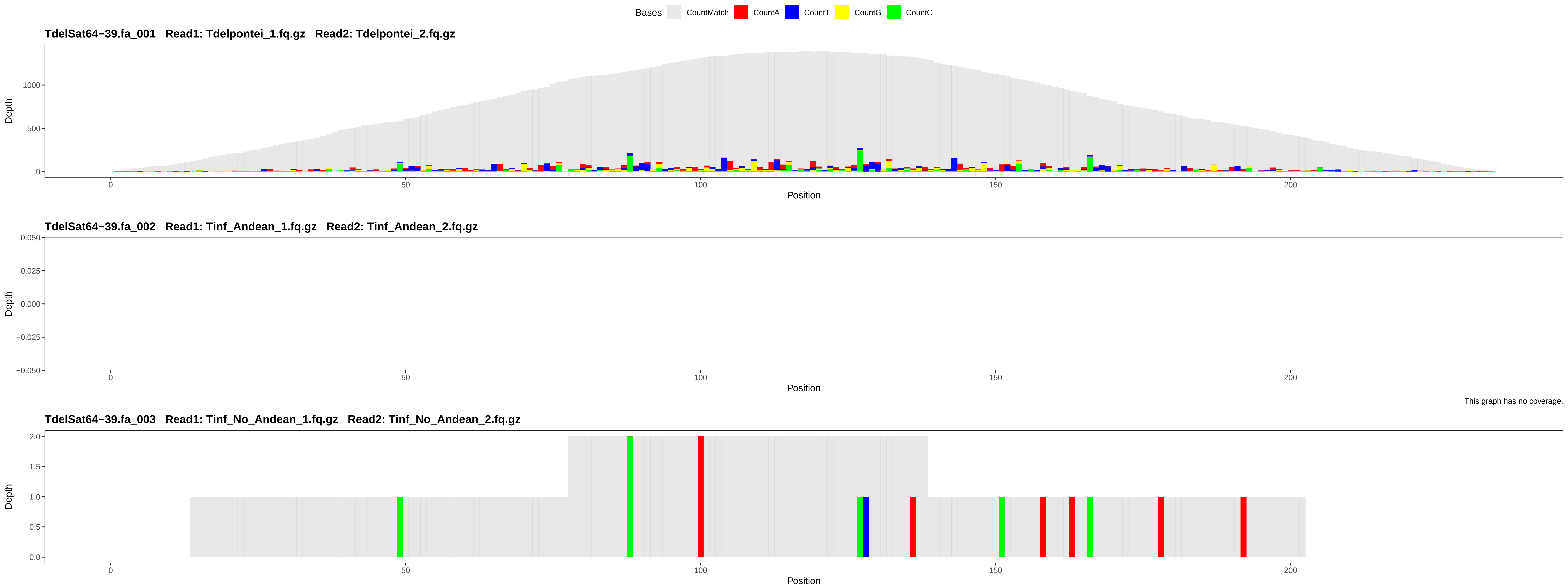
Bases CountMatch CountA CountT CountG CountC
TdelSat64−39.fa_001 Read1: Tdelpontei_1.fq.gz Read2: Tdelpontei_2.fq.gz
Depth
Position
TdelSat64−39.fa_002 Read1: Tinf_Andean_1.fq.gz Read2: Tinf_Andean_2.fq.gz
This graph has no coverage.
TdelSat64−39.fa_003 Read1: Tinf_No_Andean_1.fq.gz Read2: Tinf_No_Andean_2.fq.gz

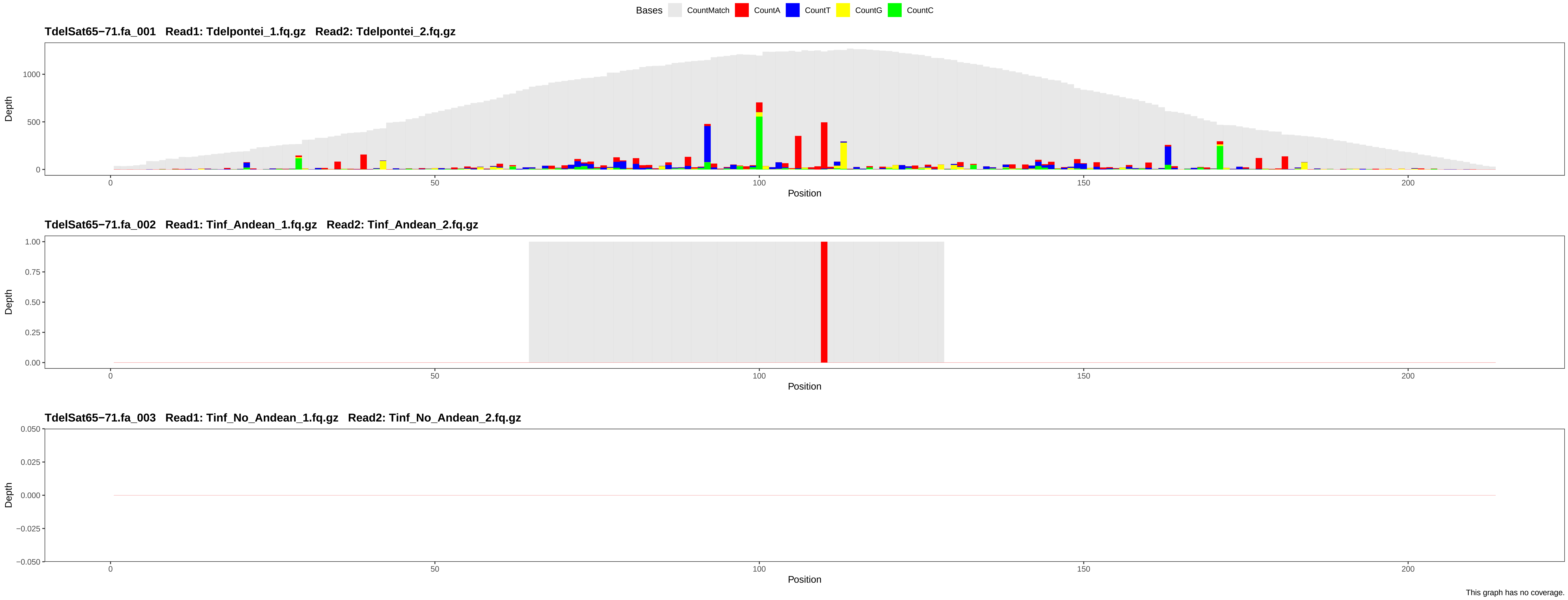
Bases
CountMatch
CountA
CountT
CountG
CountC
TdelSat65−71.fa_001 Read1: Tdelpontei_1.fq.gz Read2: Tdelpontei_2.fq.gz
Depth
Position
TdelSat65−71.fa_002 Read1: Tinf_Andean_1.fq.gz Read2: Tinf_Andean_2.fq.gz
Depth
Position
TdelSat65−71.fa_003 Read1: Tinf_No_Andean_1.fq.gz Read2: Tinf_No_Andean_2.fq.gz
Depth
Position
This graph has no coverage.

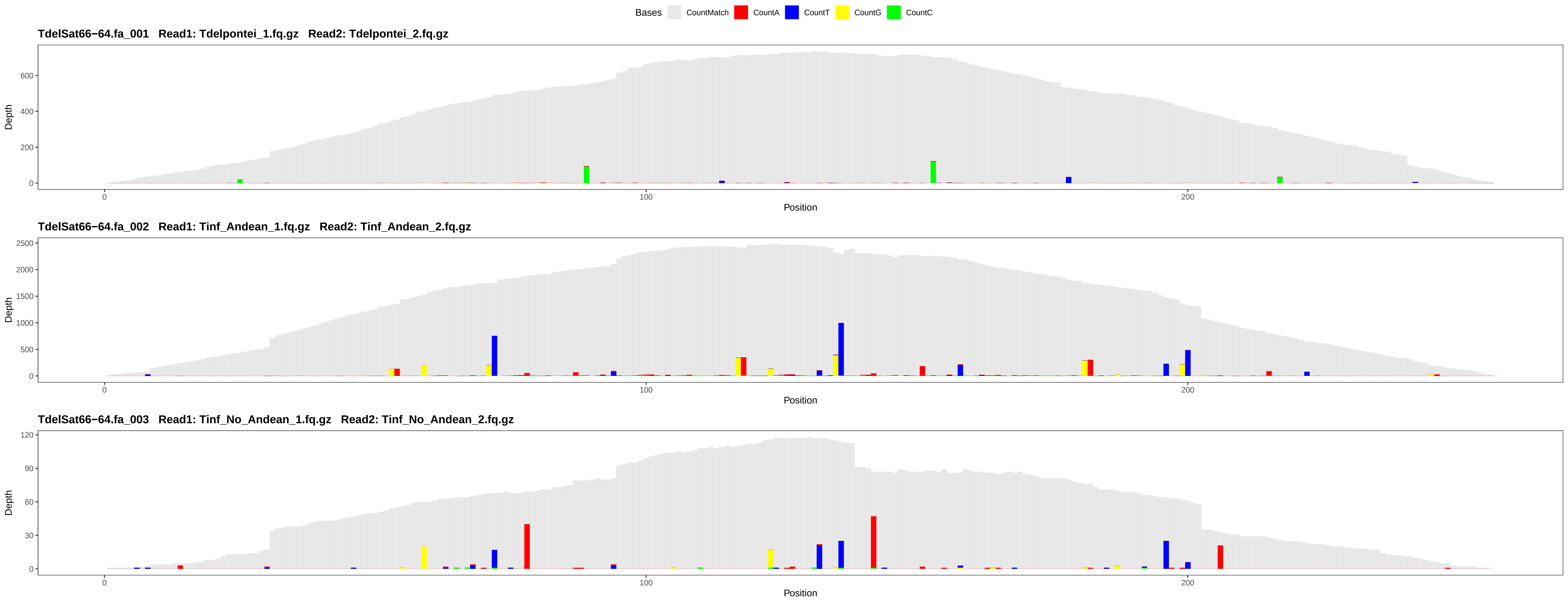
Bases CountMatch CountA CountT CountG CountC
TdelSat66−64.fa_001 Read1: Tdelpontei_1.fq.gz Read2: Tdelpontei_2.fq.gz
Depth
0
200
400
600
Position
0
100
200
TdelSat66−64.fa_002 Read1: Tinf_Andean_1.fq.gz Read2: Tinf_Andean_2.fq.gz
Depth
0
500
1000
1500
2000
2500
Position
0
100
200
TdelSat66−64.fa_003 Read1: Tinf_No_Andean_1.fq.gz Read2: Tinf_No_Andean_2.fq.gz
Depth
0
30
60
90
120
Position
0
100
200

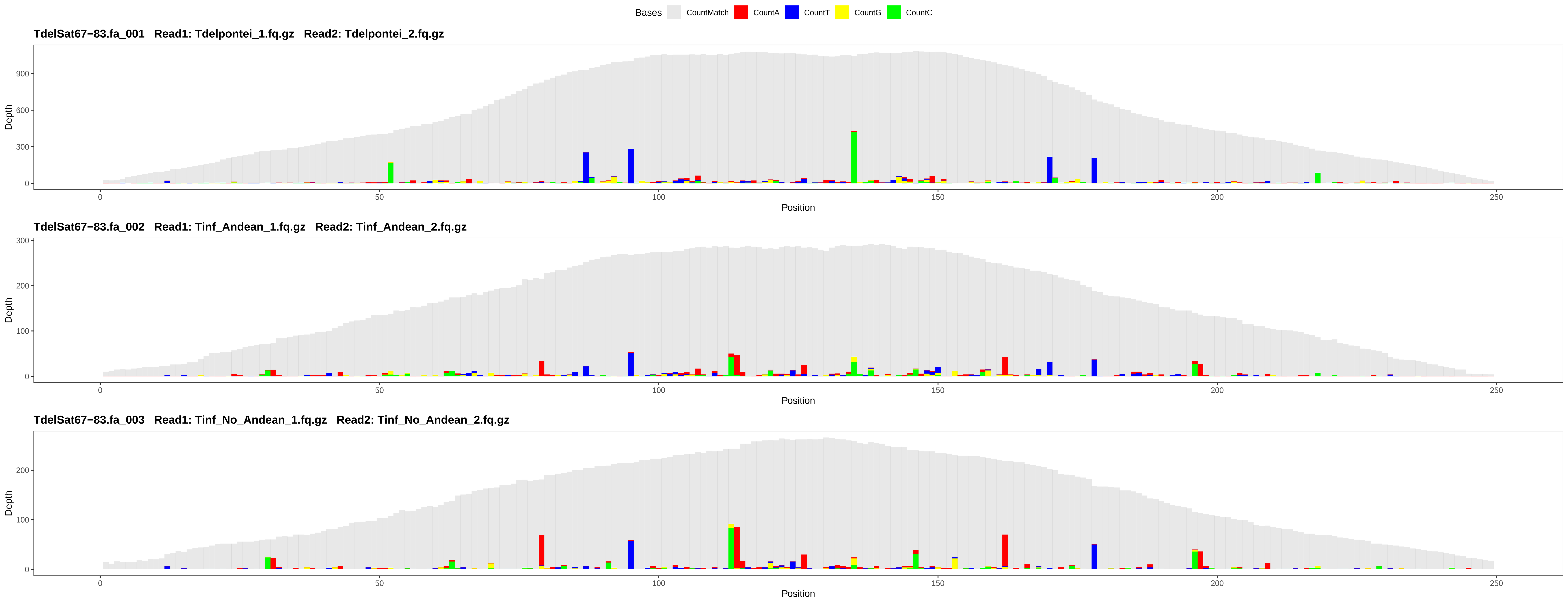
Bases CountMatch CountA CountT CountG CountC
TdelSat67−83.fa_001 Read1: Tdelpontei_1.fq.gz Read2: Tdelpontei_2.fq.gz
TdelSat67−83.fa_002 Read1: Tinf_Andean_1.fq.gz Read2: Tinf_Andean_2.fq.gz
TdelSat67−83.fa_003 Read1: Tinf_No_Andean_1.fq.gz Read2: Tinf_No_Andean_2.fq.gz
Depth
Position

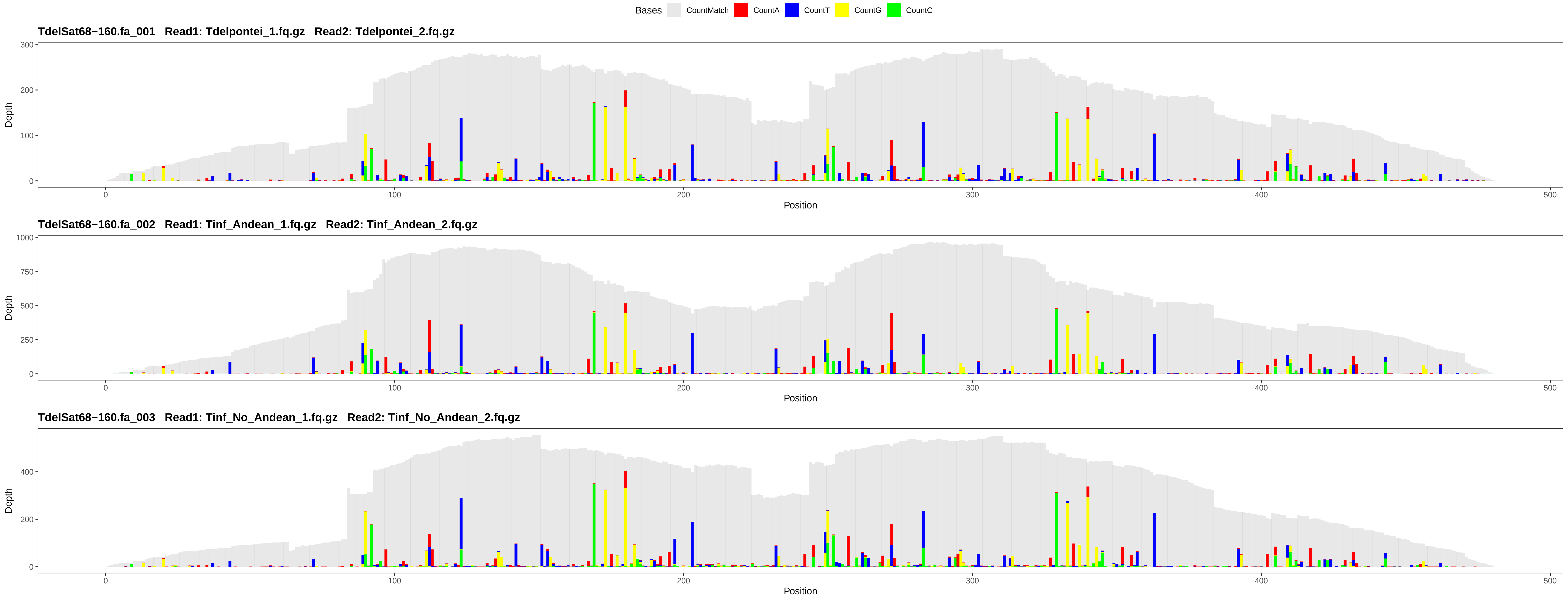
Bases CountMatch CountA CountT CountG CountC
TdelSat68−160.fa_001 Read1: Tdelpontei_1.fq.gz Read2: Tdelpontei_2.fq.gz
Depth
Position
TdelSat68−160.fa_002 Read1: Tinf_Andean_1.fq.gz Read2: Tinf_Andean_2.fq.gz
Depth
Position
TdelSat68−160.fa_003 Read1: Tinf_No_Andean_1.fq.gz Read2: Tinf_No_Andean_2.fq.gz
Depth
Position

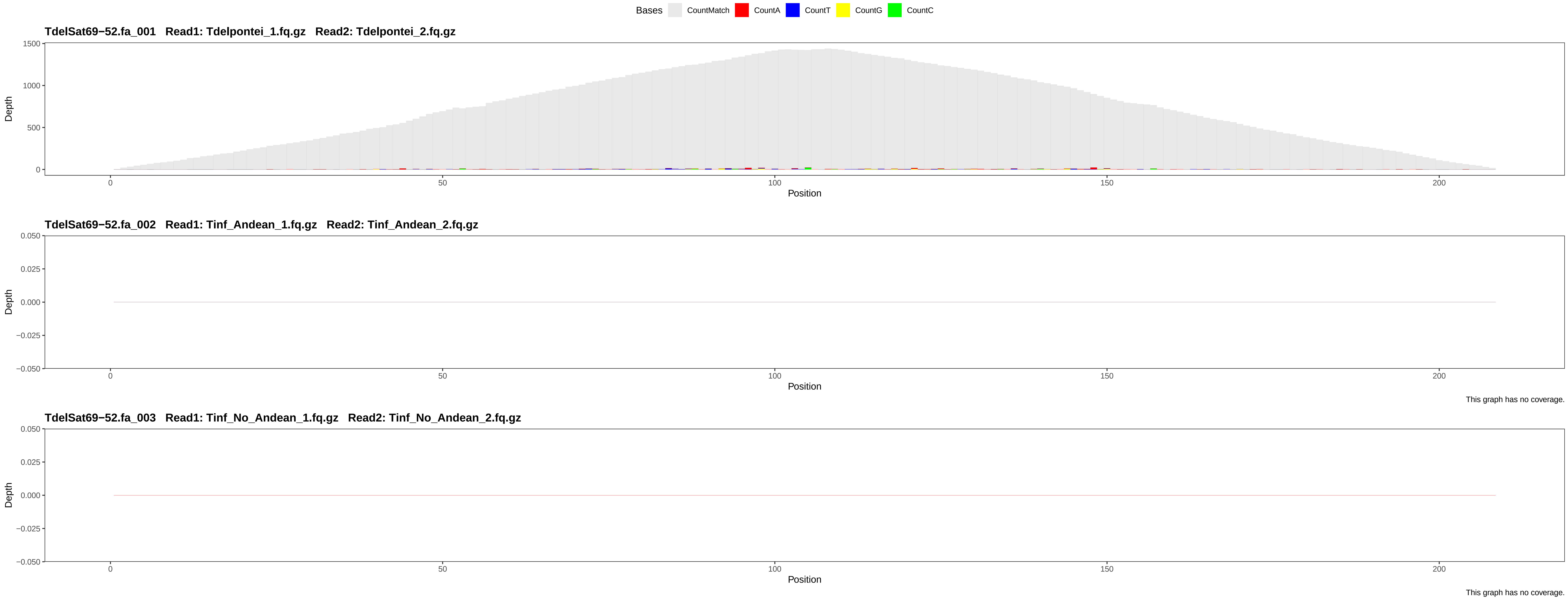

Bases
CountMatch
CountA
CountT
CountG
CountC
TdelSat69−52.fa_001 Read1: Tdelpontei_1.fq.gz Read2: Tdelpontei_2.fq.gz
Depth
Position
TdelSat69−52.fa_002 Read1: Tinf_Andean_1.fq.gz Read2: Tinf_Andean_2.fq.gz
Depth
Position
This graph has no coverage.
TdelSat69−52.fa_003 Read1: Tinf_No_Andean_1.fq.gz Read2: Tinf_No_Andean_2.fq.gz
Depth
Position
This graph has no coverage.

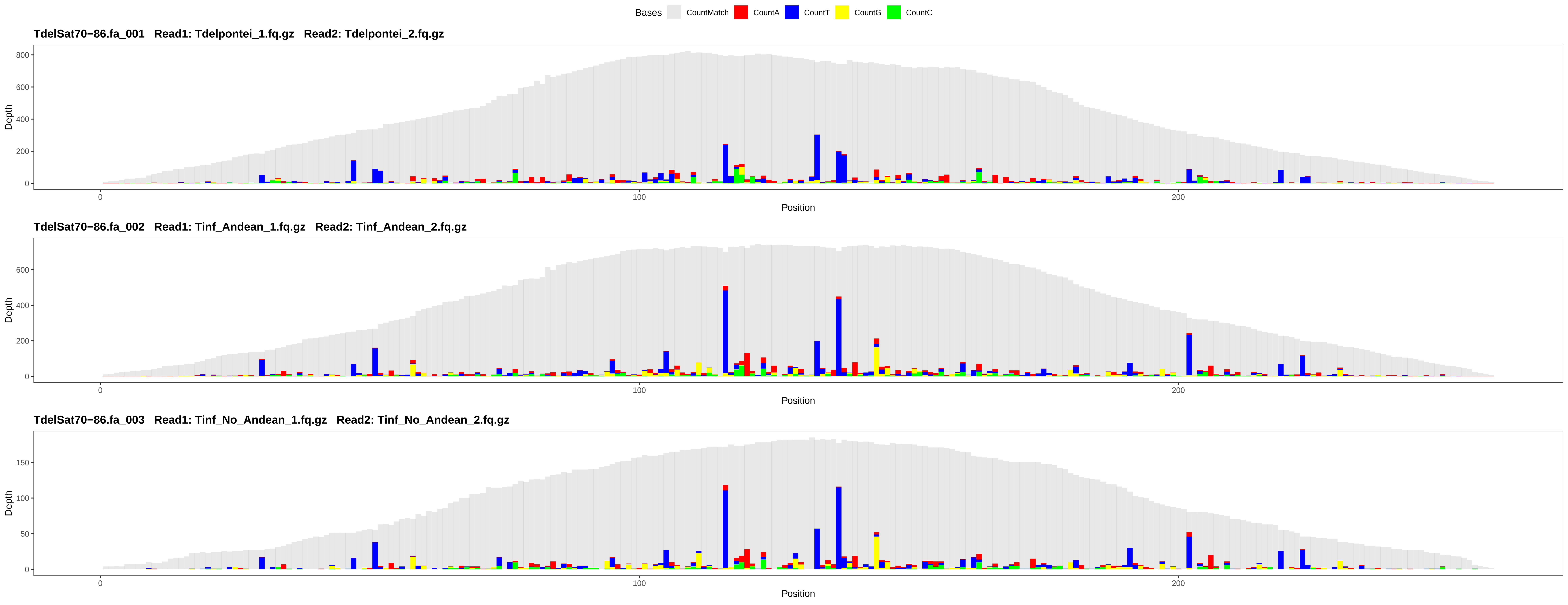
Bases
CountMatch
CountA
CountT
CountG
CountC
TdelSat70−86.fa_001 Read1: Tdelpontei_1.fq.gz Read2: Tdelpontei_2.fq.gz
Depth
0
200
400
600
800
0
100
200
Position
TdelSat70−86.fa_002 Read1: Tinf_Andean_1.fq.gz Read2: Tinf_Andean_2.fq.gz
Depth
0
200
400
600
0
100
200
Position
TdelSat70−86.fa_003 Read1: Tinf_No_Andean_1.fq.gz Read2: Tinf_No_Andean_2.fq.gz
Depth
0
50
100
150
0
100
200
Position

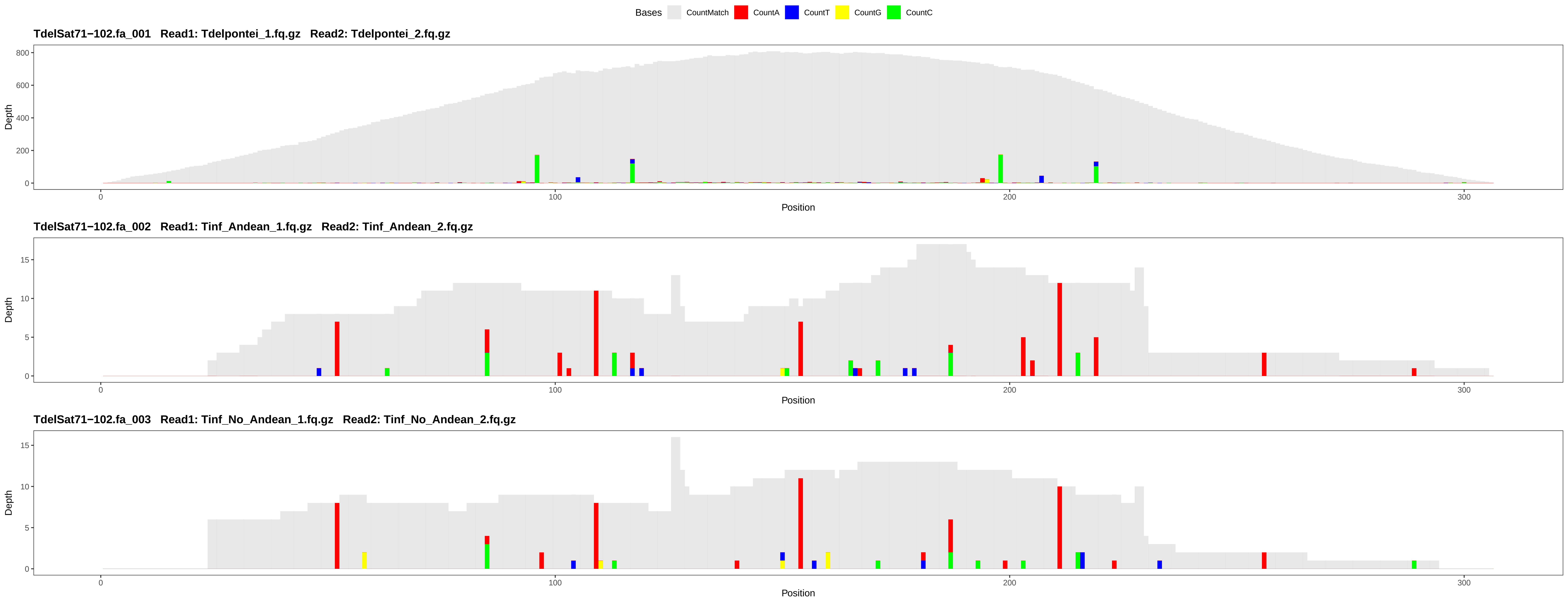
Bases
CountMatch
CountA
CountT
CountG
CountC
TdelSat71−102.fa_001   Read1: Tdelpontei_1.fq.gz   Read2: Tdelpontei_2.fq.gz
Depth
800
600
400
200
0
0
100
200
300
Position
TdelSat71−102.fa_002   Read1: Tinf_Andean_1.fq.gz   Read2: Tinf_Andean_2.fq.gz
Depth
15
10
5
0
0
100
200
300
Position
TdelSat71−102.fa_003   Read1: Tinf_No_Andean_1.fq.gz   Read2: Tinf_No_Andean_2.fq.gz
Depth
15
10
5
0
0
100
200
300
Position

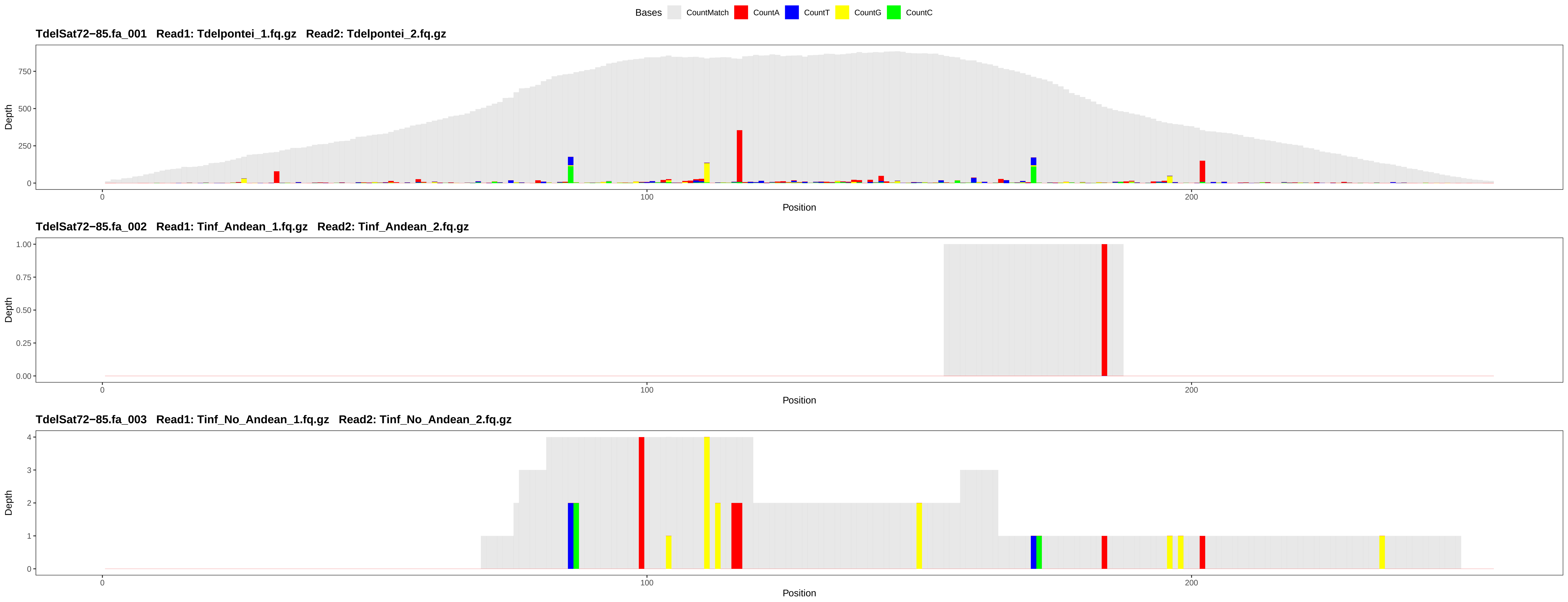
Bases
CountMatch
CountA
CountT
CountG
CountC
TdelSat72−85.fa_001 Read1: Tdelpontei_1.fq.gz Read2: Tdelpontei_2.fq.gz
Depth
0
250
500
750
0
100
200
Position
TdelSat72−85.fa_002 Read1: Tinf_Andean_1.fq.gz Read2: Tinf_Andean_2.fq.gz
Depth
0.00
0.25
0.50
0.75
1.00
0
100
200
Position
TdelSat72−85.fa_003 Read1: Tinf_No_Andean_1.fq.gz Read2: Tinf_No_Andean_2.fq.gz
Depth
0
1
2
3
4
0
100
200
Position

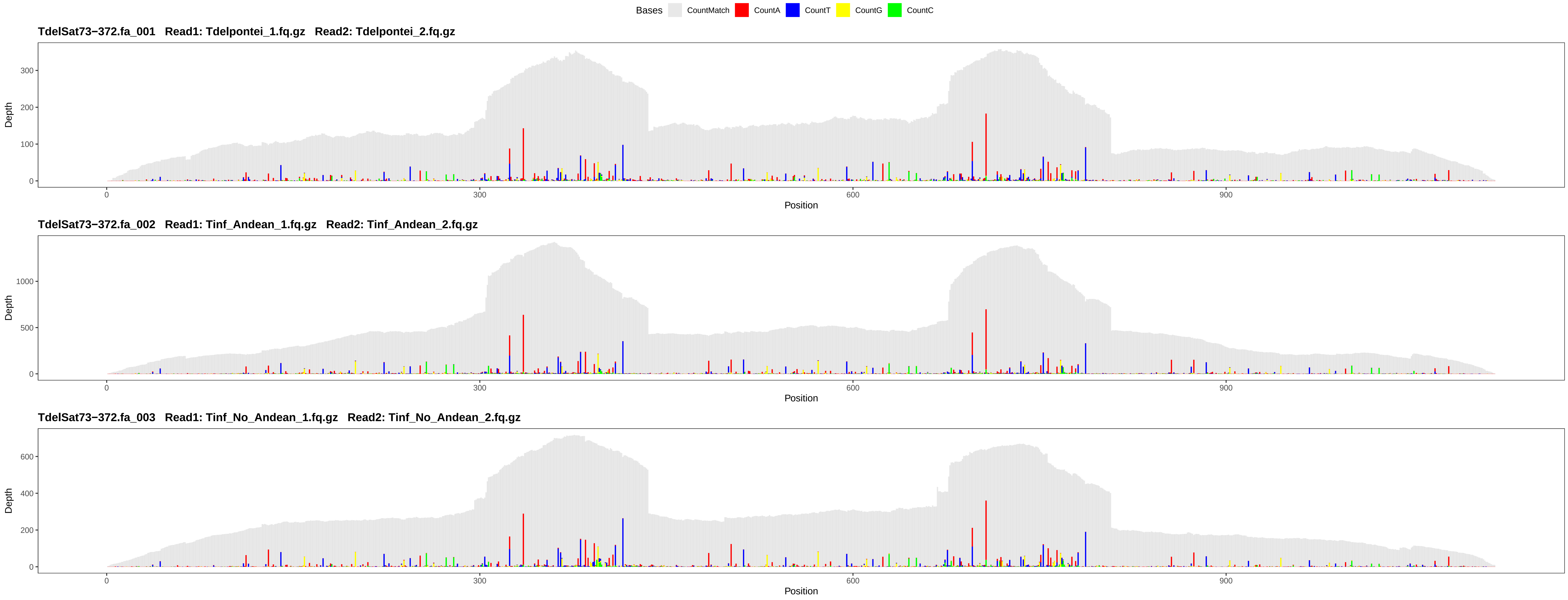
Bases CountMatch CountA CountT CountG CountC
TdelSat73−372.fa_001 Read1: Tdelpontei_1.fq.gz Read2: Tdelpontei_2.fq.gz
Depth
0
100
200
300
Position
0
300
600
900
TdelSat73−372.fa_002 Read1: Tinf_Andean_1.fq.gz Read2: Tinf_Andean_2.fq.gz
Depth
0
500
1000
Position
TdelSat73−372.fa_003 Read1: Tinf_No_Andean_1.fq.gz Read2: Tinf_No_Andean_2.fq.gz
Depth
0
200
400
600
Position

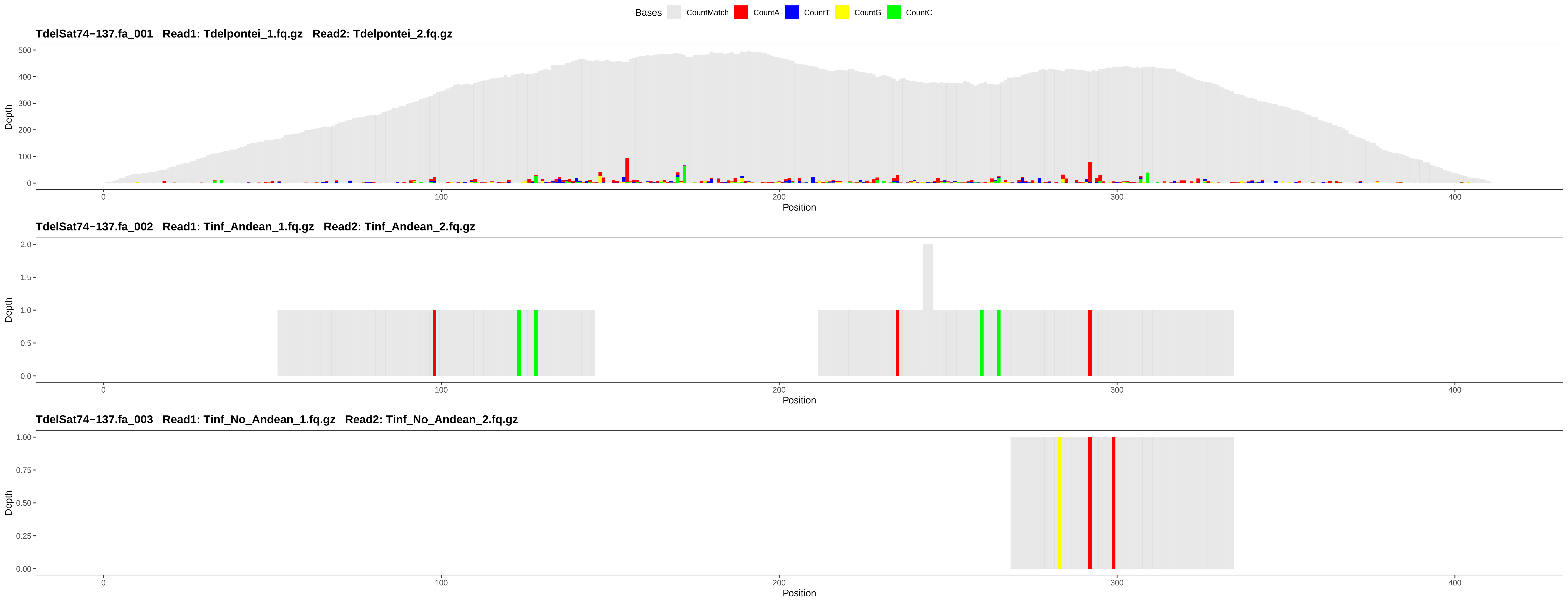
Bases CountMatch CountA CountT CountG CountC
TdelSat74−137.fa_001 Read1: Tdelpontei_1.fq.gz Read2: Tdelpontei_2.fq.gz
Depth
Position
TdelSat74−137.fa_002 Read1: Tinf_Andean_1.fq.gz Read2: Tinf_Andean_2.fq.gz
Depth
Position
TdelSat74−137.fa_003 Read1: Tinf_No_Andean_1.fq.gz Read2: Tinf_No_Andean_2.fq.gz
Depth
Position

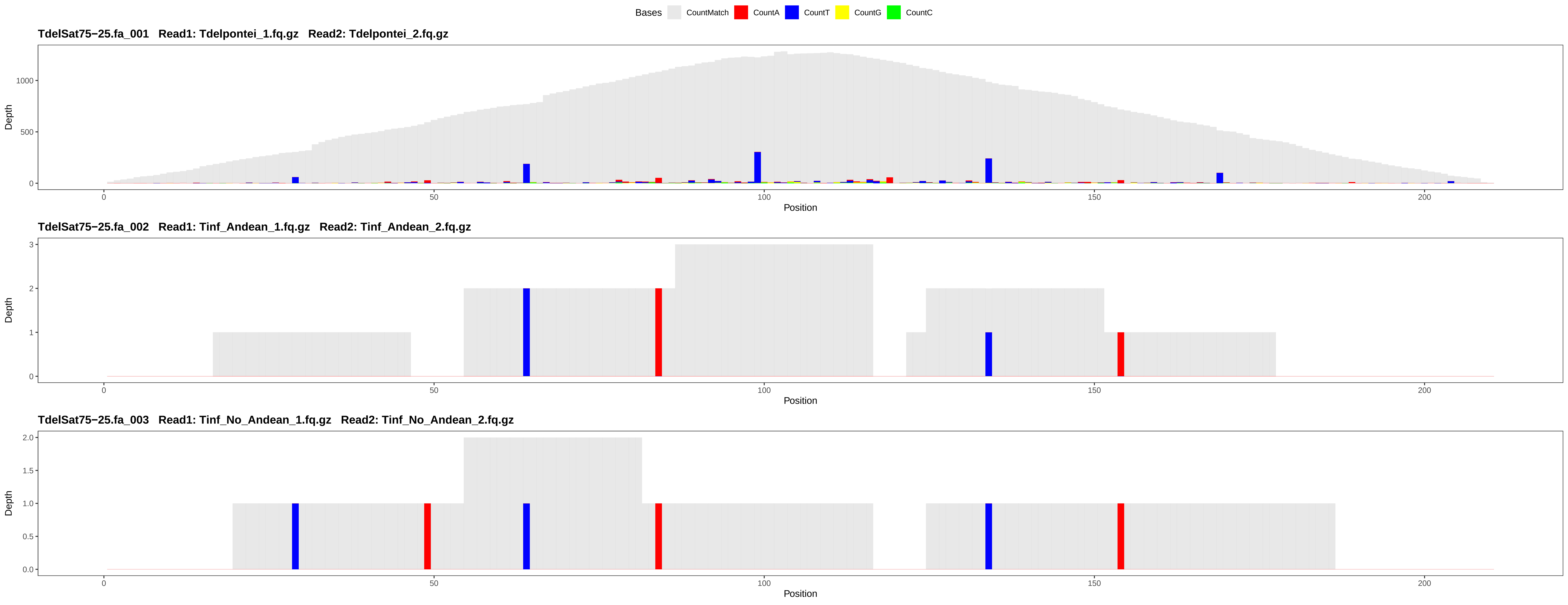

Bases CountMatch CountA CountT CountG CountC
TdelSat75−25.fa_001 Read1: Tdelpontei_1.fq.gz Read2: Tdelpontei_2.fq.gz
Depth
1000
500
0
0
50
100
150
200
Position
TdelSat75−25.fa_002 Read1: Tinf_Andean_1.fq.gz Read2: Tinf_Andean_2.fq.gz
Depth
3
2
1
0
0
50
100
150
200
Position
TdelSat75−25.fa_003 Read1: Tinf_No_Andean_1.fq.gz Read2: Tinf_No_Andean_2.fq.gz
Depth
2.0
1.5
1.0
0.5
0.0
0
50
100
150
200
Position

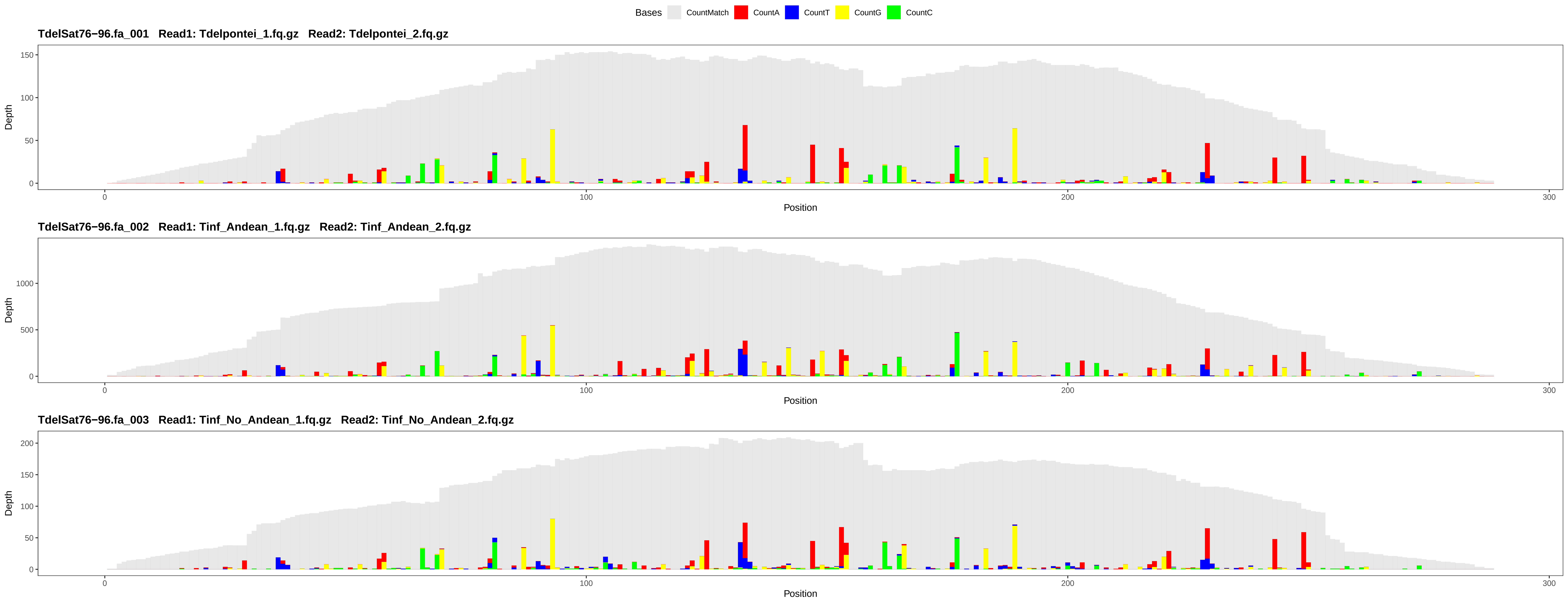
Bases
CountMatch
CountA
CountT
CountG
CountC
TdelSat76−96.fa_001 Read1: Tdelpontei_1.fq.gz Read2: Tdelpontei_2.fq.gz
Depth
150
100
50
0
0
100
200
300
Position
TdelSat76−96.fa_002 Read1: Tinf_Andean_1.fq.gz Read2: Tinf_Andean_2.fq.gz
Depth
1000
500
0
0
100
200
300
Position
TdelSat76−96.fa_003 Read1: Tinf_No_Andean_1.fq.gz Read2: Tinf_No_Andean_2.fq.gz
Depth
200
150
100
50
0
0
100
200
300
Position

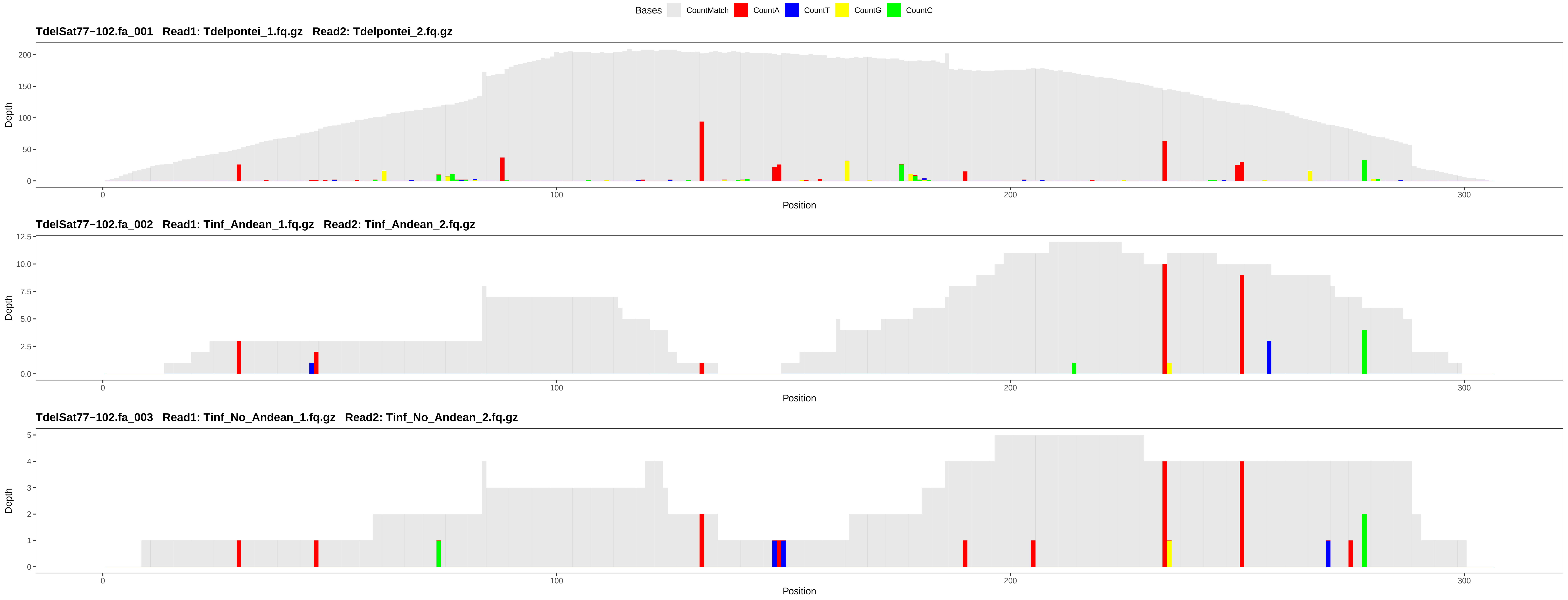
Bases CountMatch CountA CountT CountG CountC
TdelSat77−102.fa_001 Read1: Tdelpontei_1.fq.gz Read2: Tdelpontei_2.fq.gz
Depth
Position
TdelSat77−102.fa_002 Read1: Tinf_Andean_1.fq.gz Read2: Tinf_Andean_2.fq.gz
Depth
Position
TdelSat77−102.fa_003 Read1: Tinf_No_Andean_1.fq.gz Read2: Tinf_No_Andean_2.fq.gz
Depth
Position

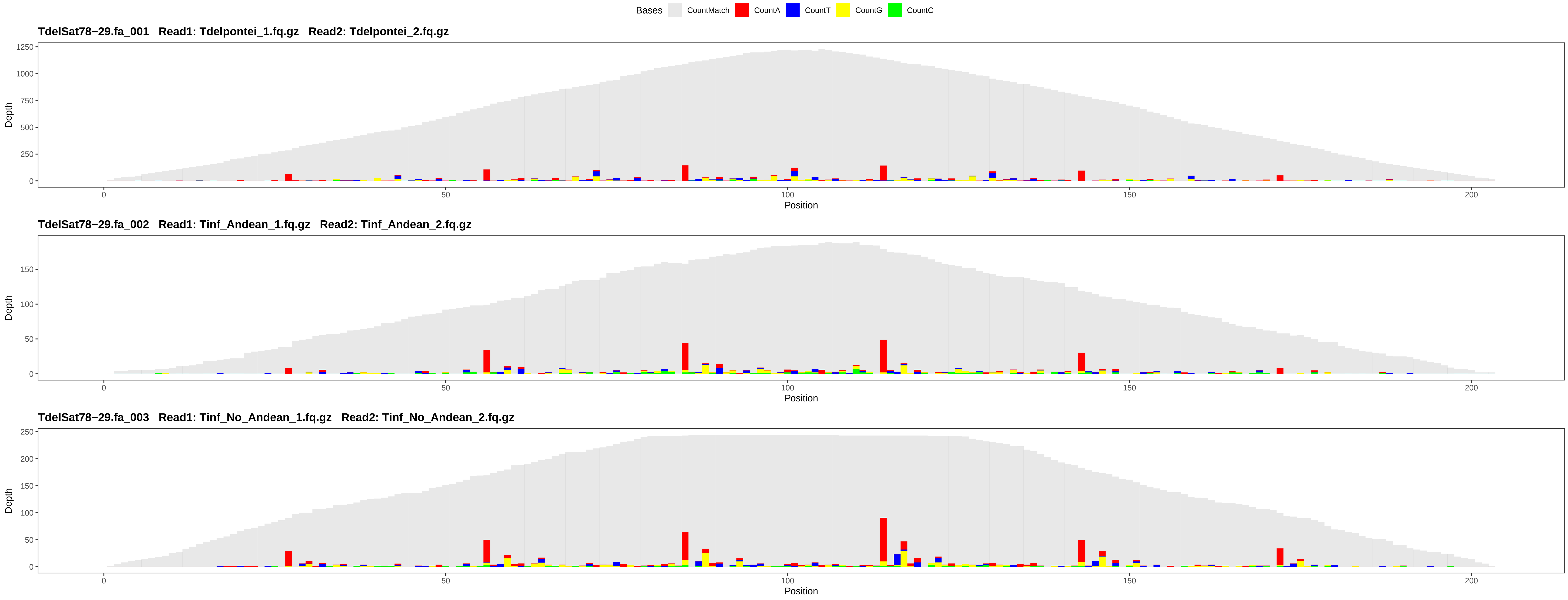
Bases
CountMatch
CountA
CountT
CountG
CountC
TdelSat78−29.fa_001 Read1: Tdelpontei_1.fq.gz Read2: Tdelpontei_2.fq.gz
Depth
1250
1000
750
500
250
0
0
50
100
150
200
Position
TdelSat78−29.fa_002 Read1: Tinf_Andean_1.fq.gz Read2: Tinf_Andean_2.fq.gz
Depth
150
100
50
0
0
50
100
150
200
Position
TdelSat78−29.fa_003 Read1: Tinf_No_Andean_1.fq.gz Read2: Tinf_No_Andean_2.fq.gz
Depth
250
200
150
100
50
0
0
50
100
150
200
Position

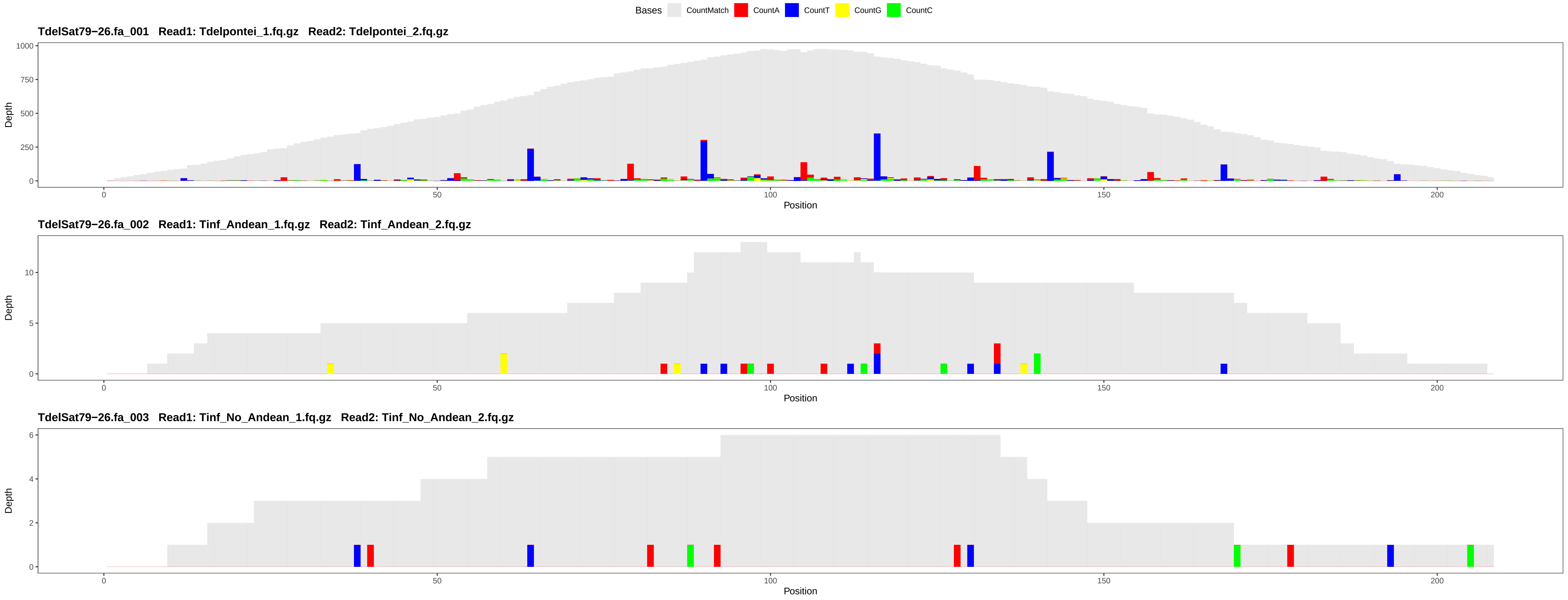
Bases
CountMatch
CountA
CountT
CountG
CountC
TdelSat79−26.fa_001 Read1: Tdelpontei_1.fq.gz Read2: Tdelpontei_2.fq.gz
Depth
1000
750
500
250
0
0
50
100
150
200
Position
TdelSat79−26.fa_002 Read1: Tinf_Andean_1.fq.gz Read2: Tinf_Andean_2.fq.gz
Depth
10
5
0
0
50
100
150
200
Position
TdelSat79−26.fa_003 Read1: Tinf_No_Andean_1.fq.gz Read2: Tinf_No_Andean_2.fq.gz
Depth
6
4
2
0
0
50
100
150
200
Position

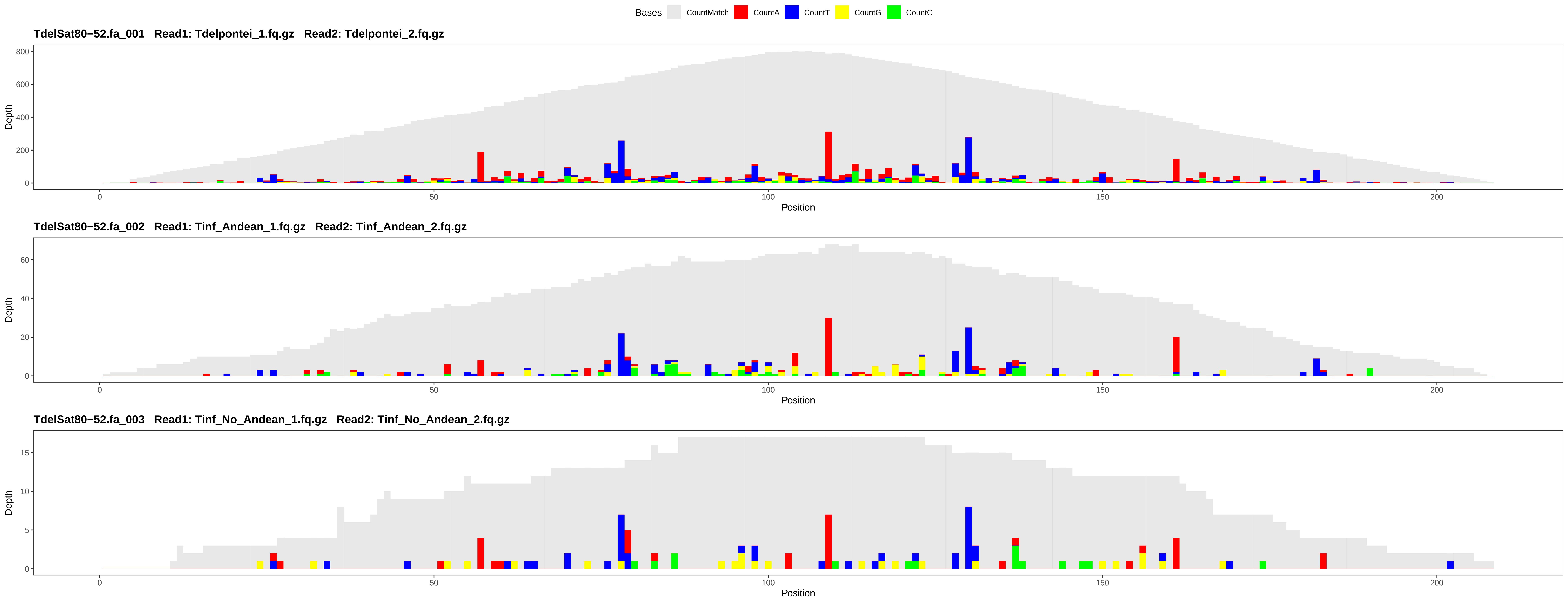

Bases CountMatch CountA CountT CountG CountC
TdelSat80−52.fa_001 Read1: Tdelpontei_1.fq.gz Read2: Tdelpontei_2.fq.gz
Depth
0
200
400
600
800
Position
0
50
100
150
200
TdelSat80−52.fa_002 Read1: Tinf_Andean_1.fq.gz Read2: Tinf_Andean_2.fq.gz
Depth
0
20
40
60
Position
0
50
100
150
200
TdelSat80−52.fa_003 Read1: Tinf_No_Andean_1.fq.gz Read2: Tinf_No_Andean_2.fq.gz
Depth
0
5
10
15
Position
0
50
100
150
200

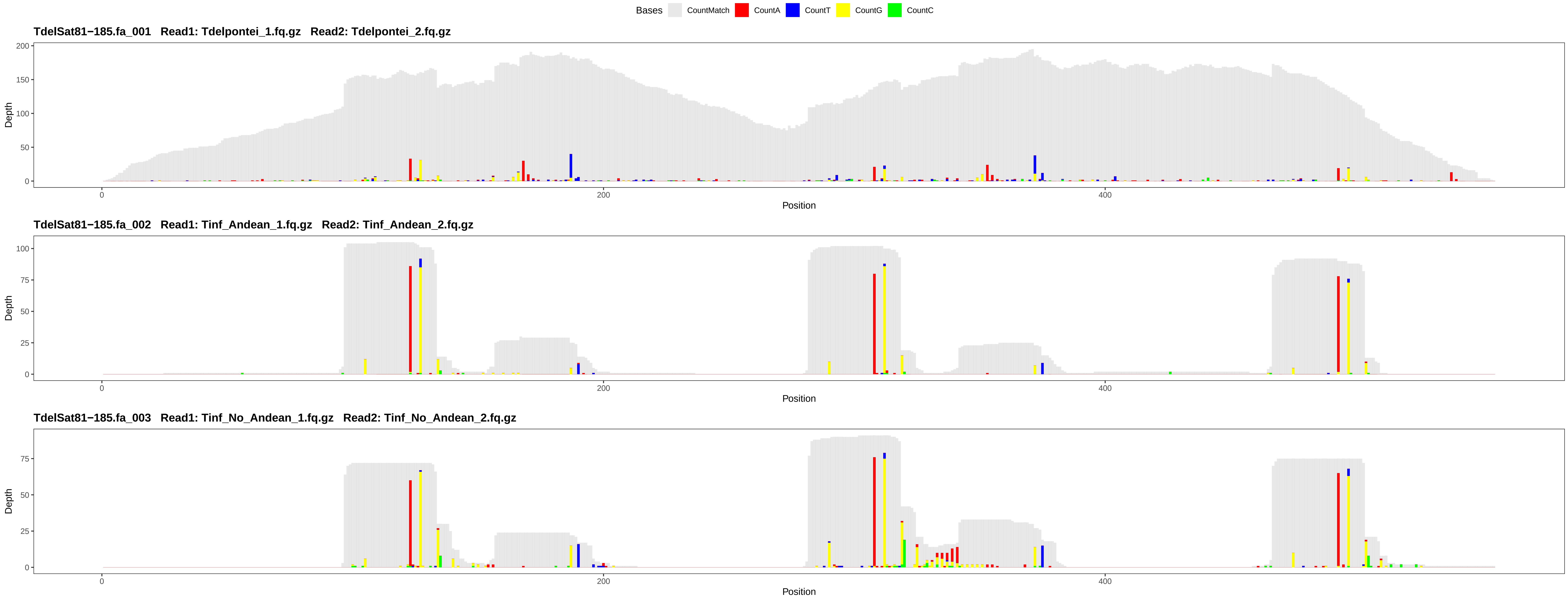
Bases
CountMatch
CountA
CountT
CountG
CountC
TdelSat81−185.fa_001 Read1: Tdelpontei_1.fq.gz Read2: Tdelpontei_2.fq.gz
Depth
Position
TdelSat81−185.fa_002 Read1: Tinf_Andean_1.fq.gz Read2: Tinf_Andean_2.fq.gz
Depth
Position
TdelSat81−185.fa_003 Read1: Tinf_No_Andean_1.fq.gz Read2: Tinf_No_Andean_2.fq.gz
Depth
Position

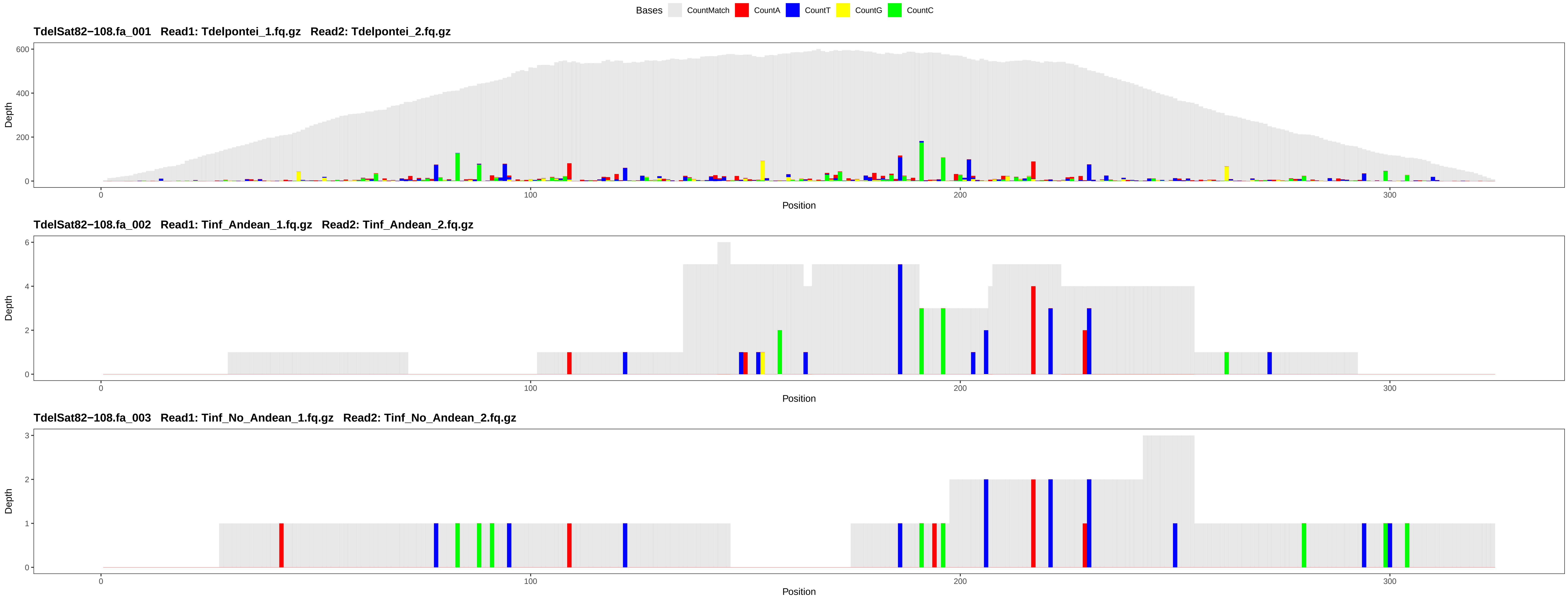
Bases
CountMatch
CountA
CountT
CountG
CountC
TdelSat82−108.fa_001 Read1: Tdelpontei_1.fq.gz Read2: Tdelpontei_2.fq.gz
Depth
Position
TdelSat82−108.fa_002 Read1: Tinf_Andean_1.fq.gz Read2: Tinf_Andean_2.fq.gz
Depth
Position
TdelSat82−108.fa_003 Read1: Tinf_No_Andean_1.fq.gz Read2: Tinf_No_Andean_2.fq.gz
Depth
Position

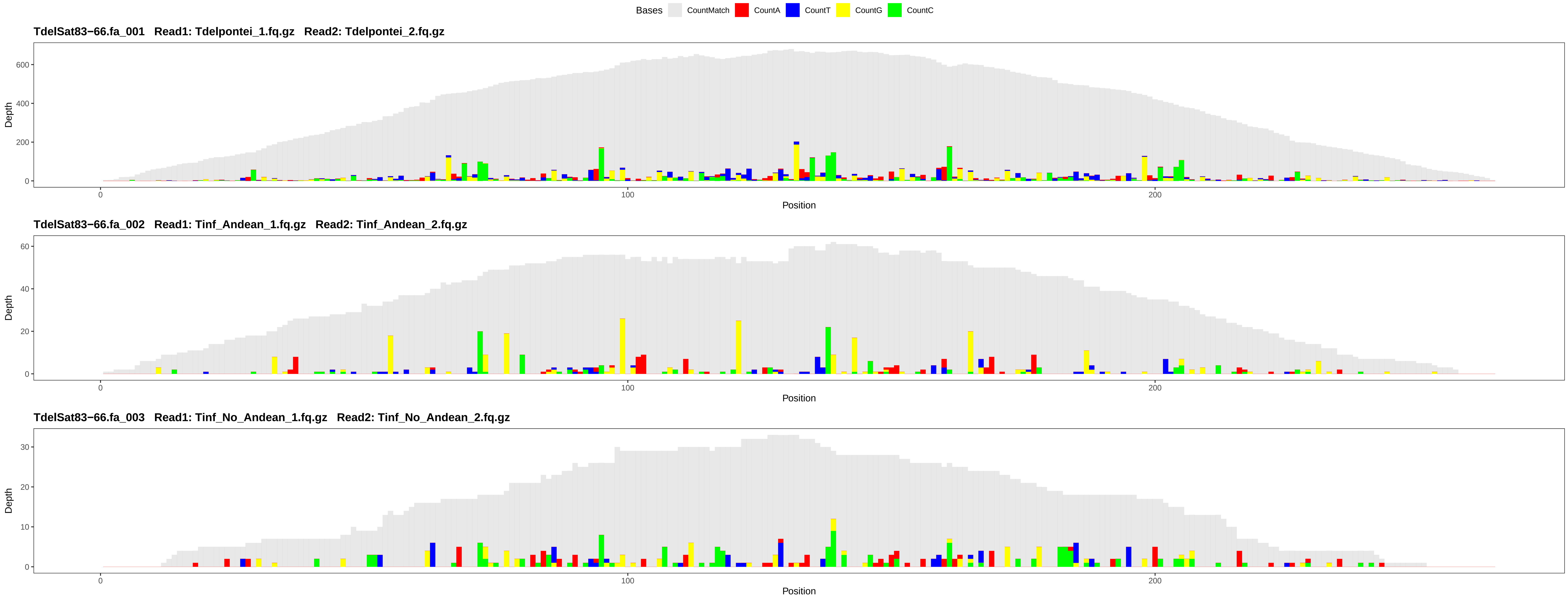

Bases
CountMatch
CountA
CountT
CountG
CountC
TdelSat83−66.fa_001 Read1: Tdelpontei_1.fq.gz Read2: Tdelpontei_2.fq.gz
Depth
0
200
400
600
Position
0
100
200
TdelSat83−66.fa_002 Read1: Tinf_Andean_1.fq.gz Read2: Tinf_Andean_2.fq.gz
Depth
0
20
40
60
Position
0
100
200
TdelSat83−66.fa_003 Read1: Tinf_No_Andean_1.fq.gz Read2: Tinf_No_Andean_2.fq.gz
Depth
0
10
20
30
Position
0
100
200

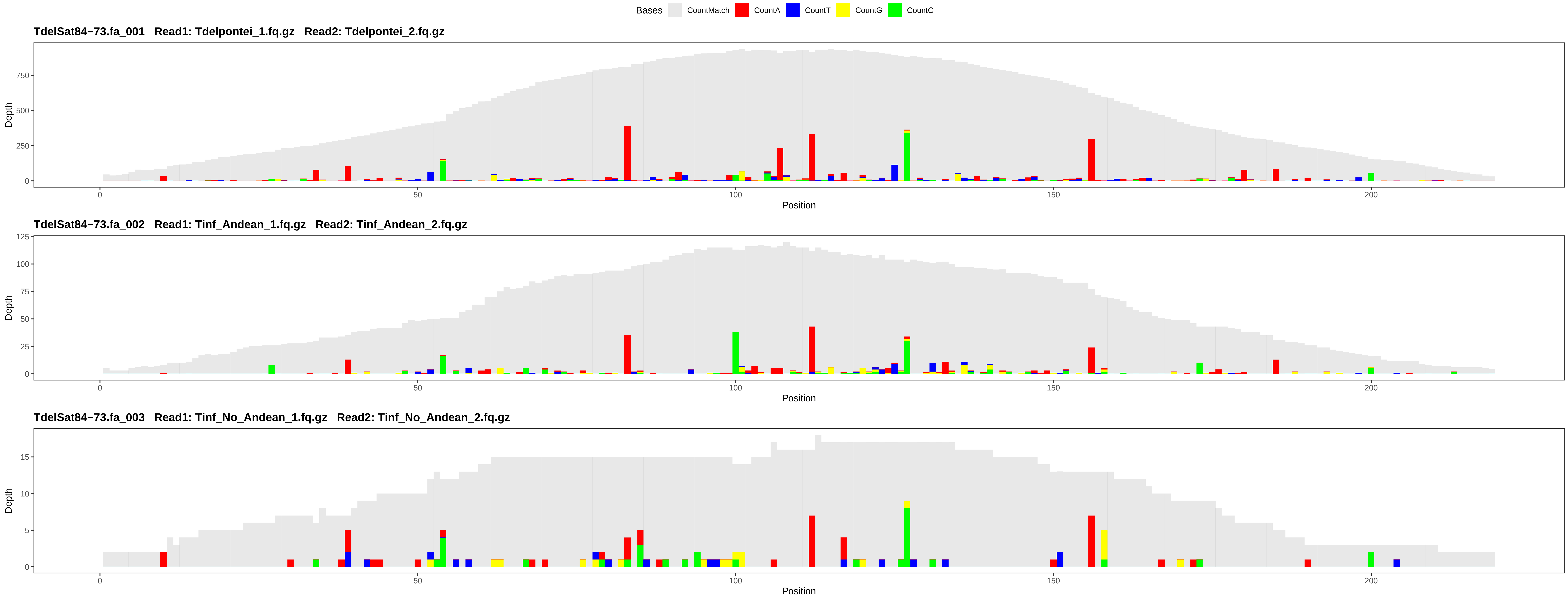
Bases CountMatch CountA CountT CountG CountC
TdelSat84−73.fa_001 Read1: Tdelpontei_1.fq.gz Read2: Tdelpontei_2.fq.gz
Depth
750
500
250
0
0
50
100
150
200
Position
TdelSat84−73.fa_002 Read1: Tinf_Andean_1.fq.gz Read2: Tinf_Andean_2.fq.gz
Depth
125
100
75
50
25
0
0
50
100
150
200
Position
TdelSat84−73.fa_003 Read1: Tinf_No_Andean_1.fq.gz Read2: Tinf_No_Andean_2.fq.gz
Depth
15
10
5
0
0
50
100
150
200
Position

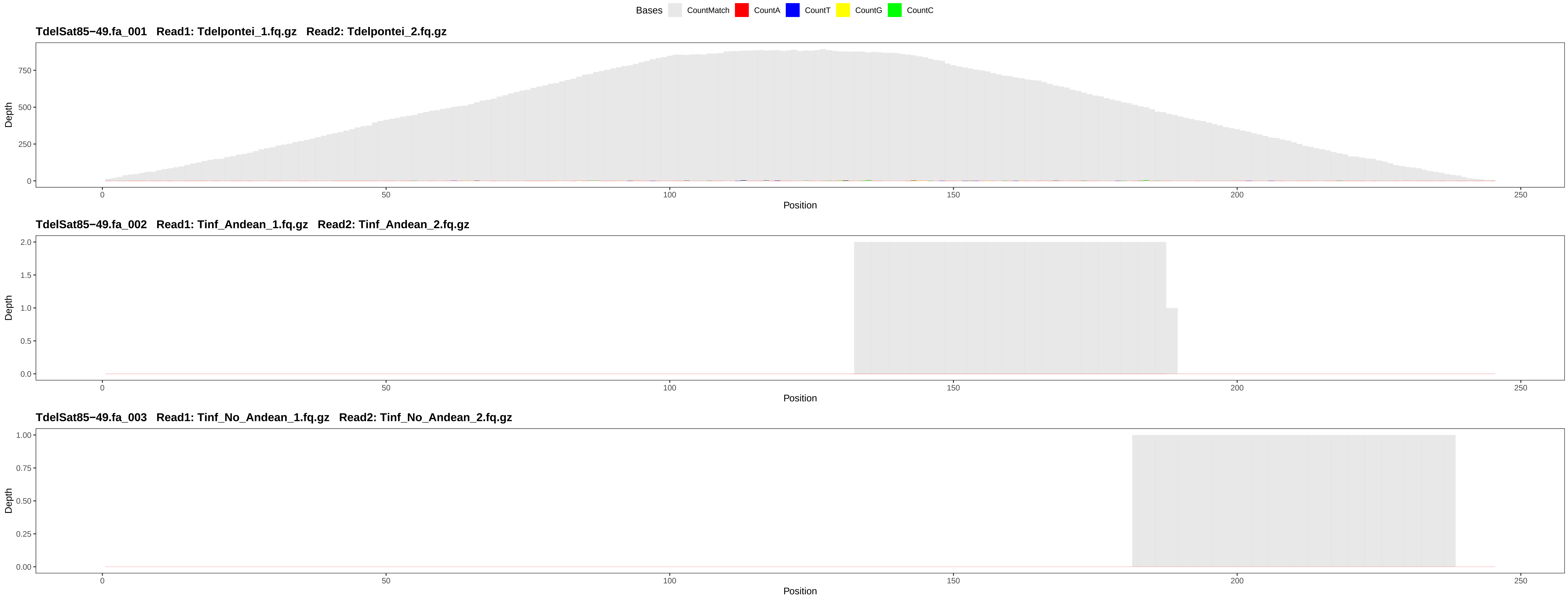

Bases CountMatch CountA CountT CountG CountC
TdelSat85−49.fa_001 Read1: Tdelpontei_1.fq.gz Read2: Tdelpontei_2.fq.gz
TdelSat85−49.fa_002 Read1: Tinf_Andean_1.fq.gz Read2: Tinf_Andean_2.fq.gz
TdelSat85−49.fa_003 Read1: Tinf_No_Andean_1.fq.gz Read2: Tinf_No_Andean_2.fq.gz
Depth
Position

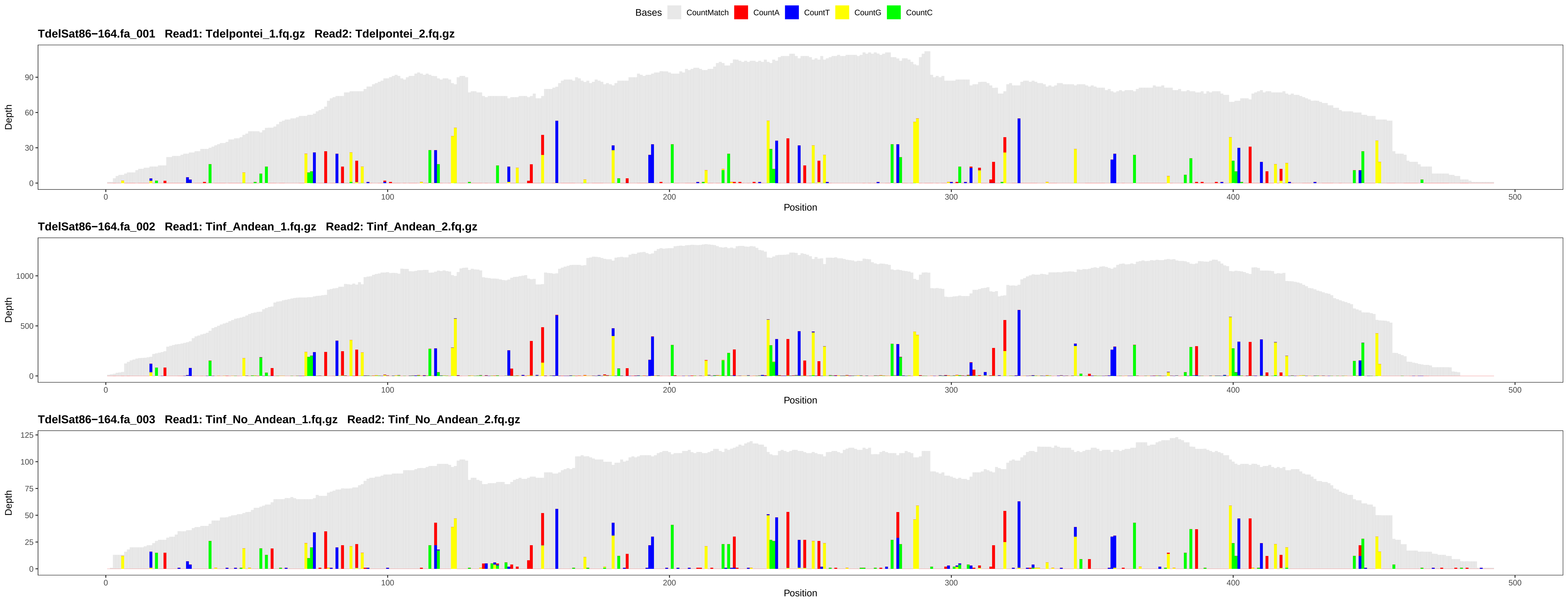
Bases CountMatch CountA CountT CountG CountC
TdelSat86−164.fa_001 Read1: Tdelpontei_1.fq.gz Read2: Tdelpontei_2.fq.gz
Depth
0
30
60
90
Position
0
100
200
300
400
500
TdelSat86−164.fa_002 Read1: Tinf_Andean_1.fq.gz Read2: Tinf_Andean_2.fq.gz
Depth
0
500
1000
Position
0
100
200
300
400
500
TdelSat86−164.fa_003 Read1: Tinf_No_Andean_1.fq.gz Read2: Tinf_No_Andean_2.fq.gz
Depth
0
25
50
75
100
125
Position
0
100
200
300
400
500

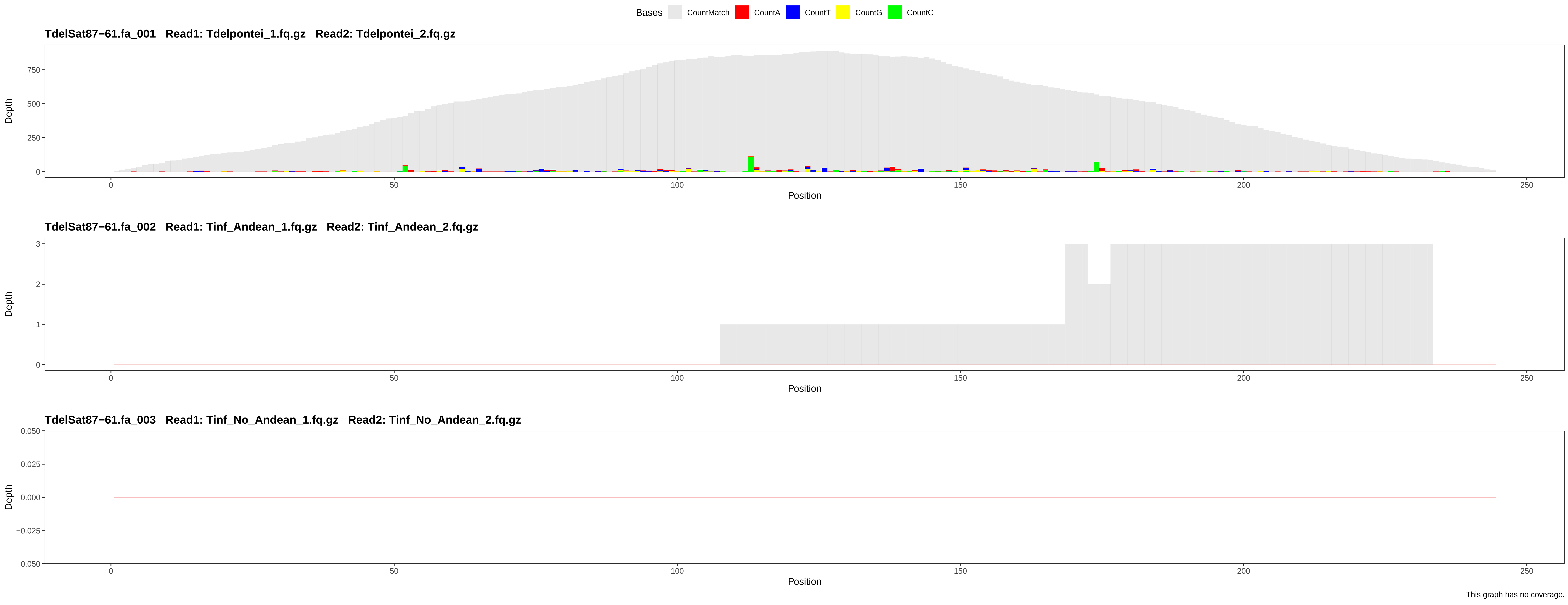

Bases
CountMatch
CountA
CountT
CountG
CountC
TdelSat87−61.fa_001 Read1: Tdelpontei_1.fq.gz Read2: Tdelpontei_2.fq.gz
Depth
750
500
250
0
Position
0
50
100
150
200
250
TdelSat87−61.fa_002 Read1: Tinf_Andean_1.fq.gz Read2: Tinf_Andean_2.fq.gz
Depth
3
2
1
0
Position
TdelSat87−61.fa_003 Read1: Tinf_No_Andean_1.fq.gz Read2: Tinf_No_Andean_2.fq.gz
Depth
0.050
0.025
0.000
−0.025
−0.050
Position
This graph has no coverage.

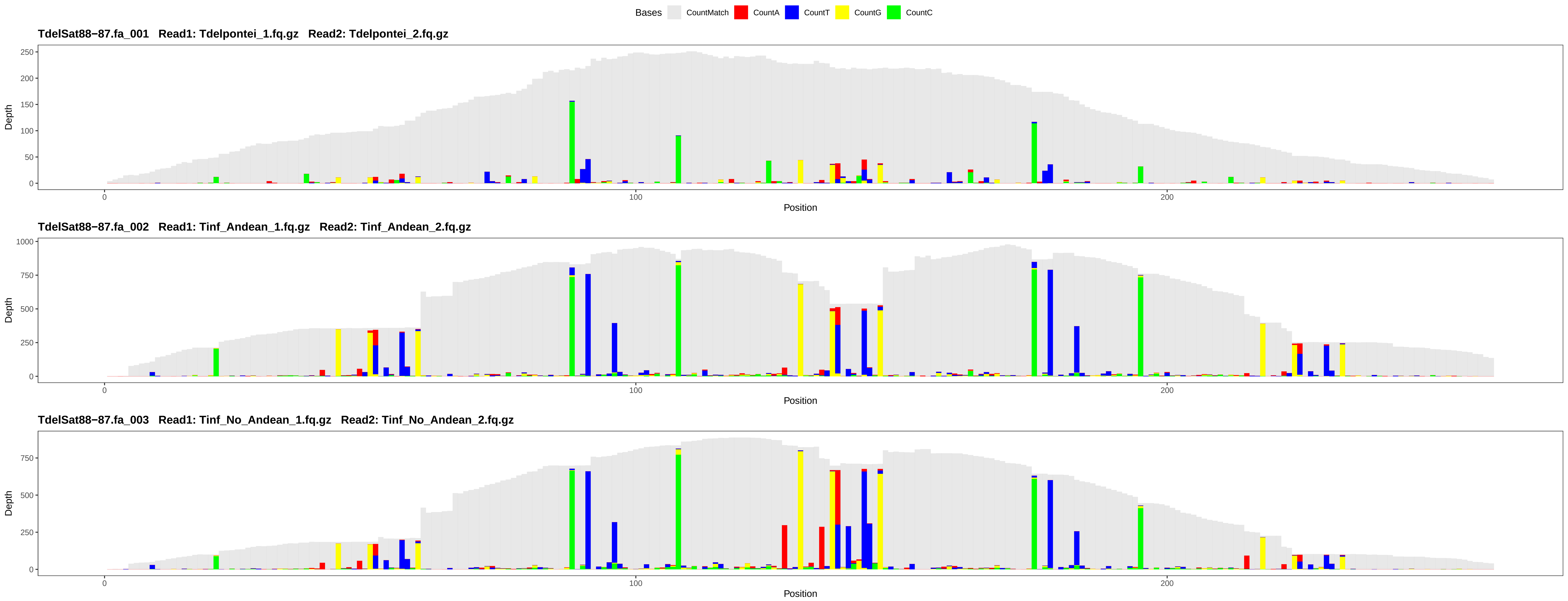
Bases CountMatch CountA CountT CountG CountC
TdelSat88−87.fa_001 Read1: Tdelpontei_1.fq.gz Read2: Tdelpontei_2.fq.gz
Depth
Position
TdelSat88−87.fa_002 Read1: Tinf_Andean_1.fq.gz Read2: Tinf_Andean_2.fq.gz
Depth
Position
TdelSat88−87.fa_003 Read1: Tinf_No_Andean_1.fq.gz Read2: Tinf_No_Andean_2.fq.gz
Depth
Position

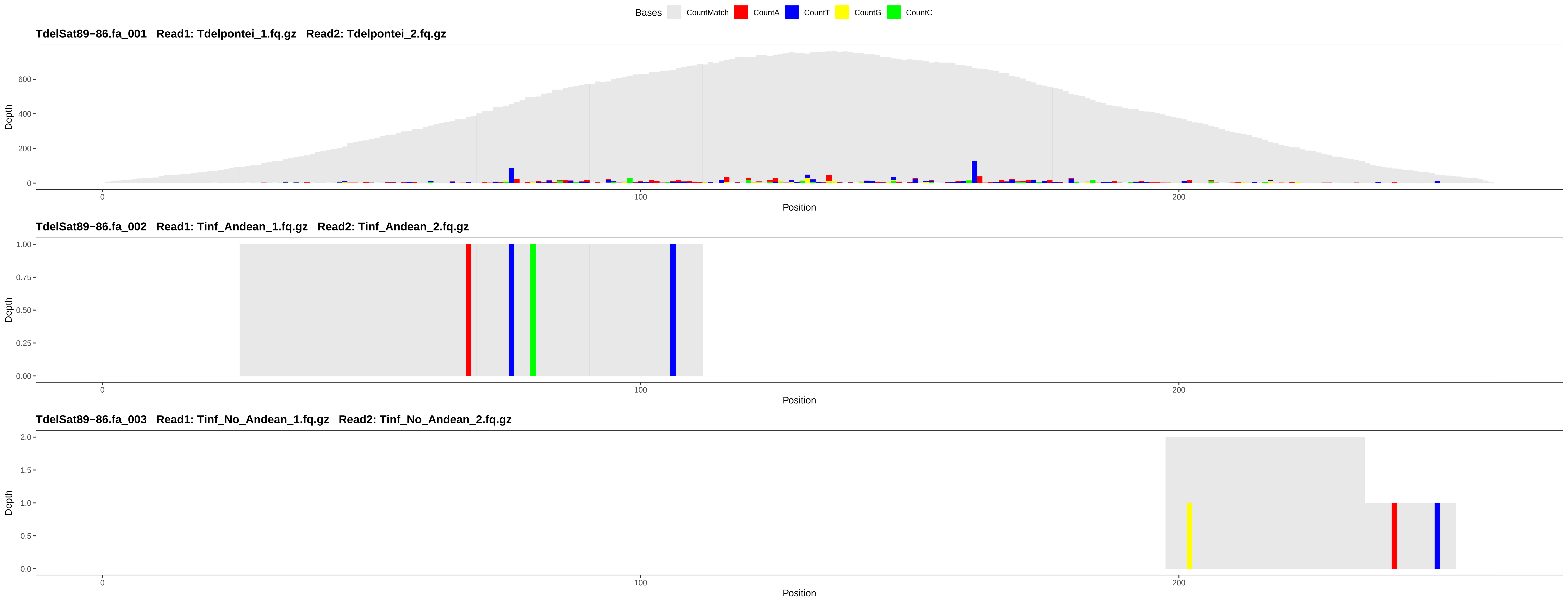
Bases
CountMatch
CountA
CountT
CountG
CountC
TdelSat89−86.fa_001 Read1: Tdelpontei_1.fq.gz Read2: Tdelpontei_2.fq.gz
Depth
Position
TdelSat89−86.fa_002 Read1: Tinf_Andean_1.fq.gz Read2: Tinf_Andean_2.fq.gz
Depth
Position
TdelSat89−86.fa_003 Read1: Tinf_No_Andean_1.fq.gz Read2: Tinf_No_Andean_2.fq.gz
Depth
Position

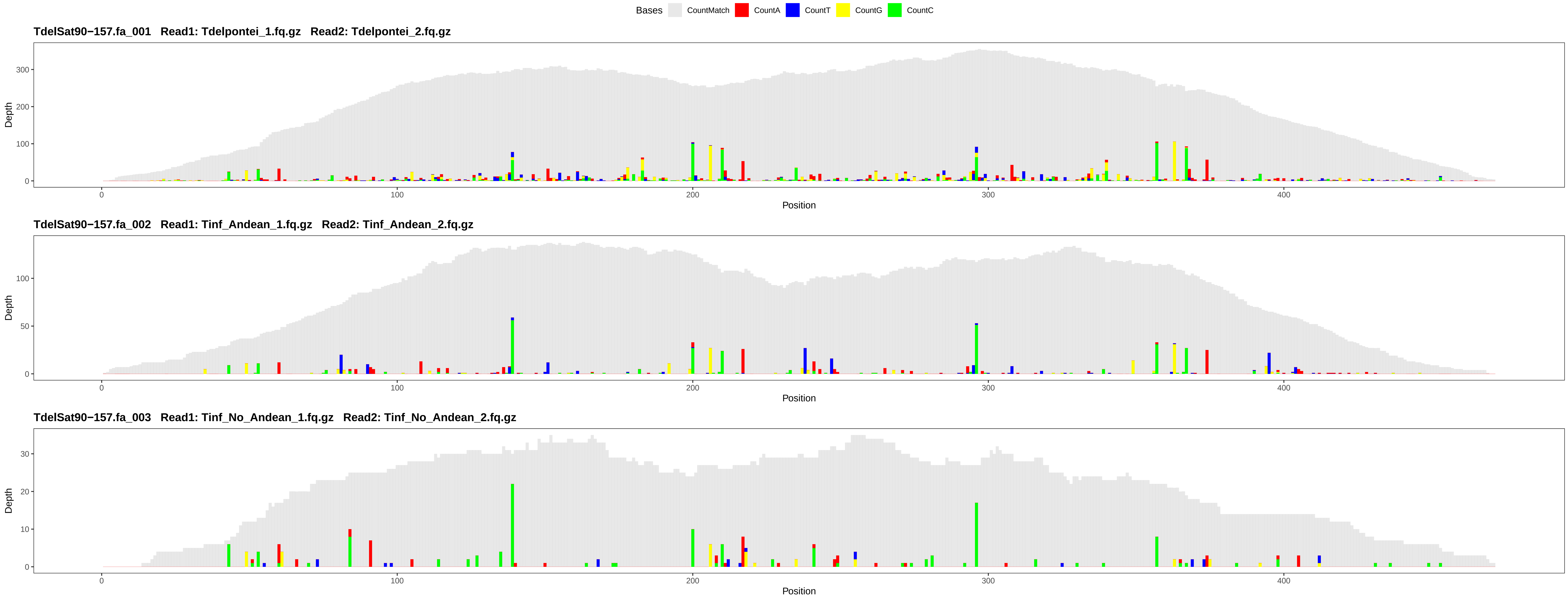
Bases CountMatch CountA CountT CountG CountC
TdelSat90−157.fa_001 Read1: Tdelpontei_1.fq.gz Read2: Tdelpontei_2.fq.gz
TdelSat90−157.fa_002 Read1: Tinf_Andean_1.fq.gz Read2: Tinf_Andean_2.fq.gz
TdelSat90−157.fa_003 Read1: Tinf_No_Andean_1.fq.gz Read2: Tinf_No_Andean_2.fq.gz
Depth
Position
0
100
200
300
400

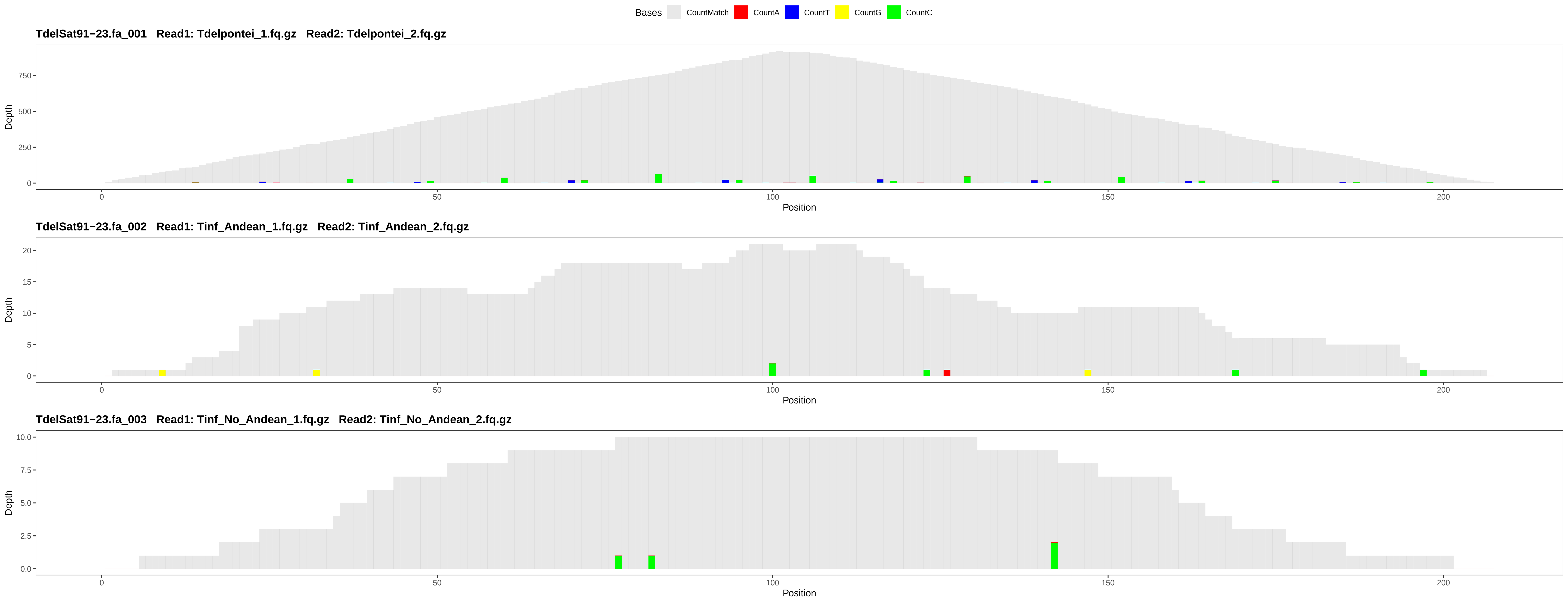
Bases CountMatch CountA CountT CountG CountC
TdelSat91−23.fa_001 Read1: Tdelpontei_1.fq.gz Read2: Tdelpontei_2.fq.gz
Depth
Position
TdelSat91−23.fa_002 Read1: Tinf_Andean_1.fq.gz Read2: Tinf_Andean_2.fq.gz
Depth
Position
TdelSat91−23.fa_003 Read1: Tinf_No_Andean_1.fq.gz Read2: Tinf_No_Andean_2.fq.gz
Depth
Position

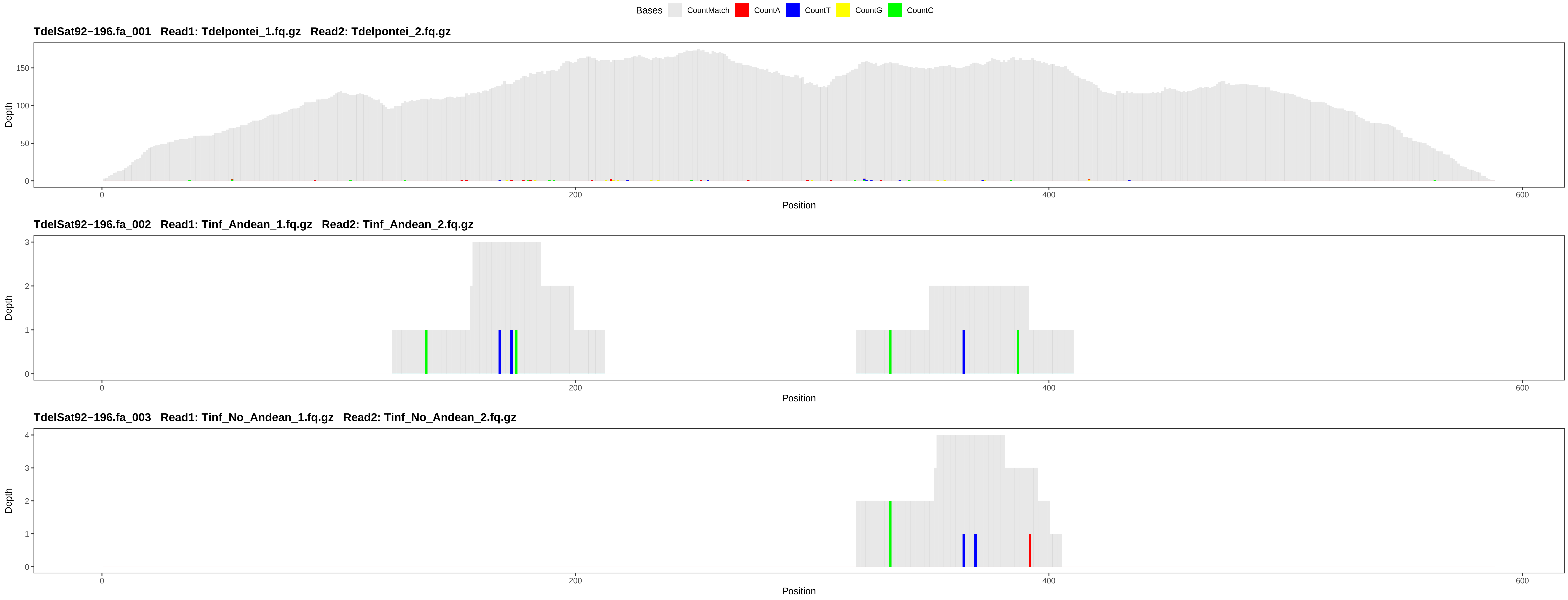

Bases CountMatch CountA CountT CountG CountC
TdelSat92−196.fa_001 Read1: Tdelpontei_1.fq.gz Read2: Tdelpontei_2.fq.gz
Depth
Position
TdelSat92−196.fa_002 Read1: Tinf_Andean_1.fq.gz Read2: Tinf_Andean_2.fq.gz
Depth
Position
TdelSat92−196.fa_003 Read1: Tinf_No_Andean_1.fq.gz Read2: Tinf_No_Andean_2.fq.gz
Depth
Position

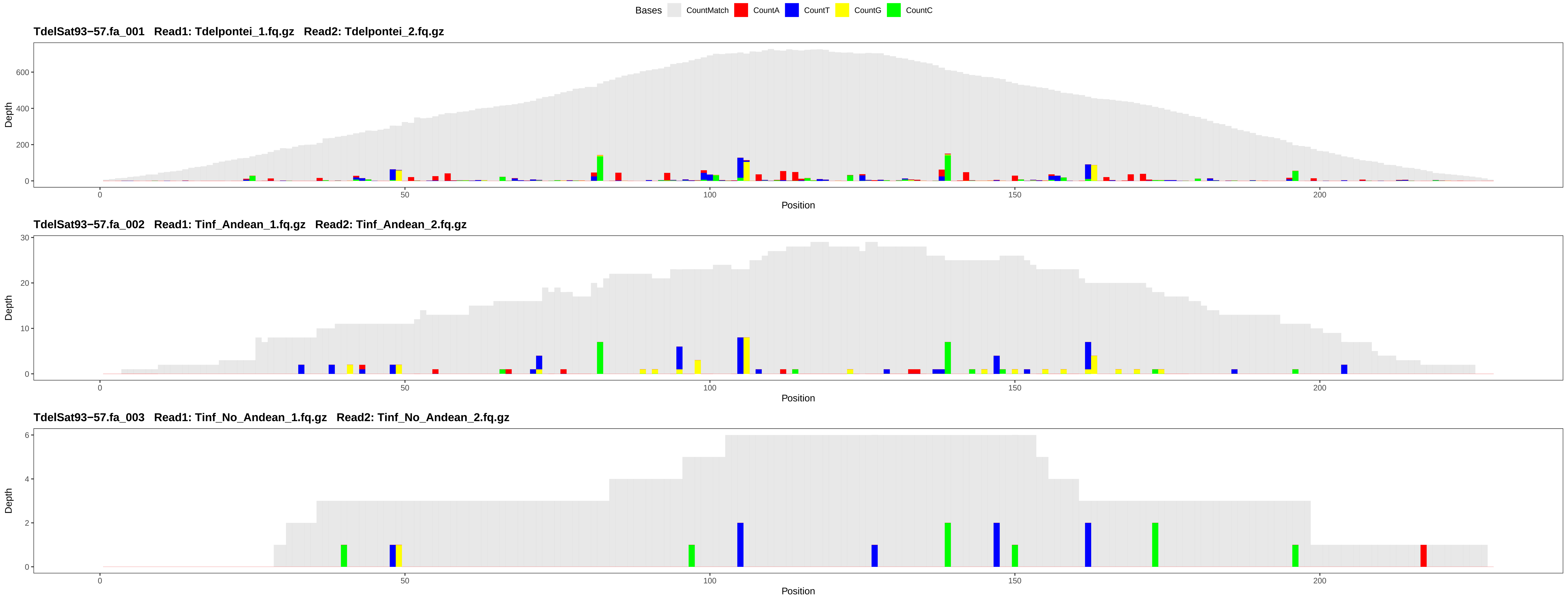
Bases
CountMatch
CountA
CountT
CountG
CountC
TdelSat93−57.fa_001 Read1: Tdelpontei_1.fq.gz Read2: Tdelpontei_2.fq.gz
Depth
Position
TdelSat93−57.fa_002 Read1: Tinf_Andean_1.fq.gz Read2: Tinf_Andean_2.fq.gz
Depth
Position
TdelSat93−57.fa_003 Read1: Tinf_No_Andean_1.fq.gz Read2: Tinf_No_Andean_2.fq.gz
Depth
Position

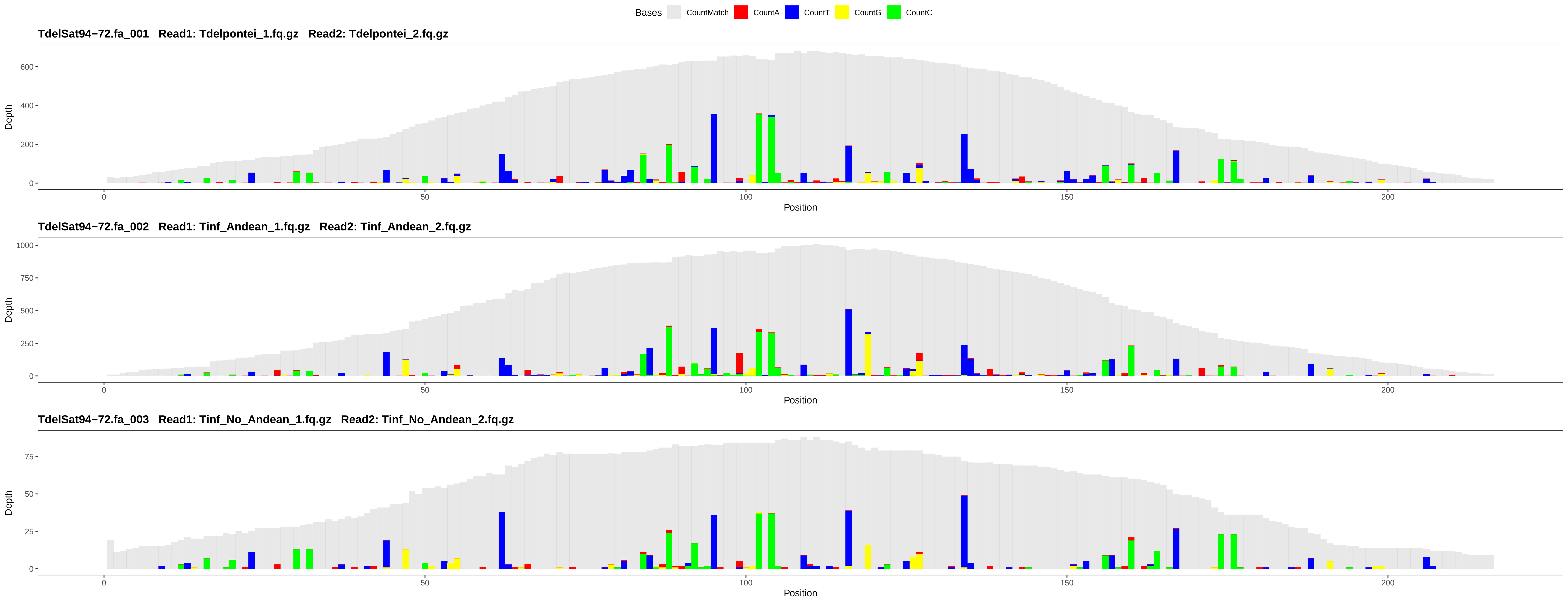
Bases CountMatch CountA CountT CountG CountC
TdelSat94−72.fa_001 Read1: Tdelpontei_1.fq.gz Read2: Tdelpontei_2.fq.gz
Depth
Position
TdelSat94−72.fa_002 Read1: Tinf_Andean_1.fq.gz Read2: Tinf_Andean_2.fq.gz
Depth
Position
TdelSat94−72.fa_003 Read1: Tinf_No_Andean_1.fq.gz Read2: Tinf_No_Andean_2.fq.gz
Depth
Position

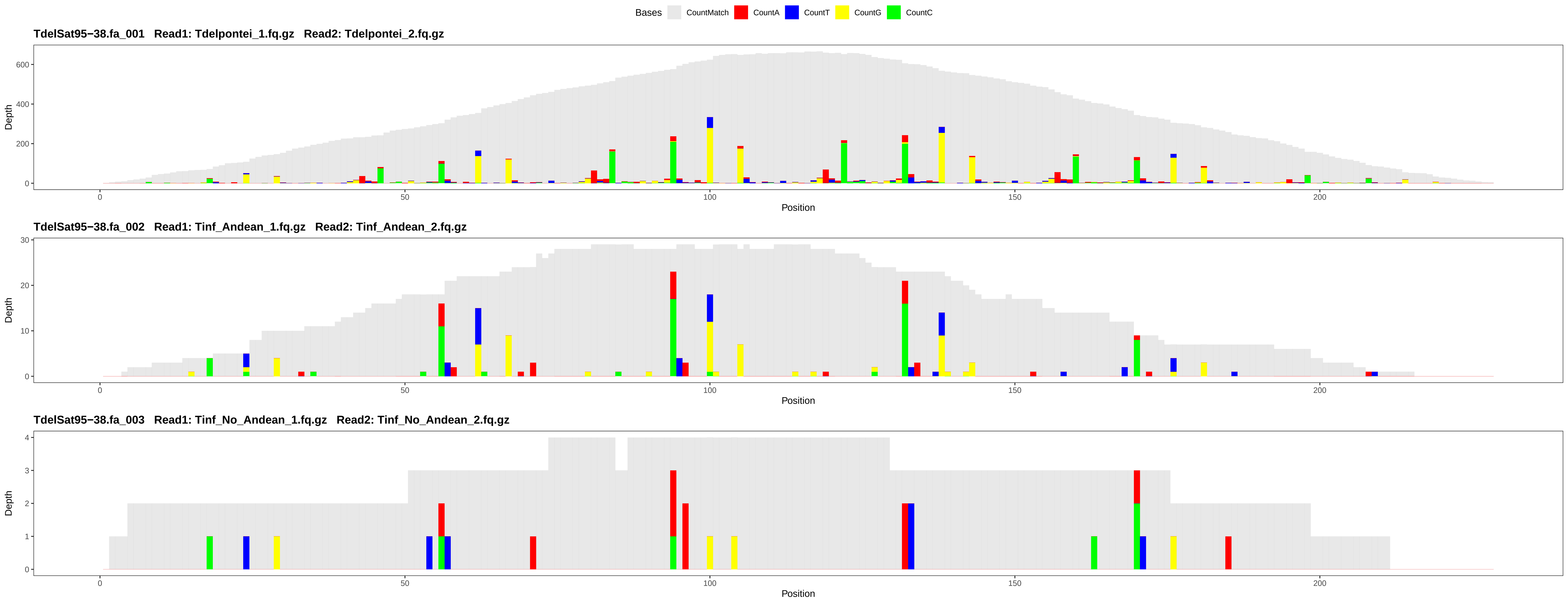

Bases CountMatch CountA CountT CountG CountC
TdelSat95−38.fa_001 Read1: Tdelpontei_1.fq.gz Read2: Tdelpontei_2.fq.gz
Depth
0
200
400
600
Position
0
50
100
150
200
TdelSat95−38.fa_002 Read1: Tinf_Andean_1.fq.gz Read2: Tinf_Andean_2.fq.gz
Depth
0
10
20
30
Position
0
50
100
150
200
TdelSat95−38.fa_003 Read1: Tinf_No_Andean_1.fq.gz Read2: Tinf_No_Andean_2.fq.gz
Depth
0
1
2
3
4
Position
0
50
100
150
200

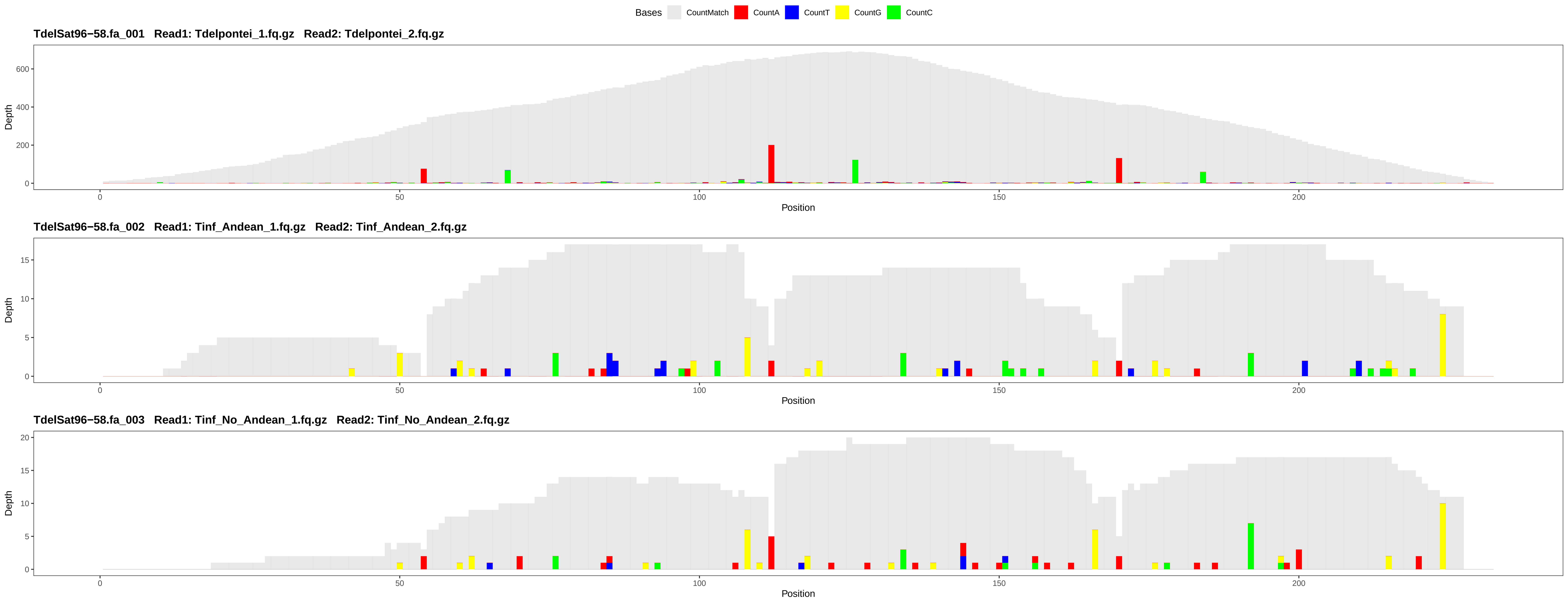

Bases
CountMatch
CountA
CountT
CountG
CountC
TdelSat96−58.fa_001 Read1: Tdelpontei_1.fq.gz Read2: Tdelpontei_2.fq.gz
Depth
0
200
400
600
Position
0
50
100
150
200
TdelSat96−58.fa_002 Read1: Tinf_Andean_1.fq.gz Read2: Tinf_Andean_2.fq.gz
Depth
0
5
10
15
Position
0
50
100
150
200
TdelSat96−58.fa_003 Read1: Tinf_No_Andean_1.fq.gz Read2: Tinf_No_Andean_2.fq.gz
Depth
0
5
10
15
20
Position
0
50
100
150
200

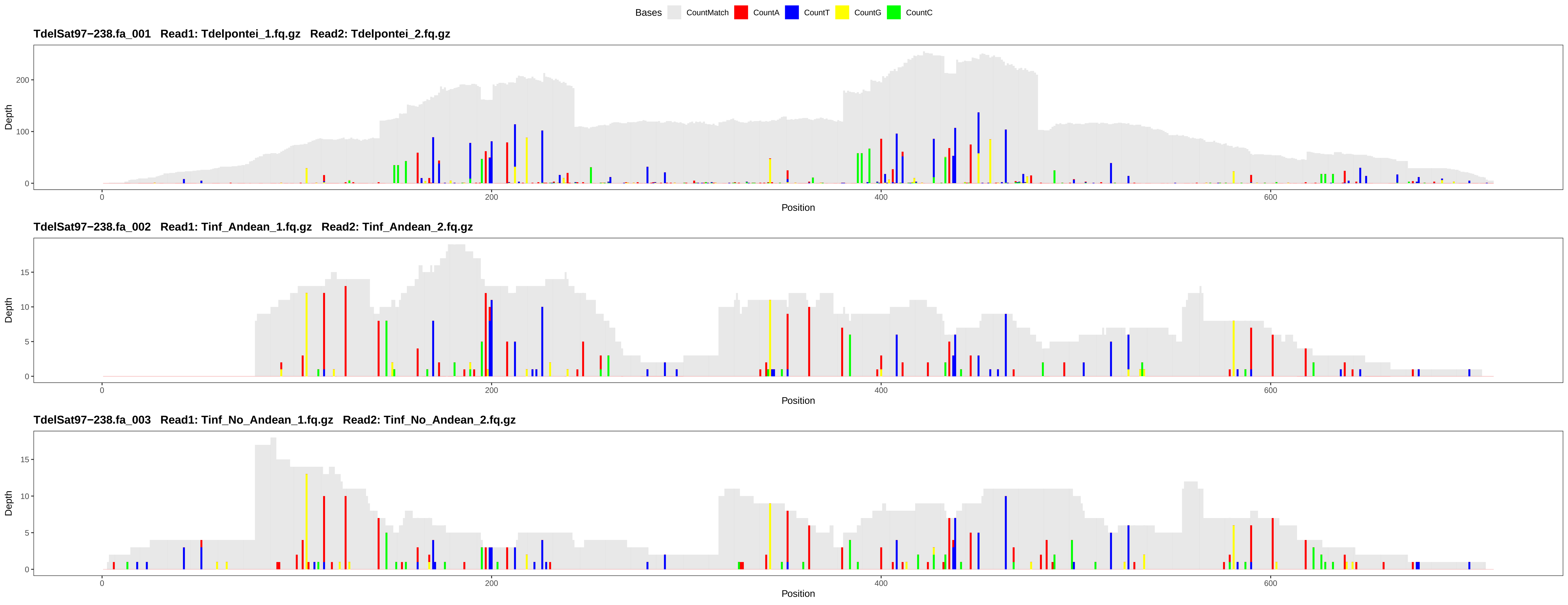
Bases CountMatch CountA CountT CountG CountC
TdelSat97−238.fa_001 Read1: Tdelpontei_1.fq.gz Read2: Tdelpontei_2.fq.gz
TdelSat97−238.fa_002 Read1: Tinf_Andean_1.fq.gz Read2: Tinf_Andean_2.fq.gz
TdelSat97−238.fa_003 Read1: Tinf_No_Andean_1.fq.gz Read2: Tinf_No_Andean_2.fq.gz
Depth
Position
0
200
400
600
100
5
10
15

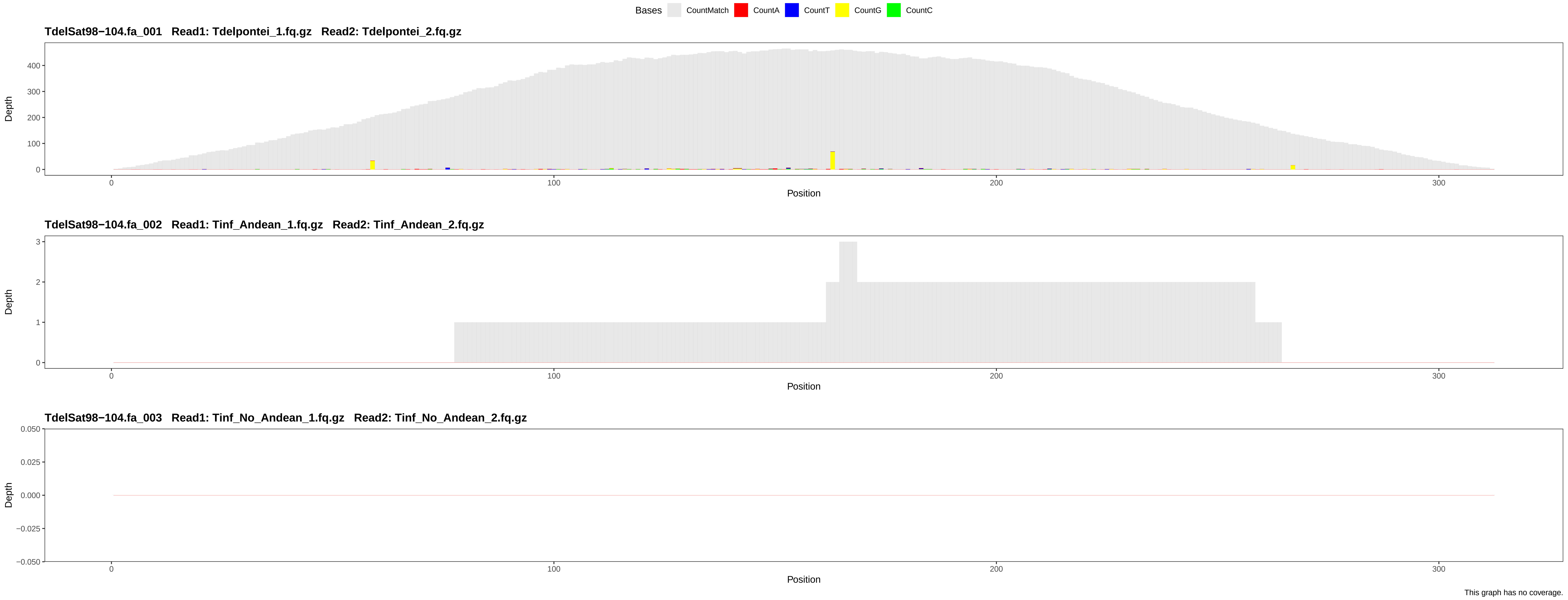

Bases
CountMatch
CountA
CountT
CountG
CountC
TdelSat98−104.fa_001 Read1: Tdelpontei_1.fq.gz Read2: Tdelpontei_2.fq.gz
Depth
Position
TdelSat98−104.fa_002 Read1: Tinf_Andean_1.fq.gz Read2: Tinf_Andean_2.fq.gz
Depth
Position
TdelSat98−104.fa_003 Read1: Tinf_No_Andean_1.fq.gz Read2: Tinf_No_Andean_2.fq.gz
Depth
Position
This graph has no coverage.

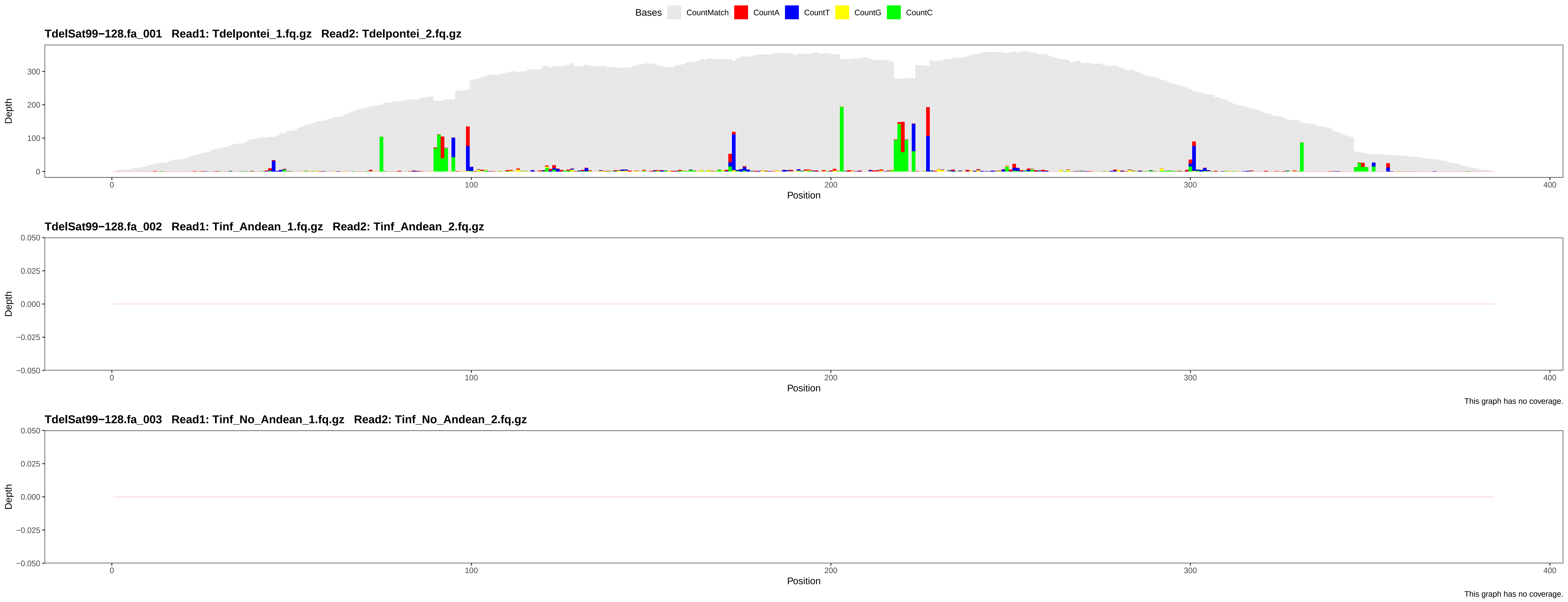
Bases CountMatch CountA CountT CountG CountC
TdelSat99−128.fa_001 Read1: Tdelpontei_1.fq.gz Read2: Tdelpontei_2.fq.gz
Depth
Position
TdelSat99−128.fa_002 Read1: Tinf_Andean_1.fq.gz Read2: Tinf_Andean_2.fq.gz
This graph has no coverage.
TdelSat99−128.fa_003 Read1: Tinf_No_Andean_1.fq.gz Read2: Tinf_No_Andean_2.fq.gz
This graph has no coverage.

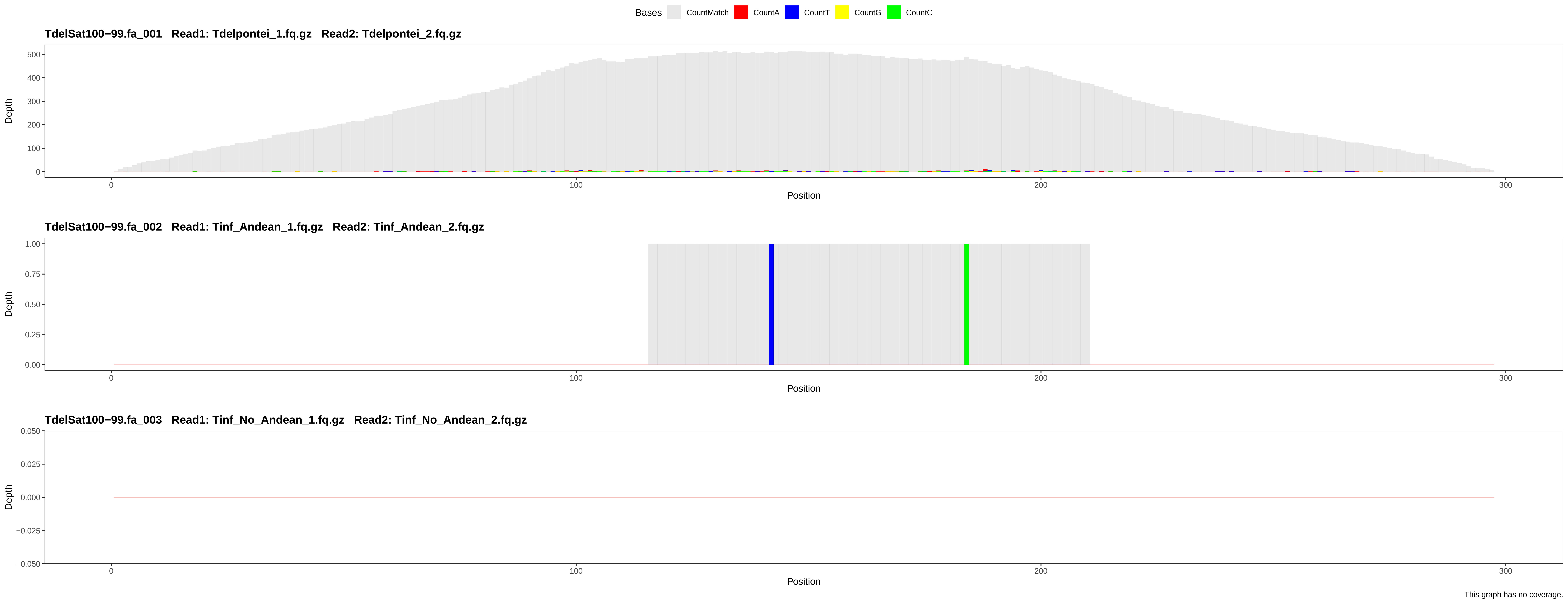
Bases CountMatch CountA CountT CountG CountC
TdelSat100−99.fa_001 Read1: Tdelpontei_1.fq.gz Read2: Tdelpontei_2.fq.gz
Depth
Position
TdelSat100−99.fa_002 Read1: Tinf_Andean_1.fq.gz Read2: Tinf_Andean_2.fq.gz
Depth
Position
TdelSat100−99.fa_003 Read1: Tinf_No_Andean_1.fq.gz Read2: Tinf_No_Andean_2.fq.gz
Depth
Position
This graph has no coverage.

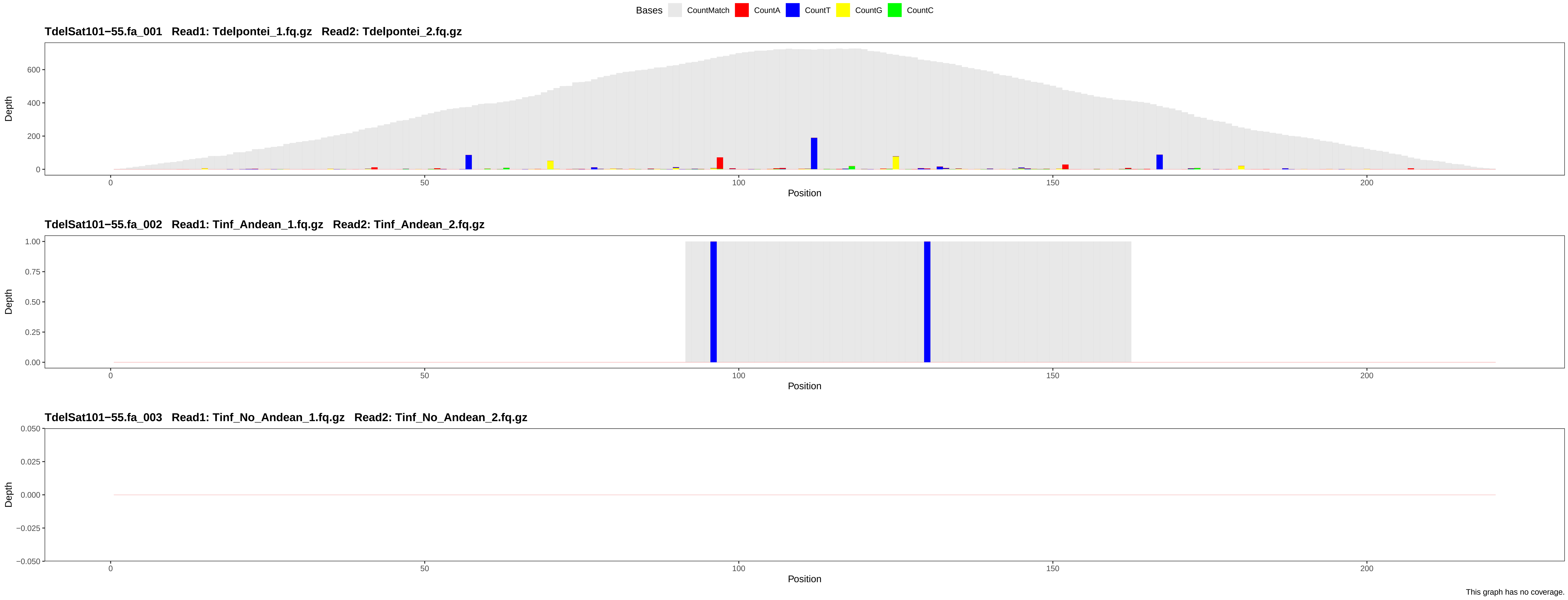
Bases
CountMatch
CountA
CountT
CountG
CountC
TdelSat101−55.fa_001 Read1: Tdelpontei_1.fq.gz Read2: Tdelpontei_2.fq.gz
Depth
Position
TdelSat101−55.fa_002 Read1: Tinf_Andean_1.fq.gz Read2: Tinf_Andean_2.fq.gz
Depth
Position
TdelSat101−55.fa_003 Read1: Tinf_No_Andean_1.fq.gz Read2: Tinf_No_Andean_2.fq.gz
Depth
Position
This graph has no coverage.

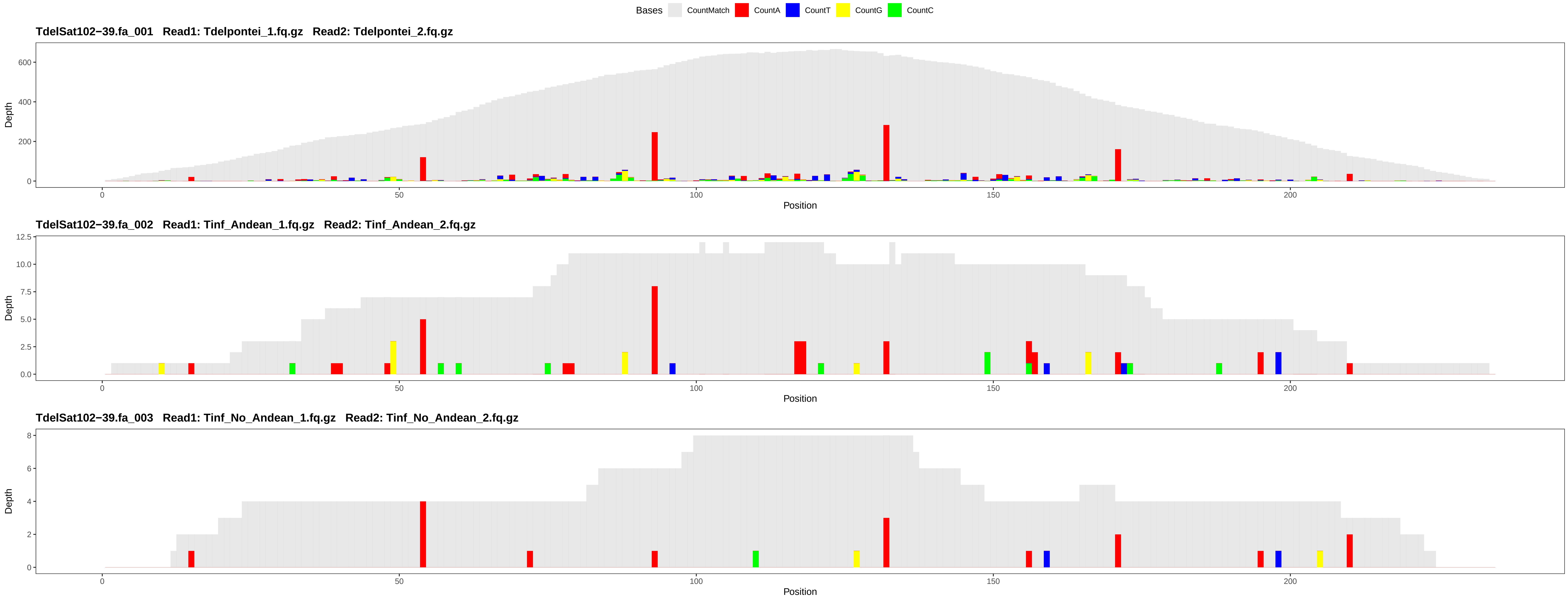

Bases
CountMatch
CountA
CountT
CountG
CountC
TdelSat102−39.fa_001 Read1: Tdelpontei_1.fq.gz Read2: Tdelpontei_2.fq.gz
Depth
Position
TdelSat102−39.fa_002 Read1: Tinf_Andean_1.fq.gz Read2: Tinf_Andean_2.fq.gz
Depth
Position
TdelSat102−39.fa_003 Read1: Tinf_No_Andean_1.fq.gz Read2: Tinf_No_Andean_2.fq.gz
Depth
Position

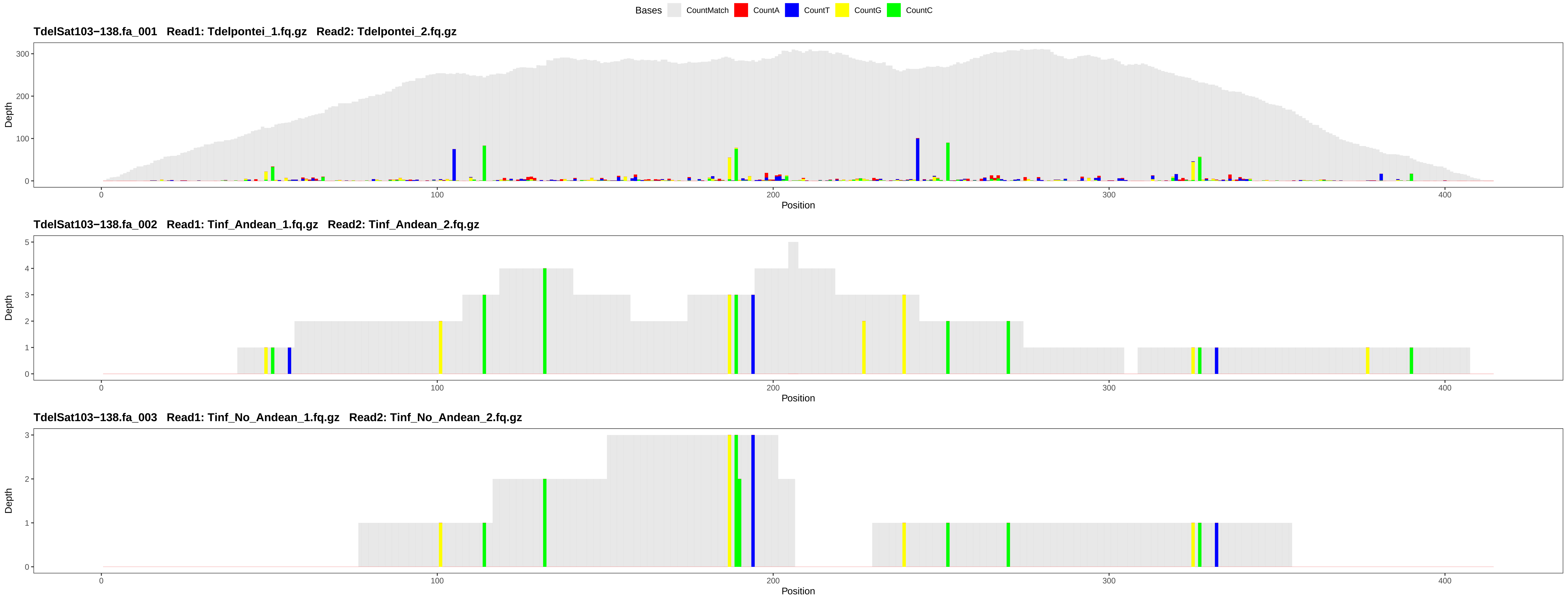

Bases CountMatch CountA CountT CountG CountC
TdelSat103−138.fa_001 Read1: Tdelpontei_1.fq.gz Read2: Tdelpontei_2.fq.gz
Depth
Position
TdelSat103−138.fa_002 Read1: Tinf_Andean_1.fq.gz Read2: Tinf_Andean_2.fq.gz
Depth
Position
TdelSat103−138.fa_003 Read1: Tinf_No_Andean_1.fq.gz Read2: Tinf_No_Andean_2.fq.gz
Depth
Position

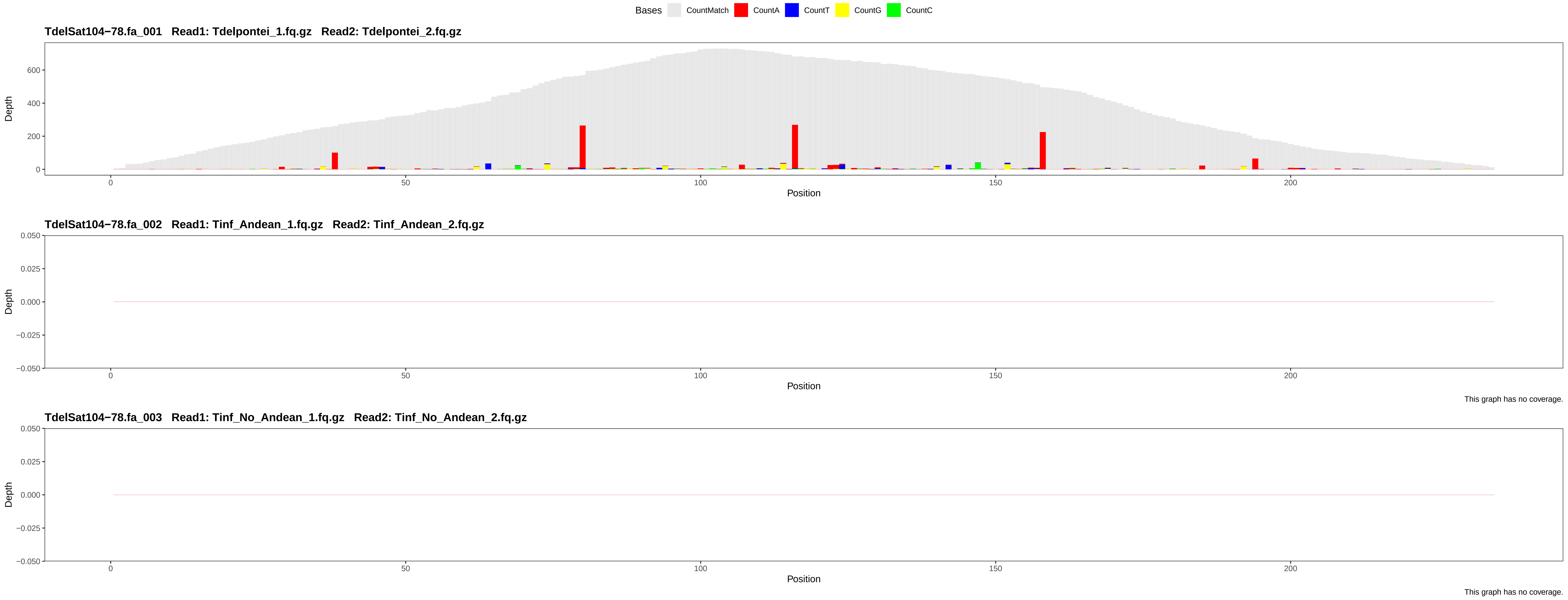
Bases CountMatch CountA CountT CountG CountC
TdelSat104−78.fa_001 Read1: Tdelpontei_1.fq.gz Read2: Tdelpontei_2.fq.gz
Depth
600
400
200
0
0
50
100
150
200
Position
TdelSat104−78.fa_002 Read1: Tinf_Andean_1.fq.gz Read2: Tinf_Andean_2.fq.gz
Depth
0.050
0.025
0.000
−0.025
−0.050
0
50
100
150
200
Position
This graph has no coverage.
TdelSat104−78.fa_003 Read1: Tinf_No_Andean_1.fq.gz Read2: Tinf_No_Andean_2.fq.gz
Depth
0.050
0.025
0.000
−0.025
−0.050
0
50
100
150
200
Position
This graph has no coverage.

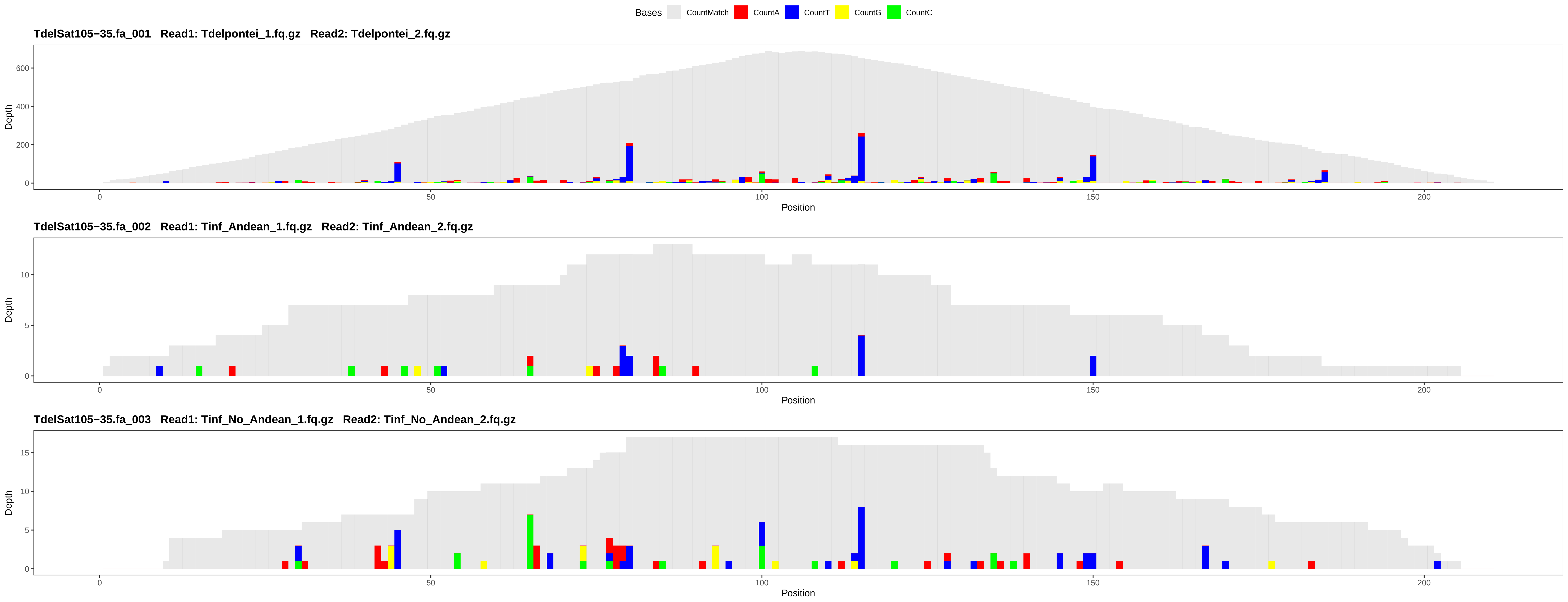
Bases CountMatch CountA CountT CountG CountC
TdelSat105−35.fa_001 Read1: Tdelpontei_1.fq.gz Read2: Tdelpontei_2.fq.gz
Depth
0
200
400
600
0
50
100
150
200
Position
TdelSat105−35.fa_002 Read1: Tinf_Andean_1.fq.gz Read2: Tinf_Andean_2.fq.gz
Depth
0
5
10
Position
TdelSat105−35.fa_003 Read1: Tinf_No_Andean_1.fq.gz Read2: Tinf_No_Andean_2.fq.gz
Depth
0
5
10
15
Position

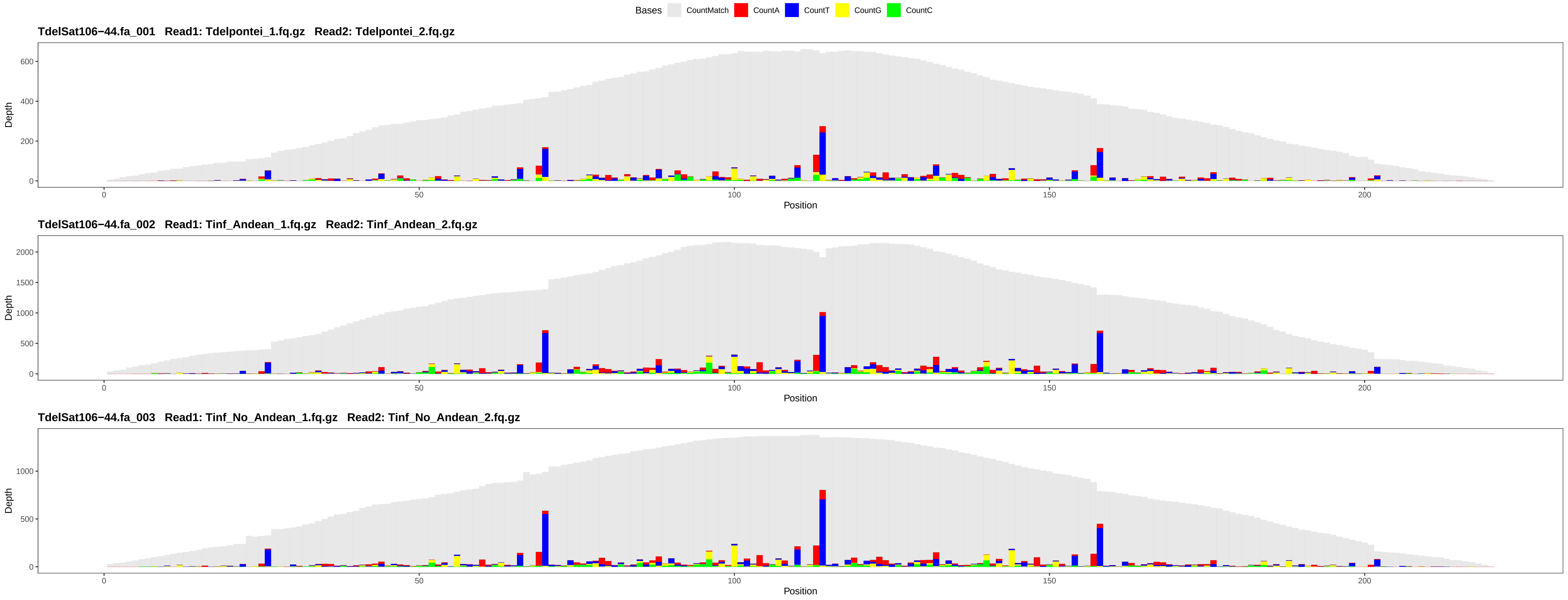
Bases CountMatch CountA CountT CountG CountC
TdelSat106−44.fa_001 Read1: Tdelpontei_1.fq.gz Read2: Tdelpontei_2.fq.gz
Depth
0
200
400
600
Position
0
50
100
150
200
TdelSat106−44.fa_002 Read1: Tinf_Andean_1.fq.gz Read2: Tinf_Andean_2.fq.gz
Depth
0
500
1000
1500
2000
Position
TdelSat106−44.fa_003 Read1: Tinf_No_Andean_1.fq.gz Read2: Tinf_No_Andean_2.fq.gz
Depth
0
500
1000
Position

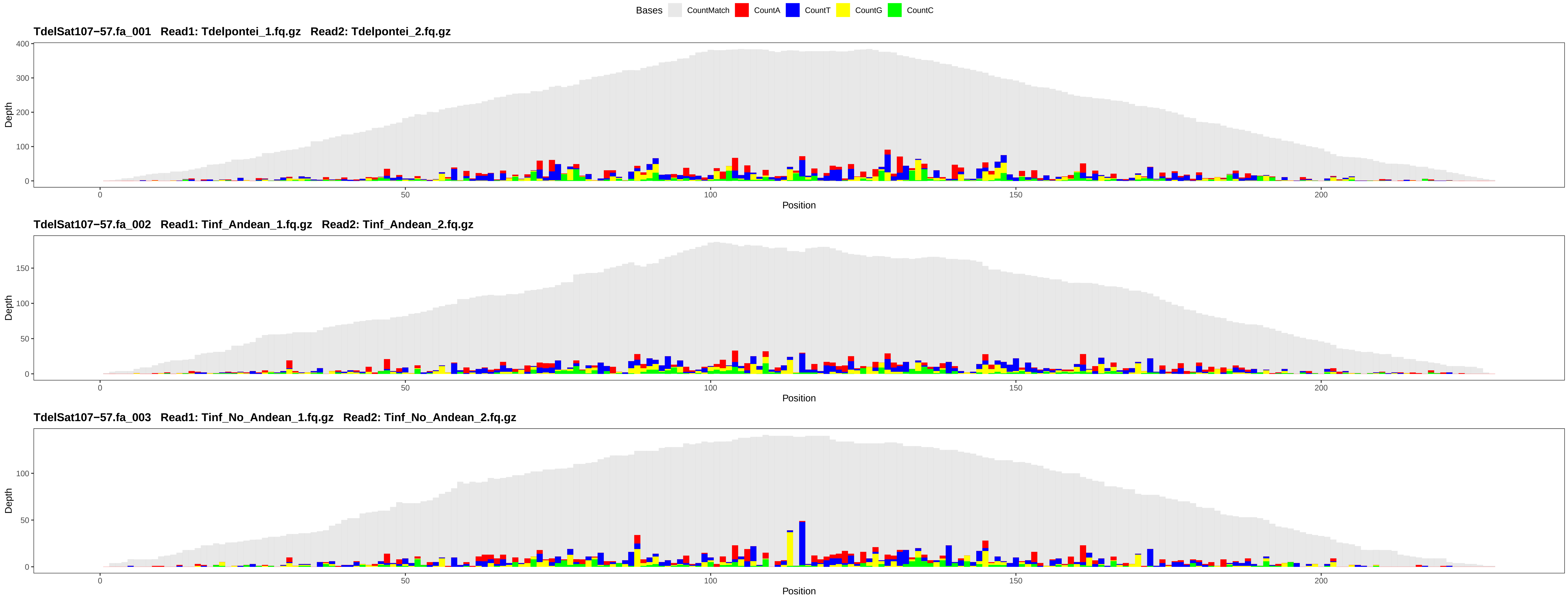

Bases CountMatch CountA CountT CountG CountC
TdelSat107−57.fa_001 Read1: Tdelpontei_1.fq.gz Read2: Tdelpontei_2.fq.gz
Depth
Position
TdelSat107−57.fa_002 Read1: Tinf_Andean_1.fq.gz Read2: Tinf_Andean_2.fq.gz
Depth
Position
TdelSat107−57.fa_003 Read1: Tinf_No_Andean_1.fq.gz Read2: Tinf_No_Andean_2.fq.gz
Depth
Position

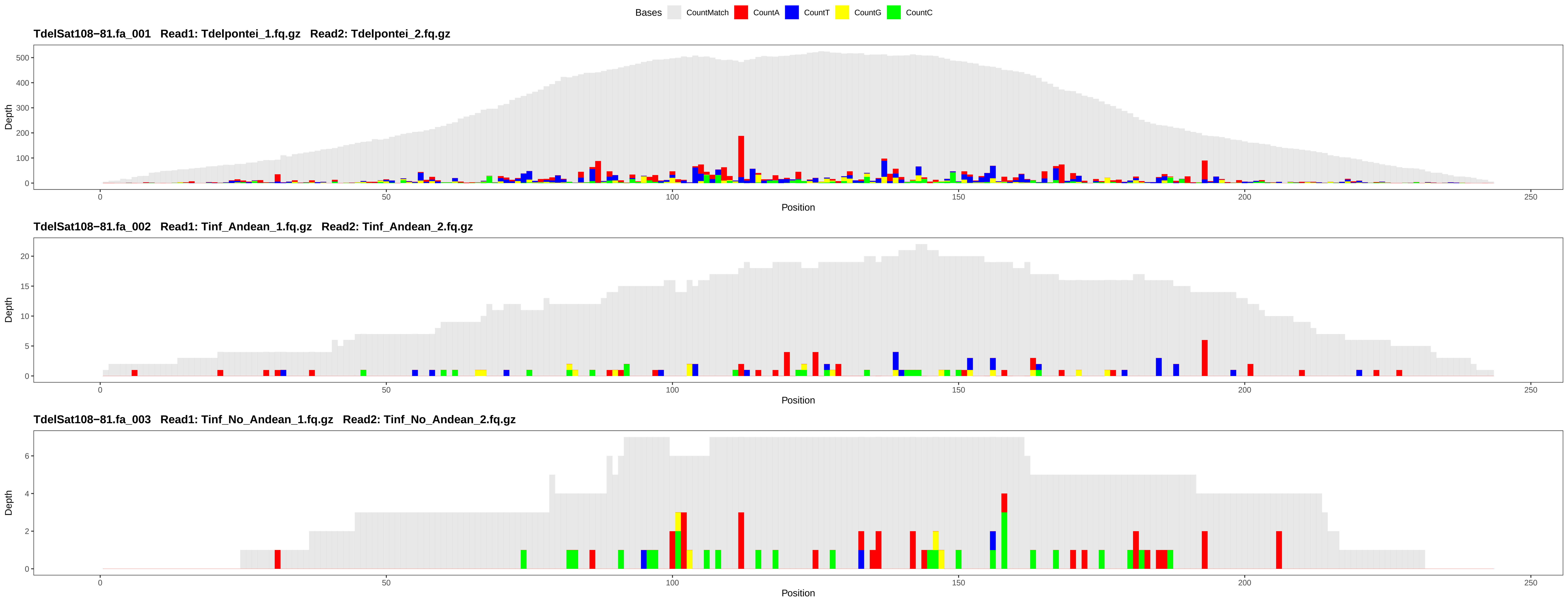

Bases
CountMatch
CountA
CountT
CountG
CountC
TdelSat108−81.fa_001 Read1: Tdelpontei_1.fq.gz Read2: Tdelpontei_2.fq.gz
Depth
Position
TdelSat108−81.fa_002 Read1: Tinf_Andean_1.fq.gz Read2: Tinf_Andean_2.fq.gz
Depth
Position
TdelSat108−81.fa_003 Read1: Tinf_No_Andean_1.fq.gz Read2: Tinf_No_Andean_2.fq.gz
Depth
Position

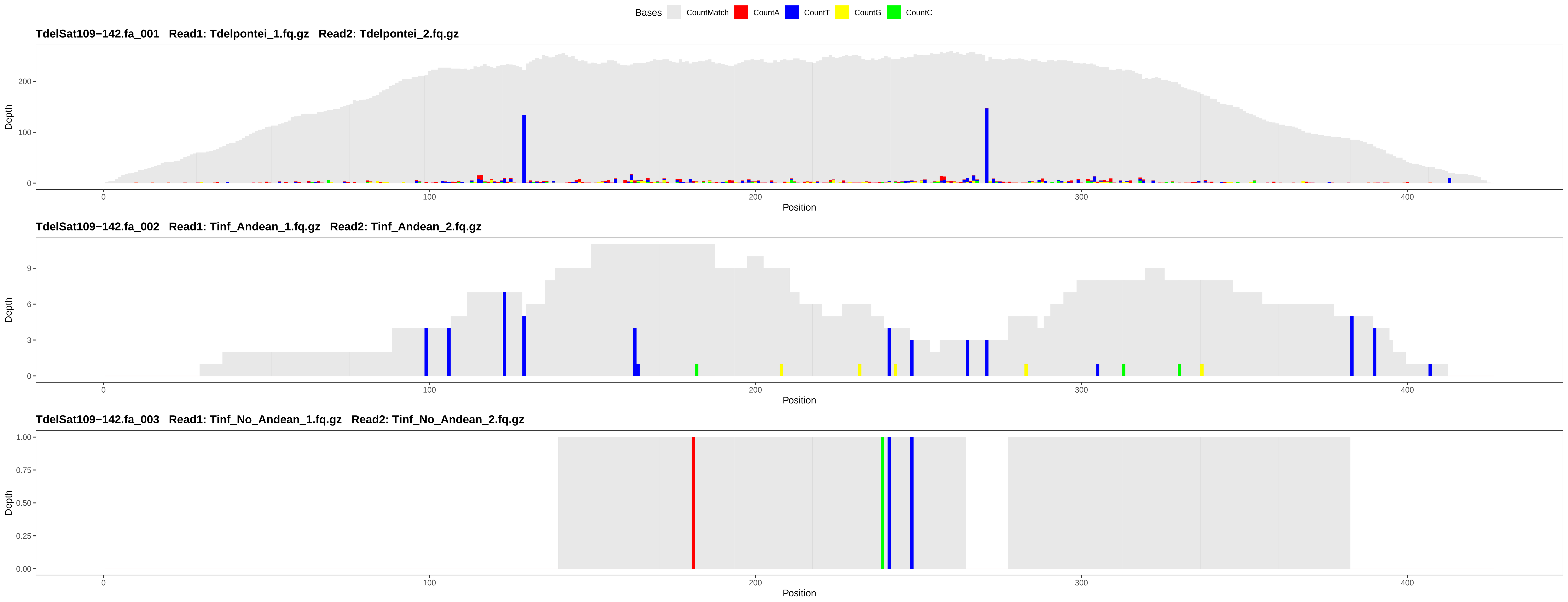
Bases
CountMatch
CountA
CountT
CountG
CountC
TdelSat109−142.fa_001   Read1: Tdelpontei_1.fq.gz   Read2: Tdelpontei_2.fq.gz
Depth
200
100
0
0
100
200
300
400
Position
TdelSat109−142.fa_002   Read1: Tinf_Andean_1.fq.gz   Read2: Tinf_Andean_2.fq.gz
Depth
9
6
3
0
0
100
200
300
400
Position
TdelSat109−142.fa_003   Read1: Tinf_No_Andean_1.fq.gz   Read2: Tinf_No_Andean_2.fq.gz
Depth
1.00
0.75
0.50
0.25
0.00
0
100
200
300
400
Position

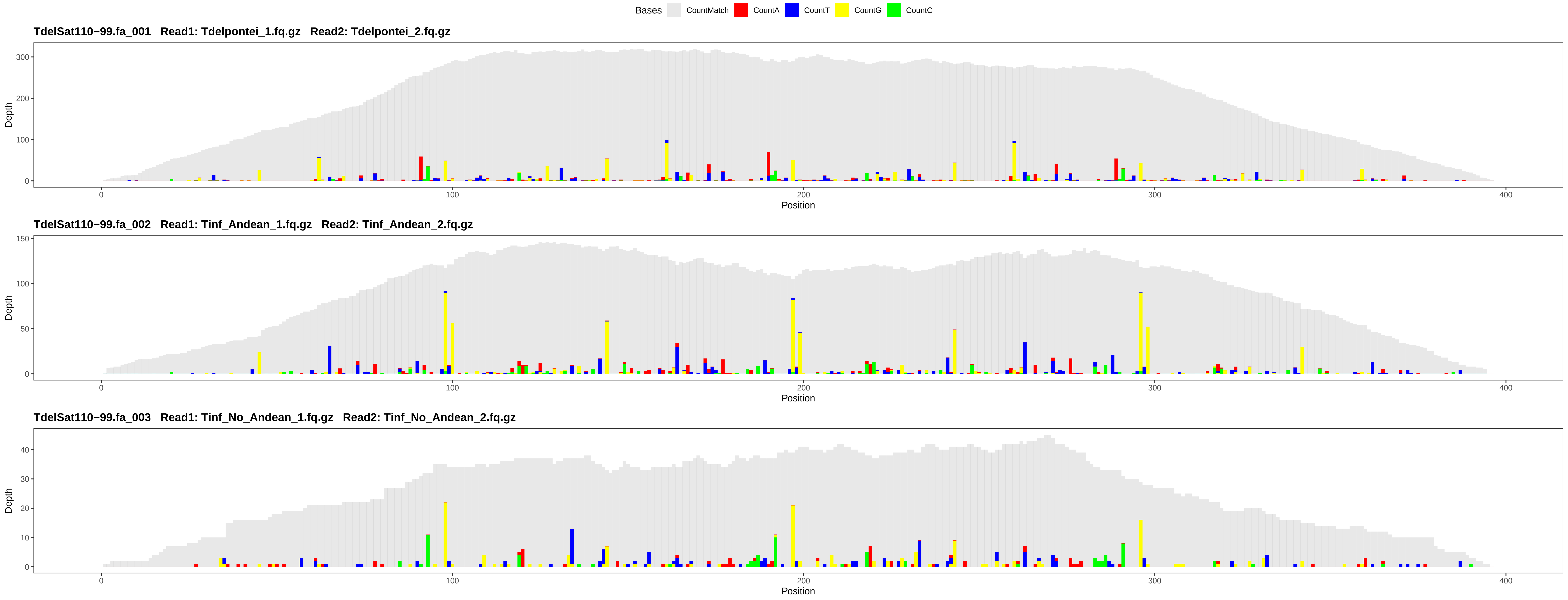

Bases CountMatch CountA CountT CountG CountC
TdelSat110−99.fa_001 Read1: Tdelpontei_1.fq.gz Read2: Tdelpontei_2.fq.gz
Depth
0
100
200
300
Position
0
100
200
300
400
TdelSat110−99.fa_002 Read1: Tinf_Andean_1.fq.gz Read2: Tinf_Andean_2.fq.gz
Depth
0
50
100
150
Position
0
100
200
300
400
TdelSat110−99.fa_003 Read1: Tinf_No_Andean_1.fq.gz Read2: Tinf_No_Andean_2.fq.gz
Depth
0
10
20
30
40
Position
0
100
200
300
400

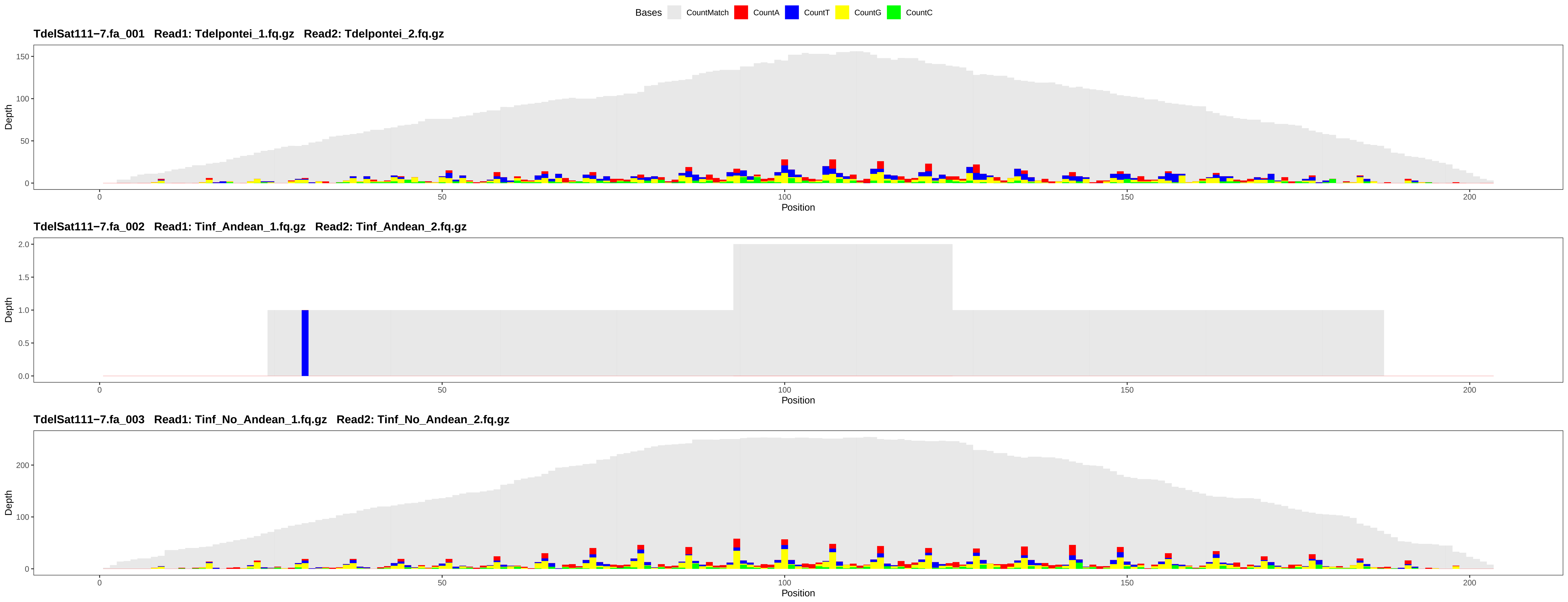
Bases CountMatch CountA CountT CountG CountC
TdelSat111−7.fa_001 Read1: Tdelpontei_1.fq.gz Read2: Tdelpontei_2.fq.gz
Depth
Position
TdelSat111−7.fa_002 Read1: Tinf_Andean_1.fq.gz Read2: Tinf_Andean_2.fq.gz
Depth
Position
TdelSat111−7.fa_003 Read1: Tinf_No_Andean_1.fq.gz Read2: Tinf_No_Andean_2.fq.gz
Depth
Position

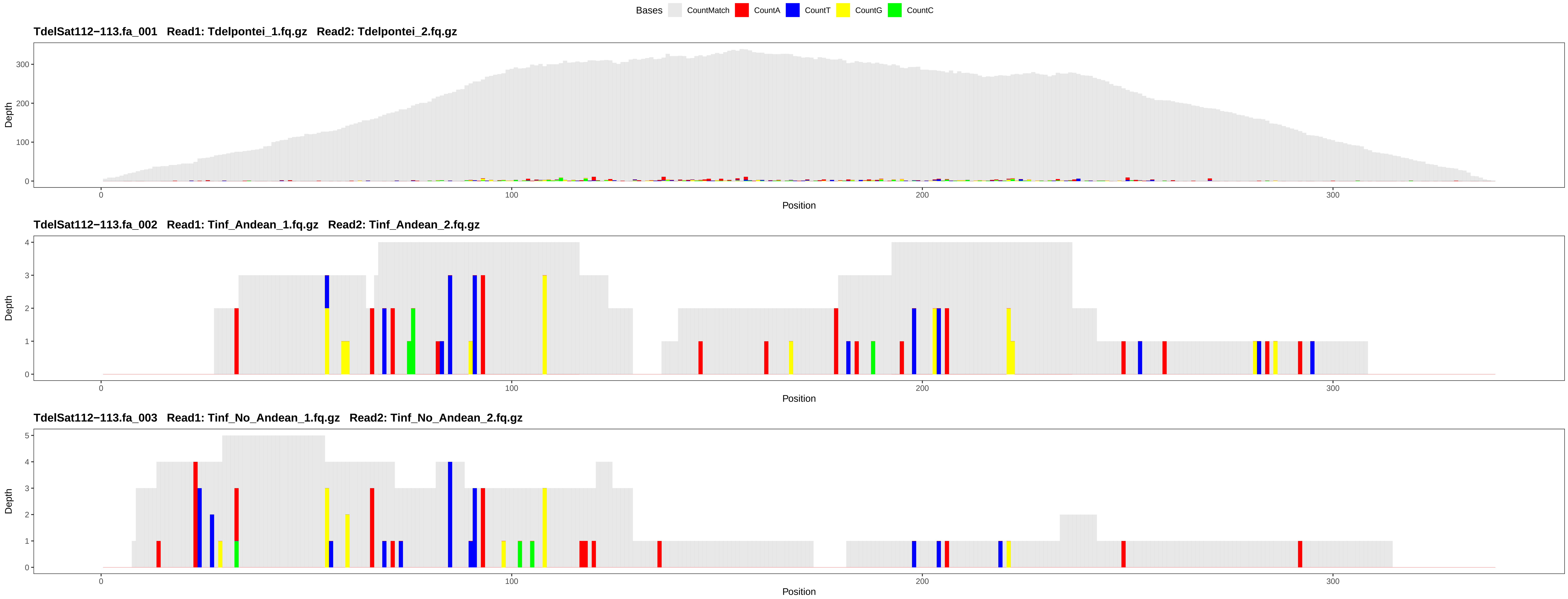

Bases
CountMatch
CountA
CountT
CountG
CountC
TdelSat112−113.fa_001 Read1: Tdelpontei_1.fq.gz Read2: Tdelpontei_2.fq.gz
Depth
Position
TdelSat112−113.fa_002 Read1: Tinf_Andean_1.fq.gz Read2: Tinf_Andean_2.fq.gz
Depth
Position
TdelSat112−113.fa_003 Read1: Tinf_No_Andean_1.fq.gz Read2: Tinf_No_Andean_2.fq.gz
Depth
Position

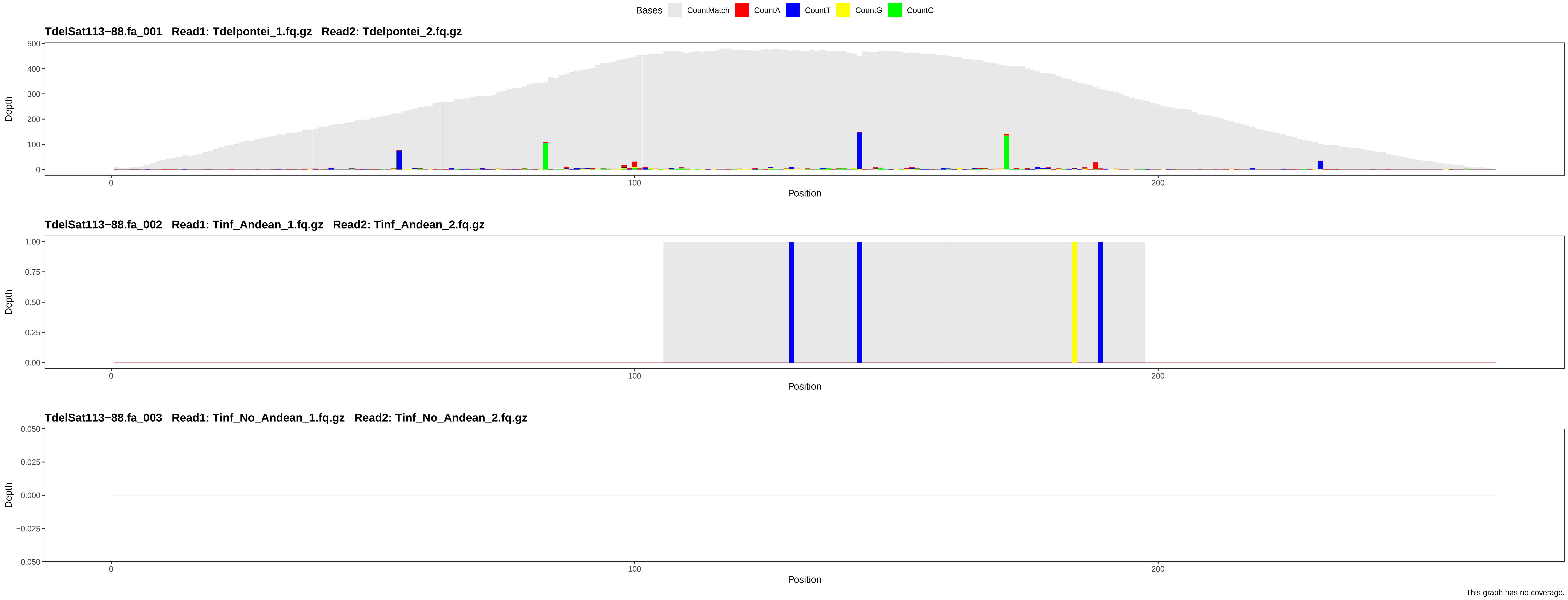

Bases CountMatch CountA CountT CountG CountC
TdelSat113−88.fa_001 Read1: Tdelpontei_1.fq.gz Read2: Tdelpontei_2.fq.gz
Depth
Position
TdelSat113−88.fa_002 Read1: Tinf_Andean_1.fq.gz Read2: Tinf_Andean_2.fq.gz
Depth
Position
TdelSat113−88.fa_003 Read1: Tinf_No_Andean_1.fq.gz Read2: Tinf_No_Andean_2.fq.gz
Depth
Position
This graph has no coverage.

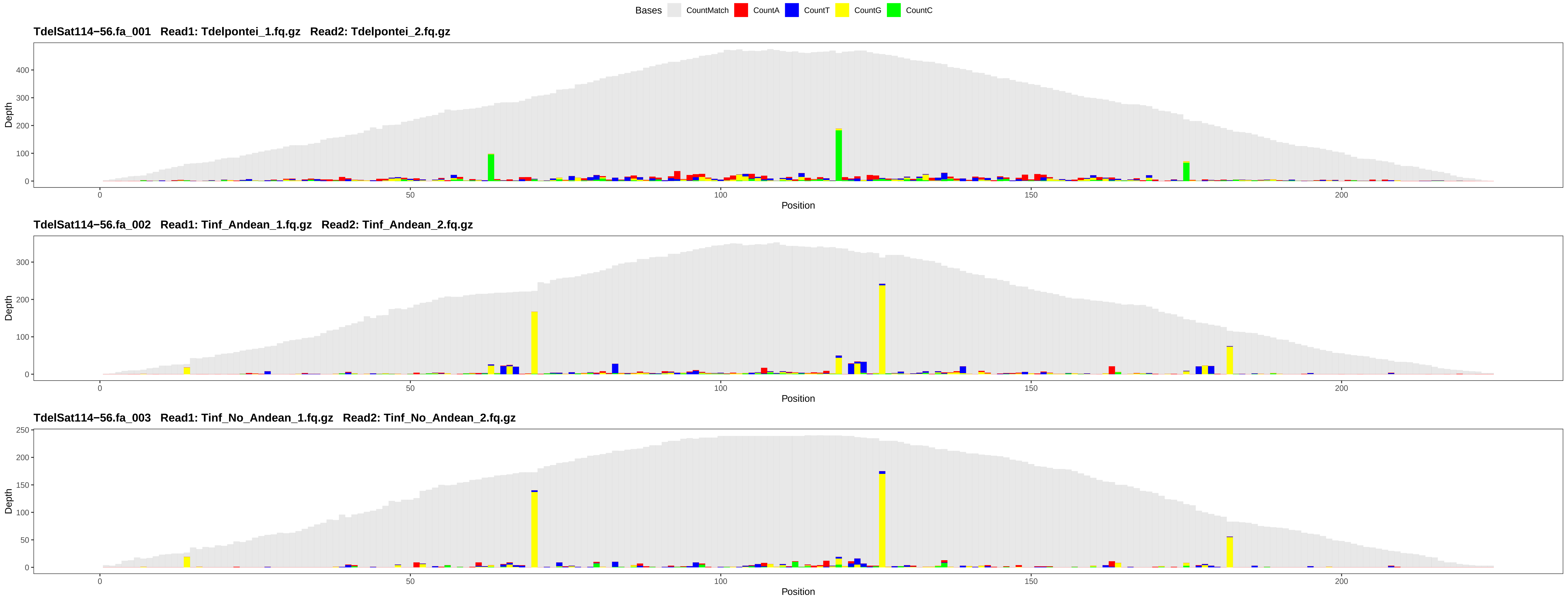
Bases CountMatch CountA CountT CountG CountC
TdelSat114−56.fa_001 Read1: Tdelpontei_1.fq.gz Read2: Tdelpontei_2.fq.gz
Depth
Position
TdelSat114−56.fa_002 Read1: Tinf_Andean_1.fq.gz Read2: Tinf_Andean_2.fq.gz
Depth
Position
TdelSat114−56.fa_003 Read1: Tinf_No_Andean_1.fq.gz Read2: Tinf_No_Andean_2.fq.gz
Depth
Position

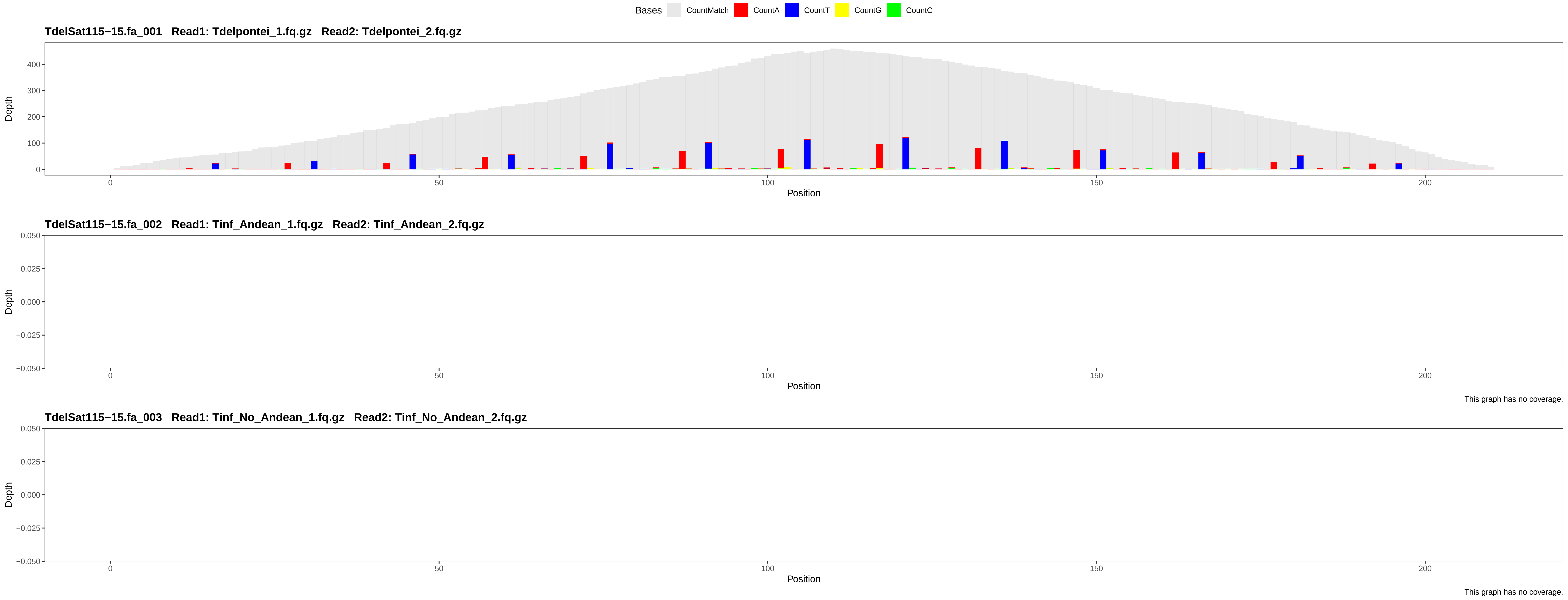
Bases CountMatch CountA CountT CountG CountC
TdelSat115−15.fa_001 Read1: Tdelpontei_1.fq.gz Read2: Tdelpontei_2.fq.gz
Depth
Position
TdelSat115−15.fa_002 Read1: Tinf_Andean_1.fq.gz Read2: Tinf_Andean_2.fq.gz
Depth
Position
This graph has no coverage.
TdelSat115−15.fa_003 Read1: Tinf_No_Andean_1.fq.gz Read2: Tinf_No_Andean_2.fq.gz
Depth
Position
This graph has no coverage.

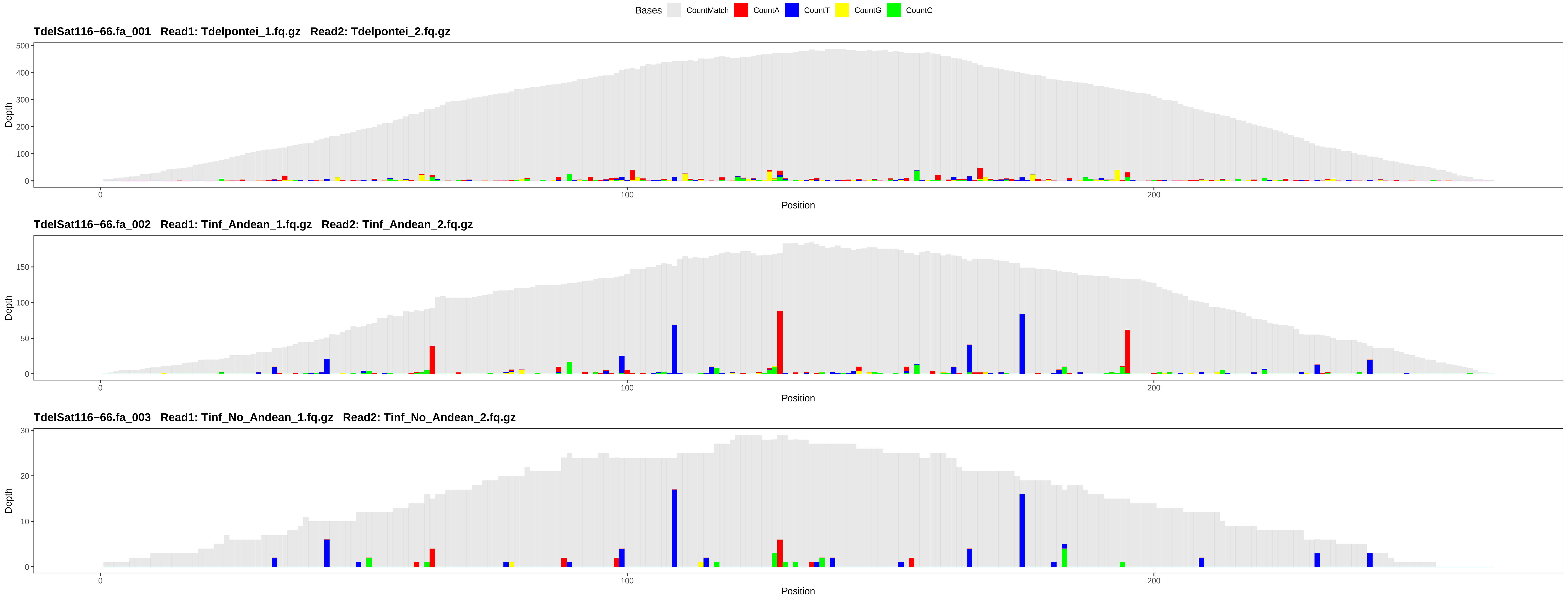
Bases
CountMatch
CountA
CountT
CountG
CountC
TdelSat116−66.fa_001 Read1: Tdelpontei_1.fq.gz Read2: Tdelpontei_2.fq.gz
Depth
500
400
300
200
100
0
0
100
200
Position
TdelSat116−66.fa_002 Read1: Tinf_Andean_1.fq.gz Read2: Tinf_Andean_2.fq.gz
Depth
150
100
50
0
0
100
200
Position
TdelSat116−66.fa_003 Read1: Tinf_No_Andean_1.fq.gz Read2: Tinf_No_Andean_2.fq.gz
Depth
30
20
10
0
0
100
200
Position

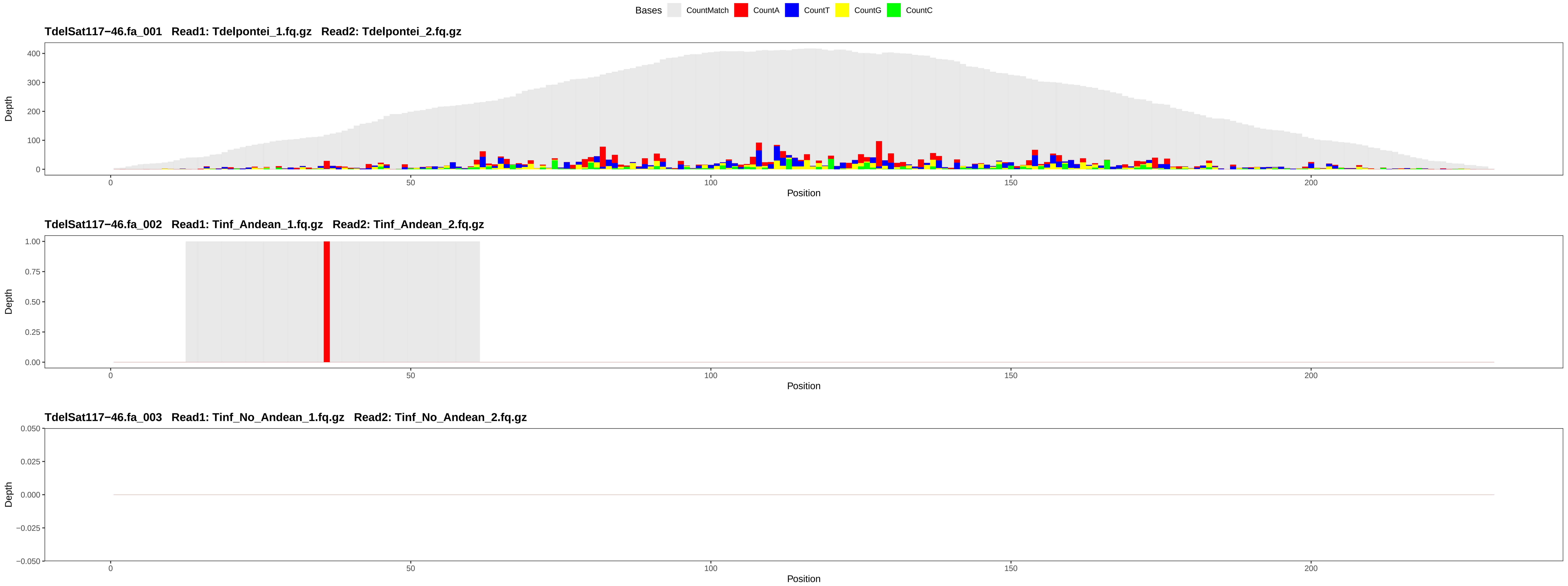

This graph has no coverage.

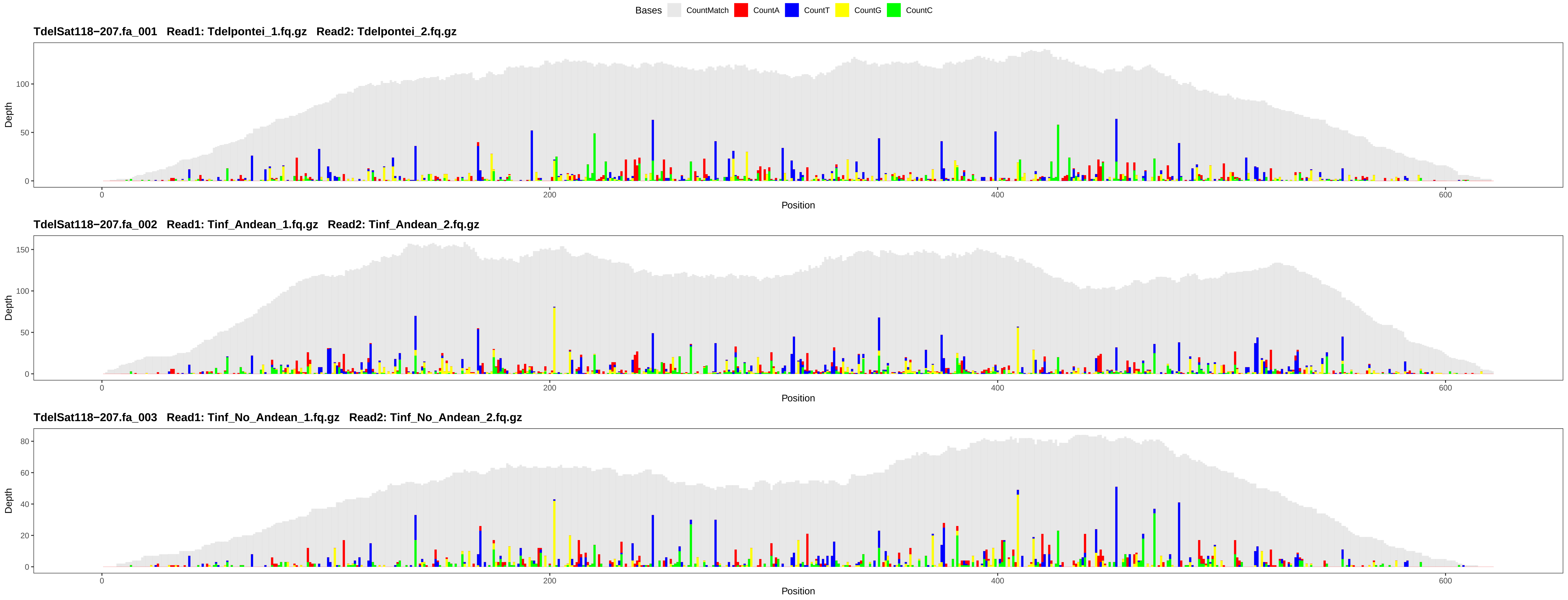
Bases CountMatch CountA CountT CountG CountC
TdelSat118−207.fa_001 Read1: Tdelpontei_1.fq.gz Read2: Tdelpontei_2.fq.gz
Depth
0
50
100
Position
0
200
400
600
TdelSat118−207.fa_002 Read1: Tinf_Andean_1.fq.gz Read2: Tinf_Andean_2.fq.gz
Depth
0
50
100
150
Position
TdelSat118−207.fa_003 Read1: Tinf_No_Andean_1.fq.gz Read2: Tinf_No_Andean_2.fq.gz
Depth
0
20
40
60
80
Position

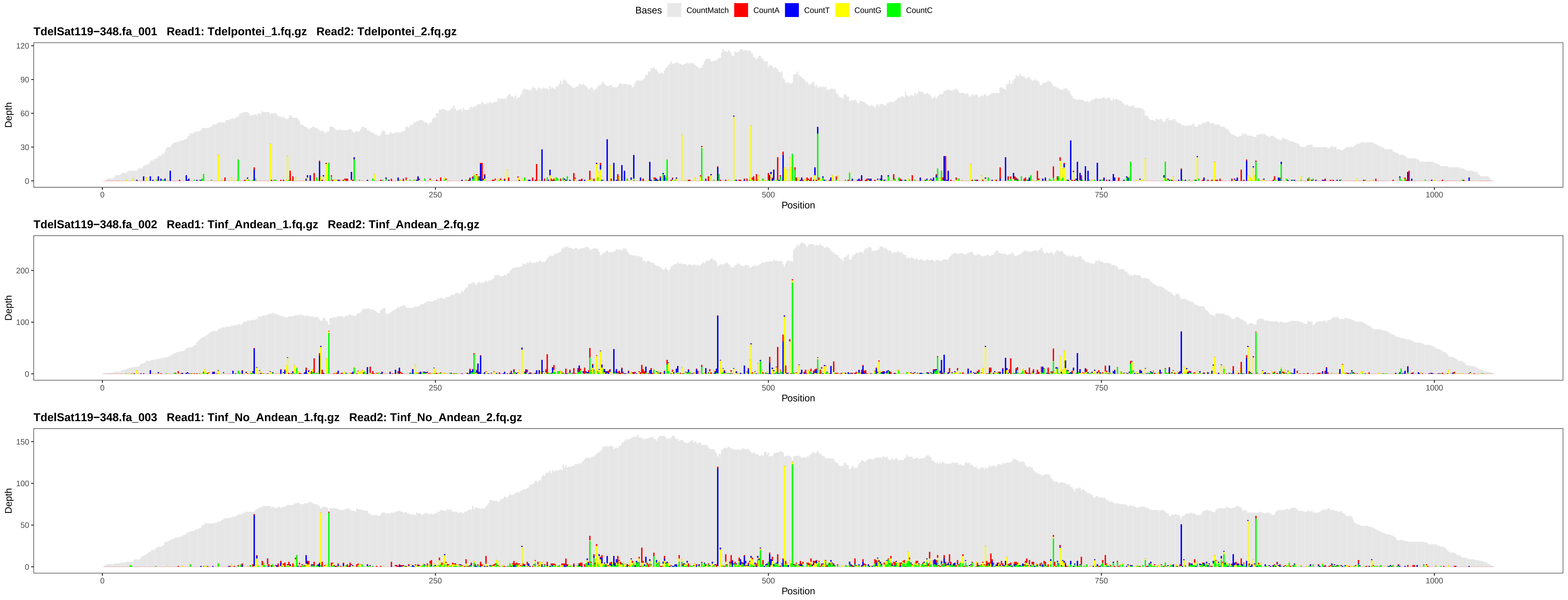

Bases
CountMatch
CountA
CountT
CountG
CountC
TdelSat119−348.fa_001 Read1: Tdelpontei_1.fq.gz Read2: Tdelpontei_2.fq.gz
Depth
120
90
60
30
0
0
250
500
750
1000
Position
TdelSat119−348.fa_002 Read1: Tinf_Andean_1.fq.gz Read2: Tinf_Andean_2.fq.gz
Depth
200
100
0
0
250
500
750
1000
Position
TdelSat119−348.fa_003 Read1: Tinf_No_Andean_1.fq.gz Read2: Tinf_No_Andean_2.fq.gz
Depth
150
100
50
0
0
250
500
750
1000
Position

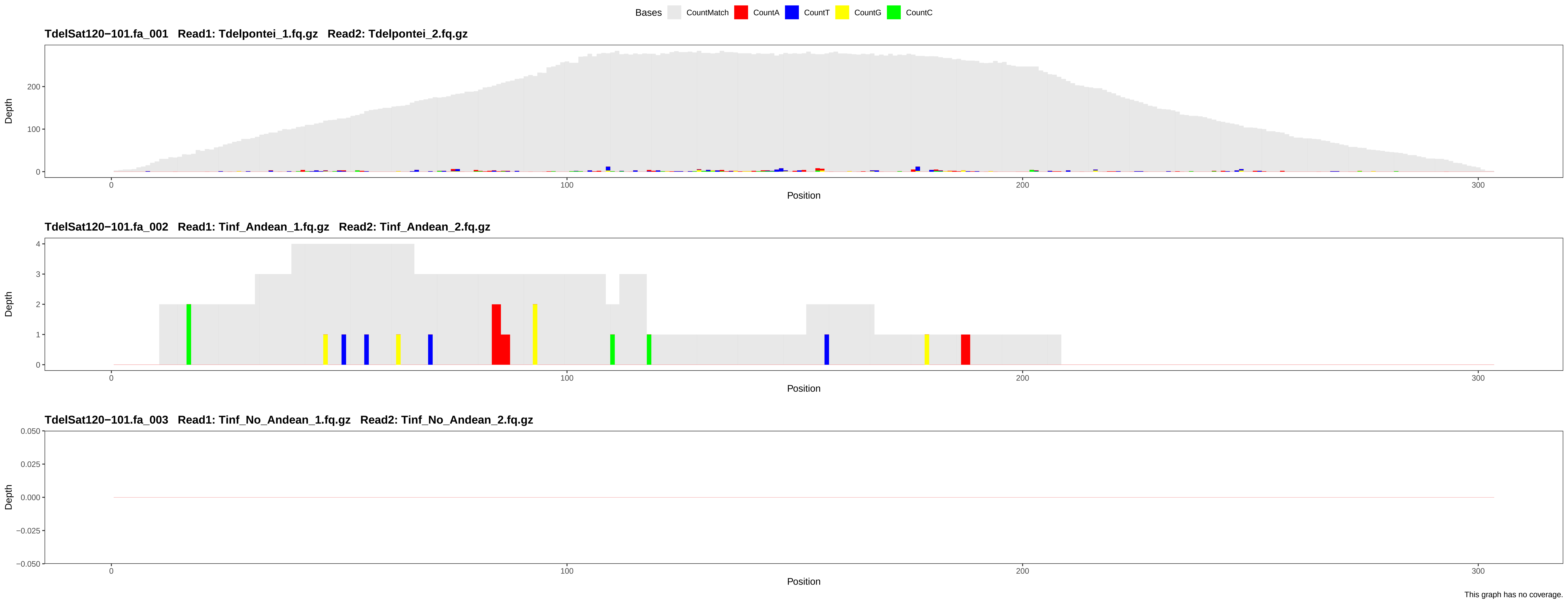
Bases CountMatch CountA CountT CountG CountC
TdelSat120−101.fa_001 Read1: Tdelpontei_1.fq.gz Read2: Tdelpontei_2.fq.gz
Depth
Position
TdelSat120−101.fa_002 Read1: Tinf_Andean_1.fq.gz Read2: Tinf_Andean_2.fq.gz
Depth
Position
TdelSat120−101.fa_003 Read1: Tinf_No_Andean_1.fq.gz Read2: Tinf_No_Andean_2.fq.gz
Depth
Position
This graph has no coverage.

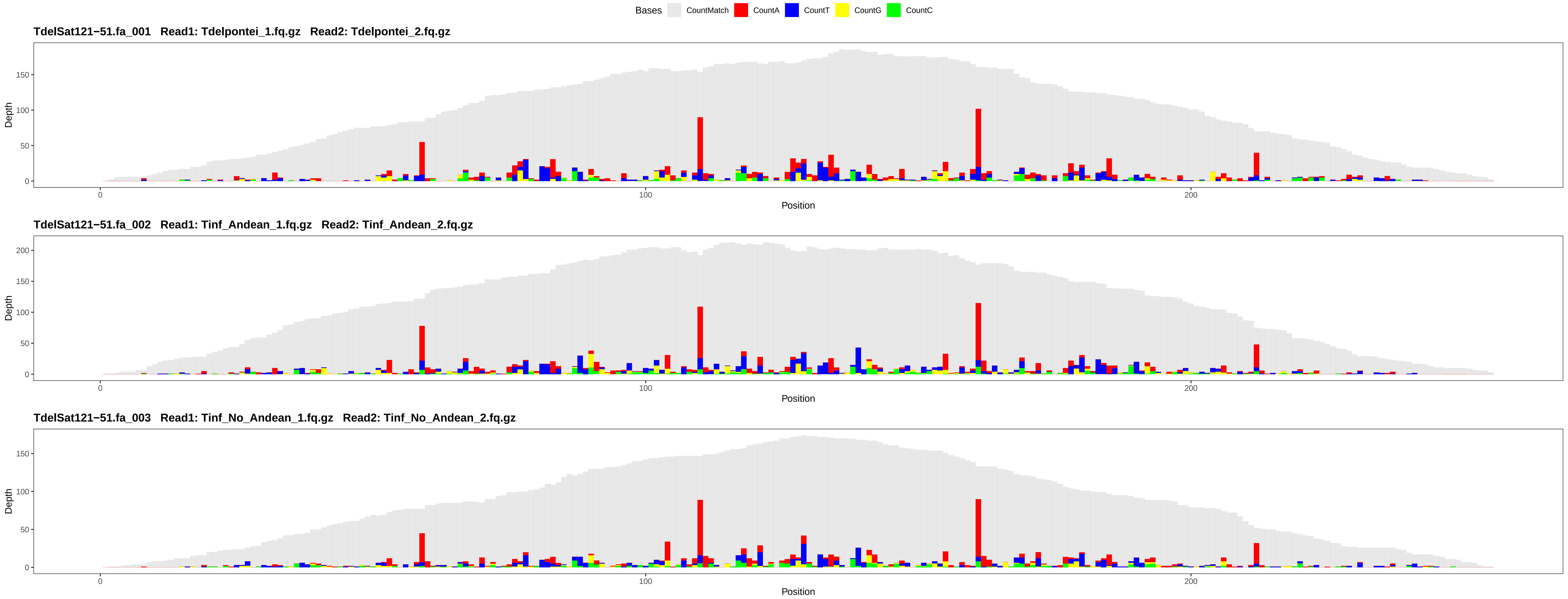
Bases CountMatch CountA CountT CountG CountC
TdelSat121−51.fa_001 Read1: Tdelpontei_1.fq.gz Read2: Tdelpontei_2.fq.gz
Depth
0
50
100
150
0
100
200
Position
TdelSat121−51.fa_002 Read1: Tinf_Andean_1.fq.gz Read2: Tinf_Andean_2.fq.gz
Depth
0
50
100
150
200
0
100
200
Position
TdelSat121−51.fa_003 Read1: Tinf_No_Andean_1.fq.gz Read2: Tinf_No_Andean_2.fq.gz
Depth
0
50
100
150
0
100
200
Position

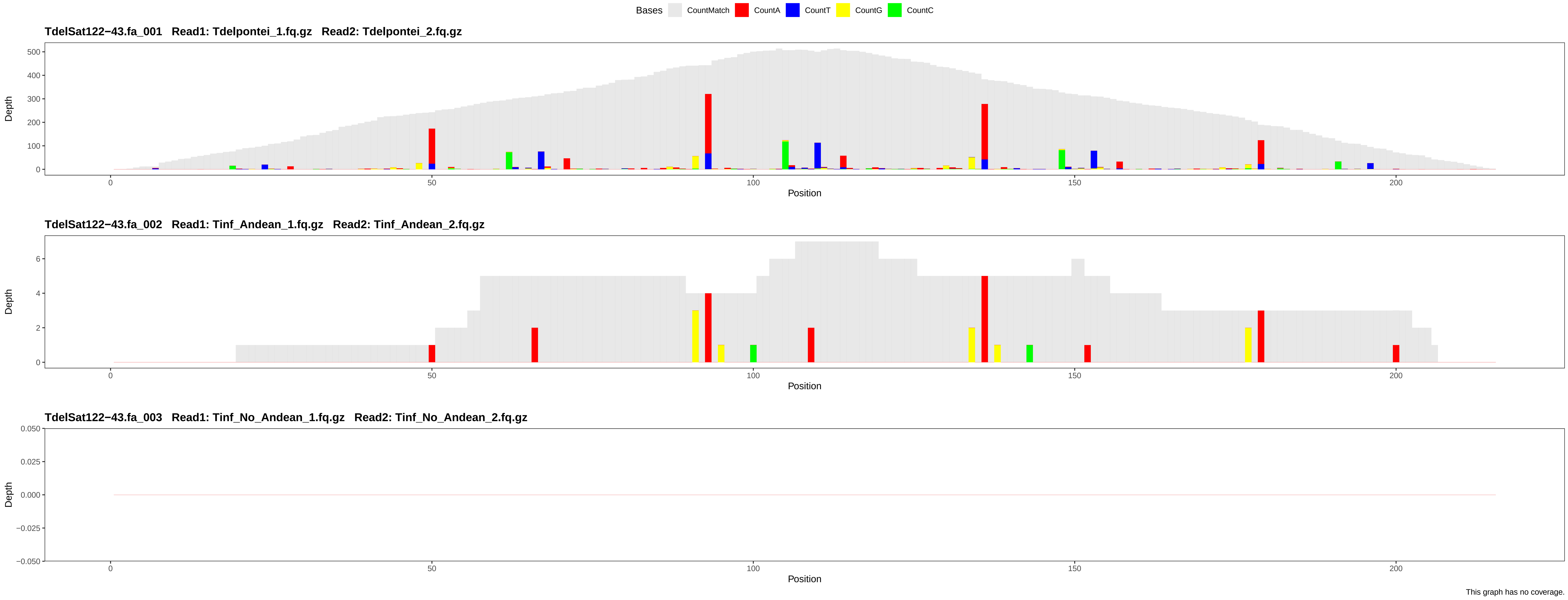

Bases CountMatch CountA CountT CountG CountC
TdelSat122−43.fa_001 Read1: Tdelpontei_1.fq.gz Read2: Tdelpontei_2.fq.gz
Depth
Position
TdelSat122−43.fa_002 Read1: Tinf_Andean_1.fq.gz Read2: Tinf_Andean_2.fq.gz
Depth
Position
TdelSat122−43.fa_003 Read1: Tinf_No_Andean_1.fq.gz Read2: Tinf_No_Andean_2.fq.gz
Depth
Position
This graph has no coverage.

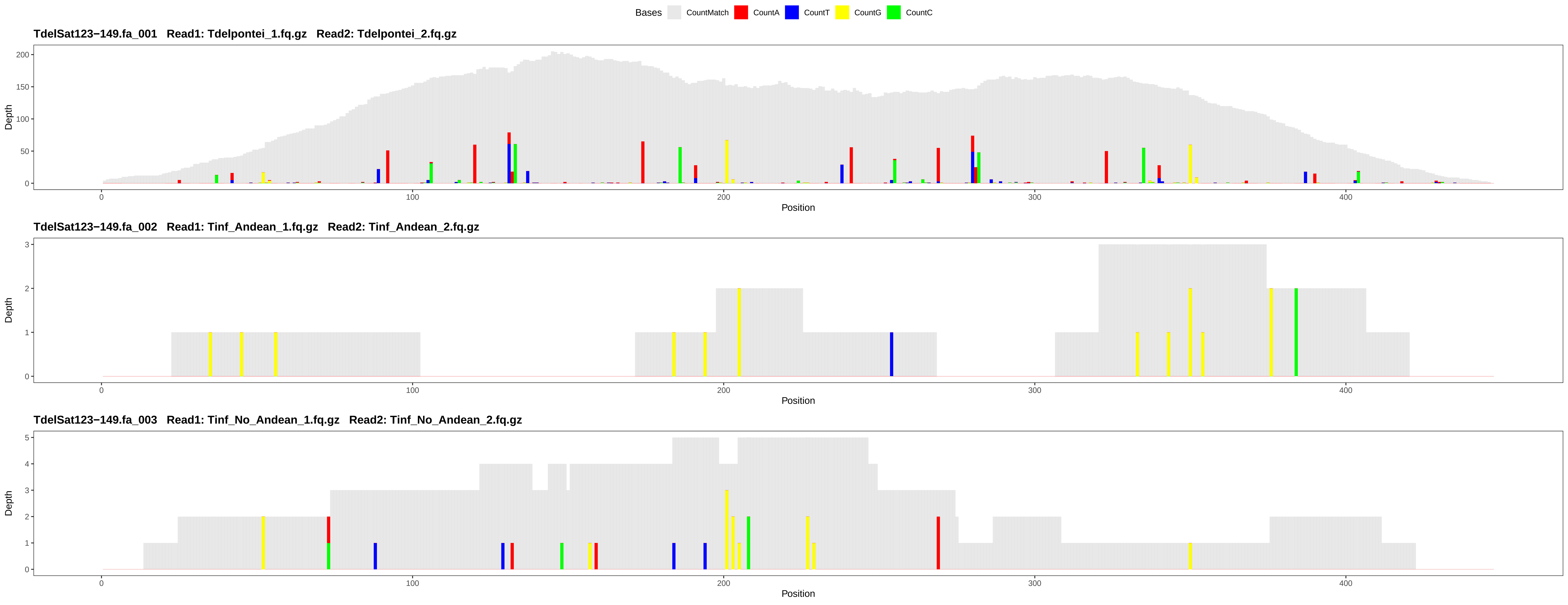
Bases
CountMatch
CountA
CountT
CountG
CountC
TdelSat123−149.fa_001 Read1: Tdelpontei_1.fq.gz Read2: Tdelpontei_2.fq.gz
Depth
Position
TdelSat123−149.fa_002 Read1: Tinf_Andean_1.fq.gz Read2: Tinf_Andean_2.fq.gz
Depth
Position
TdelSat123−149.fa_003 Read1: Tinf_No_Andean_1.fq.gz Read2: Tinf_No_Andean_2.fq.gz
Depth
Position

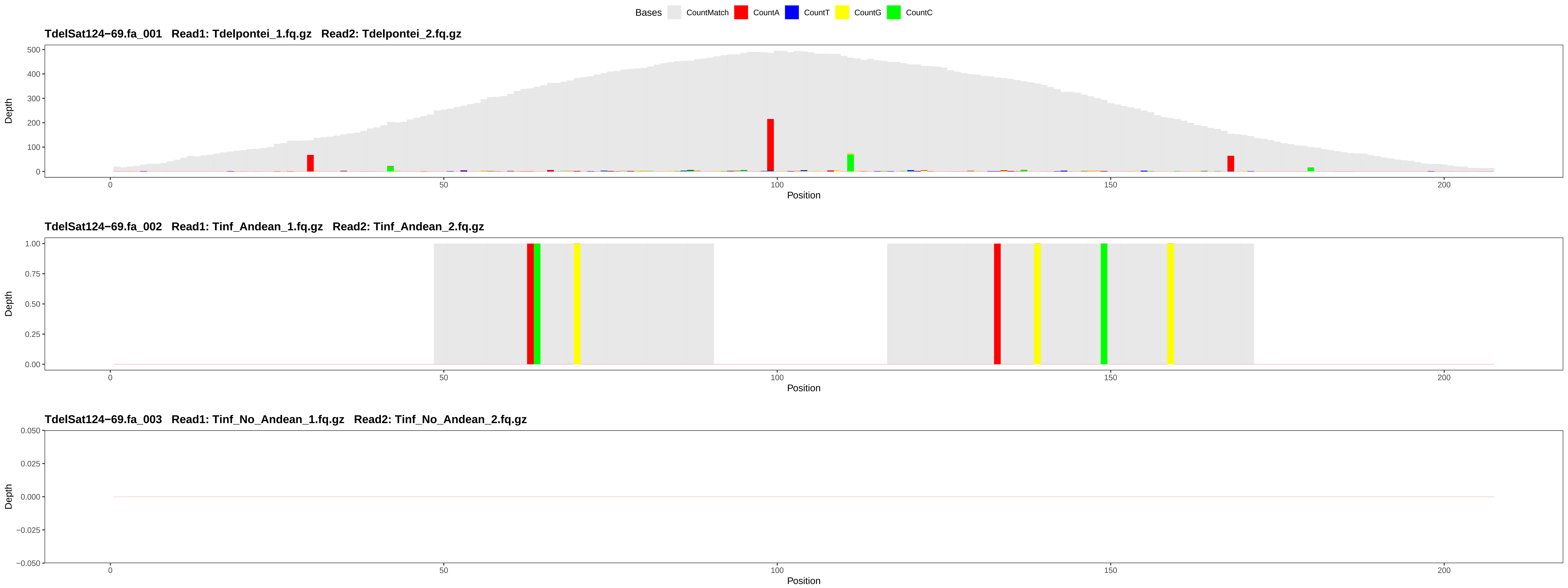

This graph has no coverage.

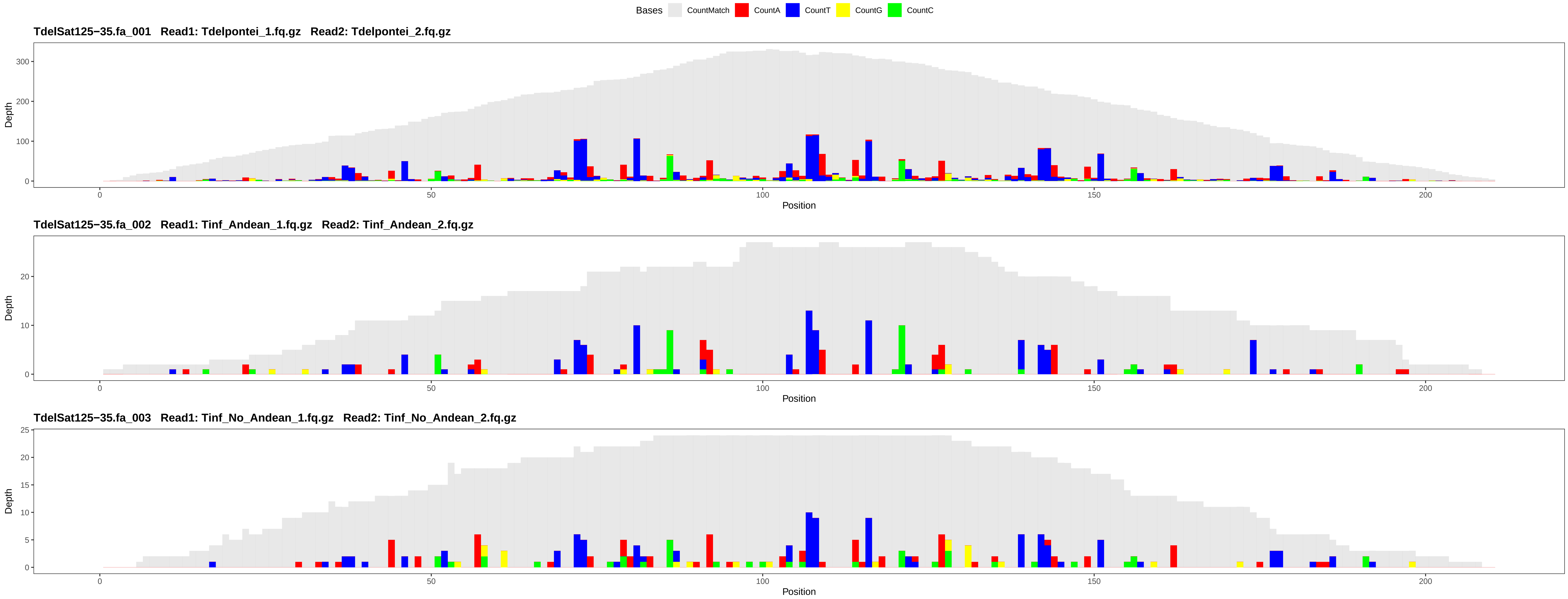
Bases CountMatch CountA CountT CountG CountC
TdelSat125−35.fa_001 Read1: Tdelpontei_1.fq.gz Read2: Tdelpontei_2.fq.gz
TdelSat125−35.fa_002 Read1: Tinf_Andean_1.fq.gz Read2: Tinf_Andean_2.fq.gz
TdelSat125−35.fa_003 Read1: Tinf_No_Andean_1.fq.gz Read2: Tinf_No_Andean_2.fq.gz
Depth
Position

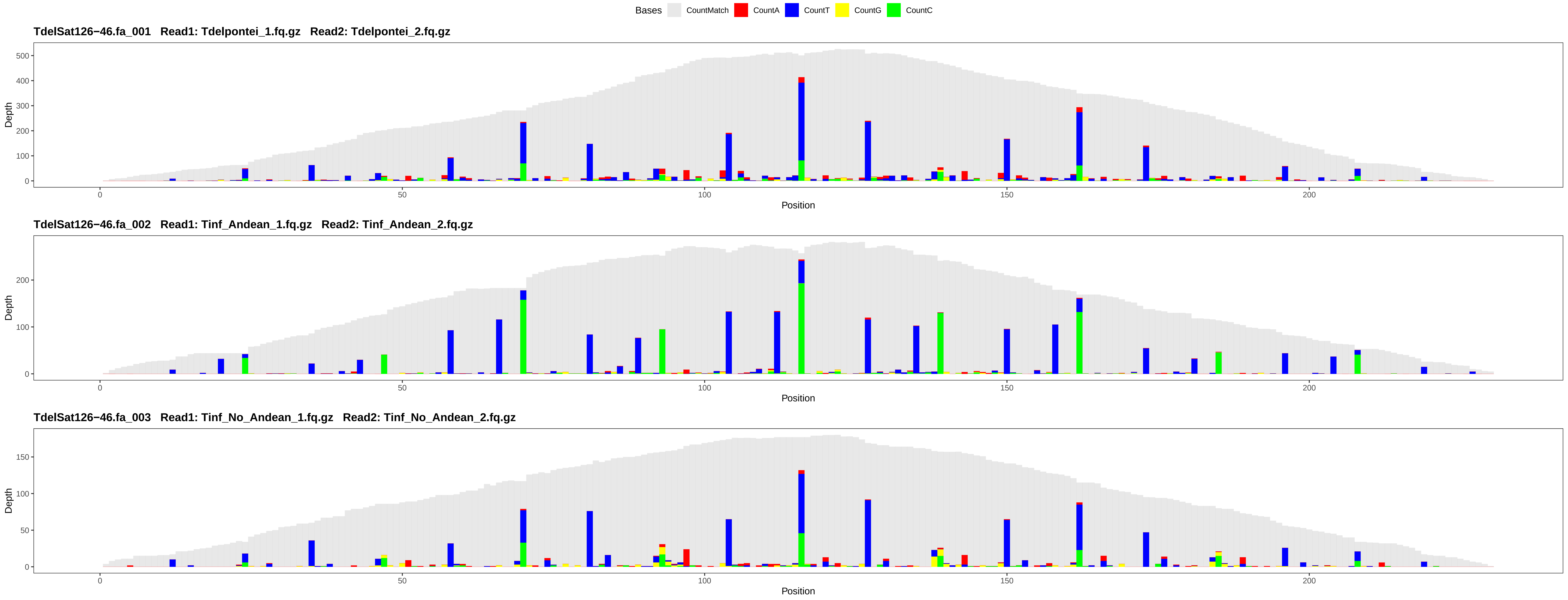

Bases CountMatch CountA CountT CountG CountC
TdelSat126−46.fa_001 Read1: Tdelpontei_1.fq.gz Read2: Tdelpontei_2.fq.gz
Depth
Position
TdelSat126−46.fa_002 Read1: Tinf_Andean_1.fq.gz Read2: Tinf_Andean_2.fq.gz
Depth
Position
TdelSat126−46.fa_003 Read1: Tinf_No_Andean_1.fq.gz Read2: Tinf_No_Andean_2.fq.gz
Depth
Position

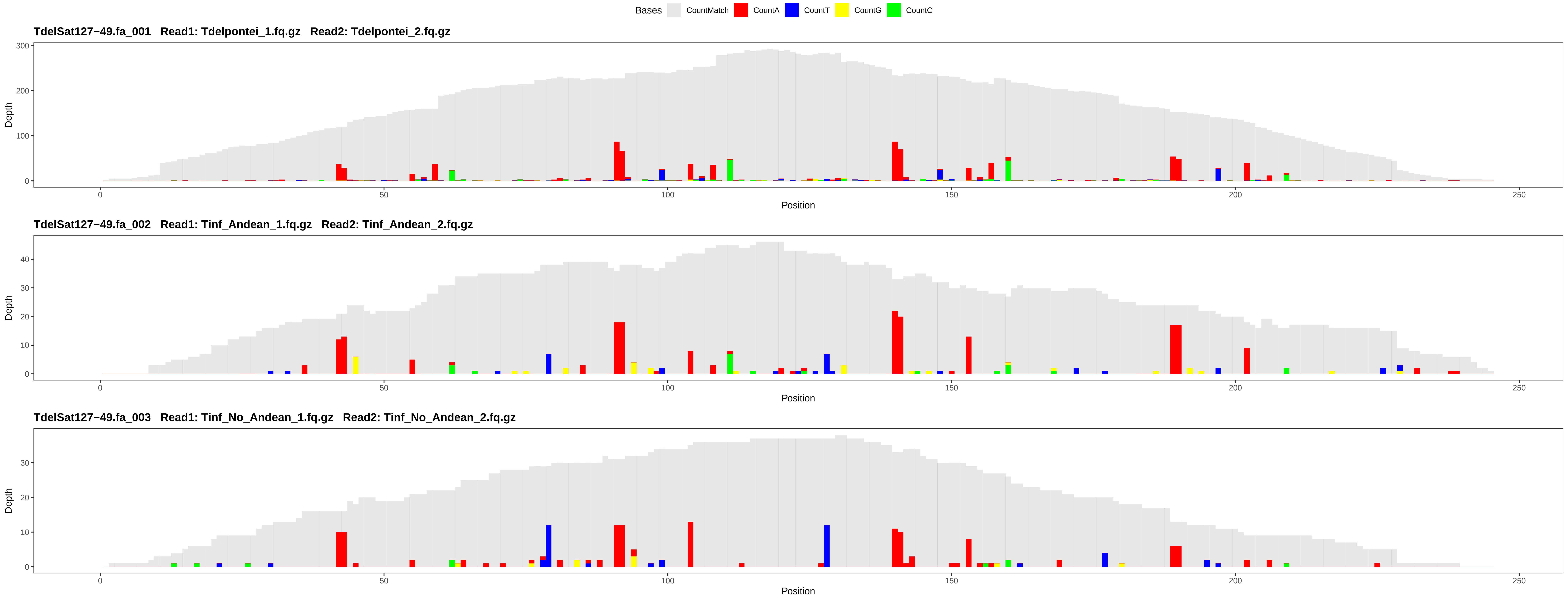

Bases CountMatch CountA CountT CountG CountC
TdelSat127−49.fa_001 Read1: Tdelpontei_1.fq.gz Read2: Tdelpontei_2.fq.gz
Depth
Position
TdelSat127−49.fa_002 Read1: Tinf_Andean_1.fq.gz Read2: Tinf_Andean_2.fq.gz
Depth
Position
TdelSat127−49.fa_003 Read1: Tinf_No_Andean_1.fq.gz Read2: Tinf_No_Andean_2.fq.gz
Depth
Position

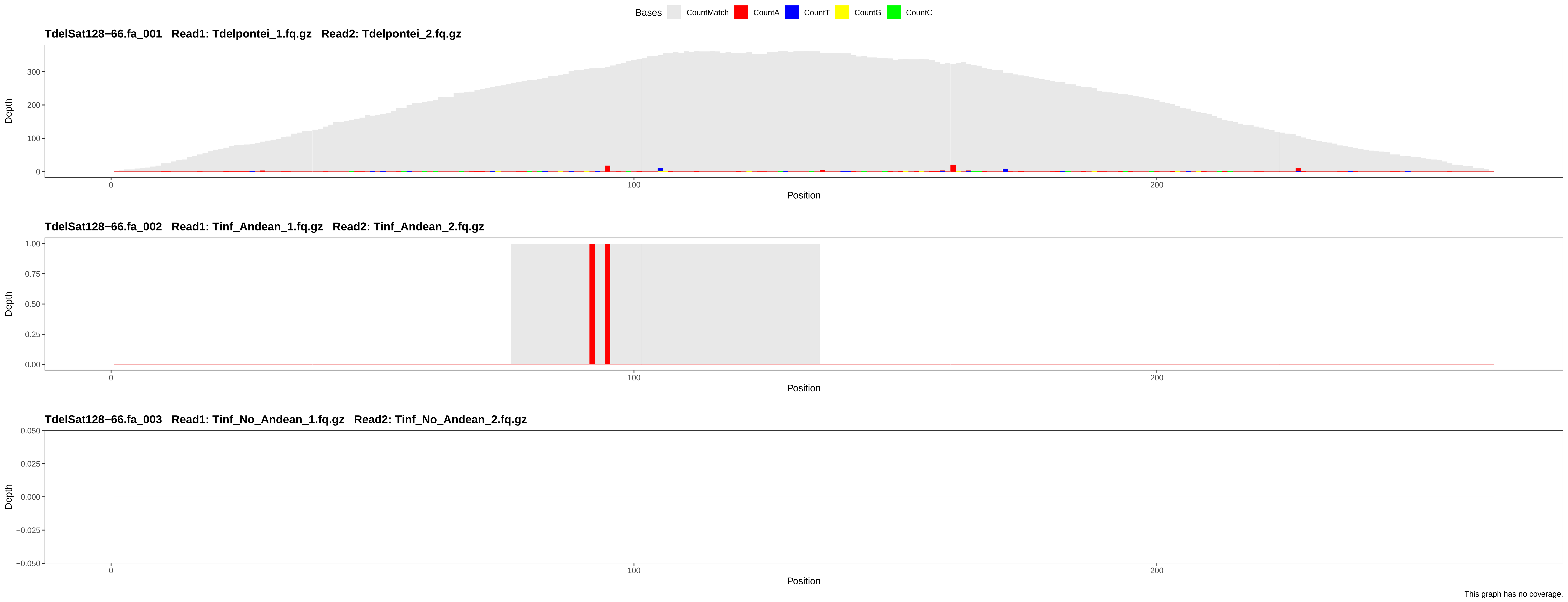
Bases
CountMatch
CountA
CountT
CountG
CountC
TdelSat128−66.fa_001 Read1: Tdelpontei_1.fq.gz Read2: Tdelpontei_2.fq.gz
Depth
Position
TdelSat128−66.fa_002 Read1: Tinf_Andean_1.fq.gz Read2: Tinf_Andean_2.fq.gz
Depth
Position
TdelSat128−66.fa_003 Read1: Tinf_No_Andean_1.fq.gz Read2: Tinf_No_Andean_2.fq.gz
Depth
Position
This graph has no coverage.

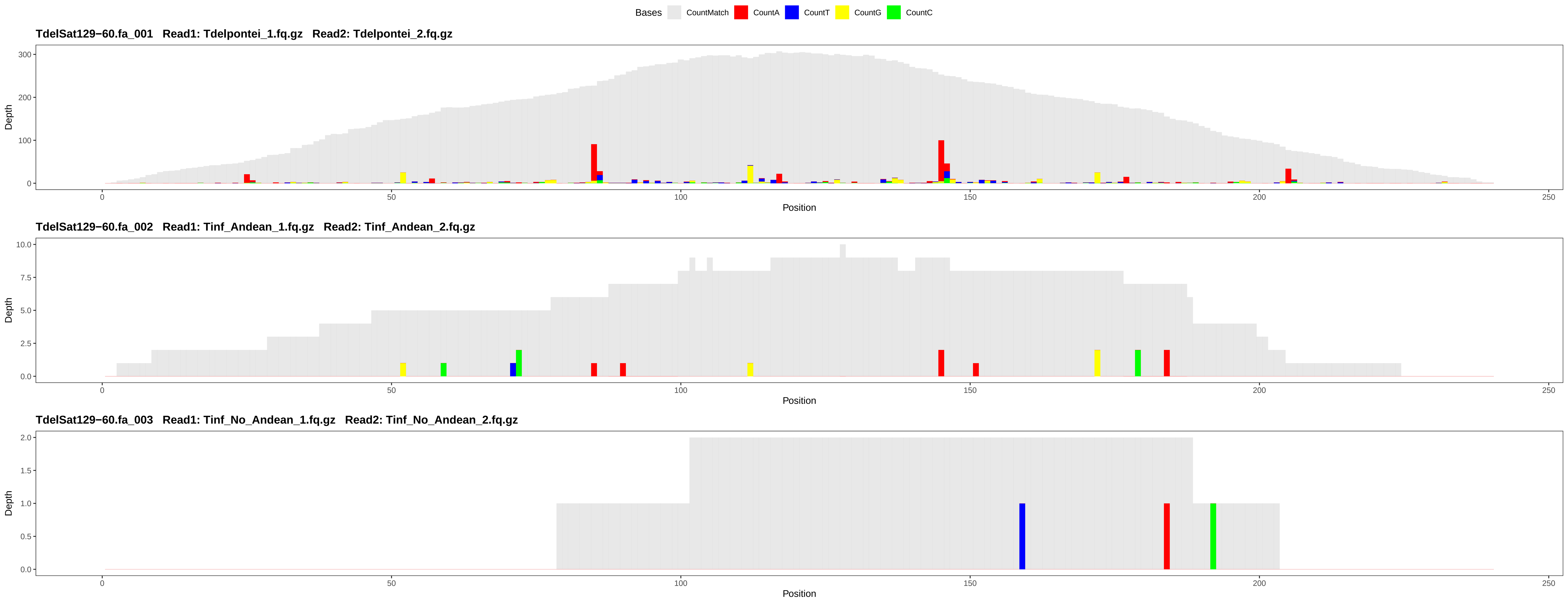
Bases
CountMatch
CountA
CountT
CountG
CountC
TdelSat129−60.fa_001 Read1: Tdelpontei_1.fq.gz Read2: Tdelpontei_2.fq.gz
Depth
Position
TdelSat129−60.fa_002 Read1: Tinf_Andean_1.fq.gz Read2: Tinf_Andean_2.fq.gz
Depth
Position
TdelSat129−60.fa_003 Read1: Tinf_No_Andean_1.fq.gz Read2: Tinf_No_Andean_2.fq.gz
Depth
Position

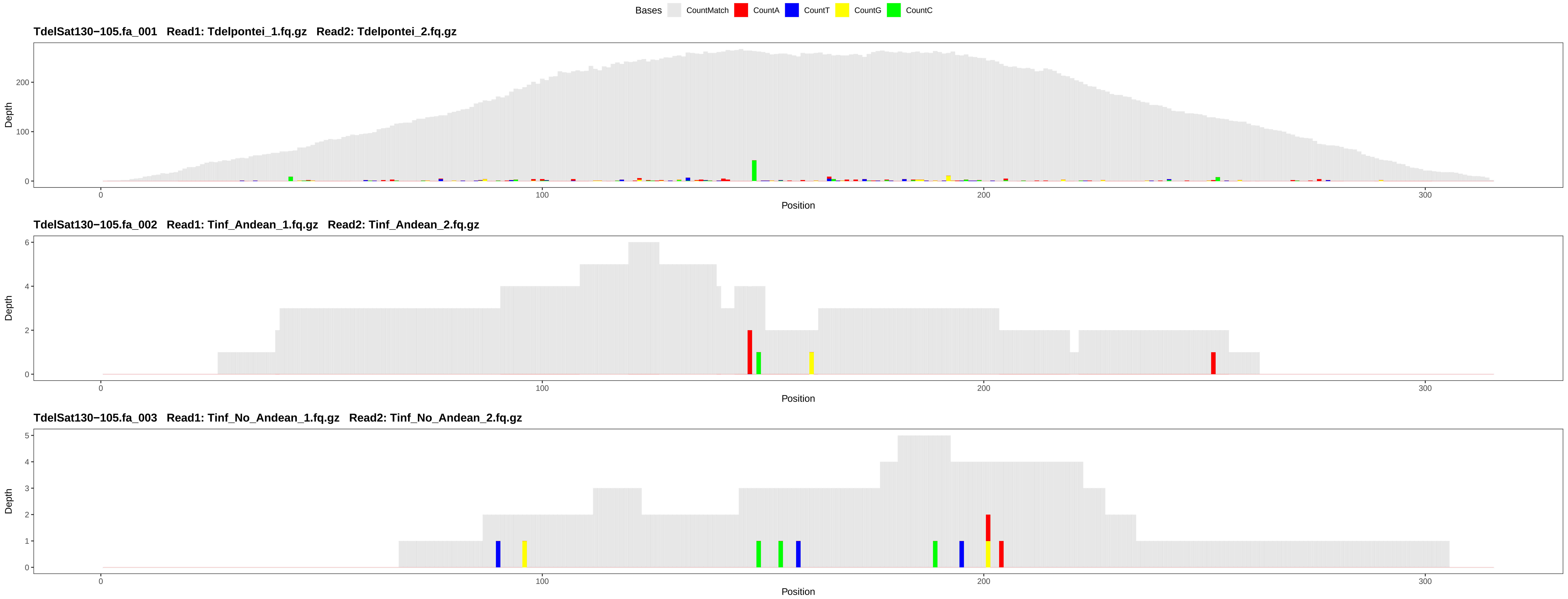
Bases CountMatch CountA CountT CountG CountC
TdelSat130−105.fa_001 Read1: Tdelpontei_1.fq.gz Read2: Tdelpontei_2.fq.gz
Depth
Position
TdelSat130−105.fa_002 Read1: Tinf_Andean_1.fq.gz Read2: Tinf_Andean_2.fq.gz
Depth
Position
TdelSat130−105.fa_003 Read1: Tinf_No_Andean_1.fq.gz Read2: Tinf_No_Andean_2.fq.gz
Depth
Position

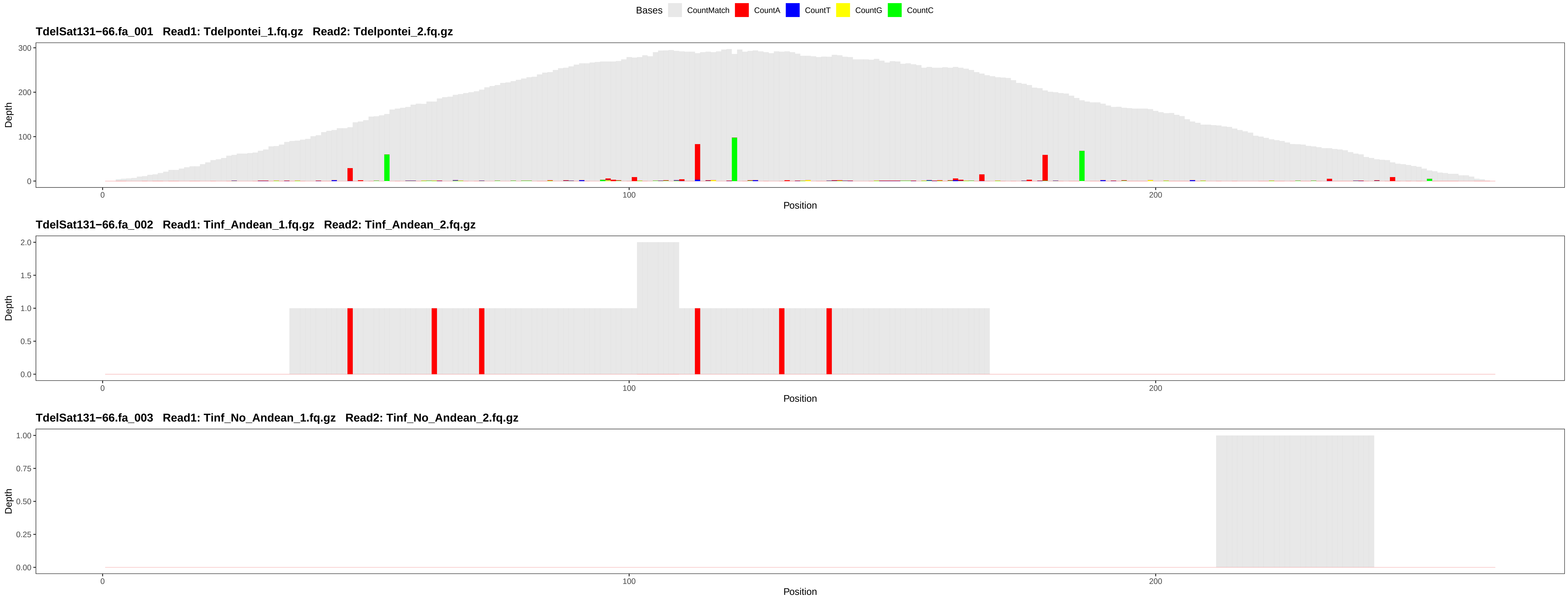

Bases CountMatch CountA CountT CountG CountC
TdelSat131−66.fa_001 Read1: Tdelpontei_1.fq.gz Read2: Tdelpontei_2.fq.gz
Depth
Position
TdelSat131−66.fa_002 Read1: Tinf_Andean_1.fq.gz Read2: Tinf_Andean_2.fq.gz
Depth
Position
TdelSat131−66.fa_003 Read1: Tinf_No_Andean_1.fq.gz Read2: Tinf_No_Andean_2.fq.gz
Depth
Position

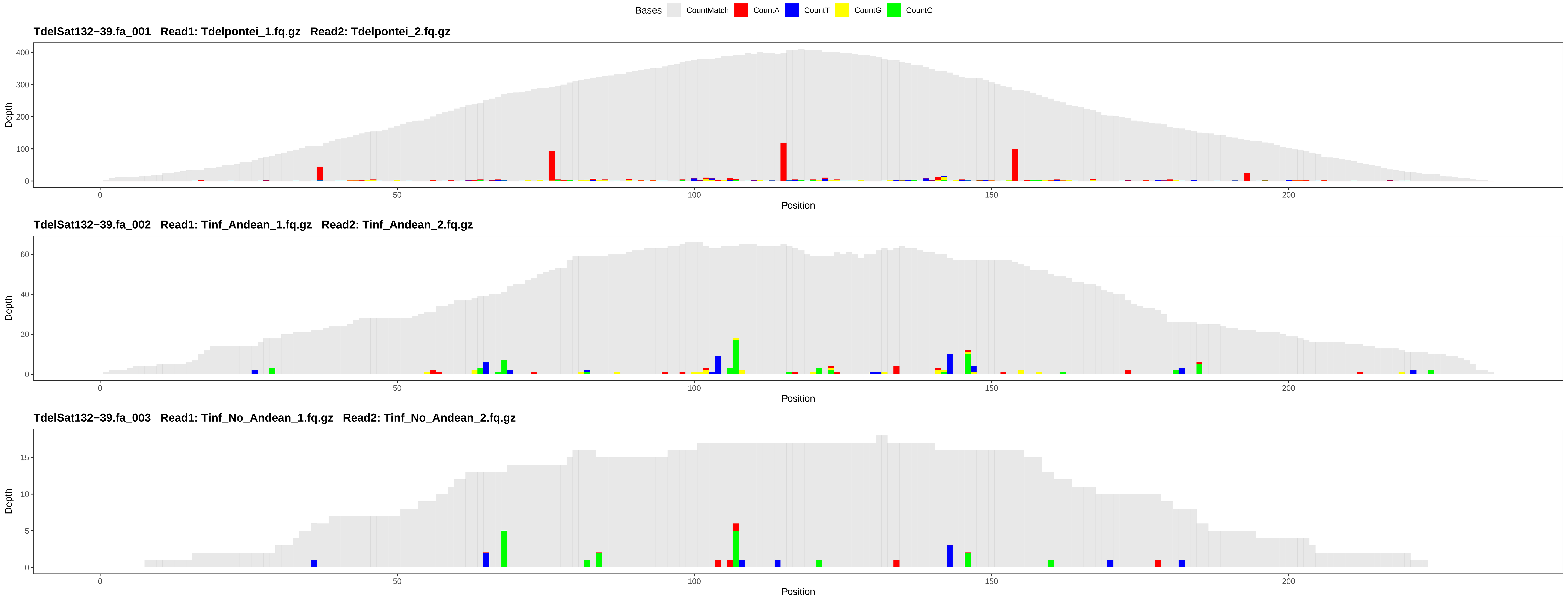

Bases
CountMatch
CountA
CountT
CountG
CountC
TdelSat132−39.fa_001 Read1: Tdelpontei_1.fq.gz Read2: Tdelpontei_2.fq.gz
Depth
Position
TdelSat132−39.fa_002 Read1: Tinf_Andean_1.fq.gz Read2: Tinf_Andean_2.fq.gz
Depth
Position
TdelSat132−39.fa_003 Read1: Tinf_No_Andean_1.fq.gz Read2: Tinf_No_Andean_2.fq.gz
Depth
Position

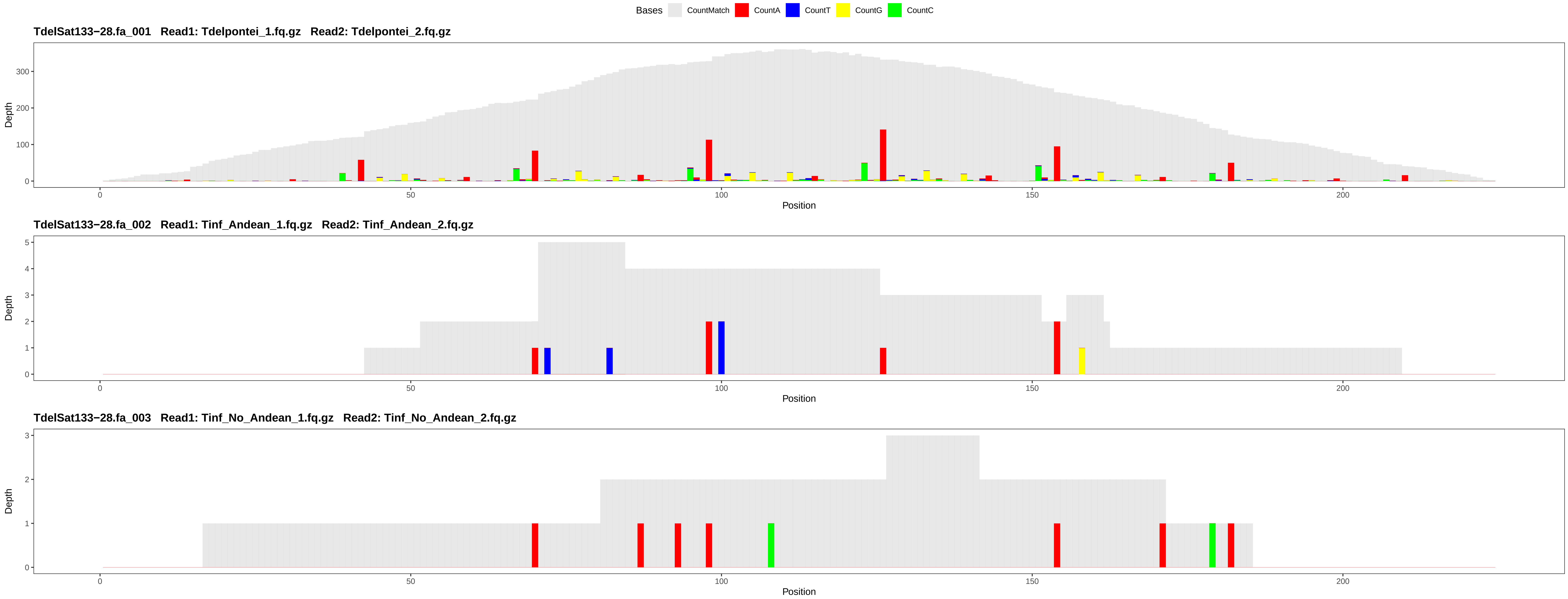
Bases
CountMatch
CountA
CountT
CountG
CountC
TdelSat133−28.fa_001 Read1: Tdelpontei_1.fq.gz Read2: Tdelpontei_2.fq.gz
Depth
300
200
100
0
0
50
100
150
200
Position
TdelSat133−28.fa_002 Read1: Tinf_Andean_1.fq.gz Read2: Tinf_Andean_2.fq.gz
Depth
5
4
3
2
1
0
0
50
100
150
200
Position
TdelSat133−28.fa_003 Read1: Tinf_No_Andean_1.fq.gz Read2: Tinf_No_Andean_2.fq.gz
Depth
3
2
1
0
0
50
100
150
200
Position

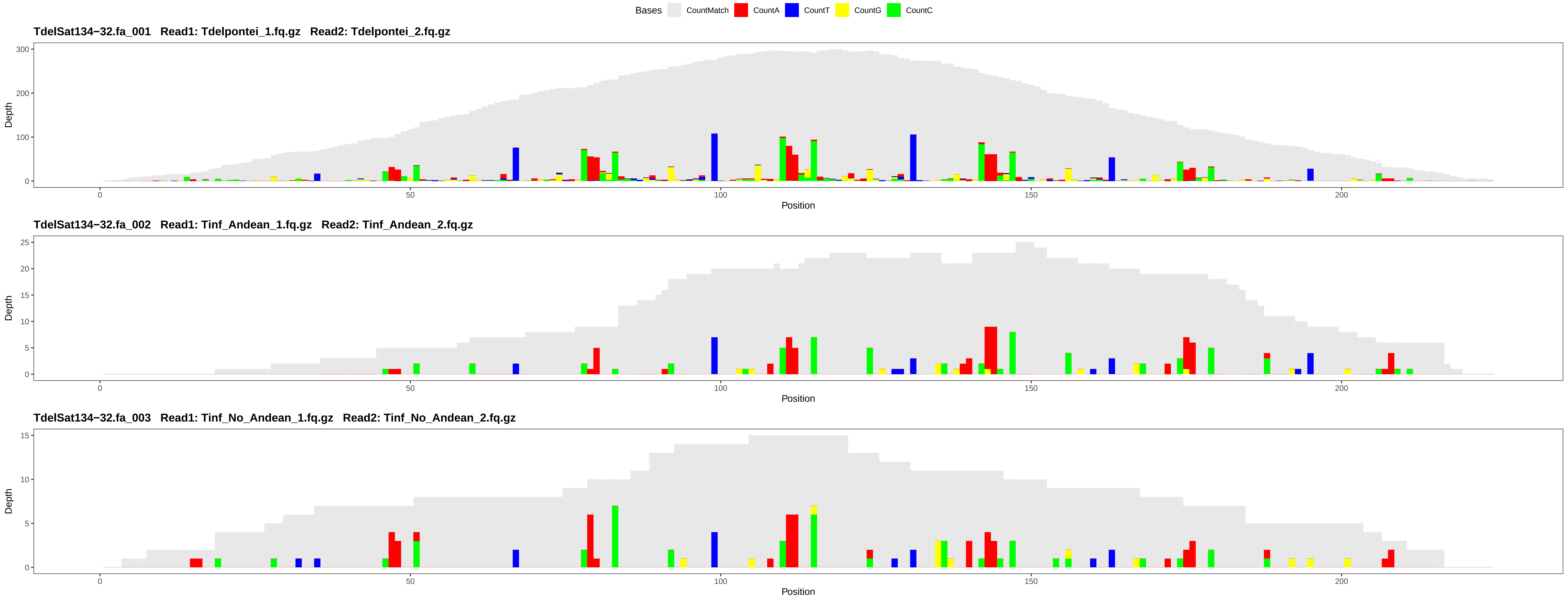

Bases
CountMatch
CountA
CountT
CountG
CountC
TdelSat134−32.fa_001 Read1: Tdelpontei_1.fq.gz Read2: Tdelpontei_2.fq.gz
Depth
0
100
200
300
0
50
100
150
200
Position
TdelSat134−32.fa_002 Read1: Tinf_Andean_1.fq.gz Read2: Tinf_Andean_2.fq.gz
Depth
0
5
10
15
20
25
0
50
100
150
200
Position
TdelSat134−32.fa_003 Read1: Tinf_No_Andean_1.fq.gz Read2: Tinf_No_Andean_2.fq.gz
Depth
0
5
10
15
0
50
100
150
200
Position

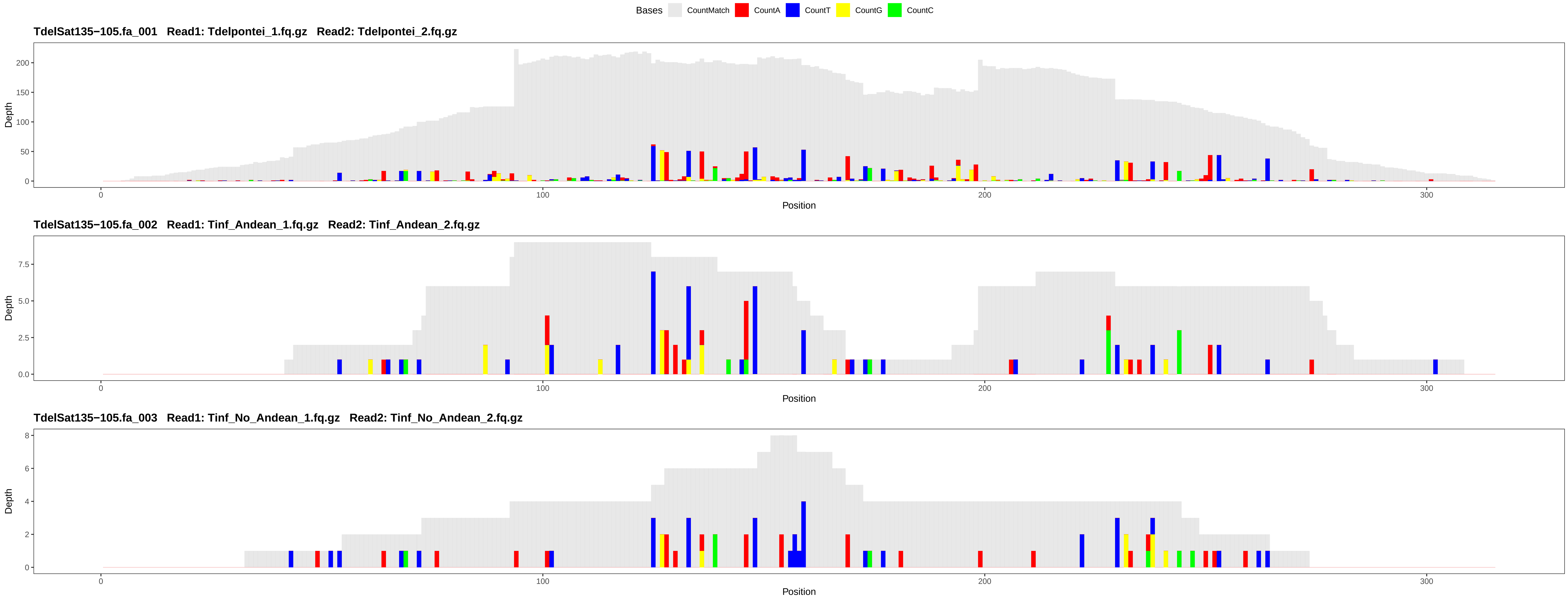
Bases
CountMatch
CountA
CountT
CountG
CountC
TdelSat135−105.fa_001 Read1: Tdelpontei_1.fq.gz Read2: Tdelpontei_2.fq.gz
Depth
Position
TdelSat135−105.fa_002 Read1: Tinf_Andean_1.fq.gz Read2: Tinf_Andean_2.fq.gz
Depth
Position
TdelSat135−105.fa_003 Read1: Tinf_No_Andean_1.fq.gz Read2: Tinf_No_Andean_2.fq.gz
Depth
Position

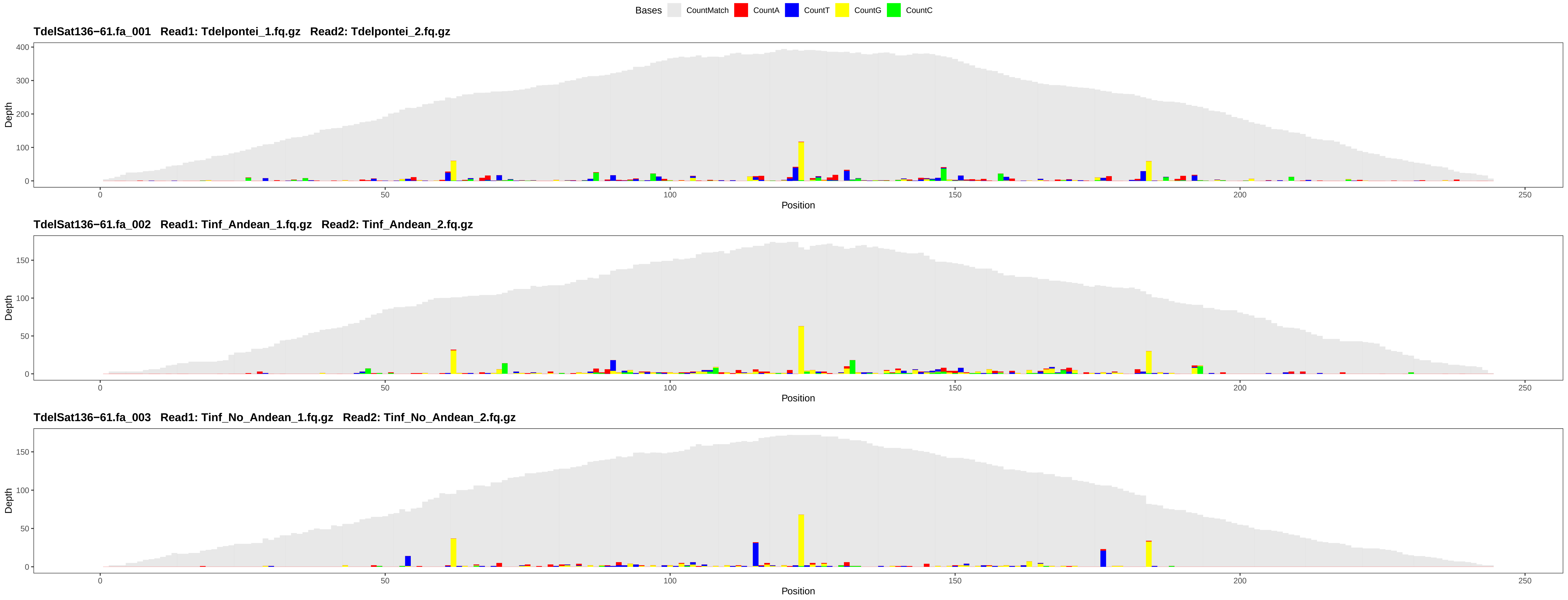
Bases
CountMatch
CountA
CountT
CountG
CountC
TdelSat136−61.fa_001 Read1: Tdelpontei_1.fq.gz Read2: Tdelpontei_2.fq.gz
Depth
Position
TdelSat136−61.fa_002 Read1: Tinf_Andean_1.fq.gz Read2: Tinf_Andean_2.fq.gz
Depth
Position
TdelSat136−61.fa_003 Read1: Tinf_No_Andean_1.fq.gz Read2: Tinf_No_Andean_2.fq.gz
Depth
Position

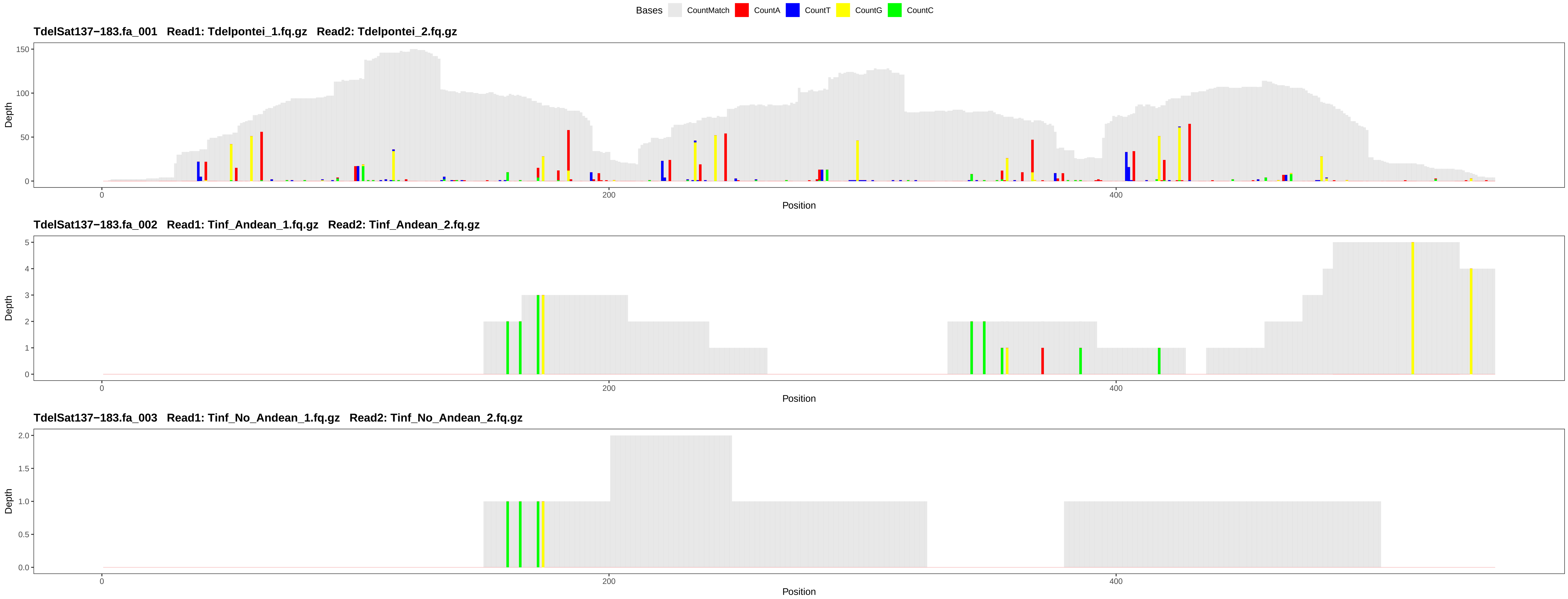

Bases
CountMatch
CountA
CountT
CountG
CountC
TdelSat137−183.fa_001 Read1: Tdelpontei_1.fq.gz Read2: Tdelpontei_2.fq.gz
Depth
Position
TdelSat137−183.fa_002 Read1: Tinf_Andean_1.fq.gz Read2: Tinf_Andean_2.fq.gz
Depth
Position
TdelSat137−183.fa_003 Read1: Tinf_No_Andean_1.fq.gz Read2: Tinf_No_Andean_2.fq.gz
Depth
Position

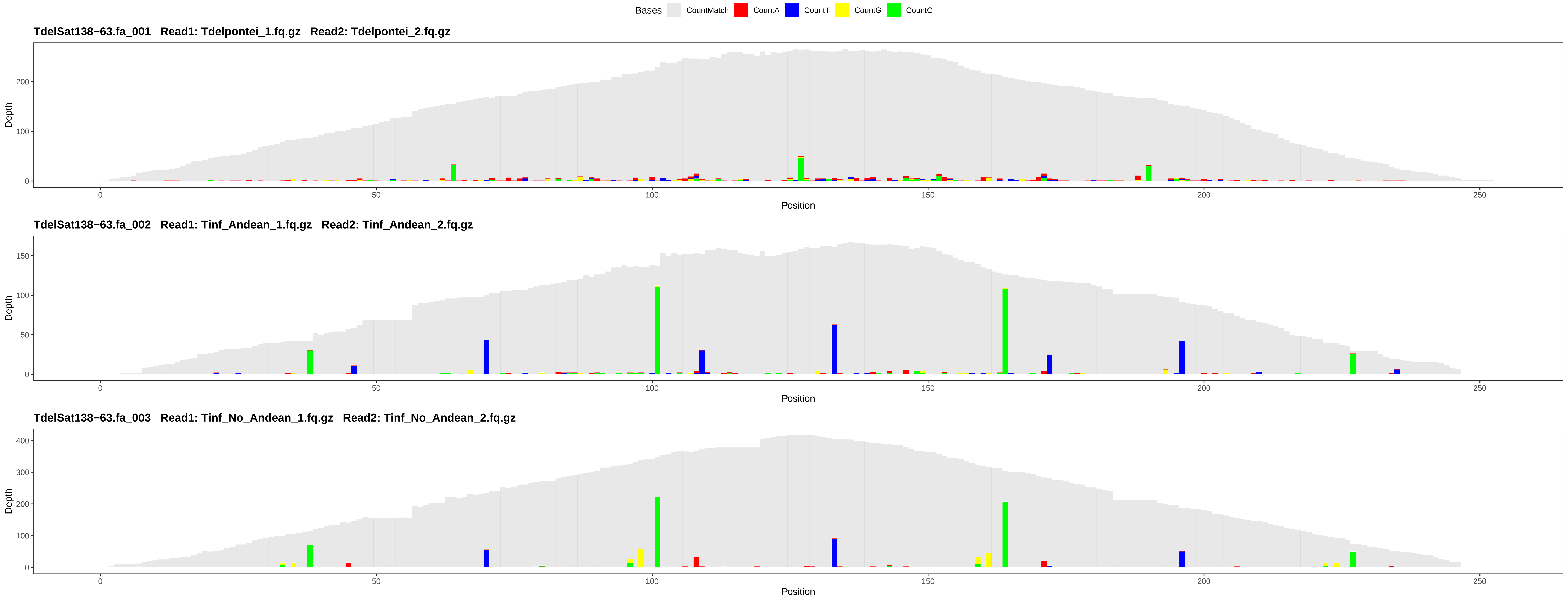

Bases CountMatch CountA CountT CountG CountC
TdelSat138−63.fa_001 Read1: Tdelpontei_1.fq.gz Read2: Tdelpontei_2.fq.gz
Depth
0
100
200
Position
0
50
100
150
200
250
TdelSat138−63.fa_002 Read1: Tinf_Andean_1.fq.gz Read2: Tinf_Andean_2.fq.gz
Depth
0
50
100
150
Position
0
50
100
150
200
250
TdelSat138−63.fa_003 Read1: Tinf_No_Andean_1.fq.gz Read2: Tinf_No_Andean_2.fq.gz
Depth
0
100
200
300
400
Position
0
50
100
150
200
250

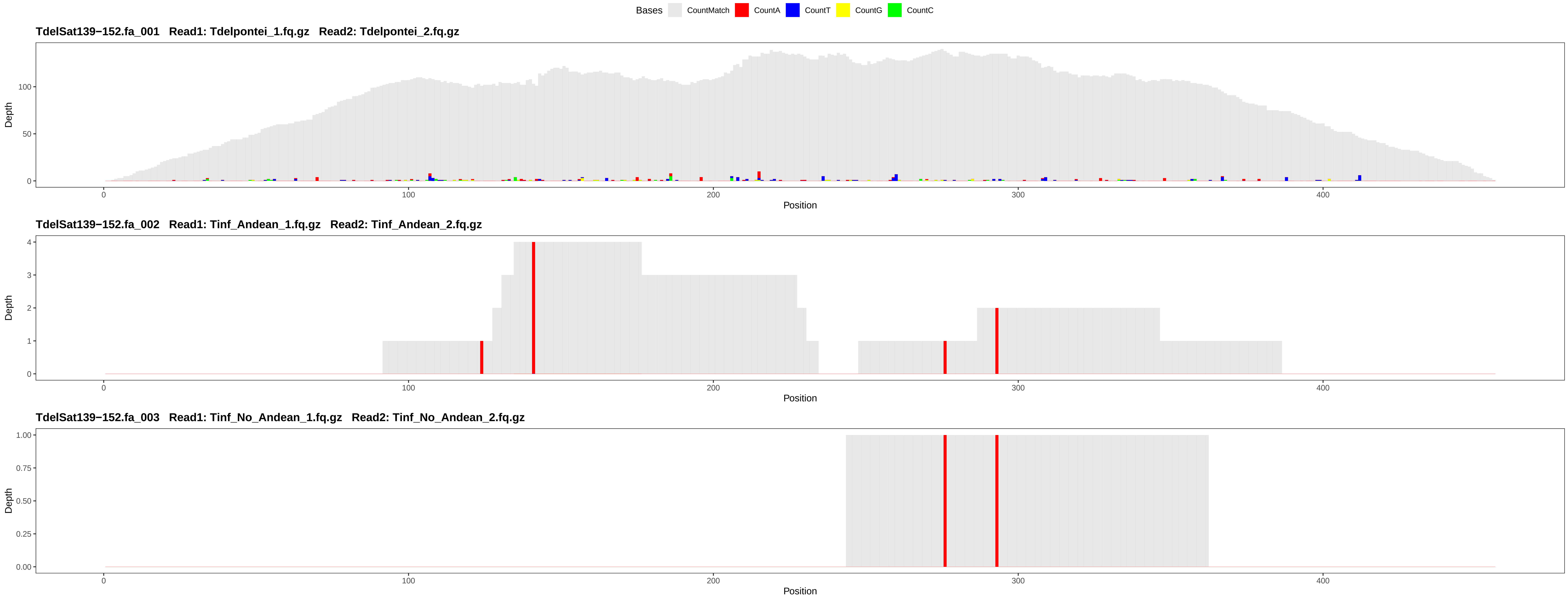
Bases CountMatch CountA CountT CountG CountC
TdelSat139−152.fa_001 Read1: Tdelpontei_1.fq.gz Read2: Tdelpontei_2.fq.gz
Depth
0
50
100
Position
0
100
200
300
400
TdelSat139−152.fa_002 Read1: Tinf_Andean_1.fq.gz Read2: Tinf_Andean_2.fq.gz
Depth
0
1
2
3
4
Position
0
100
200
300
400
TdelSat139−152.fa_003 Read1: Tinf_No_Andean_1.fq.gz Read2: Tinf_No_Andean_2.fq.gz
Depth
0.00
0.25
0.50
0.75
1.00
Position
0
100
200
300
400

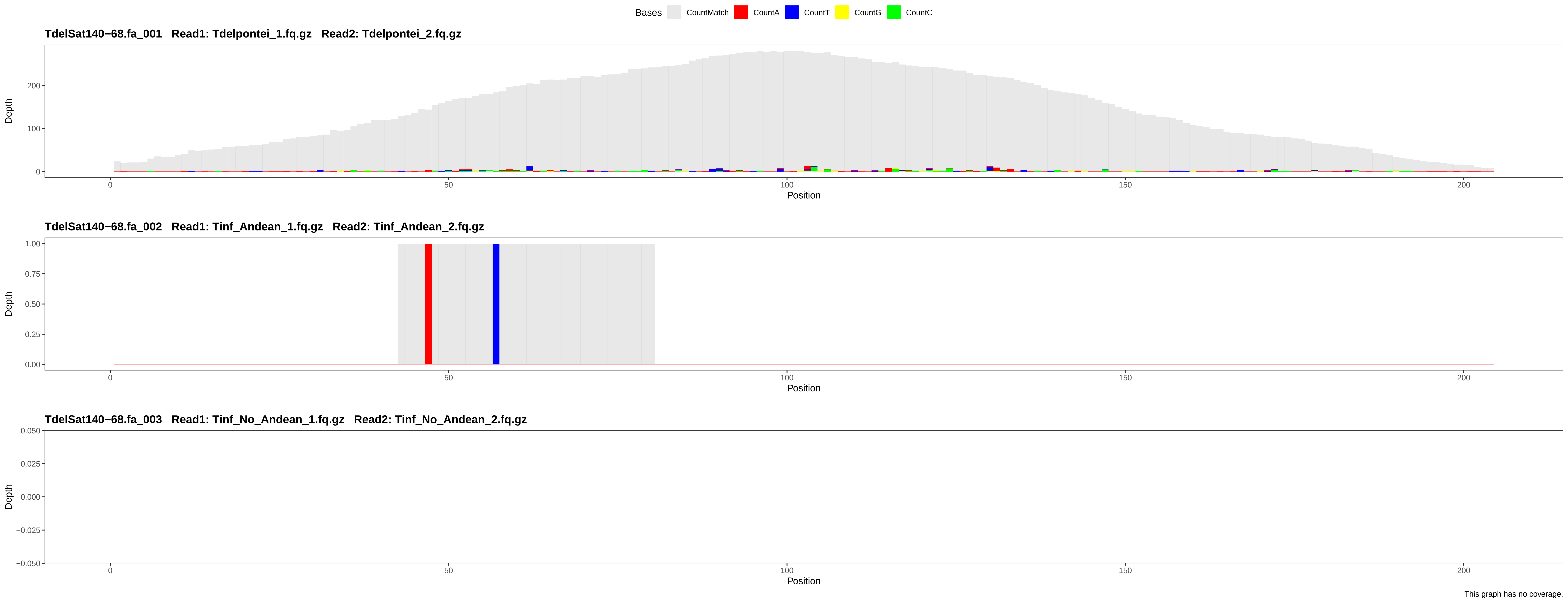

Bases CountMatch CountA CountT CountG CountC
TdelSat140−68.fa_001 Read1: Tdelpontei_1.fq.gz Read2: Tdelpontei_2.fq.gz
Depth
0
100
200
0 50 100 150 200
Position
TdelSat140−68.fa_002 Read1: Tinf_Andean_1.fq.gz Read2: Tinf_Andean_2.fq.gz
Depth
0.00
0.25
0.50
0.75
1.00
0 50 100 150 200
Position
TdelSat140−68.fa_003 Read1: Tinf_No_Andean_1.fq.gz Read2: Tinf_No_Andean_2.fq.gz
Depth
−0.050
−0.025
0.000
0.025
0.050
0 50 100 150 200
Position
This graph has no coverage.

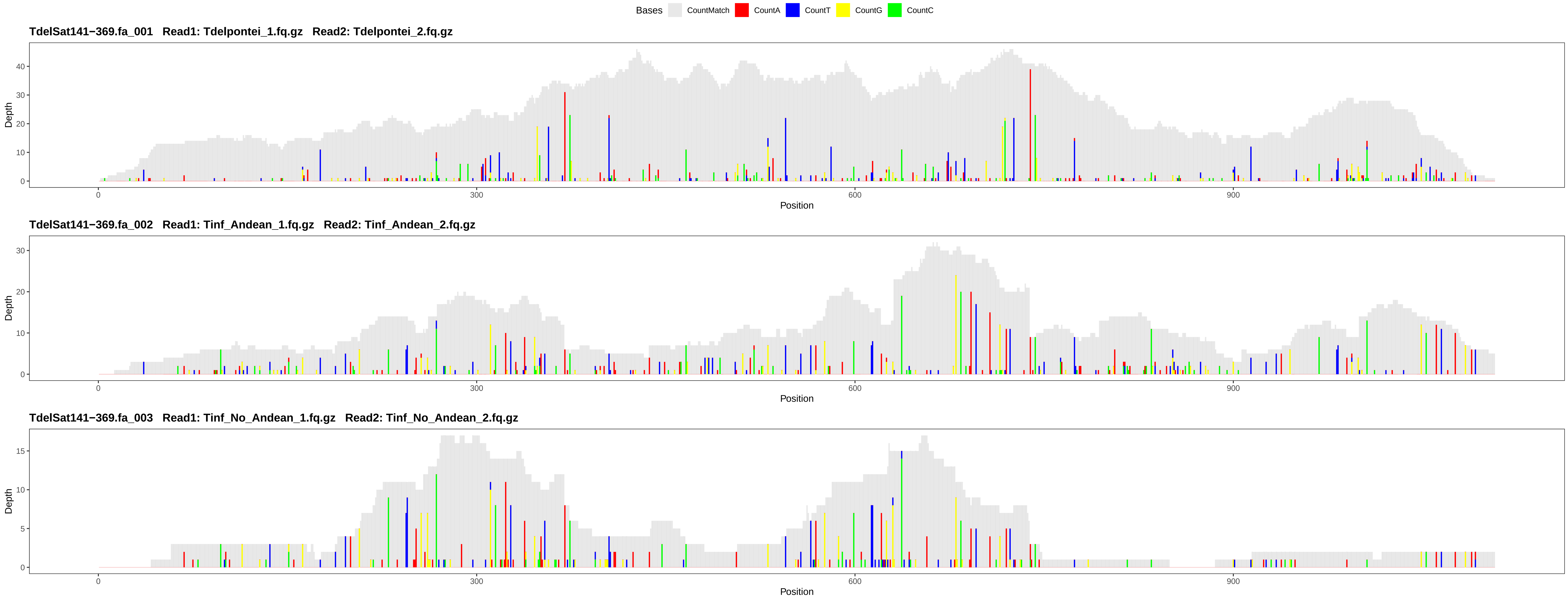

Bases CountMatch CountA CountT CountG CountC
TdelSat141−369.fa_001 Read1: Tdelpontei_1.fq.gz Read2: Tdelpontei_2.fq.gz
Depth
0
10
20
30
40
0
300
600
900
Position
TdelSat141−369.fa_002 Read1: Tinf_Andean_1.fq.gz Read2: Tinf_Andean_2.fq.gz
Depth
0
10
20
30
0
300
600
900
Position
TdelSat141−369.fa_003 Read1: Tinf_No_Andean_1.fq.gz Read2: Tinf_No_Andean_2.fq.gz
Depth
0
5
10
15
0
300
600
900
Position

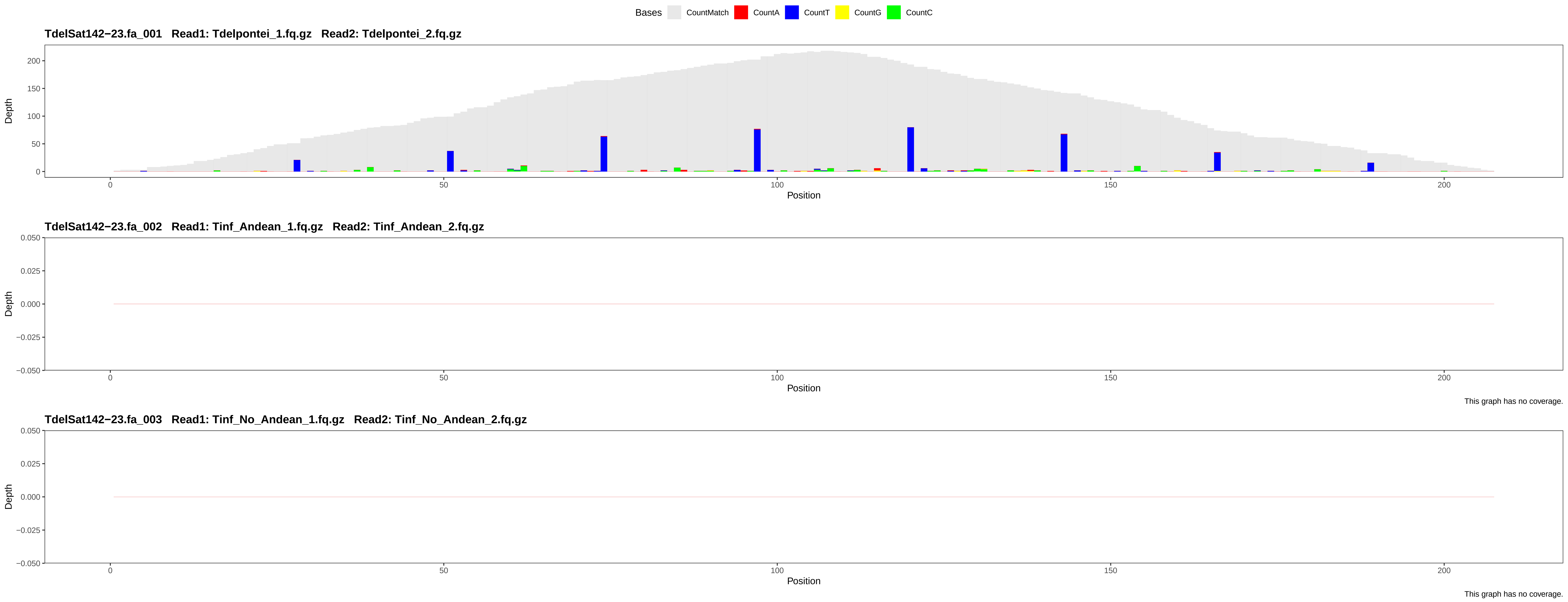
Bases CountMatch CountA CountT CountG CountC
TdelSat142−23.fa_001 Read1: Tdelpontei_1.fq.gz Read2: Tdelpontei_2.fq.gz
Depth
Position
TdelSat142−23.fa_002 Read1: Tinf_Andean_1.fq.gz Read2: Tinf_Andean_2.fq.gz
Depth
Position
This graph has no coverage.
TdelSat142−23.fa_003 Read1: Tinf_No_Andean_1.fq.gz Read2: Tinf_No_Andean_2.fq.gz
Depth
Position
This graph has no coverage.

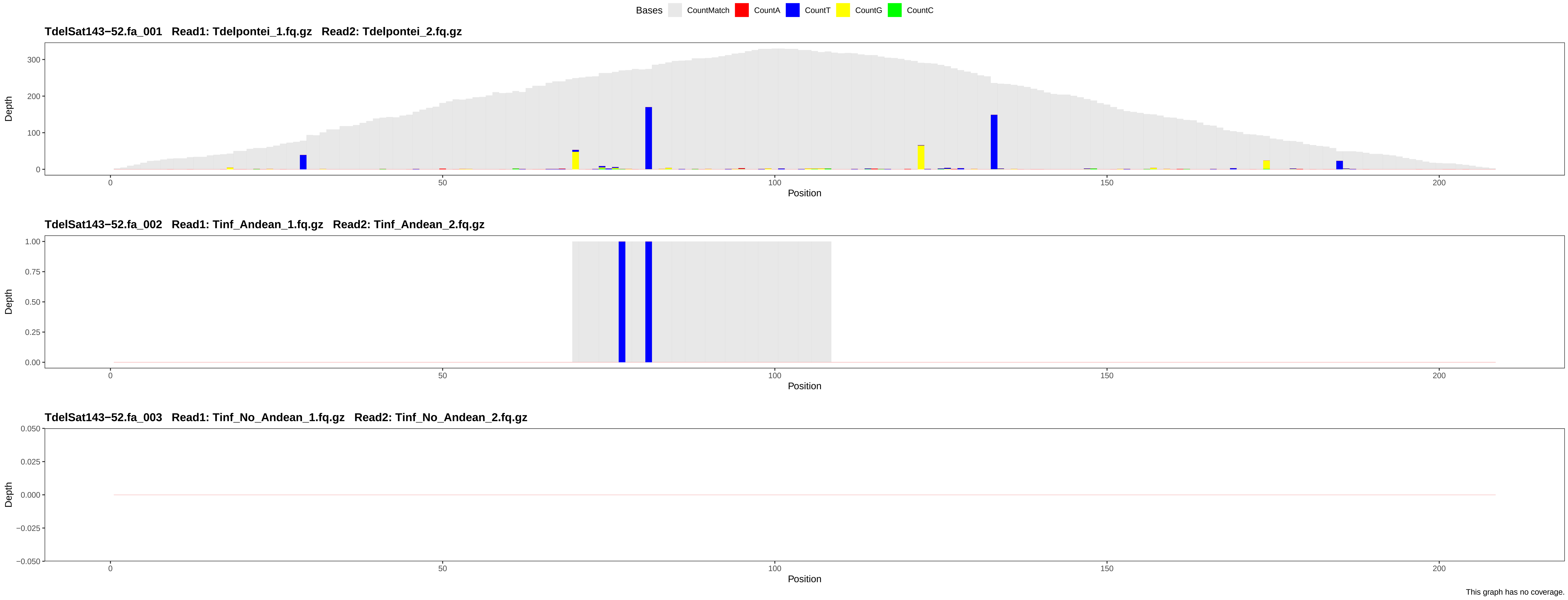
Bases
CountMatch
CountA
CountT
CountG
CountC
TdelSat143−52.fa_001 Read1: Tdelpontei_1.fq.gz Read2: Tdelpontei_2.fq.gz
Depth
Position
TdelSat143−52.fa_002 Read1: Tinf_Andean_1.fq.gz Read2: Tinf_Andean_2.fq.gz
Depth
Position
TdelSat143−52.fa_003 Read1: Tinf_No_Andean_1.fq.gz Read2: Tinf_No_Andean_2.fq.gz
Depth
Position
This graph has no coverage.

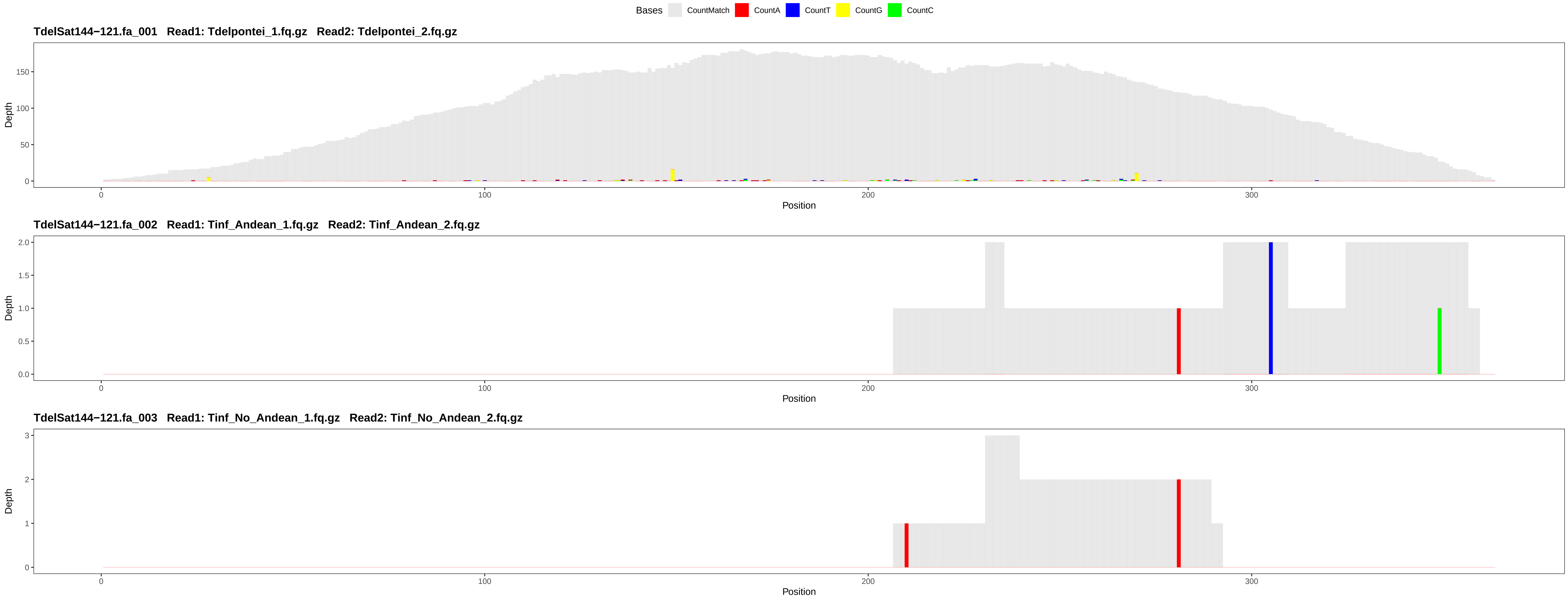

Bases
CountMatch
CountA
CountT
CountG
CountC
TdelSat144−121.fa_001 Read1: Tdelpontei_1.fq.gz Read2: Tdelpontei_2.fq.gz
Depth
Position
TdelSat144−121.fa_002 Read1: Tinf_Andean_1.fq.gz Read2: Tinf_Andean_2.fq.gz
Depth
Position
TdelSat144−121.fa_003 Read1: Tinf_No_Andean_1.fq.gz Read2: Tinf_No_Andean_2.fq.gz
Depth
Position

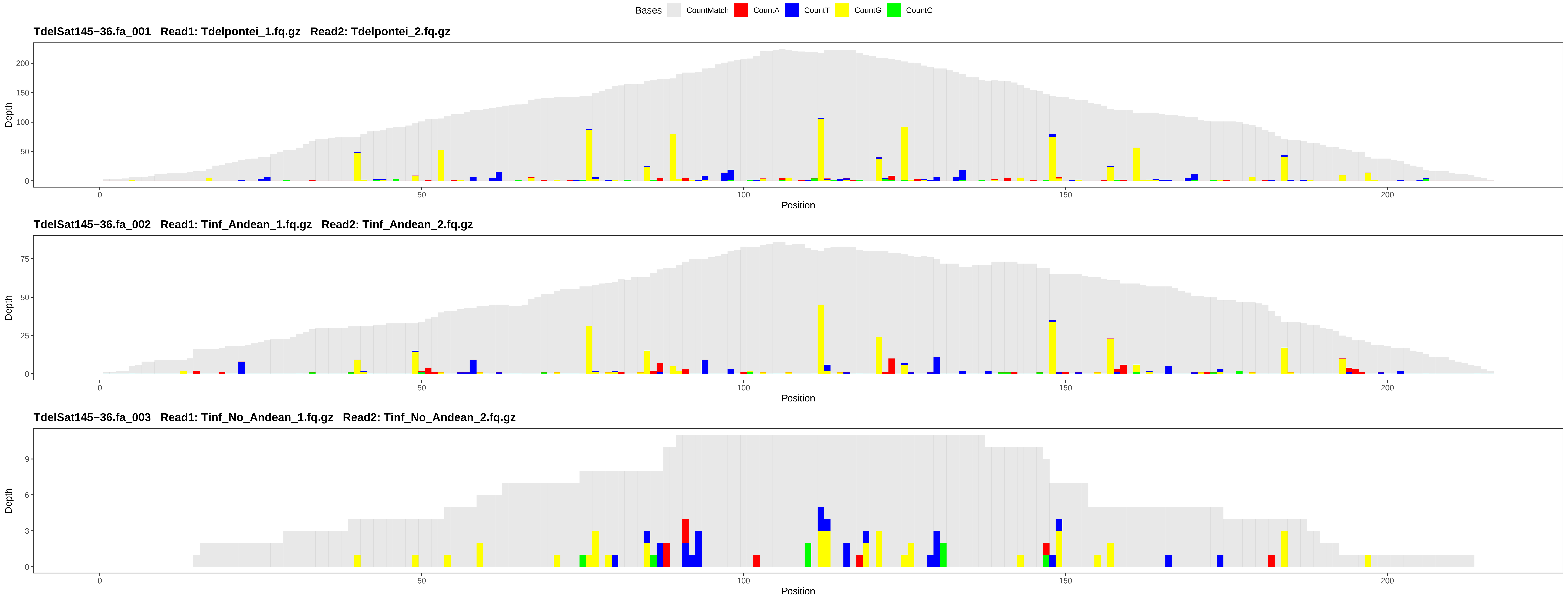

Bases CountMatch CountA CountT CountG CountC
TdelSat145−36.fa_001 Read1: Tdelpontei_1.fq.gz Read2: Tdelpontei_2.fq.gz
Depth
Position
TdelSat145−36.fa_002 Read1: Tinf_Andean_1.fq.gz Read2: Tinf_Andean_2.fq.gz
Depth
Position
TdelSat145−36.fa_003 Read1: Tinf_No_Andean_1.fq.gz Read2: Tinf_No_Andean_2.fq.gz
Depth
Position

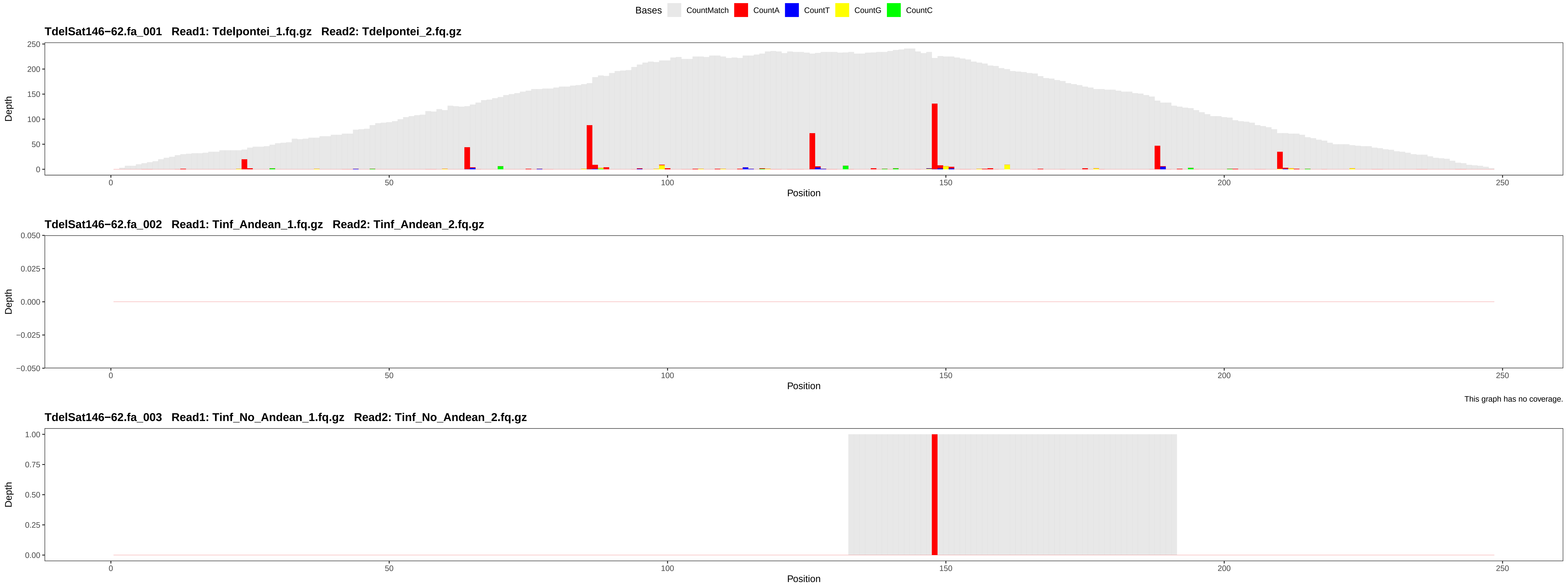

Bases CountMatch CountA CountT CountG CountC
TdelSat146−62.fa_001 Read1: Tdelpontei_1.fq.gz Read2: Tdelpontei_2.fq.gz
Depth
Position
TdelSat146−62.fa_002 Read1: Tinf_Andean_1.fq.gz Read2: Tinf_Andean_2.fq.gz
Depth
Position
This graph has no coverage.
TdelSat146−62.fa_003 Read1: Tinf_No_Andean_1.fq.gz Read2: Tinf_No_Andean_2.fq.gz
Depth
Position

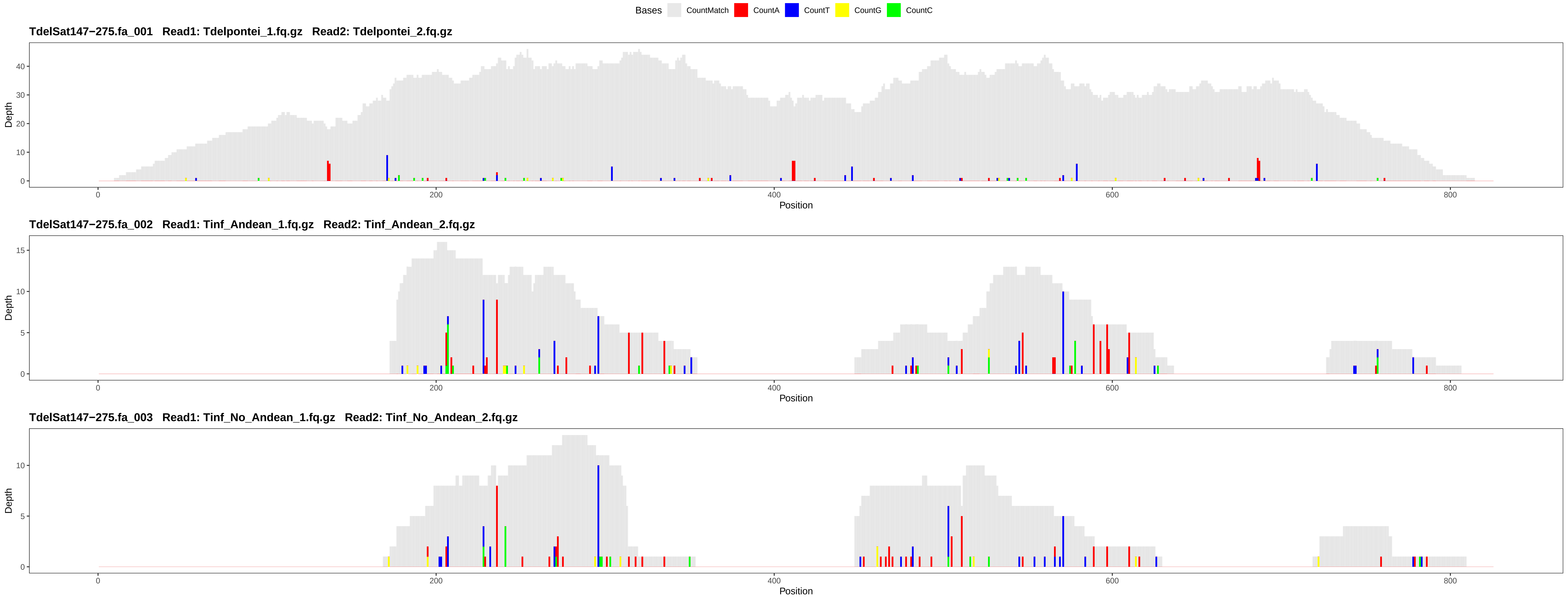

Bases CountMatch CountA CountT CountG CountC
TdelSat147−275.fa_001 Read1: Tdelpontei_1.fq.gz Read2: Tdelpontei_2.fq.gz
Depth
Position
TdelSat147−275.fa_002 Read1: Tinf_Andean_1.fq.gz Read2: Tinf_Andean_2.fq.gz
Depth
Position
TdelSat147−275.fa_003 Read1: Tinf_No_Andean_1.fq.gz Read2: Tinf_No_Andean_2.fq.gz
Depth
Position

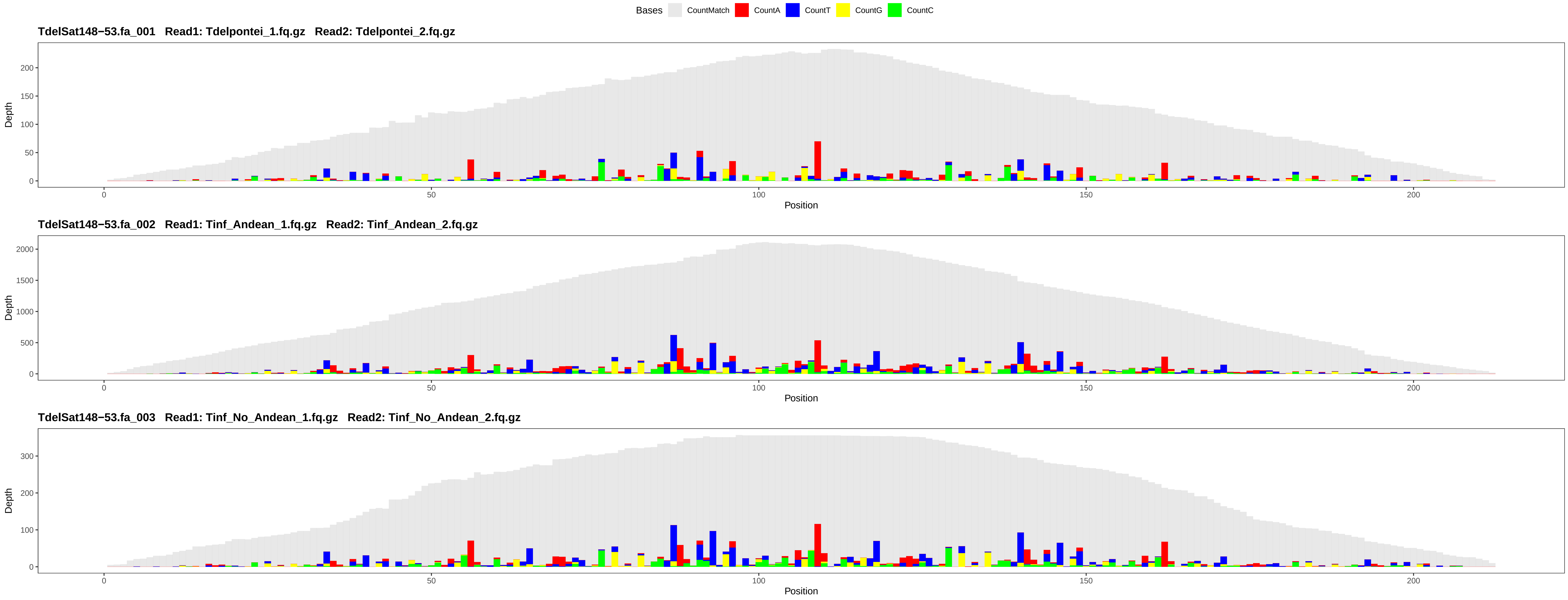

Bases
CountMatch
CountA
CountT
CountG
CountC
TdelSat148−53.fa_001 Read1: Tdelpontei_1.fq.gz Read2: Tdelpontei_2.fq.gz
TdelSat148−53.fa_002 Read1: Tinf_Andean_1.fq.gz Read2: Tinf_Andean_2.fq.gz
TdelSat148−53.fa_003 Read1: Tinf_No_Andean_1.fq.gz Read2: Tinf_No_Andean_2.fq.gz
Depth
Position

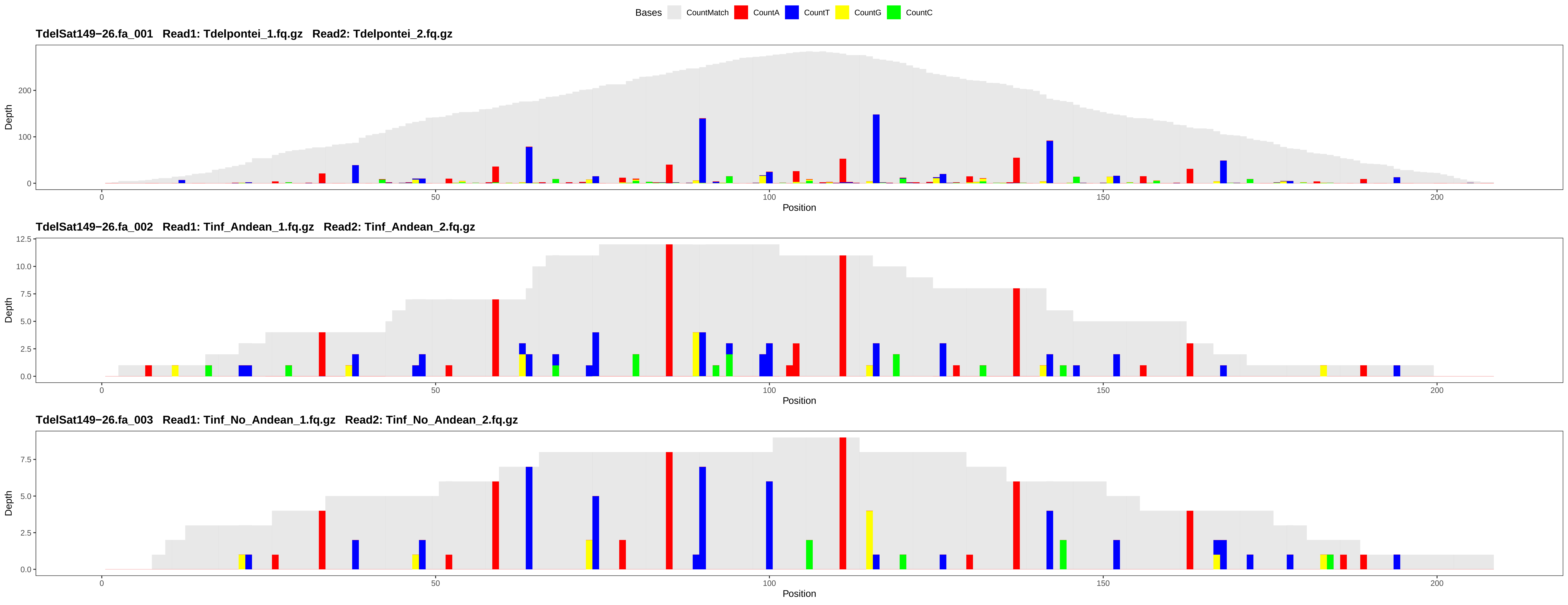
Bases CountMatch CountA CountT CountG CountC
TdelSat149−26.fa_001 Read1: Tdelpontei_1.fq.gz Read2: Tdelpontei_2.fq.gz
Depth
0
100
200
Position
0
50
100
150
200
TdelSat149−26.fa_002 Read1: Tinf_Andean_1.fq.gz Read2: Tinf_Andean_2.fq.gz
Depth
0.0
2.5
5.0
7.5
10.0
12.5
Position
0
50
100
150
200
TdelSat149−26.fa_003 Read1: Tinf_No_Andean_1.fq.gz Read2: Tinf_No_Andean_2.fq.gz
Depth
0.0
2.5
5.0
7.5
Position
0
50
100
150
200

Bases: CountMatch, CountA, CountT, CountG, CountC

TdelSat150−56.fa_001   Read1: Tdelpontei_1.fq.gz   Read2: Tdelpontei_2.fq.gz

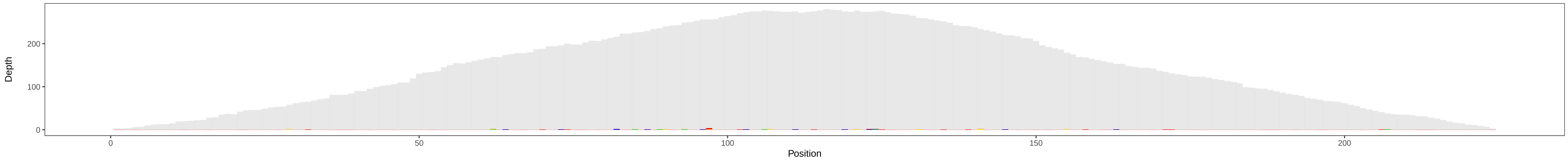

TdelSat150−56.fa_002   Read1: Tinf_Andean_1.fq.gz   Read2: Tinf_Andean_2.fq.gz

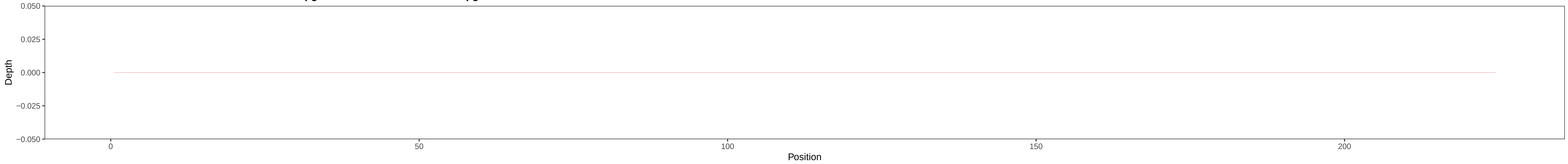

This graph has no coverage.

TdelSat150−56.fa_003   Read1: Tinf_No_Andean_1.fq.gz   Read2: Tinf_No_Andean_2.fq.gz

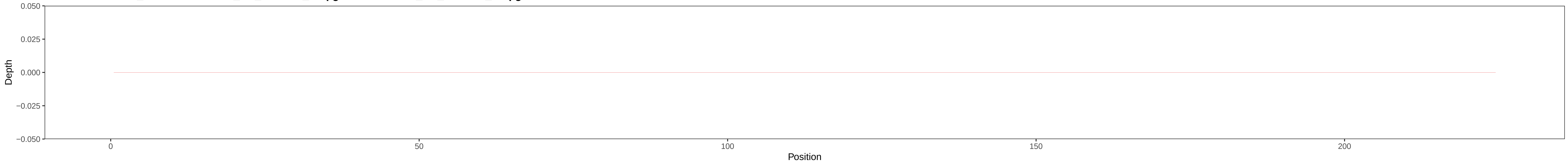

This graph has no coverage.

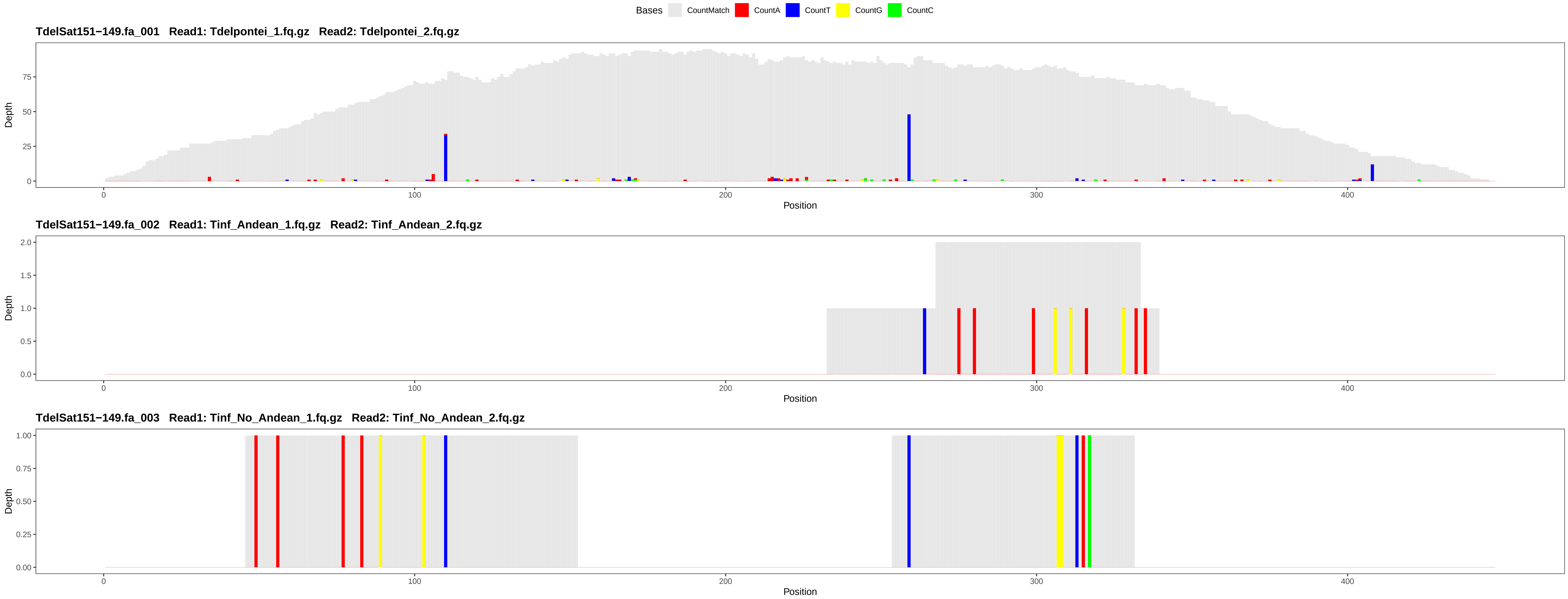
Bases
CountMatch
CountA
CountT
CountG
CountC
TdelSat151−149.fa_001 Read1: Tdelpontei_1.fq.gz Read2: Tdelpontei_2.fq.gz
Depth
Position
TdelSat151−149.fa_002 Read1: Tinf_Andean_1.fq.gz Read2: Tinf_Andean_2.fq.gz
Depth
Position
TdelSat151−149.fa_003 Read1: Tinf_No_Andean_1.fq.gz Read2: Tinf_No_Andean_2.fq.gz
Depth
Position

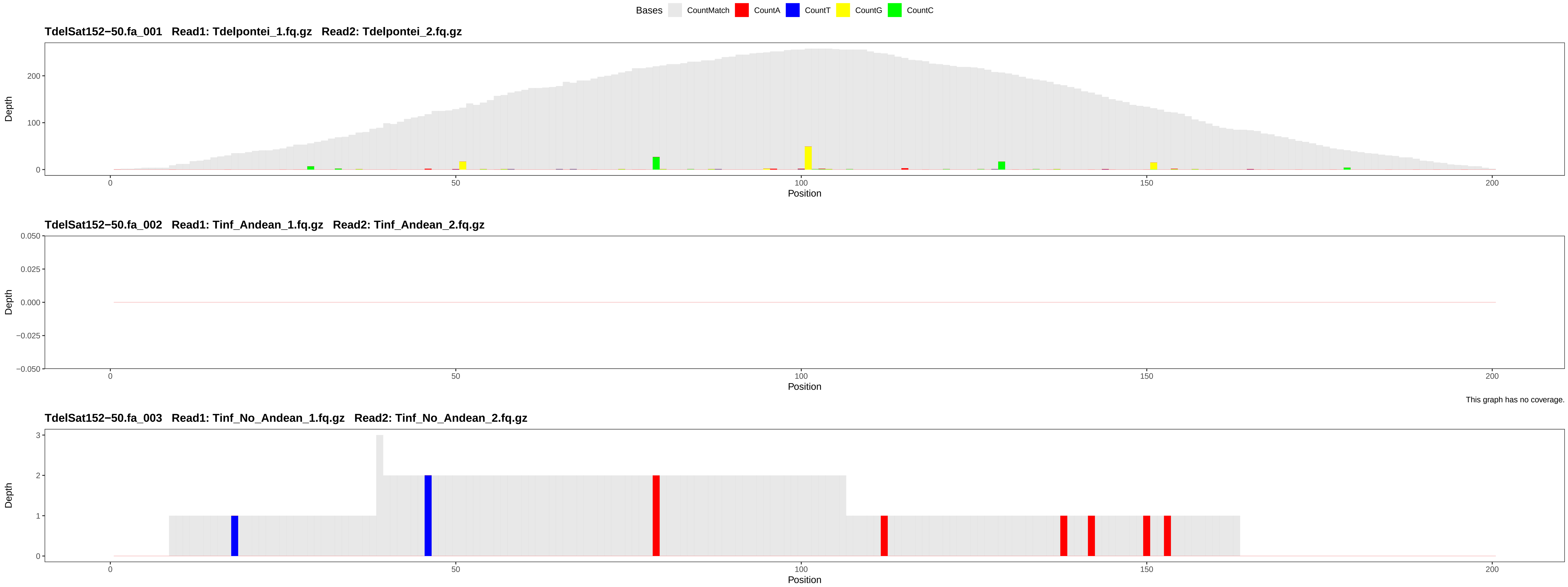
Bases
CountMatch
CountA
CountT
CountG
CountC
TdelSat152−50.fa_001 Read1: Tdelpontei_1.fq.gz Read2: Tdelpontei_2.fq.gz
Depth
Position
TdelSat152−50.fa_002 Read1: Tinf_Andean_1.fq.gz Read2: Tinf_Andean_2.fq.gz
Depth
Position
This graph has no coverage.
TdelSat152−50.fa_003 Read1: Tinf_No_Andean_1.fq.gz Read2: Tinf_No_Andean_2.fq.gz
Depth
Position

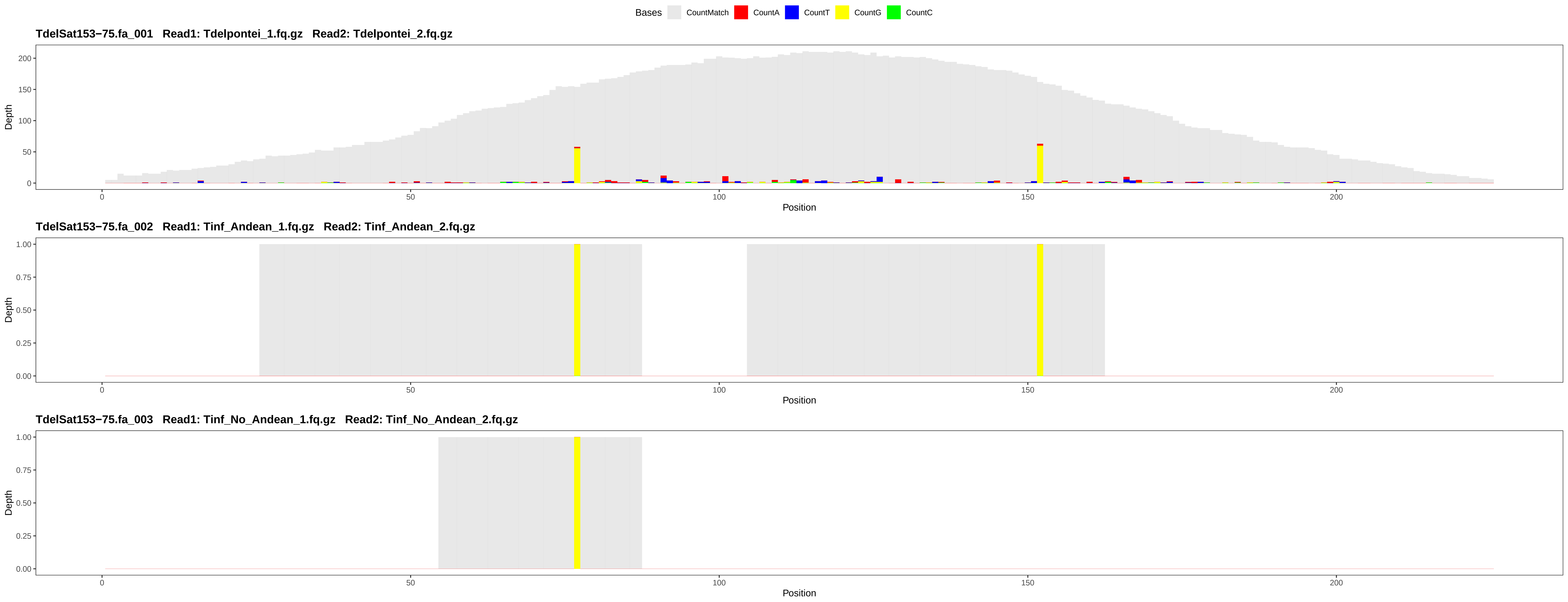
Bases
CountMatch
CountA
CountT
CountG
CountC
TdelSat153−75.fa_001 Read1: Tdelpontei_1.fq.gz Read2: Tdelpontei_2.fq.gz
Depth
Position
TdelSat153−75.fa_002 Read1: Tinf_Andean_1.fq.gz Read2: Tinf_Andean_2.fq.gz
Depth
Position
TdelSat153−75.fa_003 Read1: Tinf_No_Andean_1.fq.gz Read2: Tinf_No_Andean_2.fq.gz
Depth
Position

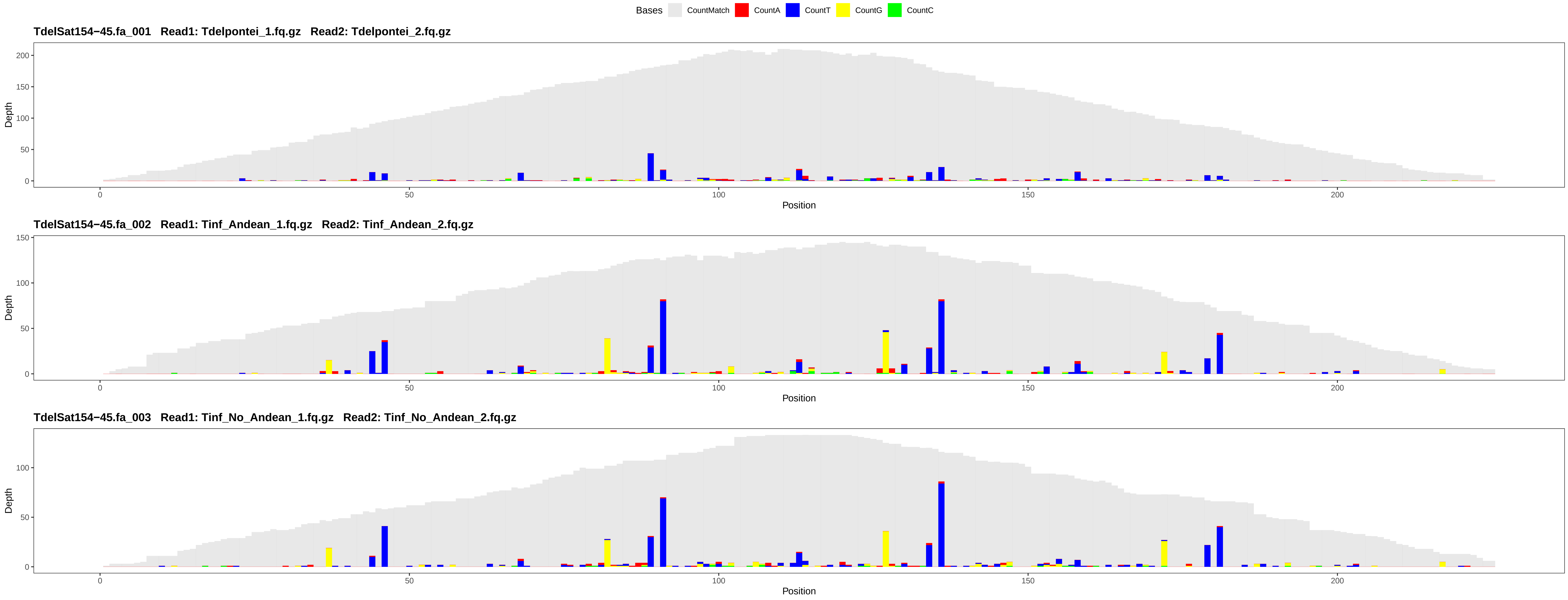

Bases
CountMatch
CountA
CountT
CountG
CountC
TdelSat154−45.fa_001 Read1: Tdelpontei_1.fq.gz Read2: Tdelpontei_2.fq.gz
TdelSat154−45.fa_002 Read1: Tinf_Andean_1.fq.gz Read2: Tinf_Andean_2.fq.gz
TdelSat154−45.fa_003 Read1: Tinf_No_Andean_1.fq.gz Read2: Tinf_No_Andean_2.fq.gz
Depth
Position

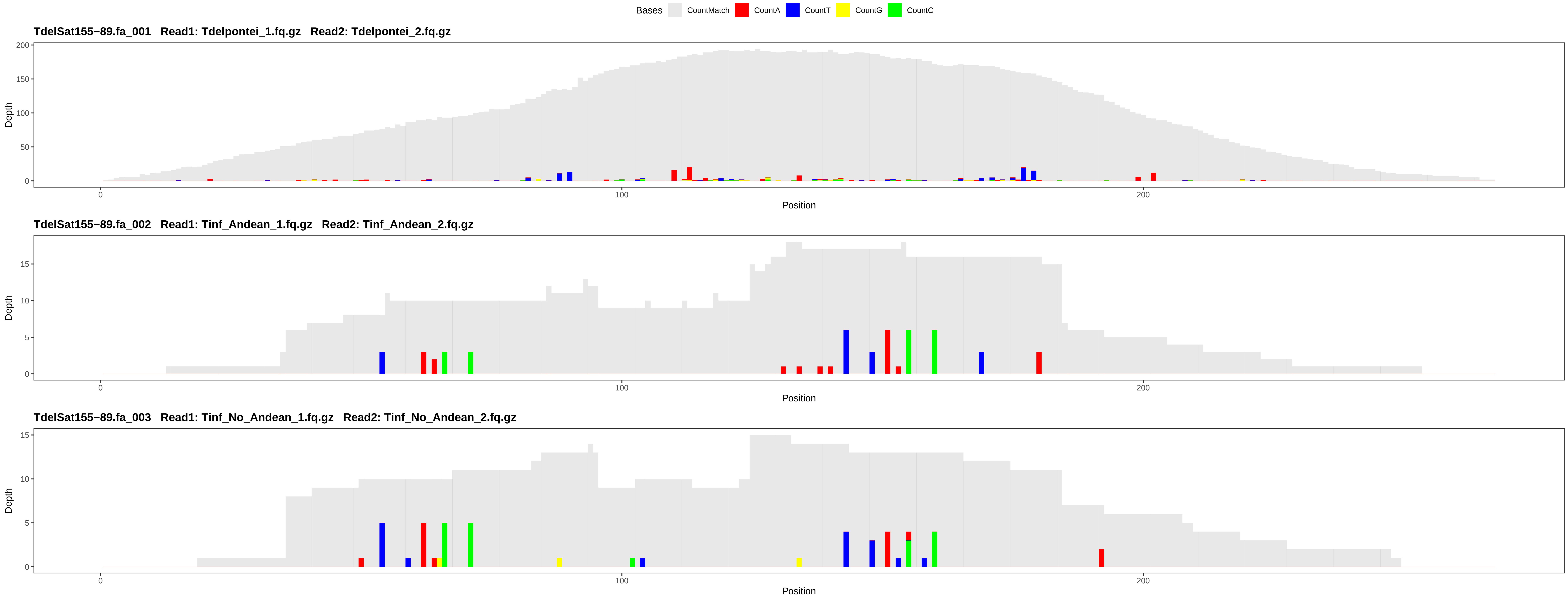

Bases
CountMatch
CountA
CountT
CountG
CountC
TdelSat155−89.fa_001 Read1: Tdelpontei_1.fq.gz Read2: Tdelpontei_2.fq.gz
Depth
Position
TdelSat155−89.fa_002 Read1: Tinf_Andean_1.fq.gz Read2: Tinf_Andean_2.fq.gz
Depth
Position
TdelSat155−89.fa_003 Read1: Tinf_No_Andean_1.fq.gz Read2: Tinf_No_Andean_2.fq.gz
Depth
Position

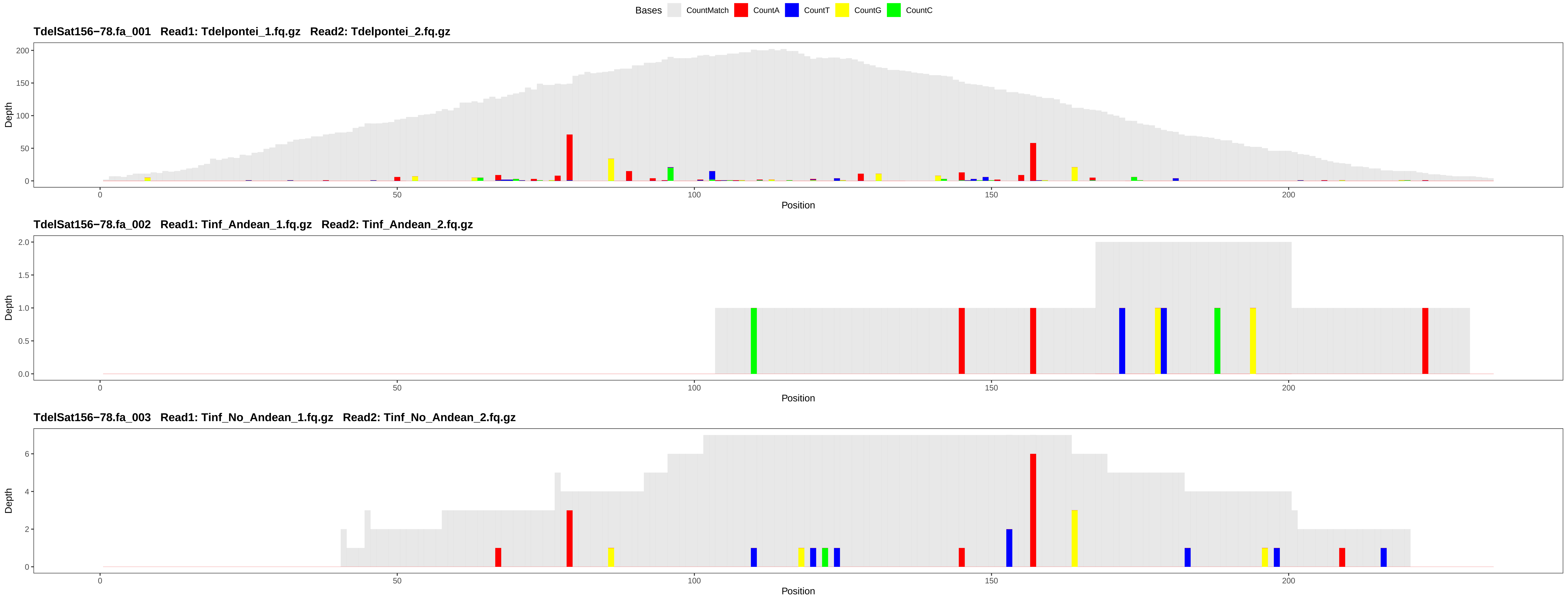
Bases CountMatch CountA CountT CountG CountC
TdelSat156−78.fa_001 Read1: Tdelpontei_1.fq.gz Read2: Tdelpontei_2.fq.gz
Depth
0
50
100
150
200
Position
TdelSat156−78.fa_002 Read1: Tinf_Andean_1.fq.gz Read2: Tinf_Andean_2.fq.gz
0.0
0.5
1.0
1.5
2.0
TdelSat156−78.fa_003 Read1: Tinf_No_Andean_1.fq.gz Read2: Tinf_No_Andean_2.fq.gz
0
2
4
6

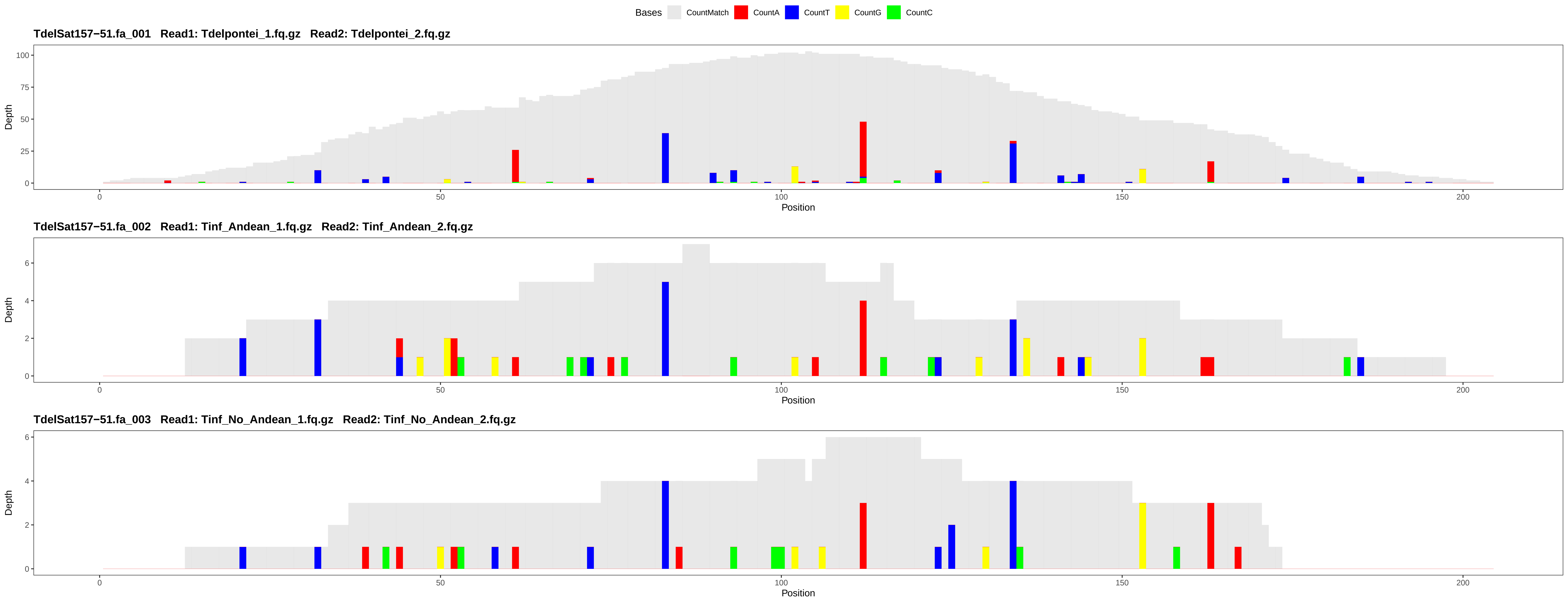
Bases CountMatch CountA CountT CountG CountC
TdelSat157−51.fa_001 Read1: Tdelpontei_1.fq.gz Read2: Tdelpontei_2.fq.gz
Depth
Position
TdelSat157−51.fa_002 Read1: Tinf_Andean_1.fq.gz Read2: Tinf_Andean_2.fq.gz
Depth
Position
TdelSat157−51.fa_003 Read1: Tinf_No_Andean_1.fq.gz Read2: Tinf_No_Andean_2.fq.gz
Depth
Position

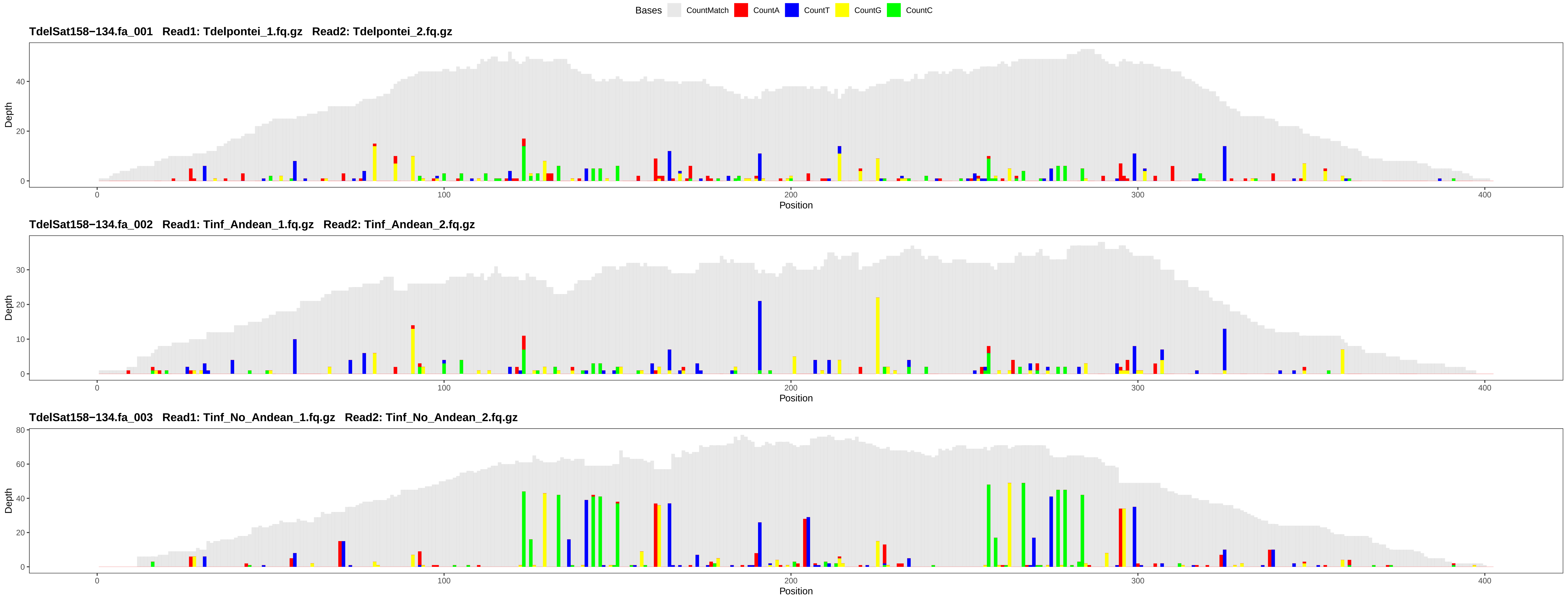
Bases
CountMatch
CountA
CountT
CountG
CountC
TdelSat158−134.fa_001   Read1: Tdelpontei_1.fq.gz   Read2: Tdelpontei_2.fq.gz
Depth
0
20
40
Position
0
100
200
300
400
TdelSat158−134.fa_002   Read1: Tinf_Andean_1.fq.gz   Read2: Tinf_Andean_2.fq.gz
Depth
0
10
20
30
Position
0
100
200
300
400
TdelSat158−134.fa_003   Read1: Tinf_No_Andean_1.fq.gz   Read2: Tinf_No_Andean_2.fq.gz
Depth
0
20
40
60
80
Position
0
100
200
300
400

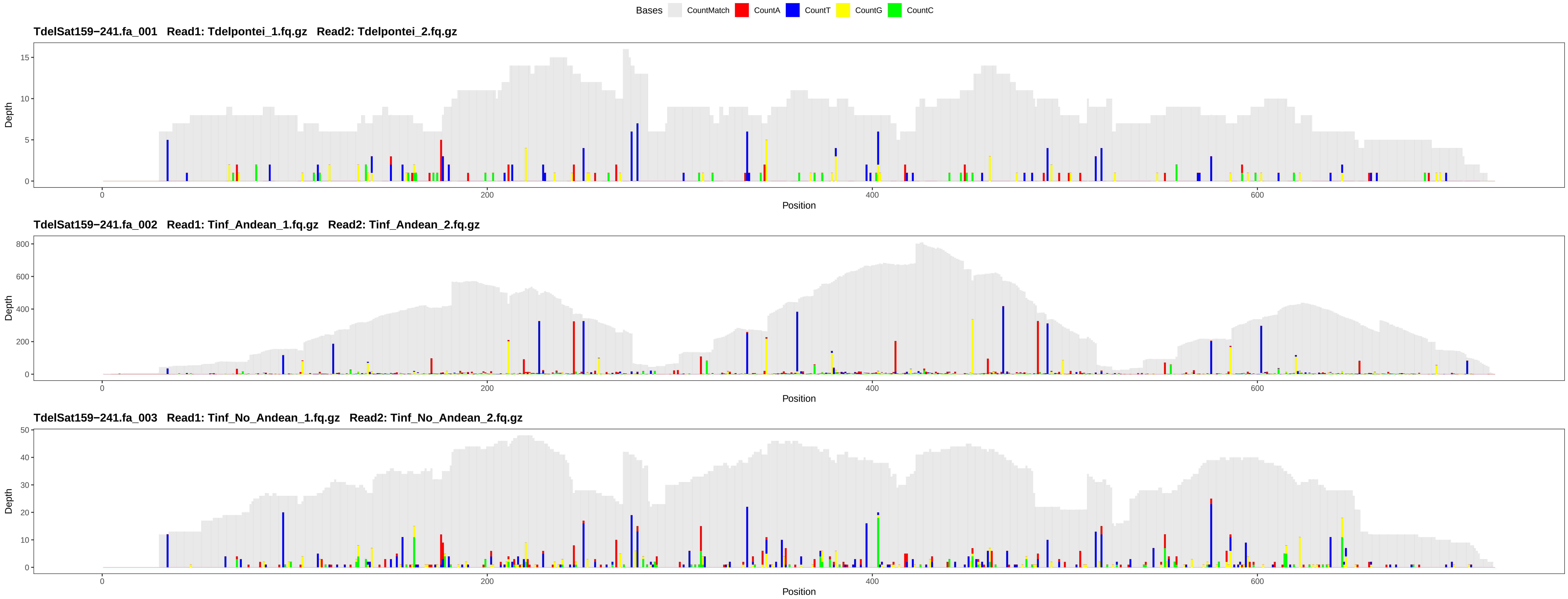

Bases CountMatch CountA CountT CountG CountC
TdelSat159−241.fa_001 Read1: Tdelpontei_1.fq.gz Read2: Tdelpontei_2.fq.gz
Depth
Position
TdelSat159−241.fa_002 Read1: Tinf_Andean_1.fq.gz Read2: Tinf_Andean_2.fq.gz
Depth
Position
TdelSat159−241.fa_003 Read1: Tinf_No_Andean_1.fq.gz Read2: Tinf_No_Andean_2.fq.gz
Depth
Position

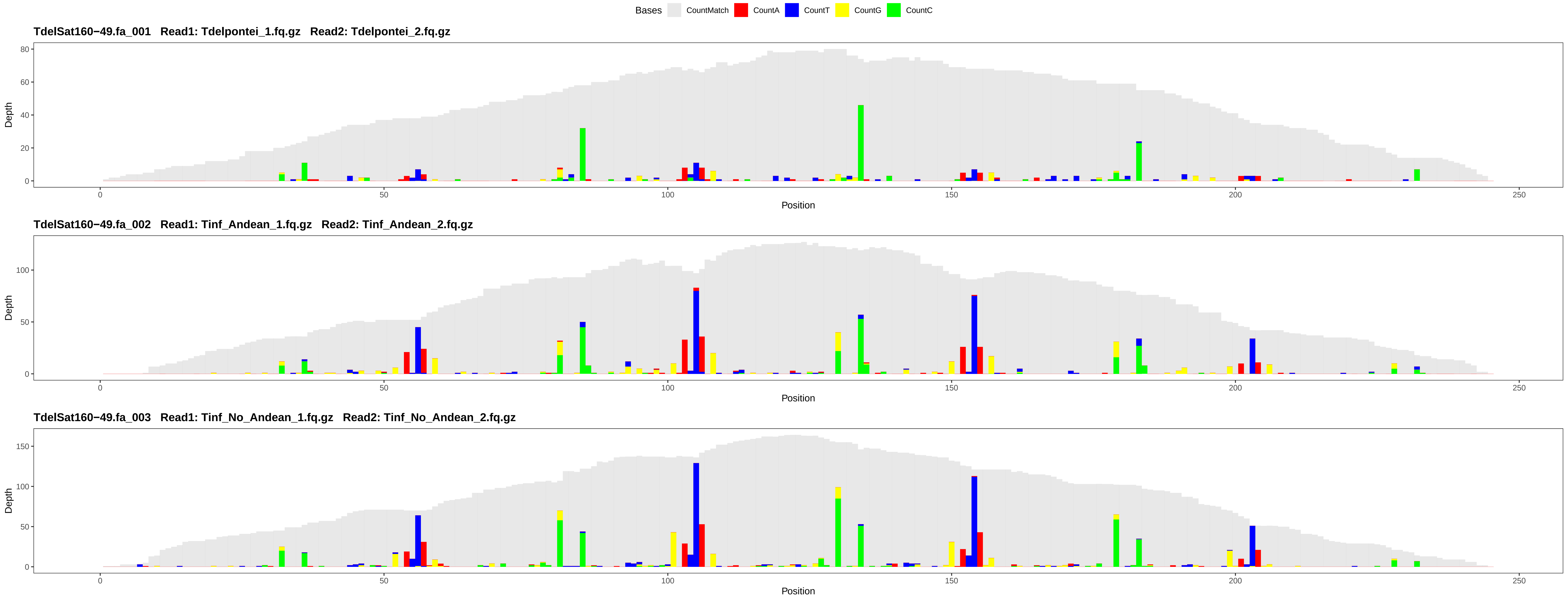
Bases CountMatch CountA CountT CountG CountC
TdelSat160−49.fa_001 Read1: Tdelpontei_1.fq.gz Read2: Tdelpontei_2.fq.gz
TdelSat160−49.fa_002 Read1: Tinf_Andean_1.fq.gz Read2: Tinf_Andean_2.fq.gz
TdelSat160−49.fa_003 Read1: Tinf_No_Andean_1.fq.gz Read2: Tinf_No_Andean_2.fq.gz
Depth
Position
0
50
100
150
200
250
