# Supplementary material for: Making the Genome Huge: The Case of Triatoma delpontei, a Triatominae Species with More than 50% of Its Genome Full of Satellite DNA
Source: Genes (Basel). 2023 Jan 31;14(2):371. doi: 10.3390/genes14020371 (PMC9957312; doi:10.3390/genes14020371)
Supplement: Supplementary file 1 [file genes-14-00371-s001.zip › Table S1 22-12-2022.pdf]

Supplementary Table S1. Data of the satDNA families found in *Triatoma delpontei*: genome abundances (%), divergences, A+T contents, accession numbers and consensus sequences.

Name	Genome abundance (%)	Divergence	A+T	Accession number	Consensus sequence
TdelSat01-79	18.158	12.73	0.59	OQ082080	>TdelSat01-79<br>CCCTAAAATCGGCGTTTCAAACGTAAGAGTGAGTTTTCGAGCAACACTCACTTCA<br>GTTGGTTGTAAGGTTCAAGAAAAT
TdelSat02-8 (GATA-GATAGTTA)	14.21	10.37	0.75		>TdelSat02-8<br>GATAGTTA
TdelSat03-10	12.786	6.73	0.50	OQ082081	>TdelSat03-10<br>RCATACTCGK
TdelSat04-53	3.146	6.59	0.58	OQ082082	>TdelSat04-53<br>AACGGTTTTGGTTATACTATTTTTCCAAACCCGCAATACACGTTTGCCCCTTC
TdelSat05-1000	2.502	12.99	0.67	OQ082083	>TdelSat05-1000<br>ATCGTCGGAGCCATTTTTGAGAAAATCGCAAAAAAGTAAAAAAAAACAACTAGT<br>AAAGTGGTACTTCCGGTTGAGGAATTTTGACAGATAACGTACTGTCAGATCCCAT<br>GGTGATACCTAAACGGAATATCAAGTTTCAACTTTCTACGGTTTTTCGTTTTTGAGC<br>TATGCTGTTCACATACATACATACACACACACACACACACACCATTTGCTAAAAA<br>CCACTTTTTTGGACTCAGGGGACCTCAAAACGGATATTTCCGGTGAAAACTCGAT<br>ATCGAAAATTTGACACGATTACAATACTTCCTCTTACTAGGAGTAAGAGAAAGTA<br>AAAAAAAAAAATCTGGTGTGAAACACTCACACAACTTTCTCTTACTCCAGTTCTC<br>AAAATTATAATTGACAAATTTTATAGTTTGTGTACCAAATTTAATGAAATTCGTCG<br>AAAACTGAAAAAAACTGTTCAGTAAAAGCGCACTTCCGGTTTACTAATTTTCTTG<br>AAACTCGGAATTTTGGCCATCTATTTATTTCTAATTAATTTTGTTTGGATGATAGAA<br>TTGTATAATCAACTATGATAGCACTTTAGAGAAAAACATAACCCCACCCCTCCCC<br>TAAAAGTGCCCTTAAATGAATTTTCCCAGAGAAATGTTTTAAATAAAAGTTGTAG<br>ATCTTTGTATGTGTAGTTTATATACGAAGTTTCAAGAAAATCGTCGGAGCCATTTT<br>TGAGAAAATCGCGAAAAAGTGAAAAAAATCATTTAGTAAAGTGGTAAAGTAAAC<br>GCACTTCCGGTTATCCGATTTTTTTCAAACTCGGAATTTCAACAACATATTTAATTC<br>TAATTAACATTATTTAGATGACAGAATTGTCTAATAAACTGAAATAGCACTTTAG<br>ATGAAAACCTAACCCCACCCCTCCCCCAAAAGTGCCCTTAAATGAATTTTCCCGG<br>AGAAATGTTTCAAATAAAAGTTGTAGCTCTTTGTATGTGTAGTTGATATACCAAGT<br>TTAAAGAAA
TdelSat06-25	0.437	13.14	0.76	OQ082084	>TdelSat06-25<br>AGAATGTATAACTTTGACAAATGTT

TdelSat07-334	0.410	4.61	0.70	OQ082085	>TdelSat07-334<br>TAAACAGGCATAGGATATTATGCGCTAAAAAACATGTTACTTTTTAATCAAAGAA<br>TATTGGAACAAGTTAAACCATTATAAGTTTCTAATCTGGGGAAATGATTGCATTAT<br>ATCCAAATTCGAAATCTTCTGTTTTGTAGCCATTGTCTAACGTCTATTAGTCCAAG<br>AAAAACACAGATATACAATAGAATATAAACATATATTGCACTAAAACATTCTTAA<br>GTTATCGCATGGCAATTATTGTGGATTGCAATAACAGTTTTGCTAGTAATTTCCAA<br>GTTCTTTCCTCAACCTTTCTAAATATGTAACAACTTCTTCTGCCATCTTACATTCT
TdelSat08-247	0.303	12.21	0.49	OQ082086	>TdelSat08-247<br>GGCGCGTCAACATCTATGGTCATTGGCGCCCGGAGCCTTCTCAGTGCACTGCCGG<br>CCTCCGGACAGTGTTCTCTCGTTACAGACAACACTGTCCATCACCGGACAGTGAG<br>CTGTAGAGGCTCCTAGGAAGAGAAAATCTAAGGGTAGCACAGCTGGACTCGAAC<br>CCGGGACCTCCGCGTGGGAGTCCAGTACGCTAACCACTTAGCTATTCCCTTCCCTT<br>TATTATTATTATTATTTTTTAATTTAA
TdelSat09-181	0.173	11.32	0.77	OQ082087	>TdelSat09-181<br>CGGCTCAAAAACAATATTAAAGTCCTTAGAATGTTTTTAGCTAAAATATTATAAGT<br>GTAAACATAATTTAAATATACATAAAAAAAAACAAAGTCTTTGTTCATAGTATTG<br>GATGATAATATATTTAATGTTTCTAGCTAAATATTATAAAATATTAGCCCTAGTCTT<br>GTATGTCATCTCA
TdelSat10-315	0.117	2.97	0.45	OQ082088	>TdelSat10-315<br>GCAGATATATGGCTAGCCAGAGTGGTAGTGGGCTGAAAGCCCGCTTAAAACTGG<br>CTGATTGGGCAACTGAGATTCAATCTGCTGACTATGCCATCACTGCCGGGGCGGC<br>GGTGTCGGAGAAATCGGCGATTAAGGTCGATAACGCCACCTATACGGCAGGTAA<br>TGACATGACCATTAGTGTGACGCTGAAGGATGCGCAGGGTAATGGGGTTACTGGC<br>CAAGCTGCTGCCTTAACGTCCCAGGCGGTAACGGTGGCCAATGCCAGTGAGAAAT<br>CCGGTGTCACGTGGATCGATAACGGCGACGGCACCTATAGCC
TdelSat11-239	0.092	12.63	0.60	OQ082089	>TdelSat11-239<br>ACCCGCGCGCACCTATTCTAAAAATTAAATTCTCTGAAATAAAAGTAAAAGTAAA<br>GAATGCCTATTTTTTCAGGCTGACGTAACATTCAACTAACAATTAGGTATCGTGGA<br>CGGGGGCAGAAACTCCTACTTCACTATTAACATTTTTCCATACCAATATAAAATTT<br>AGCAACTATACGCGGGACTCGAACTGGGGACCTCCCGCGTTCGAGTCCATGCTCA<br>CAACCACTAATGAATTAA
TdelSat12-52	0.086	4.2	0.62	OQ082090	>TdelSat12-52<br>ATATAAATCTGATGTGTAATCACACGACTTTCTCTTAGTGCTGTAGGAGGTG
TdelSat13-4 CATA	0.056	2.13	0.75		>TdelSat13-4<br>CATA
TdelSat14-94	0.053	6.3	0.73	OQ082091	>TdelSat14-94<br>CGGAAAATGTCTCTGATAAAGAATATTATGAATGTAAAGAATGTAAAGAATATTA<br>GAATAAAGAATGTAAAAAACAAAATGAAGCAGGCTACCA

TdelSat15-93	0.053	4.56	0.61	OQ082092	>TdelSat15-93<br>AGAGTGTGTGGTTTCCTCAACAAATCCATTTTATTAAGTTTAGATTCCTCCCAATG<br>ACACACAATGTAAACAAGTACACATTAGGGGAGAGGG
TdelSat16-31	0.053	1.07	0.65	OQ082093	>TdelSat16-31<br>ATATACGGTCAGTTTGGTCACAGTTATTACT
TdelSat17-84	0.045	18.73	0.64	OQ082094	>TdelSat17-84<br>AAAAGACATATGCGAACACATACAGGCGAGAAGCCATATAAATGTAGTGAATGC<br>GATTACAGTTGTACAACAACTGGAAATTTA
TdelSat18-62	0.040	9.18	0.58	OQ082095	>TdelSat18-62<br>TTTTCTGCGATTTTCTCCATAACGGTGGACTAGAAGTGCTACTTCACTGGTGAAGT<br>TCTTTG
TdelSat19-58	0.040	6.26	0.66	OQ082096	>TdelSat19-58<br>TAATCTGAGTTGGAACTATCCATTGTAGTGAACCACATATTTGTTACTATCAGCGT<br>AT
TdelSat20-27	0.035	10.77	0.74	OQ082097	>TdelSat20-27<br>CGCATTTCTTTTATCAAACCTTAATAA
TdelSat21-33	0.034	7.16	0.70	OQ082098	>TdelSat21-33<br>TCTACACACATAAATACCTATCACTGTATACTA
TdelSat22-104	0.033	6.45	0.63	OQ082099	>TdelSat22-104<br>GCTTAGTCAGTTCAATTATACATTTAGTTGGCTGAATGGTGAGCAACCTAATGTTT<br>GTATTTATTAGCGAGAATGTGATGATAGGTTAGAGTGTGCTTGTATGG
TdelSat23-112	0.031	7.68	0.63	OQ082100	>TdelSat23-112<br>GAACCCACGGTGATATCTAAACAGAATGTCAAGTTTGAATTTTCTGCGATTTTTGT<br>TTTTGTTGTGGTTGTGTTTGTTCCGGTTGTGGAATTTTGACAGATAACTTAGTGTC
TdelSat24-147	0.030	11.84	0.63	OQ082101	>TdelSat24-147<br>CCAAGAAAGTTCATTTTATGGCGTTTTGGGTGGGGGGTAAGGGGCAAGCTTTTCCC<br>AAAATAGTGGTTTTTATTGTTTAGATTCTCCCACTGATGTAAAATTGAAGACAACT<br>AAACATCTAGTGAGAGAATTAGACTTTTGAAAGTT
TdelSat25-138	0.028	24.02	0.60	OQ082102	>TdelSat25-138<br>TATGGGGTCACAGGGCGAATAGTAGCGTCAAGCTTTCCTCTACGGTACCACTTCA<br>GTACAAAATACGGTTCTCCCACCTACATACATAAATGCCAACACTTGTATTTGAA<br>GTATTGTTCCAAGAAAAGTGTTTTTTTT
TdelSat26-32	0.028	2.28	0.63	OQ082103	>TdelSat26-32<br>AATTGCCACCGTTTGTCTGTATGTACAACTAT
TdelSat27-57	0.026	2.7	0.63	OQ082104	>TdelSat27-57<br>AAAGAAAACTGAGAAGGGAAAATTCTTTGCTGTTTGTTGTGGAGTAAGGTTACTT<br>GC

TdelSat28-5-Telomere	0.024	1.14	0.60		>TdelSat28-5-telomere<br>TTAGG
TdelSat29-137	0.023	16	0.62	OQ082105	>TdelSat29-137<br>CTACAATGAGATATATATAAAGTGACGAATTTACTTTAAACTTCCAATTTTACTCA<br>TTTGTCCTAACATATAGTTTGGTGATGTTAGGAGGGGGAGGTGGTGGGGGACACT<br>CACACGACTTTCTCTTACTCCTGTTA
TdelSat30-38	0.022	3.24	0.63	OQ082106	>TdelSat30-38<br>ACACACTAAGTGAACAATAGTAATACTCGTCCTTGTAC
TdelSat31-52	0.022	2.76	0.52	OQ082107	>TdelSat31-52<br>ACTTACTGCTCAGTCCAAACTTCACACGGTCACACATGAGGTCGTTACAGTC
TdelSat32-49	0.022	5.33	0.71	OQ082108	>TdelSat32-49<br>GAAGCTGTACAATAGTCAATTAGAATTAACAGAAAATTATGTGAAAGGT
TdelSat33-46	0.022	2.27	0.54	OQ082109	>TdelSat33-46<br>AATGCCCCTAGTATGGTGTAGGTGGTAGAACCGTCTATTGTACTGA
TdelSat34-27	0.019	1.76	0.70	OQ082110	>TdelSat34-27<br>ATGGTTATACATTGTGTTCACTTATCT
TdelSat35-188	0.018	2.49	0.67	OQ082111	>TdelSat35-188<br>TGAGCCATTTCCATTGTTCTATACTCTGTTTCCTCTAGGAGCTAAGACCGGTTCAG<br>GCCCATTTATTTCTAGTTCTATTTTTATTTATTTTGTCAACCATTTTCTAAATTATCTA<br>GGTTAATAAACATGCTATTTCAATCCTACCTTTTCAAATATTTTATACTTTCTACGC<br>CCTGAAGTAGTGGTTC
TdelSat36-128	0.018	13.48	0.63	OQ082112	>TdelSat36-128<br>TCTCAATTACTCATATGTCCTAATATAAATATACAGGTGGTGGGAGACAAGTGCC<br>AGCCTAGCAACACAGACAGGTACAGAATGATTACATTGTAGACAGCCAATAATA<br>TTGAACAAACACATTTGAA
TdelSat37-38	0.017	1.13	0.58	OQ082113	>TdelSat37-38<br>TGGTGTGTAATATTCACGCGCCTTTCCCATATCTATTG
TdelSat38-147	0.016	8.43	0.59	OQ082114	>TdelSat38-147<br>GTTATCTGGTTGGCTTTTTTCCTGCGTTTCAGATTTCGATTCCTTTTCTGCCGCAGTT<br>TTTGATCCCGCTTGAATGAATGGTGGGATTGGTGGAACATTGTCTATTTCAGATAT<br>CGATTTGTCAGCATCTTCACCTTCATTACTTGA
TdelSat39-18	0.016	6.08	0.56	OQ082115	>TdelSat39-18<br>GCTAGTACTGCTCTTACT
TdelSat40-72	0.016	4.92	0.64	OQ082116	>TdelSat40-72<br>TTAGCGAAATGTCTGTATGTATGTGTGAGTGTGTGAGAGAAAGAGAAAGAGAAA<br>CCATCTCCAAAAATGTTT

TdelSat41-49	0.015	2.08	0.61	OQ082117	>TdelSat41-49<br>AGAAGACTGGAGGAGAAGAAGAAGAAGAAGTATGAAATACTGGAGTAGT
TdelSat42-128	0.015	0.72	0.60	OQ082118	>TdelSat42-128<br>CCCTTCCATACCCTTTTGTAATTTTGTTTACAGTGTTCAGTTAGGAGGTGTTAGCCATGAACAGAAAAGTGGAAGTGCTTATATTTGGCCATCTGATTATGTTTGGATCTCTAACTGGAAGGCACTTC
TdelSat43-111	0.015	9.47	0.68	OQ082119	>TdelSat43-111<br>AAAAAAAAATCTGGTGTGAAACACTAACACCTTTCTCTTACAGCTAGCATAGGAAGTACTGTGATCGTGTCATTTTTTTTAAAATTGAGTTTACACCGGAAAGACATATTT
TdelSat44-34	0.014	10	0.56	OQ082120	>TdelSat44-34<br>ATGGACAGTAATTTGGTGGGACCCTGTTGTAACA
TdelSat45-102	0.014	5.9	0.64	OQ082121	>TdelSat45-102<br>TTCGCGGAAAAAAGTTCCCAATAAAAGTGACGGCATTTGCATTCACAGTTACTGTAGGACGTTTAGTGAAATCCGTTGCCATTTTTACTAAATAAATCAACT
TdelSat46-114	0.014	4.71	0.68	OQ082122	>TdelSat46-114<br>TATCGTTACAACTATTATTAATCTCATTACAGAGAATCTCTATATCGTTTTTAGCAGAACATTCTACAATAACTCCGCCATTAGAGATTCCCTTAATGTTTTCTACACCCAATT
TdelSat47-25	0.014	4.16	0.64	OQ082123	>TdelSat47-25<br>TTGCTGGTTGTTGTAAGAAGGTATA
TdelSat48-118	0.014	16.72	0.64	OQ082124	>TdelSat48-118<br>ATACAATTTAACTGTTCAATCGCTTGTACAGCAGTAAATGGAACCATTAACTTGCTACCAGGTGTTTCAAAGTAAAACTGGTGTGAAATATTCACACGACTTTCTCTTACTCCAGTTA
TdelSat49-49	0.014	4.47	0.71	OQ082125	>TdelSat49-49<br>CAAAACAAAAATGATACGATCTGTATACATTCTCTTACACAGTAAGTGAAAA
TdelSat50-101	0.013	12.06	0.68	OQ082126	>TdelSat50-101<br>ATATTTGTAGCAAAATAGTTAATTCTAAAGTAGGGGGGGGAATGCAGAGAGCAGTCAACTTCATTTGAACATAAACATCATTTATTATGTTAACTTACACT
TdelSat51-46	0.013	2.73	0.63	OQ082127	>TdelSat51-46<br>ATGTACAGCCGATACAGAAAGTGTAAATAAAGTTGTCGCTCTTTGT
TdelSat52-88	0.013	7.19	0.73	OQ082128	>TdelSat52-88<br>AGGTGAGTGTTGTACTAAAATGAAATTTTTAGATGAATTTTGGTGAAAATAGTGGATCTAGTATTTTATTGTACATTAAACACAACTG
TdelSat53-48	0.013	4.74	0.54	OQ082129	>TdelSat53-48<br>TATAGAGAAAAGCCTAACGTTACAGTTCTCCCCAGCCCGAACTGTTAC

TdelSat54-189	0.013	17.6	0.60	OQ082130	>TdelSat54-189<br>TTTCAAATTTTGCTCATATATCCCAACATAAATATCCAGGTGGTGGGGGGAGGGA<br>CGAGTGCCAATGTACCACCAGGGCAGGTAGATAACGGATTGCACGGTAGAGAGC<br>GTCAATAATGAACAAACACATTTGAATTTTTAACGTCTCTTGATTCCTCAGAGAAC<br>CACCTAAAAACAATTTTTTTTAAA
TdelSat55-51	0.012	2.9	0.65	OQ082131	>TdelSat55-51<br>GTATACAAATGTATTATATAGCCAGCCTAACTAAACATTGTAGCCGTTCAG
TdelSat56-102	0.012	1.46	0.60	OQ082132	>TdelSat56-102<br>TTATTACTTGATGAGCTAGGGCTAGTCATTACATTTCGTACTACCAGACAAAGCTT<br>CATCTATCAACTCGCAATATTACAGGTAACAGCTGCAGATTCGGAC
TdelSat57-271	0.012	18.68	0.70	OQ082133	>TdelSat57-271<br>TCTACACATATAAAATCCCTTTCACTTTTTATTTGTAAATTTTTTTTGAAAAACATG<br>GATTTTAATGTAGTTTTGCGGAATCTGTAGCGTTGAAATTCAAATTTTATATGGGCT<br>TGATGGTTGAATGTTATTGGCACACTCTTCTGGCCGACTTGTCATCACTTCTACTAT<br>CCTACTTTTTTACTTTCTCTTACTCCTACTAACAGGAAGTATTTTATTTTTTTTGTTTT<br>TGCGATTTCCTCAAACAATGGCTTCAAATTTTTGTATACTA
TdelSat58-84	0.011	1.99	0.62	OQ082134	>TdelSat58-84<br>CTGACAGACAGCTGACGCTATATCAGAATACCATATTTTTCTCTTGTTTGAAGATG<br>TGTGATCTCCTTCTAAGATTGTGCATTC
TdelSat59-63	0.011	12.44	0.41	OQ082135	>TdelSat59-63<br>CGTGGCCTAAGTGCTCGTCCAGTTCGATGATTGGTCAGTTGGAGAGGGGTGGGGG<br>TAAGTGGG
TdelSat60-99	0.011	9.24	0.69	OQ082136	>TdelSat60-99<br>TACGTATTGCGTCATACCGTGCAACATGACATGTCTCAACATGTTCATTTTAATTT<br>ATTTCTTACTCTTGCTAAAACATCGAAAAAAATACTAGATTTG
TdelSat61-315	0.010	15.5	0.62	OQ082137	>TdelSat61-315<br>ACAATTTATAAGGCCGAGACTGTAGGTGAAAACCTGAAAGCGACTGTAAAGCTT<br>CCAGATTGGAAGGGTACAACTGAATCAGCTAATTACGCAATTACTACCGGTAACC<br>CTGCGTATATGAAGTCAACAATTACGTTAGATAAAGACATCTATCCCGCAAATAG<br>TGATATGAAGGTTACGGTAACCTTGAAAGATTTGTATGGTAATAGCGTAACTGGA<br>AAAGTTGCAGAATTAACTGATGCGGTAGTAACAGTACCAAATGCAAAAATGAAA<br>ATAGGTGAAAAATGGAAAGAAAATGAAGCTGGGACCTATACT
TdelSat62-53	0.009	0.4	0.68	OQ082138	>TdelSat62-53<br>TCACACATTATAGAGGCGAAACGGTTTGGTTTTCCAAATTTATGTTTCATTTA
TdelSat63-51	0.009	1.94	0.71	OQ082139	>TdelSat63-51<br>GGTTGGTTCCAACTAAATTAGCAATTAATAGAAAAACATAATTGTTGTGGT
TdelSat64-39	0.009	5.81	0.82	OQ082140	>TdelSat64-39<br>ACTAACTACACACATAATTATTTAAATTATATATCGTAT

TdelSat65-71	0.008	6.75	0.62	OQ082141	>TdelSat65-71<br>GGGTCAGGTGGGGACAACTGGCTGCTTGTAAAGTGATTGCAATAATACAAAATAC<br>GATACATAAATATAGA
TdelSat66-64	0.008	0.5	0.78	OQ082142	>TdelSat66-64<br>AATGTGCAAAATCTAAGTAATATTTATGACAAAGTTCTACATTCTAATACTAATAT<br>AAAATGGA
TdelSat67-83	0.008	2.95	0.71	OQ082143	>TdelSat67-83<br>AAAAACATATACATATCACCCCTGTACTATTGATTAAGCTCCCAAATCTCTTTAAC<br>ATAACTTTACACTATTACTATACTTAG
TdelSat68-160	0.008	7.98	0.63	OQ082144	>TdelSat68-160<br>TAGTGCTGCGTTTGTGCCGGCGATTCACCAAATTTCATCGTTTGTGCCGTAAAATT<br>GACAAAGATATTAAAGAAAAAAAGTGGCCAGTCCAACTTGACGTCAAGTTAGAC<br>TGGCCACCAATTTCTATTCACTTTTTTAAAATTTTCTTTTTACCAATCGT
TdelSat69-52	0.007	0.33	0.63	OQ082145	>TdelSat69-52<br>GTATATAGATAAGTGGTGGGTGTAATTTGTAGAGAATGTTGTCGTCTGTTCA
TdelSat70-86	0.007	8.15	0.58	OQ082146	>TdelSat70-86<br>GTACCACTGCTTCTAACACGAACGGAAGGTTAGTGGCAGTTTGTGATACCTAAAC<br>GGAATGTCAAACTTGGTATTCCGTTTATGTA
TdelSat71-102	0.007	0.85	0.64	OQ082147	>TdelSat71-102<br>GGAATTTAAATAGAGTCCGGTTACAAACGGTAATGAATTATTTTTTTCTAAGCCAC<br>TATAACTAGGGCTAGTAGCAACCAGCACTGTCTATCAGCTCATTTT
TdelSat72-85	0.007	6.2	0.60	OQ082148	>TdelSat72-85<br>ATAACATAAATATGGAGGTGGTGGGAGATAGGTAAATACCTCTCTACTCAGCTGG<br>CAGGTAGACAAGTGTTTATGTATTTGTCCT
TdelSat73-372	0.007	4.41	0.58	OQ082149	>TdelSat73-372<br>TAGTGCTGCGTTTGTGCCGGCGATTCACCAAATTTCAACGTTTGTGCCGTAAAATT<br>GACAAAGATATTGACGAAAAACTGTTCGTCGTACTTGAAAACCAAAGGCCATGG<br>CTGCGAGTAACATTTGAATGTGCGCGTGCTCCACTGACCGCCAACGGCCCTTATC<br>AGGGCCAGCAACTAAAATGTACTGGCCCTCGATTTCTAATCAAAATTCTGGAATT<br>TTTTCTTCGCTATTCTTAAGTGATTCATTTGTGCCAGCGACCCACCAAATTTCAGCG<br>TTTGTGCTGCAAAATTGACAAAGTTATTGAGGCGTCAAGTTAGACTGGCCACCAA<br>TTTCTATTCACTTTTTTAAACTTTTCTCTTTGCCAATCGT
TdelSat74-137	0.007	4.77	0.62	OQ082150	>TdelSat74-137<br>TCAATATTATGATGGTCGCGGCTACAGATTCTGTAAAAATTCCATGATTGAAGAA<br>AATTTGTCCCGTCGCAAGTCTTCCTCTTACGAGGAGTAAAAGGAAATAAAAAACT<br>AATAATGTACAATTATGGCCCCCCCCA

TdelSat75-25	0.007	1.7	0.71	OQ082151	>TdelSat75-25<br>TTATATAGTTGGTGTATAGTTCATTGTACTTGGTA
TdelSat76-96	0.007	7.29	0.70	OQ082152	>TdelSat76-96<br>GGCAAGAAAGGTAATGAGTACAAAGTTTTATTTTTACATAATAGGATTAAGTATT<br>ACTAAACATGTTAATAAGACCTGTGGTAACATGTCCGAACA
TdelSat77-102	0.007	1.36	0.79	OQ082153	>TdelSat77-102<br>CAAATTTTAAATTTTCGCTTTTATTGTAAGTATGCTTTTTAAATGCGATTAACATTA<br>TCATTAATCAAAATGTTAAGTCTTAATATAGATTAGTTATGTACA
TdelSat78-29	0.006	1.95	0.59	OQ082153	>TdelSat78-29<br>CTGTACAGAATAGCTAAGTGTGTGTGTGA
TdelSat79-26	0.006	4.22	0.62	OQ082155	>TdelSat79-26<br>GTACCTAACACCATCTACACAGATAA
TdelSat80-52	0.006	7.61	0.42	OQ082156	>TdelSat80-52<br>GGAGGGGGGTGACGTGGGACTTTCCCCTCTGCGATATCCTTGTAGCGATTGT
TdelSat81-185	0.006	12.38	0.68	OQ082157	>TdelSat81-185<br>CACACACCACACACACACATATATATATAGAAAGAGAAAGAAAACAATTTTATA<br>AAAATCACATTTTTGGACTCAGGGGATCTCAAAACATACACTTGCAATTTTATTTA<br>ATTGAAACTGCGTGTCGTATTCCCTAGAGATATCCATAATATGAGAGTTATATTGT<br>TTTCCCTCTCACACGCATA
TdelSat82-108	0.006	3.6	0.60	OQ082158	>TdelSat82-108<br>CCTTCTCACTTGGCAAAATTAAGAATGGTAAAACCCCTAGCTGCAACAGTAATTT<br>CTTTGGTGTATCACACCCGTTCCTAGAAGCCCAAATTACCCTAGAAGAATTAA
TdelSat83-66	0.006	7.66	0.68	OQ082159	>TdelSat83-66<br>GGTACATCTAACAGTTTGTATAGGTACCTCTAACAGTTTATATAGATACATGCAAC<br>AGTTTACATA
TdelSat84-73	0.006	4.24	0.52	OQ082160	>TdelSat84-73<br>GTTTCTCCTGGTACGAGGAGTACACCAACTTGGTATGTCATCGGAACTACCCATG<br>GCATCTACATGACTTCCT
TdelSat85-49	0.006	0.04	0.71	OQ082161	>TdelSat85-49<br>TATTCTCTACTTTCACCAATGTACATAATTGTCAATGTTTTGTCCAATA
TdelSat86-164	0.005	6.95	0.62	OQ082162	>TdelSat86-164<br>AATTGTTTGGAGTTAAATAAGGTGAGTGGCCAGTGCTGCTGTGCTGGTCACGTTTG<br>TAGCTTTGTTTAACATAATCCTTCAATTCTACCCACAGCTTGCCGTTATGTTAGTTC<br>TTAGGAATTAACATATATATGCGGCGCCTCTTTTATTTTATTTTAGCGTAG
TdelSat87-61	0.005	1.49	0.67	OQ082163	>TdelSat87-61<br>CCTGTAATCAGTAAAGGAACAGATAGAAATAAGGTCCTCATTTCCCAATTATCTA<br>TTAGTA

TdelSat88-87	0.005	16.82	0.71	OQ082164	>TdelSat88-87<br>TAACTGCAATGAGACCAAGTCGTCTGAATGTTTCACACCATTGAGTAACATTACA<br>ATATAAAGAAAATTTGTAAATTGTAATTGTAT
TdelSat89-86	0.005	1.93	0.78	OQ082165	>TdelSat89-86<br>GTAGGTACAGATACAGTTACGGTTTAAAATAAATAAATGAAAATAAAATAAAAA<br>TGATATATTTCTAGATTACATGTTTACAGTTG
TdelSat90-157	0.005	3.42	0.75	OQ082166	>TdelSat90-157<br>TTTGTAATTTAGAATTAGAGAAATTTGTAAATGTAAATATAAGCATTAATTTTGGT<br>ATGGATATTTACAAACATAGTTTACACTTTAAAATGTATGACAAGCGATTAGAAA<br>AAGTATCAAGTATTTTATTATCCTGACAGGAAATGGCGCGTTTAAA
TdelSat91-23	0.005	0.87	0.70	OQ082167	>TdelSat91-23<br>ACATGTGTACCAATGTAAACAAT
TdelSat92-196	0.005	8.89	0.67	OQ082168	>TdelSat92-196<br>TTTGTTTTATTTTTCTATTTATCCTGACATAAATATATAGAGGGGAGTAAGGGGATC<br>AGGACACTTATCCACTAACCTGGACTGGTAGGGAAGTGATATATTGTGATCAAGA<br>AGAAAGCACCAAAAACACTGAAGATTTGCTGTTACTCTGTCAGTACAATTATATA<br>CAAAAGGACAAATTTTCTTTAAAATCAAT
TdelSat93-57	0.004	4.09	0.61	OQ082169	>TdelSat93-57<br>GATCAATTTTGACCAAAACCTTAGTATCTATTCAGTAAAGTGGTGGCACAGGAGG<br>AT
TdelSat94-72	0.004	7.29	0.65	OQ082170	>TdelSat94-72<br>TCCAGCCACACTAACTTCAAATATTAGAATAGTTTTCGCCATTAAACCATATCACC<br>AATCAACGTTCTAACA
TdelSat95-38	0.004	6.35	0.63	OQ082171	>TdelSat95-38<br>TAACCCGTAGATATTGTTGGAGGAATATTGCTGGAATA
TdelSat96-58	0.004	1.79	0.74	OQ082172	>TdelSat96-58<br>GATTTGATTATGAACCTTAGTTACAGCCTTTTATAATTTTATTTTTCCTAAATTGGG<br>T
TdelSat97-238	0.004	5.81	0.71	OQ082173	>TdelSat97-238<br>CCAAACAACAATAATATGAACGGTTAACGGTCGTTTTTTTCCATCAGTTACCATTC<br>AAACAGTTACAAAGTAGTGCAATTTAACATTTTTTTAATAAACTATTTCCATGTTTT<br>CCAATTATTTCGTTATTATTTTCTCTTTGAACACTATATCCAACTTGGCACATTCCA<br>ATCTAACAAAGTTTTTATATGTTTTCCACCTATATTTTATTAAAACACTACACCCAC<br>CTTGGAACATT
TdelSat98-104	0.004	1.1	0.65	OQ082174	>TdelSat98-104<br>AGTGCAAATAGAATTTAAATCTAACGCCACACAGATTCCTTACAACTCACTCAGT<br>ATCAATTTTCTAATGTCAGAGTACATGATTACATCATTGTCCAGAAGTC

TdelSat99-128	0.004	4.57	0.56	OQ082175	>TdelSat99-128<br>GGGGTAAATTTGAAATATATTGGTGGCTGGTTGTTTGTTATTGGCGCGAACCATAT<br>TTCCCTAGCTGCTTGCCTTGCTAGCTCCCTCCATTTACGCTACATACAAGAAAGTA<br>CAAAATTGTGGGGGGA
TdelSat100-99	0.004	0.23	0.66	OQ082176	>TdelSat100-99<br>TCAGAACATACATACAAAAAAGTAGCGTGACTTGTTTTTCCTCTTGTTGTAATCGA<br>AAACTGTCATGCCCAGCTTGTCACCATATATTGGTTATATTAT
TdelSat101-55	0.004	1.2	0.71	OQ082177	>TdelSat101-55<br>AAAATTGTAAAACAATAGCCACCTCAGAAAGAAAAGTGTTATACTAGTATTGGAT
TdelSat102-39	0.004	3.44	0.74	OQ082178	>TdelSat102-39<br>TGAAGTTAGATTTTTGAAAATTATATTCTGATCGATCTG
TdelSat103-138	0.004	2.07	0.70	OQ082179	>TdelSat103-138<br>TACATTTACAGTTGCCGAAAGTATTTCGAACTATATCTTGTTTCTGGAAATTGGTC<br>GCCCTCCGATTTATTTATTGAATAGTTTATCTAAAGGATGTACTAGCACACTGCTA<br>TTAGAAATAATTCCTTTAAAAAAATA
TdelSat104-78	0.004	1.82	0.62	OQ082180	>TdelSat104-78<br>CGAAACTTGACAATGAAATATCTTTATTTTTATTGCGGTTATAGTGAGACGCGCCC<br>TAATGAGCTGATGAGATACTGC
TdelSat105-35	0.004	4.44	0.57	OQ082181	>TdelSat105-35<br>CTATCTGCCCACATATAGTTCACTAACTTGGCATG
TdelSat106-44	0.004	5.73	0.57	OQ082182	>TdelSat106-44<br>TAGGTAGTGCTAACACCAACAGAATCAATATTACCCCACCACTC
TdelSat107-57	0.004	10.56	0.58	OQ082183	>TdelSat107-57<br>GAGCCCCGCCTACACCCATAGTATAGCAAACTAAAATAGCACATTAGAGAATAG<br>CAT
TdelSat108-81	0.004	6.98	0.49	OQ082184	>TdelSat108-81<br>TACAGCAGATTGTATGGACTGCCCTCACTAGGCTGGTCCAGTCCAATCACAGACC<br>CCCCATATTTAAAACCCCAAATCCCC
TdelSat109-142	0.004	3.73	0.61	OQ082185	>TdelSat109-142<br>TAGTGTTTGATGTTCTTTAAGACCGTTGTATTTGGATATTGTATGGAATGGATGTCG<br>TGGTTGGGTCAGTATCCGACTAATATAGTAACAAATAAGAACAACACACTGTCCG<br>AACCTCTGCACCCATTGACAAGCTCAGTAA
TdelSat110-99	0.004	2.61	0.78	OQ082186	>TdelSat110-99<br>ATTTATAAAACAATACATATCTAAACTAAATATACCAAGATAACAAAGTTATAAA<br>CCAACGATACAAGAAAACCAAACTGTAAAAGTAAAGTTTAAAAC

TdelSat111-7	0.004	15.29	0.86	OQ082187	>TdelSat111-7<br>ACAATTT
TdelSat112-113	0.004	2.75	0.55	OQ082188	>TdelSat112-113<br>GTAGTGGTGTCCGTGAAGAATCGCAGACAGCATTGGATTGGCAGAGAGGATTAG<br>AGTGCATCCTGGCTCTGGCGAGTAGTTTGTACAGTCCTTGTTTAATAAAGTCACTA<br>ATA
TdelSat113-88	0.004	3.18	0.57	OQ082189	>TdelSat113-88<br>CCCTCTGCCTCTCCATTTATTATGTGATGTTATTGGCAGTACATTGTAATAGCACTT<br>CTTCTACCTGCCTGGGTAGAGTTACTTGTCC
TdelSat114-56	0.004	5.82	0.68	OQ082190	>TdelSat114-56<br>ATTTCAATGCACTATAAACAATAGTTATGTCTGGCCGTGGTTAATTAAGAGAAAC<br>T
TdelSat115-15	0.004	4.85	0.73	OQ082191	>TdelSat115-15<br>GTAGTGTTTAGTAAA
TdelSat116-66	0.003	2.22	0.79	OQ082192	>TdelSat116-66<br>AAGTAATTTTTCTGAGTAGAAGTAAAATATAAAGAAATTATTTTGGTTTGTCAAAA<br>TGACTAGAAT
TdelSat117-46	0.003	9.49	0.67	OQ082193	>TdelSat117-46<br>ATCTAGTAAACAGTTCTCCCACCTATATAATATTACAGTTACCATC
TdelSat118-207	0.003	6.97	0.62	OQ082194	>TdelSat118-207<br>TCAGTGGGCTTTTCGGTTACTCTAGTTGTAGCTTCAGTATCATGAATTGTAGATTGT<br>TCTGTGATAAGTGTAGTCGGTTGCTCAGTGTTTGTTGTAAATTCATCAGCGGTTTGT<br>GCATCCGTGCTAGATCCTATGTAAGATGCAGTATGGTCATTTGTGTAATATTTCGG<br>GTTATTTGGTGTATTCGGTTTGAATGTTGTTATAATA
TdelSat119-348	0.003	4.01	0.55	OQ082195	>TdelSat119-348<br>GTGAAGCCTATCACAACTTCGGCAACCTACACCGAAGAGTTCTCAACAATACAAT<br>CTTCATCACCTCATTCCACAGAACCAACATCAGAAAGTTATACTACTAACAGCCA<br>TCGCACAACTGAATCAGAATCTACACCAATCACACAATCAAGCCCAGAGATACA<br>TACCATATTTACGTCCGCGTCCCCATCTACTGAGAACAGTGCAACAACAACCGCC<br>ACCGCGCACACATCACAGCCTAGCTCTGAAACATTCAATACAATTAAATACACTA<br>CGGGTCCGGGCACTGAAACCTATTCAACAATAGTGGGTTCAGGCTCGACATCTCA<br>CAGCGAAGGTTATACTTCC
TdelSat120-101	0.003	2.83	0.74	OQ082196	>TdelSat120-101<br>GTAATGAAAAAATAAAGTATTTAATATCTAACTTGATAATTGAAAACATCCAATT<br>AAAATTTATTAATGCTTGCCCAAAGGCCGGTCTCTATACACATATT

TdelSat121-51	0.003	11.93	0.63	OQ082197	>TdelSat121-51<br>CTTTCTCGGGAAAAAGTTCTTCTTCCCTTCCCCAAAACTTCTTTCAATAAA
TdelSat122-43	0.003	4.33	0.72	OQ082198	>TdelSat122-43<br>ACTTAAGTATTGTAAAATTGAAGACAAGCAAACGAAATTGAAG
TdelSat123-149	0.003	2.82	0.77	OQ082199	>TdelSat123-149<br>TTTTTACAACTGACTTCGTCTATACAAAAAGGAACCTAATTCCTAAAATACAATTC<br>GAAATATTTTAGTTTTTCTTACTTTTCTATAGAAATATTAAGAATATTCTTATAGAA<br>GATAATGATTAATATGAGTGTATGAGGTGTAAAATG
TdelSat124-69	0.003	1.85	0.65	OQ082200	>TdelSat124-69<br>TAAGAGGTCTTGAATAAGTAATAGTAGTAGTAGTAGTAGAAATGTATAAAACCTC<br>TACACCGGGTGGTA
TdelSat125-35	0.003	9.52	0.54	OQ082201	>TdelSat125-35<br>TCCCCATCTCCATTTTAGAGGTAAGACACTTCCAC
TdelSat126-46	0.003	6.94	0.54	OQ082202	>TdelSat126-46<br>TACACATCTACCAGCACATCAACGACACATCTACCAGCACATCAAC
TdelSat127-49	0.003	5.5	0.65	OQ082203	>TdelSat127-49<br>CTGTACAGTGAATTGAAATAACACATTAGAGGAAAGCATAACCCCATAT
TdelSat128-66	0.003	0.23	0.73	OQ082204	>TdelSat128-66<br>AAAGGAAAATCAATCTGACAACTATTAACTAAAACAAACTGCTATTAACGAAAC<br>AACAAACACATA
TdelSat129-60	0.002	6.5	0.63	OQ082205	>TdelSat129-60<br>ACCGTTATATGTTTCTATTTATGCTGCAAGCCTCTGCCAAGTTTAAAGAAACTCTCT<br>GTT
TdelSat130-105	0.002	0.6	0.72	OQ082206	>TdelSat130-105<br>AGTGGACAAACAACAGTATAATAAATTGATTAGAACCTAAATTGACTTGACTAGT<br>ATTTCTCTGTGGAATCTTAAACATAAAATACAATGTCCTTCATCTAAATT
TdelSat131-66	0.002	1.42	0.65	OQ082207	>TdelSat131-66<br>ACAATGGTGTAAATACTCTGTCCAGTTGTCCTAATAAAAAGAAGATGACATACAT<br>GTCAGATCTAG
TdelSat132-39	0.002	1.82	0.72	OQ082208	>TdelSat132-39<br>AATAGTTGTATAAACTGTATTCACAGAGTAAGATTGTTG
TdelSat133-28	0.002	3.73	0.61	OQ082209	>TdelSat133-28<br>AGGTGTAGTTGCTGAGATAGATGTAGAT

TdelSat134-32	0.002	8.44	0.72	OQ082210	>TdelSat134-32<br>GGAAATATTACGATCGTATTAAAACTGTAAGA
TdelSat135-105	0.002	7.8	0.58	OQ082211	>TdelSat135-105<br>TTTCCTCTAAACTGTCATTGAAGTGGGATTCTATAGGAGGGGAAATGTTCGGCATT<br>CCTCTAATCTGCTATATAAGTGGGTTCTATAGCAGAGGTAATGTTCGGC
TdelSat136-61	0.002	1.46	0.64	OQ082212	>TdelSat136-61<br>TATGTGGACAAAATATTTATAGCAAGTGACGTGTGTAGTATGCTAGTGTGTAACGT<br>AAAGG
TdelSat137-183	0.002	7.1	0.69	OQ082213	>TdelSat137-183<br>TTTTCTACACTTTTACTAAAAATACTGGCATATTTCTACACTTTTACAGAAGGCAC<br>CTACATGTTTCTACATTTATACTTATACCTATTACATATTTCTTCAATTTTACAGAG<br>GACACCTGCATATTTCTACACATTACAGGGCATATATATATTTCTATACTTTTACA<br>GAGGACACCTGCAT
TdelSat138-63	0.002	3	0.75	OQ082214	>TdelSat138-63<br>CAGCAATAAGAGAAAGTAAAAAAGAAGAAAATTTGCCATTTTACATATAATTATT<br>GCCACTAA
TdelSat139-152	0.002	0.73	0.66	OQ082215	>TdelSat139-152<br>ATTCCTAGTTTTAAGAAAATTTGTCAGTAATAATGACTGTATTGACTGCAATAAAG<br>TCAATCCAGGACTGTATTTATGAAGTCCCTCGAATGTAAATAACTAAAATCATGA<br>ACCCAACAGTGTGTTGTTAGGCCGACTAACCGTGAAAATAA
TdelSat140-68	0.002	0.99	0.60	OQ082216	>TdelSat140-68<br>AAGTAATTTTTCATTGTAACCACTGTGTCTCAGACAAACTGCGTCACTCGGAGTAA<br>GGGGACTTTAAT
TdelSat141-369	0.002	6.38	0.69	OQ082217	>TdelSat141-369<br>TCCTGACGCAAAACATACCATACAGTATCATTTCCCAGAGTAGAATAGAAACAA<br>GATATGGGGTTCAGTATTAGTCAGTTTGAGAAACAGTGATGAAAACGAAGAAATT<br>CGCTACTTCTTACCAAAAAATTTTACAGAACAAATCACTGATGACGTAATTAAGG<br>ATTATAAGAATAATAAACCATCACCTATCACATATCTATAGGAGGTTGAACCCTA<br>ATAAATCTTTTAATATTCATTTCGCCTAATGGGACACATGACCTTGATGAGTATGT<br>ATGTTTTAAAGTTAAAATGTACATAACTGATGAAGATTTTTTATGTCCTGAATGTG<br>CGCAACCATTAGAATTAGAGGACAAATTAAATATAAAA
TdelSat142-23	0.002	6.6	0.65	OQ082218	>TdelSat142-23<br>TACACACATTGACAGTCTATACT
TdelSat143-52	0.002	2.04	0.67	OQ082219	>TdelSat143-52<br>CTTTCTAATACTCACCAACTGAGAATTGCAAAAGTTGTAAATCTCGTTGTTA

TdelSat144-121	0.002	0.23	0.69	OQ082220	>TdelSat144-121<br>GTATACTTGTTTTCGAGCCCAATGAGTATAAAAAAAAATTGCTGATGTTCTGCTGA<br>CATCTAGACGGACATTCTCTTAAACTAAAACAGTTATATATATAAAATGAGAAAT<br>TTTTCATGCG
TdelSat145-36	0.002	5.61	0.75	OQ082221	>TdelSat145-36<br>TGTACTACTTATAGGGAACAAATGAAATACTATTTA
TdelSat146-62	0.002	1.73	0.74	OQ082222	>TdelSat146-62<br>ATCATTTAGAGATAACATGTTACTGTCAGTTACGAACCAATAGAAATAAACAAAT<br>TAAAACT
TdelSat147-275	0.002	3.74	0.73	OQ082223	>TdelSat147-275<br>CTGGCTGTTAATAAGGTTCACAATTTATACACTCTAATTATTGATATGCAGCCAAT<br>AAATTCTTTGTGAAACTAAAAAAATTATCATCATGTTGGGACCAAAGACACCAAA<br>AATAGATCTACAAAGAAGATTCAAGTTACTAAAAATGGAAATATTCACCACTATA<br>CATAATATTCAGTGATTGTAACCCTTGATAAGAACACGTGAAGTGTTGAAACGTT<br>GGGTTTGTTAAAAAGATAATAATGTAAAATTATAAATTAAAAATATAAATTCAT
TdelSat148-53	0.001	7.71	0.62	OQ082224	>TdelSat148-53<br>CAGTAAGAGAAAGTGGGGAAATGTATCACATTCAGTTGGCAAGAACTAAATTG
TdelSat149-26	0.001	4.79	0.58	OQ082225	>TdelSat149-26<br>ATAGTGGGTAACAGTGCTACACATGT
TdelSat150-56	0.001	0.06	0.66	OQ082226	>TdelSat150-56<br>TGTTGAGGTTGGTTTGCCTCTACTAACTATGCAAATATAACATATTGTAAAATGGG
TdelSat151-149	0.001	0.52	0.70	OQ082227	>TdelSat151-149<br>GTTTAGTTTATAAAGGGCACTGTTTAGTAATACCTAGCAAATTTTAAAGGAATATT<br>TACAAAACGGATGTGGCCTCTTAAAACCAAAATTGTAAATGTAAAACCCCAGCCT<br>TACAATAAAGGTGAGAAATGAAATTAATATTATCAATG
TdelSat152-50	0.001	0.65	0.76	OQ082228	>TdelSat152-50<br>AATTAATAAAACTAGACATATACTTGTAGAATTTCACCATTTGTACTTAC
TdelSat153-75	0.001	1.86	0.47	OQ082229	>TdelSat153-75<br>ACAAGTGGGCACTCACCTTGCCCACGTAGAGATGTTGGTTAGTACCTAGCCAGGC<br>CGTTAGTAGAAGTGTAGCCC
TdelSat154-45	0.001	1.67	0.62	OQ082230	>TdelSat154-45<br>CCATATAATGTTGGGTACTATACCTTTATGGTGTAGACACCTTCA
TdelSat155-89	0.001	2.24	0.62	OQ082231	>TdelSat155-89<br>ACCAGACCAAAAAGATATTCTTCTTCCATAAAAAAAAATACTCCACATATGCTTCC<br>TCTCACGACCTAAGATAATTCACTCTGGACCGCT

TdelSat156-78	0.001	1.6	0.67	OQ082232	>TdelSat156-78<br>GGAAAATTTTTGTTGAAATTTACTGCATCAAATCTTTATTTCTTACTTCTTCCAAGG<br>ATCCACTAAGGCTCTCAGTCA
TdelSat157-51	0.001	5.78	0.75	OQ082233	>TdelSat157-51<br>GACAATACTGAAATATATATCGCTAGTAAATGGCAAATCTAAAGTTATAAA
TdelSat158-134	0.001	5.99	0.67	OQ082234	>TdelSat158-134<br>ACTTCAATTTGCATGTTTGAAGATAATTTACCAATTGGAATGTTAATAGTTGAAGT<br>AAGAGAGATTACTGGGGATGCTTCAAAACTGATTTCGCTTTCTATTACTATAGTCT<br>CTATGTGTGAGAACTAACTGTG
TdelSat159-241	0.001	7.38	0.75	OQ082235	>TdelSat159-241<br>AATGTATTGATAATGAATTTCTAAACGAAAATTCAACAATAAACGTGTTAGGATT<br>TACGGCAAATATTTTACAATTAAATGCAACACGAAATCCAACAAAATTCAATAAT<br>TCTGTTTACAACGAAACAGACAAAAATACATTAGATTTACGGCAAATATTTAACA<br>AAAATGTATAGCAAATAAATGCAACACGAAATCTAACAAAAGAATTAATAATTC<br>TGTTTACAACGAAACAGACAAA
TdelSat160-49	0.001	6.31	0.84	OQ082236	>TdelSat160-49<br>TAATGACGTTCTTGATATAAATACAATATTTTAAAATTGAATAAAATTC
**TOTAL**	**53.920**				
