# Supplementary material for: Making the Genome Huge: The Case of Triatoma delpontei, a Triatominae Species with More than 50% of Its Genome Full of Satellite DNA
Source: Genes (Basel). 2023 Jan 31;14(2):371. doi: 10.3390/genes14020371 (PMC9957312; doi:10.3390/genes14020371)
Supplement: Supplementary file 1 [file genes-14-00371-s001.zip › Table S2 22-12-2022.pdf]

Supplementary Table S2. Data of the satDNA families found in *Triatoma infestans*: genome abundances (%), divergences, A+T contents, and consensus sequences. Table includes the satDNAs families previously characterized in Pita et al. (2017) (Old families) and the new satDNAs detected in this study (New families).

Old families	New families	Genome abundance (Andean) (%)	Divergence (Andean)	Genome abundance (non-Andean) (%)	Divergence (non-Andean)	A+T	Accesion number	Consensus sequence
TinfSat01-33		11.671	7.31	2.099	7.28	0.67		Pita et al. (2017)
TinfSat02-79		9.300	3.71	11.127	4.79	0.59		Pita et al. (2017)
TinfSat03-4		6.236	4.24	3.202	4.15	0.75		Pita et al. (2017)
TinfSat04-1000		4.927	12.13	6.473	13.26	0.67		Pita et al. (2017)
TinfSat05-4		2.627	0.64	0.734	1.29	0.75		Pita et al. (2017)
	TinfSat43-242	0.679	11.28	0.697	11.63	0.48	OQ082237	>TinfSat43-242<br>GGCGCGTCAACATCTATGGTCATTGGCGCCCGGAGCCTTCT<br>CAGTGCACTGCCGGCCTCCGGACAGTGTTCTCTCGTTACAG<br>ACAACACTGTCCATCACCGGACAGTGAGCTGTAGAGGCTC<br>CTAGGAAGAAAAAATCTAAGGGCAGCACGCTGGACTCGA<br>ACCCGGGACCTCCGCGTGGAGTCCAGTCGCTAACCACTGA<br>GCTAATCCCTTCCCTTTTTTTTTTTTTTTTTTTTAATTTAA
	TinfSat44-8	0.405	18.22	0.126	18.65	0.63		>TinfSat44-8<br>GATAGTTG
	TinfSat45-94	0.318	7.81	0.342	8.15	0.48	OQ082238	>TinfSat45-94<br>CGGAAAATGTCTCTGATGCTTTCCAGCTCTGCGGAACCAA<br>CCACCACAAAACGCCCGAAACCGGTCGATGACCACAAAA<br>TGAAGCAGGCTACCA
	TinfSat46-131	0.264	17.95	0.358	19	0.65	OQ082239	>TinfSat46-131<br>GGGGTTCTGGGGGAACAGTAGCGTTATGCTTTCCTCTAAAG<br>TACCACTTCAGTATAATATATGGTTTTACCACCTACATAAT<br>ATACCTATCACTTTTATTTGAAACATTTTCCCGGGAAAATT<br>GATTTTTT
TinfSat06-181		0.204	11.49	0.129	11.94	0.76		Pita et al. (2017)
TinfSat08-239		0.196	12.36	0.184	14.34	0.59		Pita et al. (2017)

	TinfSat47-87	0.133	15.08	0.135	15.44	0.59	OQ082240	>TinfSat47-87<br>TACCTAAACGGAATACCAAGTTTGAGCTTCCTGCAATTTTC<br>GTTTTTGGATTTTTCCCGAAATCTTCGTGTCTAGATGCGGTG<br>GTAG
TinfSat36-10		0.066	10.26	0.010	10.06	0.50		Pita et al. (2017)
TinfSat09-113		0.064	3.94	0.358	11.18	0.78		Pita et al. (2017)
TinfSat07-10		0.058	7.56	0.008	7.66	0.40		Pita et al. (2017)
TinfSat12-84		0.049	19.53	0.047	20.69	0.64		Pita et al. (2017)
	TinfSat48-120	0.047	11.04	0.048	11.55	0.56	OQ082241	>TinfSat48-120<br>TATTACATCATCAGGTTCTCCCACTCTAGCCTGCTTAAGTC<br>AGAGAGTACCAGAGAGGGTCTTTTCTCTCTTAATTCTCTCG<br>TCTGTCTGCTCTCTCTTGCTGATGTAATTCTCTCTTGC
	TinfSat49-94	0.033	16.31	0.045	16.68	0.70	OQ082242	>TinfSat49-94<br>GGAGTGTATGGTTTCCTCAAAAAAGTCATTTTATTAAGTTT<br>AGATTCTCCAATTGATATAAAATTGAAGACAATTAAACAT<br>TTTGGAGAGAGGG
TinfSat10-53		0.033	4.16	0.024	3.54	0.58		Pita et al. (2017)
	TinfSat50-63	0.032	13.4	0.011	15.71	0.43	OQ082243	>TinfSat50-63<br>CGTGGCTTAAGTGCTCGTCCAGTTCGATGATTGGTCAGTTG<br>GAGAGGGGTGGGGGTAAGTGGG
	TinfSat51-137	0.032	18.4	0.041	21.03	0.61	OQ082244	>TinfSat51-137<br>TAACAGGAGTAAGAGAAAGGCGTGTGAGTGTCCCCCACC<br>ACCTCCCCCTCCTAACATCACCAAACTATATGTTAGGACA<br>AATGAGTAAAATTGGAAGTTTAAAGTAAATTCGTCACTTT<br>ATATATATCTCATTGTAG
TinfSat14-147		0.031	11.6	0.032	17.6	0.64		Pita et al. (2017)
	TinfSat52-334	0.028	4.72	0.150	4.36	0.70	OQ082245	>TinfSat52-334<br>TAAACAGGCATAGGATATTATGCGCTAAAAAACATGTTAC<br>TTTTTAATCGAAGAATATTGGAACAAGTTAAACCATTATAA<br>GTTTCTAATCTGGGGAAATGATTGCATTATATCCAAATTCG<br>AAATCTTCTGTTTTGTAGCCATTGTCTAACGTCTATTAGTCC<br>AAGAAAAACACAGATATACAATAGAATATAAACATATAT<br>TGCACTAAAACATTCTTAAGTTATCGCATGGCAATTATTGT<br>GGATTGCAATAACAGTTTTGCTAGTAATTTCCAAGTTCTTT<br>CCTCAACCTTTCTAAACATGTAACAACTTGTTCTACTGTCTT<br>ACATTCT

	TinfSat53-128	0.027	18.91	0.035	20.23	0.65	OQ082246	>TinfSat53-128<br>TTTCAATTACTCATATGTCCTAATATAAATATACAGGTGGT<br>GGGAGACAAGTGCCAGCCTAGCAACACAGACAGGTACAG<br>AATGATTACATTGTAGATAGCCAATAATATTGAACAAACA<br>CATTTGAA
	TinfSat54-128	0.027	15.2	0.025	18.41	0.61	OQ082247	>TinfSat54-128<br>AGATTGTGTGTTCACAAACACACCGACTATTAGCTCCGCAT<br>TCTTACATTGCACATATATAGGCCAGATACGGTGGCAGTGT<br>CTAAACAGAATACCATATTTGAGCTTCCTGTAATTTTTCGTT<br>ATTG
	TinfSat55-89	0.026	13.66	0.031	16.21	0.71	OQ082248	>TinfSat55-89<br>TAACTGCAGTGAGAGTAAGTTGTGTGAATGTTTTTCACACC<br>ATTGAGTAACATTCCATTATAAAGAAAATTTGTCAATTGTA<br>ATTGTAT
TinfSat13-147		0.025	6.57	0.014	6.8	0.59		Pita et al. (2017)
	TinfSat56-118	0.023	14.48	0.026	16.71	0.64	OQ082249	>TinfSat56-118<br>TAACTGGAGTAAGAGAAAGTCGTGTGAATATTTCCCACCA<br>GTTTTACTTTGAAACACCTGGTAGCAAGTTAATGGTTCCGT<br>TTACTGCTGTACAAGCGATTTAACAGTTAAATTGTAT
TinfSat21-38		0.021	10.16	0.007	10.58	0.63		Pita et al. (2017)
TinfSat39-5		0.021	3.39	0.011	4.67	0.60		Pita et al. (2017)
TinfSat11-85		0.020	4.68	0.020	4.43	0.71		Pita et al. (2017)
TinfSat19-104		0.020	5.79	0.002	6.36	0.63		Pita et al. (2017)
TinfSat17-118		0.017	4.04	0.003	3.99	0.60		Pita et al. (2017)
	TinfSat57-164	0.017	7.43	0.006	7.62	0.62	OQ082250	>TinfSat57-164<br>AATTGTTTGGAGTTAAATAAGGTGAGTGGCCAGTGCTGCT<br>GTGCTGGTCACGTTTGTAGCTTTGTTTAACATAATCCTTCAA<br>TTCTACCCACAGCTTGCCGTTATGTTAGTTCTTAGGAATTA<br>GCATATATATGCGGCGCCCTTTTATATATATATTAGTGTAG
	TinfSat58-96	0.016	7.24	0.010	8.14	0.68	OQ082251	>TinfSat58-96<br>GGCAAGAAAGGTAATGAGTACAAAGTTTAATTTTTACATA<br>ATAGGATTAAGTATTACTAAACATGCTAATAAGACCTGTG<br>GTAACATGTCCGGACA

	TinfSat59-132	0.015	3.92	0.004	4.32	0.56	OQ082252	>TinfSat59-132<br>GAGACACGAGTTTCCCTTATTACAATTACAGTTATAGTTTG<br>GTAAATTAACACAGAGTGGTGGGGGTACATGTAGCAGGTA<br>GCAGCTAGTACTTTACCCGGTCCACAATACGCCACCTCCAT<br>CACACTGAAT
TinfSat33-372		0.015	6.59	0.017	5.98	0.58		Pita et al. (2017)
TinfSat16-49		0.015	5.2	0.004	6.1	0.73		Pita et al. (2017)
TinfSat20-46		0.014	6.16	0.002	16.64	0.70		Pita et al. (2017)
TinfSat18-102		0.014	8.94	0.006	23.45	0.63		Pita et al. (2017)
	TinfSat60-33	0.014	10.3	0.015	8.8	0.70	OQ082253	>TinfSat60-33<br>TCTACACACATAAATACCTATCACTGTATACTA
TinfSat42-112		0.014	15.5	0.017	14.36	0.63		Pita et al. (2017)
TinfSat30-58		0.013	4.63	0.005	4.53	0.66		Pita et al. (2017)
TinfSat15-99		0.013	7.69	0.011	8.44	0.69		Pita et al. (2017)
TinfSat23-51		0.012	3.61	0.007	3.43	0.67		Pita et al. (2017)
TinfSat24-112		0.012	4.75	0.010	4.38	0.58		Pita et al. (2017)
TinfSat22-64		0.012	2.46	0.002	3.15	0.78		Pita et al. (2017)
	TinfSat61-89	0.012	6.35	0.007	8.82	0.71	OQ082254	>TinfSat61-89<br>CAGTTGGGTGTAATGTTCAAGAAAAATACTAAATACTCTG<br>TTTTCAGCAAAATACATCGGAAAATTTTATTTTAGTACAAC<br>ACACACTT
TinfSat25-62		0.012	8.95	0.019	8.56	0.56		Pita et al. (2017)
	TinfSat62-120	0.011	12.05	0.019	11.58	0.61	OQ082255	>TinfSat62-120<br>TAAGAGAAAGTTGAGTGAGTATTTCACACCAGTTTTTAGTC<br>ACACTCAAGAACTGTCAGCAATGTGTGCAATAAAAATGCA<br>CTAACACAACCAGTGTGTCCATGCTAGTACTAACTGCAG
TinfSat26-53		0.011	9.73	0.002	8.97	0.62		Pita et al. (2017)

	TinfSat63-237	0.010	2.94	0.002	9.85	0.74	OQ082256	>TinfSat63-237<br>AATATATTTATAATGAATTTCTAAAGAAAATTCGGAAAAC<br>ACAATGCGTTAGGTTAAAGGCAAATATTTAACCAAAATGC<br>AACACGAAATCTAACAAAAGAATAATTCTGTTTACTACGA<br>AACAGACGACAAAAATACATTAGATTTACGGCAAATATTT<br>AACAAAAATGTATAGCAAATAAATGCAACACGAAATCTA<br>ACAAAAAGAATAATTCTGTTTACTACGAAACAGACAAA
	TinfSat64-88	0.009	8.71	0.003	5.44	0.73	OQ082257	>TinfSat64-88<br>AGGTGAGTGTTGTACTAAAATGAAATTTTTAGATGAATTTT<br>GGTGAAAATAGTGGATTTAGTATTTTATTGTACATTAAACG<br>CAACTG
	TinfSat65-103	0.009	6.44	0.007	10.6	0.67	OQ082258	>TinfSat65-103<br>TATAGCCTTTTCGAAACAATCGCGAAAAAAAACATTCAGT<br>TACTTCTTTCAGGTAGAAGTTACAGACCATTATGCGTGTAT<br>TTAGTGTGTGAAGTAATGTTTT
TinfSat31-71		0.009	9.18	0.001	10.47	0.70		Pita et al. (2017)
TinfSat27-47		0.008	5.57	0.008	5.47	0.62		Pita et al. (2017)
	TinfSat66-161	0.008	8.29	0.011	8.21	0.61	OQ082259	>TinfSat66-161<br>TAGTGCTGCGTTTGTGCCGGCGATTCACCAAATTTCATCGT<br>TTGTGCCGTAAAATTGACAAAGATATTGAAGAAAAAAAGT<br>GGCCAGTCCAACTTGACGTCAAGTTAGACTGGCCACAAAA<br>TTTCTATTCACTTTTTTAAACTTTTCTCTTTGCCAATCGT
TinfSat28-46		0.008	3.87	0.009	4.22	0.65		Pita et al. (2017)
TinfSat32-52		0.008	5.97	0.012	6.37	0.60		Pita et al. (2017)
	TinfSat67-114	0.008	4.55	0.007	4.78	0.68	OQ082260	>TinfSat67-114<br>TATCGTTACAACTATTATTAATCTCATTACAGAGAATCTCT<br>ATATCGTTTTTAGCAGAACATTCTACAATAACTCCGCCATT<br>AGAGATTCCCTTAATGTTTTCTACACCCAATT
TinfSat29-87		0.008	2.42	0.007	2.68	0.64		Pita et al. (2017)
	TinfSat68-136	0.007	13	0.008	15.19	0.65	OQ082261	>TinfSat68-136<br>TTTCAAATTTGCTCATTTGTCATGACATAAATATACAAATA<br>GTGGGGGAACAAGAGCCATCCAAACTACCTATAGAGGCA<br>GCTACAGAATGAGTACATTGTAGGGAGGCCAATTATAGTA<br>AACAAACATATTTGAA
	TinfSat69-58	0.007	1.87	0.006	1.85	0.71	OQ082262	>TinfSat69-58<br>TATGAGGAAGGAGAAATCATTAACTGTAACTCCACAAGA<br>AACATAAAGAAAGAAAATT

	TinfSat70-170	0.006	11.64	0.004	13.72	0.70	OQ082263	>TinfSat70-170<br>TTAAATTTACTACAATTTATGTTCTGTGGTATTTTTAGATAG<br>GACTACTCCTTAAGGAGATATTGCTGAAATACCAGTTAGG<br>TGGAAGGTGAAGCTCCCAATATTTTAGCTCATATCTTGGTT<br>AATATTAACTCTATGATAAAACTGTATAGAACTAAAATTGT<br>AGAGAA
	TinfSat71-99	0.006	7.56	0.002	12.27	0.59	OQ082264	>TinfSat71-99<br>TTTGTAGGTTGGCTGGCTGAAGGTAAACAGGTACAAGAAA<br>CCGTTAGATTAAGACAATATACTTATTGCCATTCCCCCCCC<br>CCCTTAGAATAGAAGAAA
	TinfSat72-49	0.005	3.67	< 0.001	2.32	0.69	OQ082265	>TinfSat72-49<br>GCAAACTATATAGTGAGATGATATGAGGGCAAGTATAACT<br>ATATTTTGT
	TinfSat73-44	0.005	3.45	0.007	4.14	0.66	OQ082266	>TinfSat73-44<br>TTTGGTTCTCACATAAATACAGAACATTCGCCGTTATTCAG<br>ATA
	TinfSat74-147	0.005	13.65	0.007	12.82	0.72	OQ082267	>TinfSat74-147<br>AAGATAATCTTCTTGTAATTTTATTGTTTCATCTGTTTTTGTT<br>TTTATCATGAGTTTAACTGTATCGATCTTTCTATGAATGGTT<br>TGATCACTTTTAAACTTTTTGGAAAAACTTTTTGGAGGATA<br>TACTCTTTCAGGTATTCCTGG
	TinfSat75-167	0.005	2.89	N.D.	-	0.64	OQ082268	>TinfSat75-167<br>GTGTGAGGAGACGACTGCTAAAATTGATGTGCTTGTAACT<br>CCTAAAATTCTCAAGATATTATGCCGATATTTGGTCAAAAT<br>ATACTACACTAGTATAGCATTAAACGAGCCAAAATTCACT<br>TAGATCTGAGCAACGGAAGTGGGTTTTTTTTGCGGGAAATT
TinfSat34-28		0.005	7.46	0.006	8.32	0.61		Pita et al. (2017)
	TinfSat76-70	0.005	3.78	0.006	3.96	0.63	OQ082269	>TinfSat76-70<br>TGTTTTGAGATCCTTGAGTGGAATGTTTTCAGCCTTGATGA<br>CTCTATGTTTCTGTGTATGTGTGAATAGA
	TinfSat77-133	0.004	10.19	N.D.	-	0.68	OQ082270	>TinfSat77-133<br>AATTGGAGTACTGTAAAATAGAAAACGGATCCGTTTACCA<br>TTGAGGCAACGTTGTTTTTTTGTGATGAAAAATCTACGATA<br>ATAGCATTGCAAAGCAACTTTTTCTCAAATCCATTTTGAAA<br>GATTTACAGTA

	TinfSat78-348	0.004	3.4	0.004	4.48	0.54	OQ082271	>TinfSat78-348<br>GTGAAGCCTATCACAACTTCGGCAACCTACACCGAAGAGT<br>TCTCAACAATACAATCTTCATCACCTCATTCCACAGAACCA<br>ACATCAGAAAGTTATACTACTAACAGCCATCGCACAACTG<br>AATCAGAATCTACACCAATCACACAATCAAGCCCAGAGAT<br>ACGTACCACATTTACGTCCGCGTCCCCATCTACTGAGAAC<br>AGTGCAACAACAACCGCCACCGCGCACACATCACAGCCT<br>AGCTCTGAAACATTCAATACAATTAAATACACTACGGGTC<br>CGGGCACTGAAACCTATTCAACAATAGTGGGTTCAGGCTC<br>GACATCTCACAGCGAAGGTTATACTTCC
	TinfSat79-72	0.003	6.97	0.001	6.48	0.65	OQ082272	>TinfSat79-72<br>TCCAGCCACACTAACTTCAAATATTAGAATAGTTTTCGCCA<br>TTAAACCATATCACCAATCAACGTTCTAACA
	TinfSat80-61	0.003	6.82	0.005	8.43	0.67	OQ082273	>TinfSat80-61<br>TATTAAGTGACTTTCTCTTACTGTAATTCTCACAGTCGAGTC<br>AATTTTGAAAATGAAAGGC
	TinfSat81-51	0.003	14.36	0.003	14.56	0.65	OQ082274	>TinfSat81-51<br>CTTTCTCAGGAAAAAGTTCTTCTTCCCTTCCCCAAAACTTCT<br>TTCAATAAA
TinfSat35-35		0.002	2.99	0.007	2.91	0.69		Pita et al. (2017)
	TinfSat82-207	0.002	9.59	0.002	11.44	0.63	OQ082275	>TinfSat82-207<br>TCAGTGGGCTTTTCGGTTACTCTAGTTGTAAATTCAGTATCA<br>TGAATTGTAGATTCTTCTGTGATATGTGTAGTCGGTTGCTCA<br>GTGTTTGTTGTAAATTCTTCAGCGGTTTGTGCATCCGTGCTA<br>GATCCTATGTAAGATGCAGTATGGTCATTTGTGTAATATTT<br>CGGGTTATTTGGTGTATTCGGTTTGAATGTTGTTGTAATA
	TinfSat83-235	0.002	2.36	0.005	2.23	0.70	OQ082276	>TinfSat83-235<br>AGAGATTGTTATATTCTTGTTAGTTTATAGCTTTGGTAATAT<br>TTTATCGAATTGAATGTTAATTACATAGATGCAGTAGAGAT<br>TGTCACAACTTGCAACATTCTTGATACTTTACACCTTTTACA<br>GTTTTGCTACTTTTTGTGGTTTATACCCTTACCATATCACAA<br>TTGCAAAAAAATAATAATGTTTGCAATATTTTACCAAGAGTT<br>TGAACGTTAATTGCCGTTACATGCGGA

TinfSat843-<br>379	0.001	7.3	0.002	8.71	0.69	OQ082277	>TinfSat84-379<br>ACCTCACACAAAATATACCATATACTATCCTTTCCATGCGA<br>AGAATTGAGACGAAATATGGGGCTTCGGTATTAGTTAGTA<br>TGAGAAACAGTACTGACGACGAAGAAATTCGTTGTTTTTT<br>ACCCAAAAATTTTACAGAACAGATCACAGACGACGTCGTT<br>AAGGATTATAAACATTATAAACCATCATCATTATCACTTAT<br>ATATAGGGGGTTGAACCCTAATAAATCTTTCAATATTCATT<br>TCGTTTAATGGAAGAACAGATTATTGAGCTTGATCAGTATG<br>TATGTTTTAAAGTTAAAATGTACATAACTGATGAAGGTTTC<br>TTATGTCATGATTGTGCGCAACAATTAGAAGTAGAAGATA<br>AATTAAATATAAAA
TinfSat85-83	0.001	4.27	0.002	4.88	0.71	OQ082278	>TinfSat85-83<br>AAAAACATATACATATCACCCCTGTACTATTGATTAAGCTC<br>CCAAATCTCTTTAACATAACTTTACACTATTACTATACTTA<br>G
TinfSat86-114	0.001	7.29	0.001	7.46	0.57	OQ082279	>TinfSat86-114<br>GTAGAGGTGTCCGTGATGTCATGAAGATGGGAAATAATTG<br>GCAGAGAGGGTTAGGGTGCGTCCTGACTCTGGCGAGTAGT<br>TTTTTCAGTCTTAGCTTTAATAAAGTCAGTTATA
TinfSat87-98	0.001	17.91	0.001	17.1	0.60	OQ082280	>TinfSat87-98<br>ACCAGACCAATTACATAAATACTATAACGACCAATACTCC<br>ACATATACGCTTTCTCTTACAACCCAAGACAATTCACTCTG<br>GACCGCTC
TinfSat88-121	0.001	17.31	0.006	9.21	0.67	OQ082281	>TinfSat88-121<br>ATCCACTGAACAAACCAAATAGCATTTGAAATTTAACACA<br>TTAGTCCTAACATTAAATATAGTGGGGGATCAACACAGGC<br>GGTATAATTTGAATACATTACTGTCCTATAGATAGCGATAA
TinfSat89-49	0.001	8.62	0.002	8.06	0.86	OQ082282	>TinfSat89-49<br>TAATGATGTTCTTGATATAAATACAATATTTTAAAATTGAA<br>TAAAATTC
TinfSat90-29	0.001	3.83	0.001	5.1	0.59	OQ082283	>TinfSat90-29<br>CTGTACAGAATAGCTAAGTGTGTGTGTGA
TinfSat91-238	0.001	9.79	0.001	8.89	0.74	OQ082284	>TinfSat91-238<br>CCAAACAACAATAATATGAACGGTTAACGGTCGTTTTTTTC<br>TATCAGTTATCATTCAATCAGTTACAAAGTTTTACACTTTA<br>ACATTTTTTTAATAAACTATTTGCATGTTTTCCAATTATTTC<br>ATTATTATTTTCTCTTTAAACACTATATCCAACTTGGCACAT<br>TCCTATCTAACAAAGTTTTTATATGTTTTCAATTTATATTTA<br>ATTTAAACACTACACCCTCCTTGGAACATT

TinfSat40-73		< 0.001	5.28	< 0.001	6.34	0.60		Pita et al. (2017)
	TinfSat92-61	< 0.001	4.28	0.003	3.54	0.72	OQ082285	>TinfSat92-61<br>TTGCCACTAACAGCAATAAGAGAAAGTAAAAAAGAAGCA<br>AAT
	TinfSat93-102	< 0.001	5.83	< 0.001	7.22	0.65	OQ082286	>TinfSat93-102<br>GGAATTAAAATAGAGTCCGGTTACAAACGGTAATGAATTA<br>TTTTTTTCTAAACCACTATAACTAGGGCTAGTAGCAACCAG<br>CACTGTCTATCAGCTCATTTT
	TinfSat94-89	< 0.001	10.09	< 0.001	19.98	0.71	OQ082287	>TinfSat94-89<br>TAACTGCAGTGAGAGTAAGTTGTGTGAATGTTTTTCACACC<br>ATTGAGTAACATTCCATTATAAAGAAAATTTGTCAATTGTA<br>ATTGTAT
	TinfSat95-7	< 0.001	11.88	0.005	14.63	0.86	OQ082288	>TinfSat95-7<br>ACAATTT
	TinfSat96-101	< 0.001	16.29	0.010	12.33	0.70	OQ082289	>TinfSat96-101<br>ATATTTGTAGCAAAATAGTTAATTCTAAAGTAGGGGGAGG<br>AATGCAGAGAGCAGTCAAATTCATTTGAACATAAACATCA<br>TTTATTATGTTAACTTACACT
TinfSat37-314		N.D.	-	0.037	3.48	0.45		Pita et al. (2017)
TinfSat38-315		N.D.	-	0.031	9.36	0.62		Pita et al. (2017)
TinfSat41-101		N.D.	-	0.006	2.34	0.67		Pita et al. (2017)
**TOTAL**		**38.078**		**26.891**				

N.D.: non detected
